# Solid-phase synthesis and structural characterisation of phosphoroselenolate-modified DNA: a backbone analogue which does not impose conformational bias and facilitates SAD X-ray crystallography†
†Electronic supplementary information (ESI) available: PDB ID: 6S7D. See DOI: 10.1039/c9sc04098f


**DOI:** 10.1039/c9sc04098f

**Published:** 2019-10-11

**Authors:** Patrick F. Conlon, Olga Eguaogie, Jordan J. Wilson, Jamie S. T. Sweet, Julian Steinhoegl, Klaudia Englert, Oliver G. A. Hancox, Christopher J. Law, Sarah A. Allman, James H. R. Tucker, James P. Hall, Joseph S. Vyle

**Affiliations:** a School of Chemistry and Chemical Engineering , Queen's University Belfast , David Keir Building, Stranmillis Road , Belfast , BT9 5AG , UK . Email: j.vyle@qub.ac.uk; b Reading School of Pharmacy , University of Reading , Whiteknights , Reading RG6 6AP , UK . Email: james.hall@reading.ac.uk; c School of Chemistry , University of Birmingham , Edgbaston , Birmingham B15 2TT , UK; d School of Biological Sciences , Queen's University Belfast , 15 Chlorine Gardens , Belfast BT9 5AH , UK; e Diamond Light Source , Chilton , Didcot , Oxfordshire OX11 0DE , UK

## Abstract

Oligodeoxynucleotides incorporating internucleotide phosphoroselenolate linkages have been prepared under solid-phase synthesis conditions using dimer phosphoramidites. These dimers were constructed following the high yielding Michaelis–Arbuzov (M–A) reaction of nucleoside *H*-phosphonate derivatives with 5′-deoxythymidine-5′-selenocyanate and subsequent phosphitylation. Efficient coupling of the dimer phosphoramidites to solid-supported substrates was observed under both manual and automated conditions and required only minor modifications to the standard DNA synthesis cycle. In a further demonstration of the utility of M–A chemistry, the support-bound selenonucleoside was reacted with an *H*-phosphonate and then chain extended using phosphoramidite chemistry. Following initial unmasking of methyl-protected phosphoroselenolate diesters, pure oligodeoxynucleotides were isolated using standard deprotection and purification procedures and subsequently characterised by mass spectrometry and circular dichroism. The CD spectra of both modified and native duplexes derived from self-complementary sequences with A-form, B-form or mixed conformational preferences were essentially superimposable. These sequences were also used to study the effect of the modification upon duplex stability which showed context-dependent destabilisation (–0.4 to –3.1 °C per phosphoroselenolate) when introduced at the 5′-termini of A-form or mixed duplexes or at juxtaposed central loci within a B-form duplex (–1.0 °C per modification). As found with other nucleic acids incorporating selenium, expeditious crystallisation of a modified decanucleotide A-form duplex was observed and the structure solved to a resolution of 1.45 Å. The DNA structure adjacent to the modification was not significantly perturbed. The phosphoroselenolate linkage was found to impart resistance to nuclease activity.

## Abstract 2

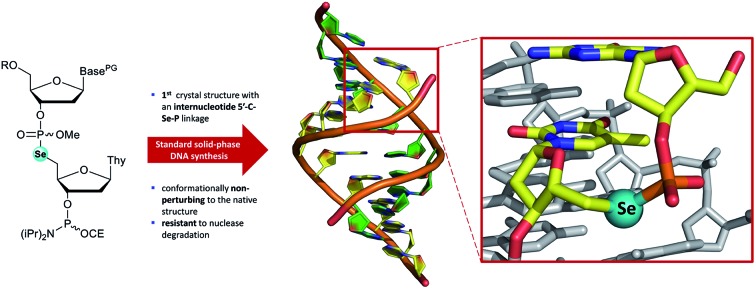
Stable selenium-modified DNA which maintains the native tertiary structure has been prepared under automated conditions enabling SAD X-ray crystallography.

## Introduction

**Fig. 1:**
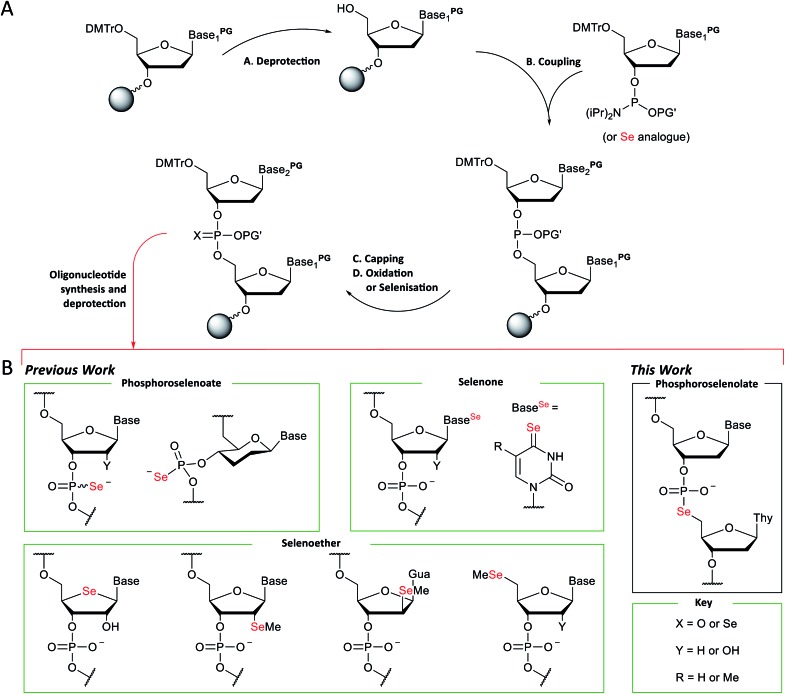
(A) General scheme for the solid-phase synthesis of oligonucleotides using phosphoramidite chemistry. (B) Examples of selenium-modified nucleic acid analogues prepared using this chemistry.

The automated solid-phase synthesis of oligonucleotides using phosphoramidite chemistry^1^ (Fig. 1A) is a platform technology which has been transformative in a wide range of applications^2^ including the measurement^3^,^4^ and manipulation^5^,^6^ of gene expression, DNA nanotechnology^7^,^8^ and data storage.^9^

Whilst simple Watson–Crick base-pairing rules can be used to programme both intramolecular and intermolecular assembly of three-dimensional structures from simple duplexes,^10^,^11^ the tertiary interactions of folded nucleic acids derived from more complex assemblies and their recognition, especially by nucleic acid binding proteins or small molecule effectors, typically requires structural elucidation at the atomic level.^12^,^13^ Characterisation of novel structural motifs including such complexes by X-ray crystallography is augmented by the introduction of an anomalous heavy atom scattering centre.^14^,^15^

Anomalous scattering of X-rays at the Se–K edge has been of considerable value in facilitating structural biology studies of proteins following the pioneering work of Hendrickson and co-workers using either selenomethionine^16^ or a high-affinity ligand.^17^ The molecular biology techniques required to engineer polypeptide sequences containing a mutated selenomethionine residue have now become routine and as this residue does not disrupt protein folding, it is widely used in MAD (multiple wavelength anomalous dispersion) or SAD (single wavelength anomalous diffraction) applications.^18^ Concurrent increased access to third generation synchrotron sources which generate more focussed, tunable X-rays of high brightness and stability coupled with automation of the screening of crystallisation conditions has engendered a considerable upsurge in protein crystal structure solution such that in the year 1990, 132 protein structures were deposited in the protein databank whereas in 2018 this number had risen to 10 306. Of the 137 000 protein crystal structures in the PDB repository by the end of 2018, over 10% had been solved using SAD or MAD methods. These methodologies are particularly important for “first in class” structures of proteins and nucleic acids, where new structural folds or ligand-induced distortions exclude using other approaches to structure solution such as molecular replacement (which relies upon prior knowledge of a structure based upon its sequence). However, the true impact of using Se-SAD and Se-MAD may never fully be known as once the structure of a new fold or motif has been determined, this can then be used as the basis for solving future structures without requiring anomalous data. SAD and MAD phasing is therefore a great facilitator of structural science and is used in drug development programmes worldwide, due to the expeditious ability to determine pharmacophore–target interactions. However, for a structure to be biologically “valid” the incorporation of a heavy atom (such as Se or Br) into the crystal needs to have little or no effect on the conformation or topology of the molecule, whilst also not modifying the molecular arrangement of any potential binding sites. This is a particular problem when working with nucleic acids as the most commonly used nucleotide analogues, which contain heavy atom groups, either alter the topology of the nucleic acid (shifting its conformation) or change the groups present in either the major or minor groove, both of which are binding sites for most DNA-targeting drugs.

Methodology for the synthesis of selenium-modified nucleic acid (Fig. 1B) sequences under solid-phase conditions was first described three decades ago.^19^ In this report, selenisation of support-bound *H*-phosphonate precursors required treatment every 3–4 hours over three or four days and gave mixtures of the diastereomeric phosphoroselenoates. Subsequently, more efficient selenisation protocols^20^,^21^ and stereoselective synthesis^22^ have been developed and this modification has enabled the solution of refractory structures such as homo-DNA.^23^ However, the sensitivity of intermediates during oligomerisation and short half-life of the final product (*ca.* 30 days under ambient conditions in aqueous solution) has limited their application, especially *in vivo*. Likewise, nucleobase analogues which incorporate a selenone function can be accessed using concise synthetic pathways^24^–^28^ but are sensitive towards oxidation, hydrolysis and photochemical degradation.

A third class of selenium-modified oligonucleotides in which selenoether functions have been introduced at the 2′,^29^–^32^ 4′,^33^,^34^ or terminal 5′-positions^35^ or appended to a nucleobase^36^ exhibit greater resilience towards the conditions typically employed during standard solid-phase oligonucleotide synthesis using phosphoramidite chemistry although mitigation of side-reactions arising from selenoether oxidation is required^37^,^38^ and replacement of an oxygen at a stereogenic centre provides additional synthetic demands, *e.g.*, up to 14-steps are required to prepare 4′-seleno-4′-deoxynucleosides from d-ribose.^39^,^40^ The 2′-selenomethyl ether analogues (especially pyrimidine derivatives) are considerably more accessible (requiring only six steps to prepare on large scale)^41^ and can also promote crystallisation of modified oligomers.^29^,^42^–^45^ However, although the furanoside ring of individual nucleosides modified in this fashion adopt a conformation typically found in B-form DNA,^46^ when incorporated into oligodeoxynucleotide duplexes, the location of the 2′-selenomethyl function in the minor groove provides conformational steering towards the formation of RNA-like A-form duplexes. Although the epimeric arabino-2′-selenomethyl analogue reverses this trend,^31^ full details of its preparation have not yet been reported and the steric bulk of both isomers can interfere with groove-binding interactions.^46^

Replacement of a bridging oxygen (3′ or 5′) within an internucleotide phosphate diester by selenium gives rise to phosphoroselenolate analogues. These are achiral and potentially more stable than the corresponding phosphoroselenoates but have been documented in only a very limited number of communications.^47^–^49^

Alkylation of the lead salt of *O*,*O*-diethylphosphoroselenoate was first reported in 1911 50 and this strategy has been applied to the preparation of internucleotide phosphoroselenolate linkages using 5′-halothymidine derivatives either in DMF^47^ or under aqueous conditions.^48^ In the former case, characterisation of the dimer product was equivocal indicating that perhaps both *O*- and Se-alkylation had occurred.^51^–^53^ Subsequently, Xu and Kool exploited related chemistry for the synthesis of oligomers incorporating this analogue following template-directed chemical ligation. However, consistent with the extreme sensitivity of phosphoroselenoate monoesters towards ariel oxidation in aqueous solution,^54^ incomplete reaction was observed. In contrast, Michaelis–Arbuzov (M–A) reaction of a nucleoside 3′-*H*-phosphonate with a 5′-selenocyanate enabled synthesis of a fully-characterised phosphoroselenolate-bridged dimer (TpSedT) with high efficiency.^49^

Herein, we describe the further developments to this methodology enabling the isolation of pure dinucleoside phosphoroselenolate triesters in good to excellent yields, their subsequent phosphitylation and efficient introduction into oligodeoxynucleotides under solid-phase conditions. Furthermore, we report a model DNA crystal structure incorporating this modification which evinces an A-form morphology with minimal perturbation of the backbone.

## Results and discussion

### Preparation of dinucleotide phosphoramidites

The protected M–A product from the synthesis of TpSedT^49^ appeared to be an attractive target as efficient dimer coupling has been reported during the solid-phase construction of oligonucleotides containing sulfur.^55^,^56^ Furthermore, this strategy would avoid the preparation and use of intermediates containing a labile chalcogen–P(iii) bond.^57^,^58^ We therefore sought to optimise the synthesis and isolation of the protected dinucleoside phosphoroselenolate triester from the corresponding selenocyanate.

**Scheme 1:**
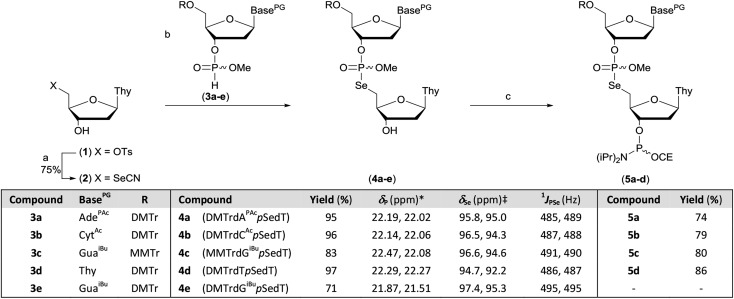
Preparation of dinucleotide phosphoramidites **5a–d**. Reagents and conditions: (a) (i) KSeCN (1.5 eq.), MeCN, MW power 20 W, 100 °C, 90 min; (ii) BnBr (0.6 eq.), MeOH, rt, 60 min. (b) **3a–e** (1.5 eq.), 2,6-lutidine (5 eq.), MeCN, rt, 30–60 min. (c) (i) ClP(OCE)NiPr_2_ (2 eq.), iPr_2_NEt (3 eq.), DCM, rt, 45 min; (ii) MeOH (1.5 eq.), rt, 15 min. *in MeCN (**4d**) or DCM, ‡in DMSO-*d*_6_. Key. Base^PG^. Ade^PAc^: 9-(*N*^6^-phenoxyacetyladeninyl). Cyt^Ac^: 1-(*N*^4^-acetylcytosinyl). Gua^iBu^: 9-(*N*^2^-isobutyrylguaninyl). Thy: 1-thyminyl. DMTr: 4,4′-dimethoxytrityl. MMTr: 4-methoxytrityl CE: 2-cyanoethyl. *p*: P(O)OMe. Ts: *p*-toluenesulfonyl.

5′-Deoxythymidine-5′-selenocyanate has been prepared on small scales in solution over 24 hours 59 or using liquid assisted grinding over 9–11 hours.^60^,^61^ In order to prepare this material more efficiently, we therefore examined the application of microwaves which has been reported to enhance the rate of *S*-selective alkylation of the thiocyanate anion.^62^ In the presence of excess potassium selenocyanate, clean transformation of 5′-tosylthymidine (**1**) into the corresponding 5′-selenocyanate (**2**) was observed within 90 minutes following irradiation (20 W, 100 °C) of the stirred reaction mixture in acetonitrile (Scheme 1). As previously observed,^60^ quenching of the excess selenocyanate with benzyl bromide greatly facilitated subsequent purification by silica gel chromatography and pure 5′-deoxythymidine 5′-selenocyanate (**2**) was isolated in 75% yield.

During optimisation of both the reaction conditions and subsequent purification of the M–A product derived from **2** and cyanoethyl-protected nucleoside 3′-*H*-phosphonate diesters, several issues became apparent. Specifically, the neutral silylating agent, *N*,*O*-bis(trimethylsilyl)acetamide (BSA), which served both to render the selenonucleoside soluble in chloroform and enhance the rate of the M–A reaction also promoted decyanoethylation of both the starting material and the product. Furthermore, subsequent quenching of the reaction following addition of water resulted in detritylation in the absence of a strong organic base but if such a base was used during work-up, further decyanoethylation of the phosphoroselenolate was evident.^63^

In order to ameliorate issues associated with β-elimination of the cyanoethyl function, methyl-protection of the phosphate was adopted. This provides greater resilience towards phosphate deprotection in the presence of stronger organic bases such as triethylamine and has recently been utilised for the preparation of di- and trinucleotide phosphoramidites.^63^–^65^ The methyl-protected 3′-*H*-phosphonates (**3a–e**) were prepared from the corresponding phosphoramidites under modified literature conditions.^66^ Following extractive work-up, the materials were pure by ^31^P NMR.

However, in our hands, these *H*-phosphonates were less stable towards dephosphonylation than those derived from the cyanoethyl-protected precursors and low levels of the nucleoside congeners were identified by TLC as contaminants. Thus, once isolated, the solids were stored at –20 °C and used within 24 hours. To accommodate the presence of these impurities, excess *H*-phosphonate (1.5 eq.) was used in the M–A reaction. In a further change to the previous published procedure, acetonitrile was used as solvent and in the presence of 2,6-lutidine, nucleoside selenocyanate **2** could be dissolved in this mixture under gentle heating without silylation. Addition of this solution to thymidine *H*-phosphonate (**3d**) in acetonitrile at room temperature gave complete and clean M–A reaction within 30 minutes. We note that these conditions contrast with those typically employed to promote such reactions with chalcogen electrophiles following conversion of the *H*-phosphonate into the corresponding phosphite tautomer in the presence of a silylating agent or a strong base. The protected dimers (**4a–e**) were isolated as mixtures of diastereoisomers following silica gel column chromatography. Phosphitylation of the dimers under modified literature conditions^67^ enabled isolation of the corresponding phosphoramidites derived from 5′-dimethoxytrityl-protected dA (**5a**), dC (**5b**) or T (**5d**) and 5′-monomethoxytrityl-protected dG (**5c**) in greater than 70% yields. During attempted processing of **4e** (DMTrdG^iBu^*p*SedT) under the same conditions, detritylation was evident and therefore further reaction to the corresponding phosphoramidite was not attempted.

**Fig. 2:**
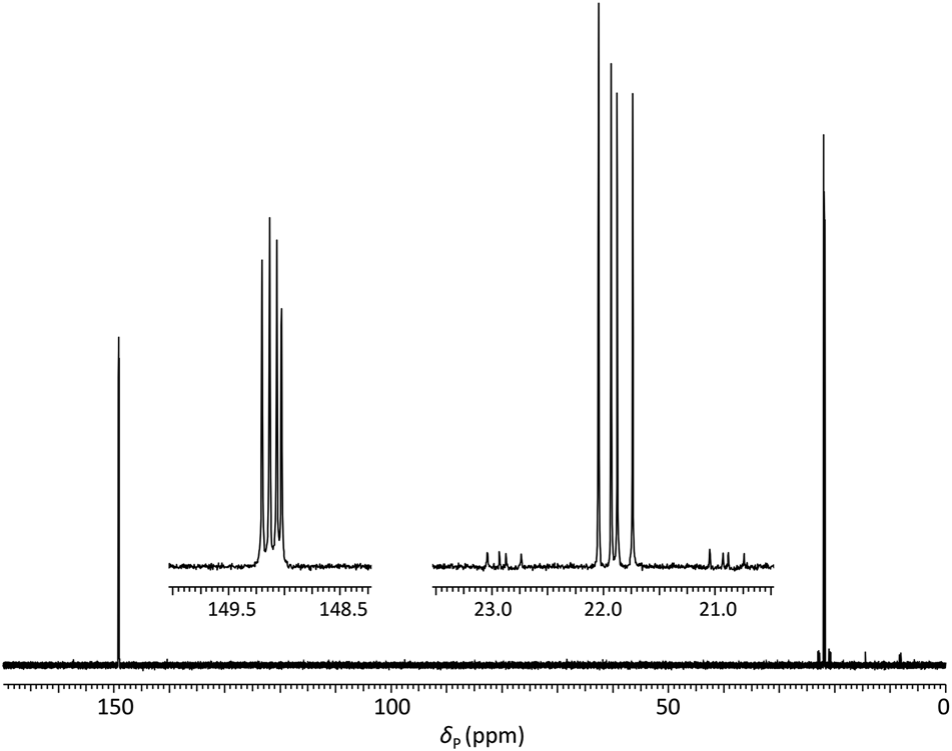
^31^P{^1^H} NMR of phosphoramidite **5a** (prepared from DMTrdA^PAc^*p*SedT – **4a**) in MeCN with (inset) expansions of resonances associated with P(iii) and P(v) nuclei.

Analysis of the dimer phosphoramidites by ^31^P NMR showed expected resonances associated with two separate diastereomeric P(iii) and P(v) phosphorus centres (*e.g.*, Fig. 2). Low levels (<4%) of putative *H*-phosphonate derivatives were also apparent and as this can be associated with autocatalytic degradation,^68^,^69^ the stability of a dimer phosphoramidite in solution was examined by ^31^P NMR. At 4 °C, **5a** (0.1 M in acetonitrile) remained untransformed over 24 days. A separately prepared solution of the same composition was stored on the DNA synthesiser at ambient temperature. After 24 days, solid deposition was observed and ^31^P NMR analysis of the combined materials (solid and liquid) showed over 80% degradation of the phosphoramidite although no transformation of the phosphoroselenolate function was evident.

### Preparation of phosphoroselenolate-modified oligodeoxynucleotides under solid-phase conditions *via* coupling of dimer phosphoramidites

**Fig. 3:**
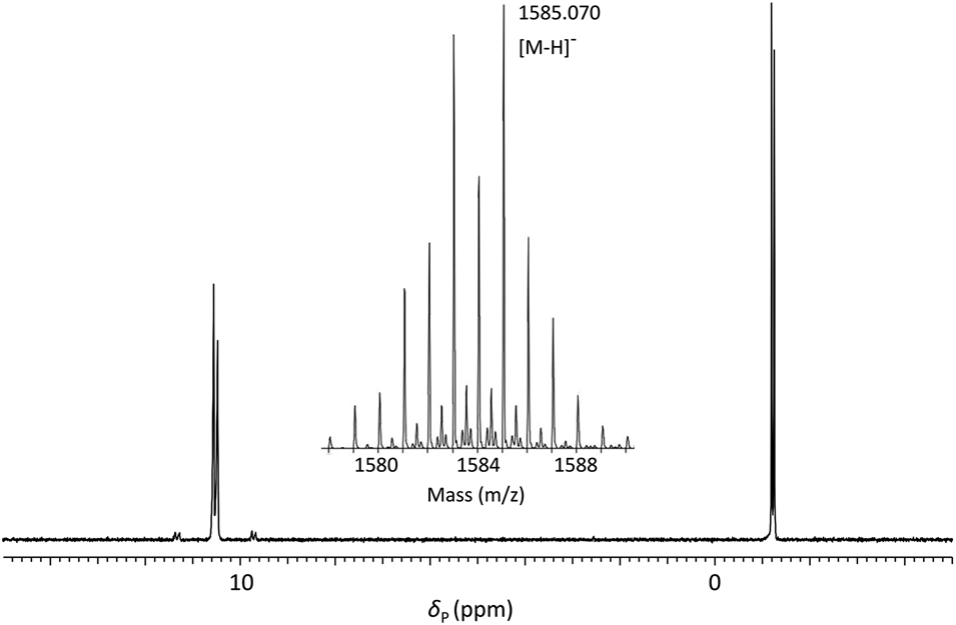
^31^P{^1^H} NMR of **ODN 1** in 60 : 40 (v/v) H_2_O : D_2_O and (inset) ESI-mass spectrum showing isotope pattern associated with molecular ion.

Optimisation of solid-phase synthesis conditions was performed with the dimer phosphoramidite **5d** (derived from DMTrdT*p*SedT) using manual syringe addition of all reagents and acetonitrile washes to prepare a model homothymidine-derived pentamer incorporating two phosphoroselenolate linkages (**ODN 1** – Table 1). Under optimised conditions, quantitative coupling (by trityl release) of **5d** in the presence of the activator 5-*S*-benzylthiotetrazole was observed over five minutes with subsequent oxidation using 0.02 M iodine. Duplicate, one μmol-scale syntheses of the trityl-on, model pentamer were performed. Removal of phosphate diester protecting groups and cleavage from the controlled pore glass (CPG) support required a two-step procedure. Initial demethylation of the phosphoroselenolate triesters was effected following treatment with a solution of 0.2 M sodium diethyldithiocarbamate (NaDEC) in acetonitrile for 30 minutes at room temperature. This method was found to be equally efficient in a side-by-side comparison with standard literature conditions using 1 : 2 : 2 (v/v/v) thiophenol/triethylamine/dioxane.^55^ Excess reagents were removed following washing with acetonitrile, the CPG dried and suspended in 1 : 1 (v/v) 35% (w/v) NH_3_ (aq)/40% (w/v) MeNH_2_ (aq) (AMA) at room temperature for 60 minutes. Following removal of volatile amines, the crude tritylated oligomer **ODN 1** was purified using reversed phase HPLC. Finally, treatment with aqueous acetic acid under standard conditions effected cleavage of the 5′-DMTr function and following desalting, pure **ODN 1** was isolated in 33% yield. Analysis of **ODN 1** by ^31^P NMR (Fig. 3) showed four resonances associated with the two internucleotide phosphate diesters (*δ*_P_ = –1.19, –1.25) and two internucleotide phosphoroselenolate diesters (*δ*_P_ = 10.56, 10.48).

**Table 1:** Phosphoroselenolate-modified oligodeoxynucleotide sequences prepared on solid support using dinucleotides phosphoramidites **5a–d**

Phosphoramidite (coupling time)	Oligodeoxynucleotide sequence 5′→3′	Yield^*a*^ (%)	Mol. ion^*b*^ (calcd)
**5d** (5 min)	**ODN 1** d(TSeTTSeTT)	657 nmol (33%)	1585.070 (1585.112)
**5a** (2 × 7.5 min)	**ODN 2** d(ASeTCCCGGGAT)	541 nmol (27%)	3090.370 (3090.472)
**5b** (2 × 7.5 min)	**ODN 3** d(CSeTCCCGGGAG)	251 nmol (13%)	3091.365 (3091.468)
**5c** (2 × 7.5 min)	**ODN 4** d(GSeTCCCGGGAC)	484 nmol (24%)	3091.351 (3091.468)
**5d** (5 min)	**ODN 5** d(TSeTCCCGGGAA)	337 nmol (17%)	3090.360 (3090.472)
**5a** (2 × 7.5 min)	**ODN 6** d(CGCGAASeTTCGCG)	198 nmol (20%)*	3708.450 (3708.450)

^*a*^Determined using *ε*_260 nm_ of the corresponding native sequence on a 2 μmol scale (* from 1 μmol).

^*b*^ *m*/*z* [M – H]^–^ (^80^Se).

Satellite peaks associated with ^31^P–^77^Se coupling were consistent with a phosphorus–selenium single bond (^1^*J*_PSe_ = 393 Hz and 390 Hz)^70^ although somewhat weaker than that found in the corresponding triester.

**ODN 1** was further characterised using ESI-MS (Fig. 3 – inset) which gave a molecular ion peak ([M – H]^–^) displaying the expected isotope distribution pattern.

In order to further examine the scope of the manual dimer phosphoramidite coupling and deprotection protocol developed for **ODN 1**, a series of self-complementary 10-mer sequences designed to form A-form duplexes (**ODN 2–5**) were prepared. Chain extension of support-bound octanucleotides using phosphoramidite **5d** was performed using the same cycle as previously described. However, less efficient couplings were observed using **5a–c** and therefore an extended double coupling^71^ was employed with these phosphoramidites.

Following oxidation, the columns were washed with acetonitrile and dried under argon. Deprotection using NaDEC followed by AMA liberated the crude, tritylated oligodeoxynucleotides which were purified by RP-HPLC and reduced *in vacuo*. Detritylation of the purified oligomers was accomplished under literature conditions using an extended treatment to fully remove the monomethoxytrityl function from **ODN 4**.

The modified Dickerson–Drew dodecamer^72^ sequence **ODN 6** was prepared under automated conditions using the phosphoroselenolate-linked dimer phosphoramidite **5a** which was incorporated in place of the central AT motif. In addition to the extended coupling of **5a**, the standard cycle was modified to include a wash using 10% (v/v) pyridine/MeCN following the oxidation step. In the absence of this wash, the CPG retained iodine residues which were found to engender lower yields of the modified oligomers. The subsequent five monomer coupling cycles were performed under standard conditions and gave efficient nucleotide incorporation according to trityl yields.

Once complete, the support-bound dodecamer was treated with a solution of 150 mM dithiothreitol (DTT) in 1 : 1 (v/v) H_2_O : absolute ethanol to remove potential oxidised selenium species^37^,^45^ and further demethylated using NaDEC as previously described. Further deprotection with AMA enabled the pure trityl-on oligomer to be isolated following RP-HPLC purification. This material was then detritylated and desalted under standard conditions to afford pure **ODN 6** in 20% yield. Under the same conditions the corresponding native sequence was isolated in 45% yield. All selenium-containing oligodeoxynucleotides were characterised by mass spectrometry in which the *m*/*z* ratios exhibited the expected isotope distribution patterns and did not show any peaks associated with selenoxide formation.

### Preparation of a model phosphoroselenolate-modified oligodeoxynucleotide under solid-phase conditions *via* Michaelis–Arbuzov chemistry

**Scheme 2:**
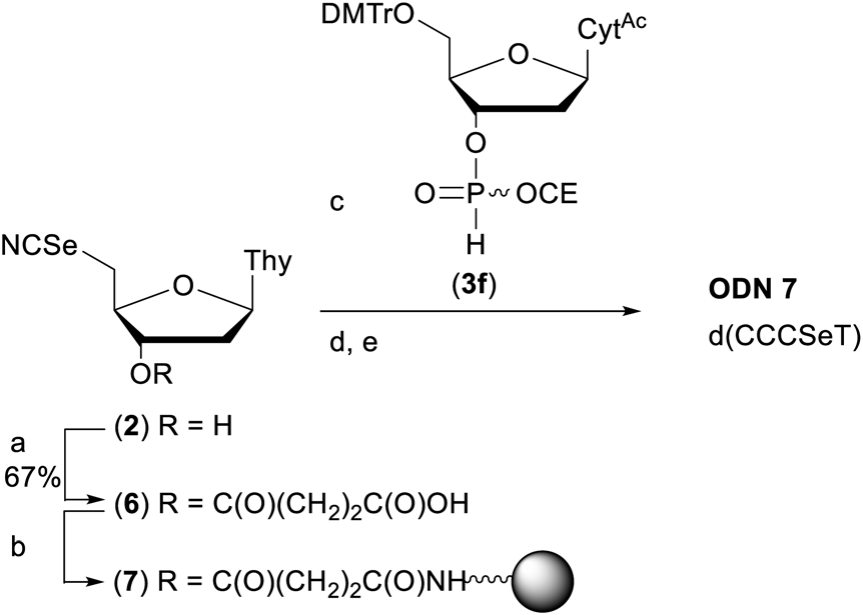
Preparation of **ODN 7**. Reagents and conditions: (a) succinic anhydride (1 eq.), DMAP (0.8 eq.), pyridine, rt, overnight; (b) (i) *p*-nitrophenol (1 eq.), DCC (2.5 eq.), DCM, rt, 2 h; (ii) amino SynBase 500/110 CPG (500 mg), DMF, Et_3_N (4 eq.), rt, 5 h. (c) **3f**, MeCN, rt, 1 h; (d) solid-phase DNA synthesis using manual syringe addition. (e) (i) AMA, rt, 2 h. (ii) 80% AcOH, rt, 1 h. See ESI† for full details.

In order to demonstrate the general utility of the M–A reaction for constructing internucleotide phosphoroselenolates under solid-phase conditions, this chemistry was used to prepare a tetranucleotide sequence which, in its native form, crystallises in the four-stranded, i-motif conformation.^73^,^74^ Thus, **ODN 7** was prepared from the corresponding support-bound nucleoside selenocyanate (Scheme 2). Succinylation of selenonucleoside **2** under standard conditions^75^ gave complete consumption of starting material. Following extraction with cold citric acid, the succinyl ester (**6**) was isolated contaminated with the corresponding symmetrical diselenide (30 mol%). This crude material was treated with excess condensing agent (DCC) in the presence of 4-nitrophenol^76^ and following removal of precipitated DCU, the mixture of activated esters reacted with amino-functionalised controlled pore glass. The CPG-bound selenonucleoside (**7**) thus obtained was reacted with the nucleoside *H*-phosphonate **3f**. After 60 minutes, trityl assay indicated that the support loading (39 μmol g^–1^) was consistent with that typically used in solid-phase DNA synthesis indicating that efficient M–A reaction had occurred.

Using this material (1.75 μmol), chain extension was performed under manual conditions using standard monomer phosphoramidites using the cycle developed for **ODN 1**. Subsequent deprotection with AMA liberated the crude tritylated tetramer which was purified by RP-HPLC, detritylated and desalted to afford pure **ODN 7**.

### Enzymatic digestion

**Fig. 4:**
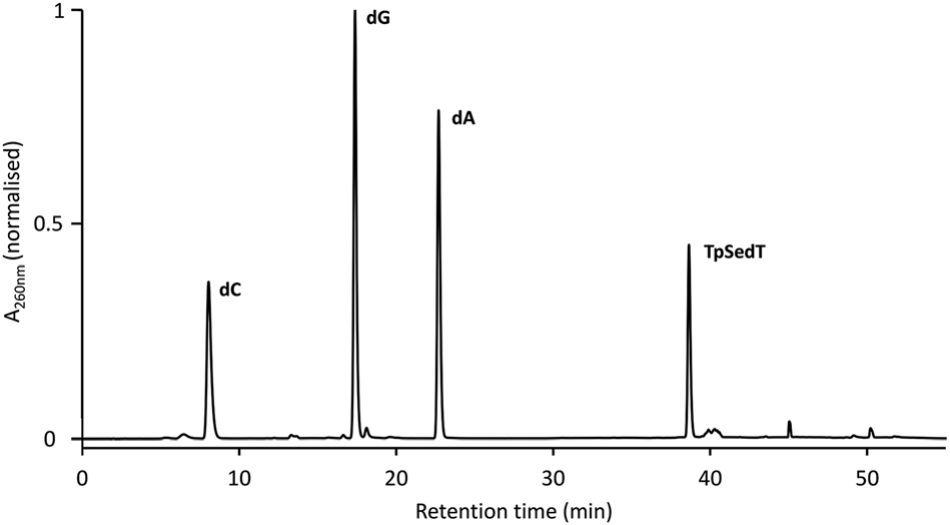
RP-HPLC chromatogram of the snake venom digest of **ODN 5** (after 8 hours at 37 °C). For full conditions see ESI.†

Further confirmation of the integrity of the internucleotide phosphoroselenolate linkage during both coupling and downstream processing was provided by enzymatic digestion of both **ODN 5** and the corresponding native sequence. Using commercially-sourced snake venom with both phosphodiesterase and phosphatase activities, complete digestion of the native 10-mer to the corresponding nucleosides was found within eight hours at 37 °C (Fig. S2†). Under the same conditions, **ODN 5** liberated the nucleosides dA, dC and dG in the expected ratios but less than 1 mol% of T (*t*_R_ = 18.1 min) was observed (Fig. 4). However, a single major peak with significantly longer retention time (*t*_R_ = 38.7 min) was identified as the dimer TpSedT following coinjection with an authentic sample.^49^ Previously, Stec and coworkers examined nuclease digestion of putative TpSedT and described using ten times the quantity of enzyme required to effect cleavage of the corresponding phosphorothioate substrate. During this treatment, formation of the corresponding diselenide; (SedT)_2_, was observed and this compound is assumed to be the origin of a novel hydrophobic material (*t*_R_ = 45.1 min) which was also apparent during extended treatment of pure TpSedT with snake venom. Early studies on the digestion of dimers incorporating a 5′-thiothymidylate (*e.g.*, TpSdT) also reported slow cleavage activities^77^,^78^ although Xu and Kool subsequently described snake venom digestion of a 20-mer containing a single internal modification which was shown to proceed at essentially the same rate as the unmodified sequence and cleaved the internal phosphorothiolate linkage.^79^

Although the scissile bond (3′O–P) is not modified in the phosphoroselenolate analogues examined in this study, it is assumed that distortion in the phosphoryl moiety arising out of the extended bond lengths and compressed bond angle associated with the P–Se–C5′ bridge (*vide infra*) inhibits productive binding of the dimer.

### UV thermal denaturation studies

The effect of the phosphoroselenolate linkage upon duplex stability was investigated by comparing thermal denaturation of the modified oligomers with the corresponding native sequences (Table 2). Sequence-dependent destabilisation of duplexes designed to exhibit A-form conformations was observed following phosphoroselenolate substitution of the 5′-terminal phosphodiester linkage (entries 1–4). The largest reductions in melting temperatures were observed for sequences in which the native oligomers had the greatest stabilities by virtue of the presence of a terminal C–G (entry 2) or G–C (entry 3) base pair and in these circumstances, Δ*T*_m_ values of –6.2 °C and –4.9 °C (respectively) were observed. In contrast, introduction of a phosphoroselenolate-linkage downstream from a terminal A–T base pair (entry 1) had a minor effect upon duplex stability (Δ*T*_m_ = –0.7 °C) although the effect upon the T–A (entry 4) pairing was more significant (Δ*T*_m_ = –4.0 °C).

**Table 2:** Melting temperatures (*T*_m_) of self-complementary phosphoroselenolate-modified and native oligodeoxynucleotide duplexes

Entry	Sequence 5′→3′	X =	*T*_m_/°C	X =	*T*_m_/°C	Δ*T*_m_^*a*^/°C
1	d(A**X**CCCGGGAT)	SedT (**ODN 2**)	46.9	T	47.6	–0.7
2	d(C**X**CCCGGGAG)	SedT (**ODN 3**)	48.1	T	54.3	–6.2
3	d(G**X**CCCGGGAC)	SedT (**ODN 4**)	50.7	T	55.6	–4.9
4	d(T**X**CCCGGGAA)	SedT (**ODN 5**)	43.0	T	47.0	–4.0
5	d(CGCGAA**X**TCGCG)	SedT (**ODN 6**)	55.9	T	57.8	–1.9

^*a*^Δ*T*_m_ = *T*_m_(X = SedT) – *T*_m_(X = T). Conditions: ssDNA (10 μM), NaP_i_ (10 mM, pH 7.0), NaCl (100 mM).

The effect of introducing juxtaposed phosphoroselenolate bridges into the more extended backbone typical of B-form helices was tested using the Dickerson–Drew dodecamer sequence **ODN 6** (entry 5). A decrease in stability of 1.9 °C was observed in comparison with the native oligomer. Xu and Kool reported the effect upon duplex stability of substituting a single internal phosphate diester-linkage within a 17-mer (B-form) sequence with either a phosphorothiolate or phosphoroselenolate analogue.^48^ Duplexes formed between these chalcogen-modified oligomers and the native compliment displayed Δ*T*_m_ values of –3.0 °C (Se) and –2.0 °C (S). These values are consistent with those obtained in the current study in which two modifications are introduced per duplex.

### Solution phase conformation studies

**Fig. 5:**
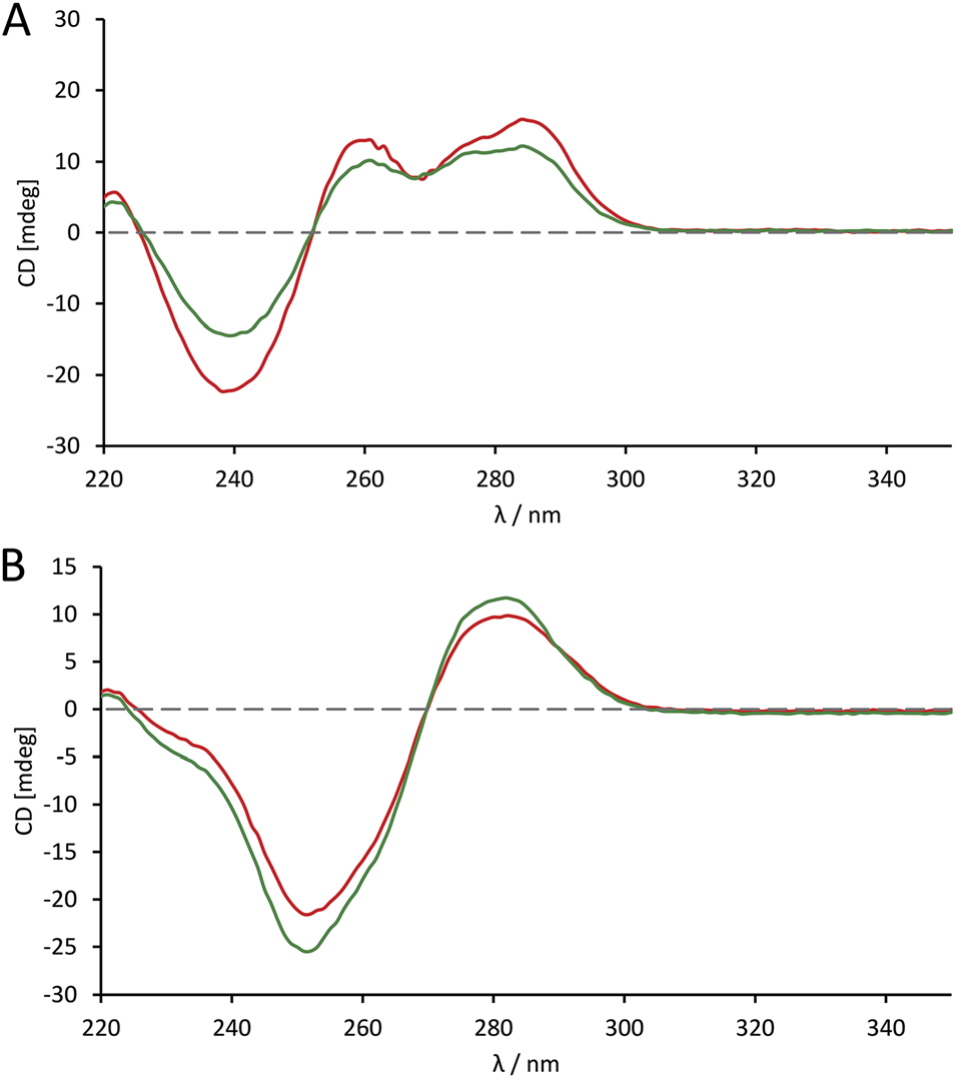
CD spectra comparing the conformation of phosphoroselenolate-modified sequences (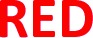) and the corresponding native (all phosphodiester) sequences (
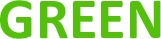
). (A) **ODN 4**; (B) **ODN 6**.

The effects of introducing the phosphoroselenolate modification upon the conformations of self-complementary duplex DNA sequences were investigated using circular dichroism. The spectral shape and peak positions of native and modified duplexes displayed a high level of congruence, with only small changes in band intensity observed (Fig. S5–S9†). For example, conservation of the mixed A/B conformation adopted in solution by **ODN 4** (Fig. 5A) highlights that the modification is not perturbing or directing the conformation of the duplex in a boarderline case.

The advantage of the phosphoroselenolate modification over ribose modifications, such as replacement of O with Se in the ring, which typically leads to formation of an A-form duplex,^33^ is clear. It should also be noted that both the native and modified dodecamer (Fig. 5B) exhibited the classical B-form again illustrating that the modification has had little effect on the solution conformation of the oligonucleotide. In all cases the incorporation of the Se into the DNA backbone means that the environment in the major and minor grooves is unaffected, unlike with modifications such as 5-bromo-dU or most selenium modified DNA. Whilst some of these modifications (such as a 2′-SeMe analogues)^42^ may not significantly affect the native conformation if this is A-form DNA, the addition of groups into possible binding sites could affect the validity of drug–DNA binding studies, which is not an issue with a phosphoroselenolate modification.

### Crystal structure

To confirm that the structure of the nucleic acid is not significantly perturbed by the introduction of a phosphoroselenolate modification, crystallisation trials of oligodeoxynucleotide sequences containing the group were performed. This resulted in the crystal structure of d(GSeTCCCGGGAC) – **ODN 4** – being obtained, which allows for visualisation of the modification at 1.45 Å resolution.

**Fig. 6:**
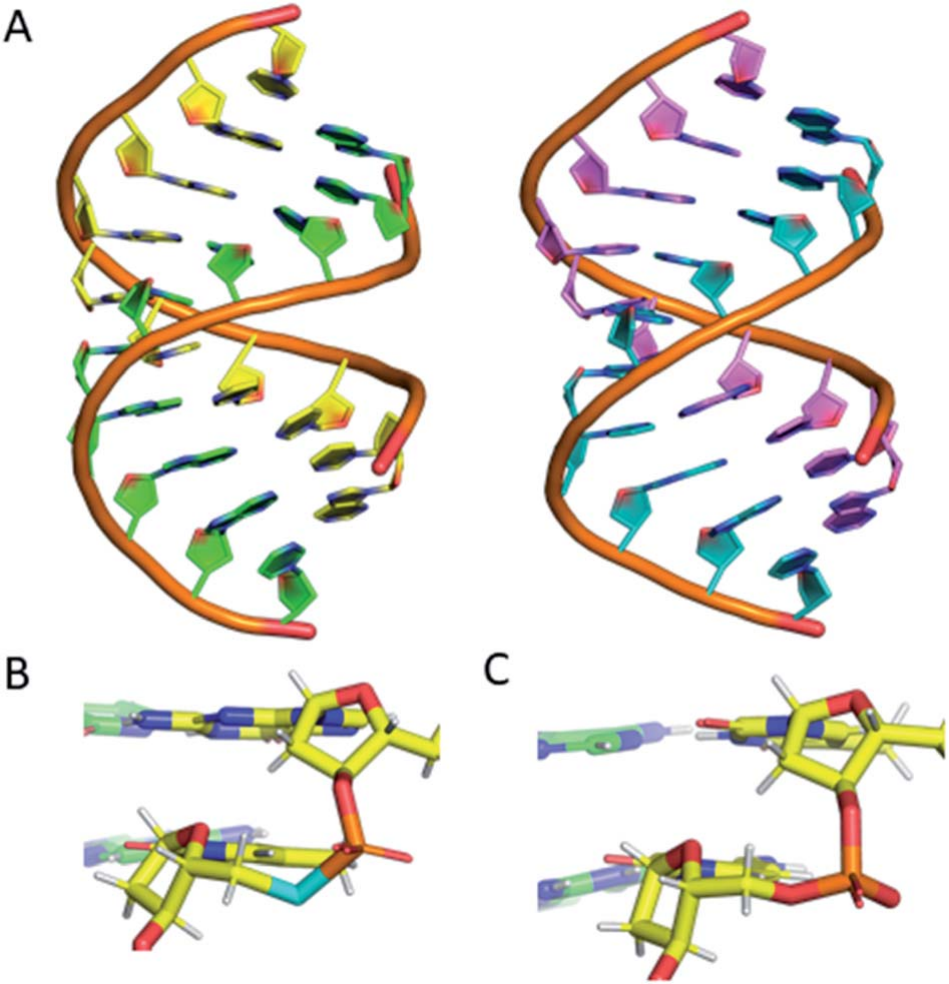
(A) (left) The DNA structure reported here (**ODN 4**) and (right) a standard A-form DNA 10-mer.^81^ (B) The phosphoroselenoate modification (cyan) compared with (C) the naturally occurring backbone at the terminal AC step. The chains are coloured according to atom type, with carbon in red, green, cyan or magenta (as a function of DNA chain), nitrogen in blue, oxygen in red, selenium in cyan, phosphorus in orange and hydrogen in white. PDB ID: ; 6S7D.†

The structure shows that the overall conformation of the duplex is that of A-DNA (Fig. 6A). The average twist of the helix is 31.8° which compares favourably to a standard value of 32.7°. However, at the modification site the T*–A base pair has a twist of *ca.* 41°, which can be attributed to the increased bond lengths from P–Se (2.25 Å) and Se–C5′ (1.94 Å) compared to the oxygen-containing equivalent (P–O5′ 1.60 Å, O5′–C5′ 1.42 Å).

Apart from this slight increase in bond lengths, and subsequent increase in helical twist angle, the base pair containing the phosphoroselenolate modification is otherwise consistent with a standard A-DNA base pair (Fig. 6B and C), with parameters such as roll, tilt, rise and slide all within the normal range for this conformation (analysis performed using W3DNA).^80^ Whilst it could be expected that the increased twist or bond lengths could affect the sugar pucker of the nucleotide, this is not the case, with the nucleotide possessing a C3′-*endo* sugar pucker, consistent with that of A-DNA.

The nucleotide 3′ to the modified base also possesses a C3′-*endo* pucker. The base 5′ to the modification site possesses a non-standard pucker, C2′-*exo*, which is attributed to the proximity of a neighbouring strand, forming a crystal contact within the lattice. This structure therefore confirms that the introduction of the modification does not significantly perturb the solid-state conformation of the duplex.

## Conclusions

For the first time, a phosphoroselenolate-modified oligomer has been prepared *de novo* using phosphoramidite chemistry with only minor modification to the standard solid-phase DNA synthesis cycle. All modified oligonucleotides were characterised by electrospray and/or MALDI mass spectrometry which gave molecular ion masses (with appropriate isotope distribution patterns) consistent with the single-stranded sequence and if self-complimentary, the corresponding duplex. No M+16 peaks were apparent in these spectra suggesting that either the oxidation conditions did not lead to oxygen transfer to selenium or that downstream processing of such modified oligomers leads to their removal. This was further confirmed by ^31^P NMR analysis of a double-labelled model pentamer.

Unlike previous generations of DNA analogues in which selenium is introduced within the sugar moiety, the phosphoroselenolate modification described here is readily accessible requiring only four high yielding steps and does not require handling highly air-sensitive materials. Furthermore, the furanose pseudorotation angles of the most common modification, 2′-SeMe, typically results in conformational steering towards an A-form duplex. Using the related 2′-SMe analogue, such conformational restrictions were not observed and both A- and B-DNA duplex crystal structures were solved^82^,^83^ although longer collection times (and concomitant DNA damage) are associated with the weaker anomalous scattering from sulfur at the CuK_α_ wavelength. Furthermore, both analogues interfere with minor groove binding interactions. Finally, the expeditious crystallisation processes observed using other selenium-modified nucleic acids was maintained with the phosphoroselenolate.

Selenium-SAD has recently been applied to the *de novo* phasing of data generated from an X-ray-free electron laser including serial femtosecond crystallography^84^,^85^ and therefore offers the potential to observe conformational changes within nucleic acids during folding, recognition and processing.

The resistance of the internucleoside phosphoroselenolate linkage towards the exonuclease activity of snake venom offers the potential to both facilitate *in vivo* activity of therapeutic nucleic acid sequences^86^ and to probe biochemical processes in the same fashion as have phosphorothiolate^56^,^87^ and we envisage that the ability to label an oligonucleotide backbone using 100% ^77^Se may provide a valuable tool for investigating the biological processing of such materials.^88^ Towards these future goals we are currently investigating the application of this chemistry for the preparation of phosphoroselenolate-linkages derived from other selenonucleoside analogues.

## Conflicts of interest

There are no conflicts to declare.

## Supplementary Material

Supplementary information

Supplementary information

Supplementary information

## References

[cit1] Caruthers M. H. (1985). Science.

[cit2] Kosuri S., Church G. M. (2014). Nat. Methods.

[cit3] Saiki R. K., Scharf S., Faloona F., Mullis K. B., Horn G. T., Erlich H. A., Arnheim N. (1985). Science.

[cit4] Heid C. A., Stevens J., Livak K. J., Williams P. M. (1996). Genome Res..

[cit5] Smith M. (1985). Annu. Rev. Genet..

[cit6] Jinek M., Chylinski K., Fonfara I., Hauer M., Doudna J. A., Charpentier E. (2012). Science.

[cit7] Shen H., Wang Y., Wang J., Li Z., Yuan Q. (2019). ACS Appl. Mater. Interfaces.

[cit8] Seeman N. C., Sleiman H. F. (2017). Nat. Rev. Mater..

[cit9] Ceze L., Nivala J., Strauss K. (2019). Nat. Rev. Genet..

[cit10] Rothemund P. W. K. (2006). Nature.

[cit11] Andersen E. S., Dong M., Nielsen M. M., Jahn K., Subramani R., Mamdouh W., Golas M. M., Sander B., Stark H., Oliveira C. L. P., Pedersen J. S., Birkedal V., Besenbacher F., Gothelf K. V., Kjems J. (2009). Nature.

[cit12] Sheng J., Gan J., Huang Z. (2013). Med. Res. Rev..

[cit13] Satange R., Chang C. K., Hou M. H. (2018). Nucleic Acids Res..

[cit14] Hendrickson W. A. (2014). Q. Rev. Biophys..

[cit15] Wimberly B. T., Brodersen D. E., Clemons W. M., Morgan-Warren R. J., Carter A. P., Vonrheln C., Hartsch T., Ramakrishnan V. (2000). Nature.

[cit16] Hendrickson W. A., Horton J. R., LeMaster D. M. (1990). EMBO J..

[cit17] Hendrickson W. A., Pähler A., Smith J. L., Satow Y., Merritt E. A., Phizackerley R. P. (1989). Proc. Natl. Acad. Sci. U. S. A..

[cit18] Su X. D., Zhang H., Terwilliger T. C., Liljas A., Xiao J., Dong Y. (2015). Crystallogr. Rev..

[cit19] Mori K., Boiziau C., Cazenave C., Matsukura M., Subasinghe C., Cohen J. S., Broder S., Toulmé J. J., Stein C. A. (1989). Nucleic Acids Res..

[cit20] Wilds C. J., Pattanayek R., Pan C., Wawrzak Z., Egli M. (2002). J. Am. Chem. Soc..

[cit21] Tram K., Wang X., Yan H. (2007). Org. Lett..

[cit22] Guga P., Maciaszek A., Stec W. J. (2005). Org. Lett..

[cit23] Egli M., Lubini P., Pallan P. S. (2007). Chem. Soc. Rev..

[cit24] Salon J., Sheng J., Jiang J., Chen G., Caton-Williams J., Huang Z. (2007). J. Am. Chem. Soc..

[cit25] Salon J., Jiang J., Sheng J., Gerlits O. O., Huang Z. (2008). Nucleic Acids Res..

[cit26] Hassan A. E. A., Sheng J., Zhang W., Huang Z. (2010). J. Am. Chem. Soc..

[cit27] Habuchi T., Yamaguchi T., Aoyama H., Horiba M., Ito K. R., Obika S. (2019). J. Org. Chem..

[cit28] Liczner C., Grenier V., Wilds C. J. (2018). Tetrahedron Lett..

[cit29] Du Q., Carrasco N., Teplova M., Wilds C. J., Egli M., Huang Z. (2002). J. Am. Chem. Soc..

[cit30] Höbartner C., Micura R. (2004). J. Am. Chem. Soc..

[cit31] Zhang W., Szostak J. W., Huang Z. (2016). Front. Chem. Sci. Eng..

[cit32] Morihiro K., Kodama T., Kentefu, Moai Y., Veedu R. N., Obika S. (2013). Angew. Chem., Int. Ed..

[cit33] Watts J. K., Johnston B. D., Jayakanthan K., Wahba A. S., Pinto B. M., Damha M. J. (2008). J. Am. Chem. Soc..

[cit34] Inagaki Y., Minakawa N., Matsuda A. (2008). Nucleic Acids Symp. Ser..

[cit35] Carrasco N., Ginsburg D., Du Q., Huang Z. (2001). Nucleosides, Nucleotides Nucleic Acids.

[cit36] Nuthanakanti A., Boerneke M. A., Hermann T., Srivatsan S. G. (2017). Angew. Chem., Int. Ed..

[cit37] Höbartner C., Rieder R., Kreutz C., Puffer B., Lang K., Polonskaia A., Serganov A., Micura R. (2005). J. Am. Chem. Soc..

[cit38] Tarashima N., Hayashi K., Terasaki M., Taniike H., Inagaki Y., Hirose K., Furukawa K., Matsuda A., Minakawa N. (2014). Org. Lett..

[cit39] Taniike H., Inagaki Y., Matsuda A., Minakawa N. (2011). Tetrahedron.

[cit40] Ishii K., Saito-Tarashima N., Ota M., Yamamoto S., Okamoto Y., Tanaka Y., Minakawa N. (2016). Tetrahedron.

[cit41] Micura R., Höbartner C., Rieder R., Kreutz C., Puffer B., Lang K., Moroder H. (2006). Curr. Protoc. Nucleic Acid Chem..

[cit42] Salon J., Sheng J., Gan J., Huang Z. (2010). J. Org. Chem..

[cit43] Sheng J., Jiang J., Salon J., Huang Z. (2007). Org. Lett..

[cit44] Sheng J., Salon J., Gan J., Huang Z. (2010). Sci. China: Chem..

[cit45] Moroder H., Kreutz C., Lang K., Serganov A., Micura R. (2006). J. Am. Chem. Soc..

[cit46] Thompson R. A., Spring A. M., Sheng J., Huang Z., Germann M. W. (2015). J. Biomol. Struct. Dyn..

[cit47] Wozniak L. A., Sochacki M., Mitsuya H., Kageyama S., Stec W. J. (1994). Bioorg. Med. Chem. Lett..

[cit48] Xu Y., Kool E. T. (2000). J. Am. Chem. Soc..

[cit49] Eguaogie O., Conlon P. F., Vyle J. S. (2016). Tetrahedron Lett..

[cit50] Pistschimuka P. (1911). J. Prakt. Chem..

[cit51] Mielniczak G., Lopusiński A. (2003). Heteroat. Chem..

[cit52] Michelini M. del C., Russo N., Alcaro S., Wozniak L. A. (2012). Tetrahedron.

[cit53] Wozniak L. A., Gra M., Kaminski Z., Stec W. J. (2008). Phosphorus, Sulfur Silicon Relat. Elem..

[cit54] Kullberg M., Stawinski J. (2005). Nucleosides, Nucleotides Nucleic Acids.

[cit55] Cosstick R., Eckstein F. (1985). Biochemistry.

[cit56] Grandas A., Marshall W. S., Nielsen J., Caruthers M. H. (1989). Tetrahedron Lett..

[cit57] Cosstick R., Vyle J. S. (1990). Nucleic Acids Res..

[cit58] Misiura K., Stec W. J. (1994). Bioorg. Med. Chem. Lett..

[cit59] Belostotskii A. M., Lexner J., Hassner A. (1999). Tetrahedron Lett..

[cit60] Eguaogie O., Conlon P. F., Ravalico F., Sweet J. S. T., Elder T. B., Conway L. P., Lennon M. E., Hodgson D. R. W., Vyle J. S. (2017). Beilstein J. Org. Chem..

[cit61] Eguaogie O., Vyle J. S., Conlon P. F., Gîlea M. A., Liang Y. (2018). Beilstein J. Org. Chem..

[cit62] Ju Y., Kumar D., Varma R. S. (2006). J. Org. Chem..

[cit63] Suchsland R., Appel B., Janczyk M., Müller S. (2019). Appl. Sci..

[cit64] Hassler M., Wu Y. Q., Mallikarjuna Reddy N., Chan T. H., Damha M. J. (2011). Tetrahedron Lett..

[cit65] Suchsland R., Appel B., Müller S. (2018). Beilstein J. Org. Chem..

[cit66] Vyle J. S., Williams N. H., Grasby J. A. (1998). Tetrahedron Lett..

[cit67] Thorpe C., Epple S., Woods B., El-Sagheer A. H., Brown T. (2019). Org. Biomol. Chem..

[cit68] Hargreaves J. S., Kaiser R., Wolber P. K. (2015). Nucleosides, Nucleotides Nucleic Acids.

[cit69] Krotz A. H., Rentel C., Gorman D., Olsen P., Gaus H. J., McArdle J. V., Scozzari A. N. (2004). Nucleosides, Nucleotides Nucleic Acids.

[cit70] Glidewell C., Leslie E. J. (1977). J. Chem. Soc., Dalton Trans..

[cit71] Yu L. J., Wiederholt C. J., Patro J. N., Haraguchi K., Greenberg M. M. (2005). J. Org. Chem..

[cit72] Drew H. R., Wing R. M., Takano T., Broka C., Tanaka S., Itakura K., Dickerson R. E. (1981). Proc. Natl. Acad. Sci. U. S. A..

[cit73] Kang C. H., Berger I., Lockshin C., Ratliff R., Moyzis R., Rich A. (1994). Proc. Natl. Acad. Sci. U. S. A..

[cit74] Assi H. A., Garavís M., González C., Damha M. J. (2018). Nucleic Acids Res..

[cit75] Adams S. P., Kavka K. S., Wykes E. J., Holder S. B., Galluppi G. R. (1983). J. Am. Chem. Soc..

[cit76] Zuckermann R., Corey D., Schultz P. (1987). Nucleic Acids Res..

[cit77] Cook A. F. (1970). J. Am. Chem. Soc..

[cit78] Chladek S., Nagyvary J. (1972). J. Am. Chem. Soc..

[cit79] Xu Y., Kool E. T. (1998). Nucleic Acids Res..

[cit80] Li S., Olson W. K., Lu X.-J. (2019). Nucleic Acids Res..

[cit81] Frederick C. A., Quigley G. J., Teng M. K., Coll M., van der Marel G. A., van Boom J. H., Rich A., Wang A. H.-J. (1989). Eur. J. Biochem..

[cit82] Pallan P. S., Prakash T. P., Li F., Eoff R. L., Manoharan M., Egli M. (2009). Chem. Commun..

[cit83] Harp J., Pallan P., Egli M. (2016). Crystals.

[cit84] Martin-Garcia J. M., Conrad C. E., Coe J., Roy-Chowdhury S., Fromme P. (2016). Arch. Biochem. Biophys..

[cit85] Yoon C. H., DeMirci H., Sierra R. G., Dao E. H., Ahmadi R., Aksit F., Aquila A. L., Batyuk A., Ciftci H., Guillet S., Hayes M. J., Hayes B., Lane T. J., Liang M., Lundström U., Koglin J. E., Mgbam P., Rao Y., Rendahl T., Rodriguez E., Zhang L., Wakatsuki S., Boutet S., Holton J. M., Hunter M. S. (2017). Sci. Data.

[cit86] Setten R. L., Rossi J. J., ping Han S. (2019). Nat. Rev. Drug Discovery.

[cit87] Cosstick R., Gaynor J. (2008). Curr. Org. Chem..

[cit88] Veres Z., Tsai L., Scholz T. D., Politino M., Balaban R. S., Stadtman T. C. (1992). Proc. Natl. Acad. Sci. U. S. A..

