# Supplementary material for: Solid-phase synthesis and structural characterisation of phosphoroselenolate-modified DNA: a backbone analogue which does not impose conformational bias and facilitates SAD X-ray crystallography
Source: Chem Sci. 2019 Oct 11;10(47):10948–57. doi: 10.1039/c9sc04098f (PMC7066676; doi:10.1039/c9sc04098f)
Supplement: Supplementary file 1 [file SC-010-C9SC04098F-s001.pdf]

Electronic Supplementary Material (ESI) for Chemical Science.
This journal is © The Royal Society of Chemistry 2019

Electronic Supplementary Information (ESI)

Solid-phase synthesis and structural characterisation of phosphoroselenolate-modified DNA: a backbone analogue which does not impose conformational bias and facilitates SAD X-ray crystallography

Patrick F. Conlon,[a] Olga Eguaogie,[a] Jordan J. Wilson,[a] Jamie S. T. Sweet,[a] Julian Steinhoegl,[b] Klaudia Englert,[c] Oliver G. A. Hancox,[b] Christopher J. Law,[d] Sarah A. Allman,[b] James H. R. Tucker,[c] James P. Hall[b,e]* and Joseph S. Vyle[a]*

[a] School of Chemistry and Chemical Engineering, Queen's University of Belfast, David Keir Building, Stranmillis Road, Belfast BT9 5AG, UK. *E-mail: j.vyle@qub.ac.uk; Tel: +44 (0)28 9097 5485.
[b] Reading School of Pharmacy, University of Reading, Whiteknights, Reading RG6 6AP, UK.
[c] School of Chemistry, University of Birmingham, Edgbaston, Birmingham B15 2TT, UK.
[d] School of Biological Sciences, Queen's University Belfast, 15 Chlorine Gardens, Belfast BT9 5AH, UK.
[e] Diamond Light Source, Chilton, Didcot, Oxfordshire, UK. *E-mail: james.hall@reading.ac.uk; Tel: +44 (0) 118 378 4638

Table of Contents

Section A. General Information 2

Section B. Experimental Procedure and Material Characterisation 4
5′-deoxythymidine-5′-selenocyanate (**2**). 4
DMTrdA^{PAc}*p*SedT (**4a**). 5
DMTrdC^{Ac}*p*SedT (**4b**). 6
MMTrdG^{iBu}*p*SedT (**4c**). 7
DMTrT*p*SedT (**4d**). 8
DMTrdG^{iBu}*p*SedT (**4e**). 9
DMTrdA^{PAc}*p*SedT-Phosphoramidite (**5a**). 10
DMTrdC^{Ac}*p*SedT-Phosphoramidite (**5b**). 10
MMTrdG^{iBu}*p*SedT-Phosphoramidite (**5c**). 11
DMTrT*p*SedT-Phosphoramidite (**5d**). 11
5′-deoxythymidine-5′-selenocyanate-3′-*O*-succinate (**6**). 12
5′-deoxythymidine-5′-selenocyanate-functionalised CPG (**7**). 12

Oligodeoxynucleotide synthesis: 13
d(TSeTTSeTT) (**ODN 1**) 13
d(ASeTCCCGGGAT) (**ODN 2**). 14
d(CSeTCCCGGGAG) (**ODN 3**). 14
d(GSeTCCCGGGAC) (**ODN 4**). 14
d(TSeTCCCGGGAA) (**ODN 5**). 14
d(CGCGAASeTTCGCG) (**ODN 6**). 15
d(CCCSeT) (**ODN 7**). 16

Oligodeoxynucleotide Characterisation: 17
MALDI-TOF 17
ESI 17
Enzyme Digest 17
UV Thermal Denaturation Studies 19
CD Spectroscopy Studies 19
Crystallisation, X-ray data processing and refinement 21

References 23

Section C. Supplementary Figures 24
DMTrdA^{PAc}*p*SedT (**4a**). 24
DMTrdC^{Ac}*p*SedT (**4b**). 28
MMTrdG^{iBu}*p*SedT (**4c**). 32
DMTrT*p*SedT (**4d**). 36
DMTrdG^{iBu}*p*SedT (**4e**). 40
DMTrdA^{PAc}*p*SedT-Phosphoramidite (**5a**). 44
DMTrdC^{Ac}*p*SedT-Phosphoramidite (**5b**). 47
MMTrdG^{iBu}*p*SedT-Phosphoramidite (**5c**). 50
DMTrT*p*SedT-Phosphoramidite (**5d**). 53
5′-deoxythymidine-5′-selenocyanate-3′-O-succinate (**6**) 56
d(TSeTTSeTT) (**ODN 1**). 59
d(ASeTCCCGGGAT) (**ODN 2**) 61
d(CSeTCCCGGGAG) (**ODN 3**) 63
d(GSeTCCCGGGAC) (**ODN 4**) 65
d(TSeTCCCGGGAA) (**ODN 5**) 67
d(CGCGAASeTTCGCG) (**ODN 6**) 69
d(CCCSeT) (**ODN 7**). 71
Structural analysis for **ODN 4** 73

Section A. General Information

CAUTION! Strict safety guidelines are required when handling selenium-containing compounds due to the potential for generating highly toxic materials.[1,2] Potassium selenocyanate and its adducts must always be handled in a fume hood and wearing the appropriate PPE to avoid exposure to airways, eyes and skin. Contaminated equipment must be treated with aqueous sodium hypochlorite (bleach) to inactivate the selenium species to a less harmful oxidised form.[3]

Unless further purified (see below), reagents and solvents were of HPLC or analytical grade and used as supplied (Merck / Sigma-Aldrich / Chemgenes or LGC / Link Technologies). Anhydrous acetonitrile used in reactions was diluent grade (LGC / Link Technologies) which was stored under argon over 1 g or 5 g molecular traps (LGC Biosearch). Methyl-protected phosphoramidite derivatives used in the preparation of the corresponding *H*-phosphonates were purchased from LGC / Link Technologies (dA, dC and T) or ChemGenes (dG). Analytical grade dichloromethane used in reactions was dried and distilled from calcium hydride, stored over 3 Å activated molecular sieves for a minimum of 24 h in the absence of light, purged with argon for 30 min and used within 7 days. When used to co-evaporate trityl protected material the anhydrous dichloromethane was deacidified by passing over a plug of activated basic alumina prior to use. HPLC grade methanol >99% (Fischer) was rendered anhydrous following storage over 3 Å activated molecular sieves 48-72 h prior to use. *N*,*N*-diisopropylethylamine 99% (TCI) was dried over 3 Å activated molecular sieves for at least 48 h prior to use. Column chromatography was performed using silica (Fluorochem 60 Å, 40 – 63 µm) which had been dried at 150 °C. Mobile phases were prepared using HPLC grade acetone, analytical grade dichloromethane and rendered anhydrous following storage over 3 Å activated molecular sieves (minimum 24 h) and purged with argon for 30 min prior to their use to purify phosphoramidites **5a-d**. Pyridine (Acros 99+% extra pure) was refluxed and distilled from potassium hydroxide immediately prior to use.

TLC analysis was performed using (aluminium backed) Merck Kieselgel 60 F_{254} plates and materials visualised using one or more of the following: UV illumination (254 nm); treatment with 0.1% (w/v) Ellman's reagent (5, 5′-dithiobis(2-nitrobenzoic acid)) in 1:1 (v/v) EtOH : 0.45 M Tris·HCl (pH 8.5) (for P(III)-containing materials); exposure to gaseous HCl (for tritylated materials); and 3% (w/v) phenol in 95:5 (v/v) ethanol : conc. H_2SO_4 followed by heating (for tritylated and sugar-positive materials). Where appropriate, the plates were subsequently heated at high temperature (ca. 100 – 200 °C).

^{1}H NMR spectra were recorded on a Bruker Ascend 600 MHz at 300K. ^{31}P NMR spectra were recorded on a Bruker III-400 MHz or a Bruker Ascend 600 MHz at 300 K with an internal D_2O lock. ^{77}Se NMR spectra were recorded on a Bruker Ascend 600 MHz at 300 K with an internal standard of KSeCN (0.25 M in D_2O, δ_{Se} = -329.0).[4]

Mass Spectroscopy

For nucleosides, dinucleotides and dinucleotide phosphoramidites, mass spectrum were recorded using a VG Quattro II Triple Quadrupole Mass Spectrometer (Electrospray) or using a Waters Xevo G2-XS QTof Mass Spectrometer (Electrospray). Mass spectrometry was performed by Analytical Services and Environmental Projects (ASEP) at Queen's University Belfast.
For oligodeoxynucleotides mass spectrum were recorded using a Waters Xevo G2-XS QTof Liquid Chromatography Instrument : Waters Acquity UPLC H-Class, equipped with Waters UPLC Oligonucleotide BEH C18 column, 2.1 x 50 mm, 1.7 µm particle size, part number: 186002350. Buffering system: 75 mM TEAA in HPLC grade H_2O (A) and 75 mM TEAA in HPLC grade MeCN (B). Method: 100% A - 100% B over 20 min linear gradient, flow rate 0.2 mL/min. Mass Spectrometry conditions: Capillary voltage 3 kV, Sampling Cone Voltage – 60 V, source offset 130 V, source temp – 120 °C, desolvation temp 350 °C, cone gas 20 L/h, desolvation gas 500 L/h using dry nitrogen. Instrument monitors accuracy using Leucine Enkephalin.
For oligodeoxynucleotides MALDI-TOF spectra were acquired using an Ultraflex MALDI-TOF (Bruker-Daltonik, Germany) controlled using flex control 3.0 software (Bruker-Daltonik, Germany). The instrument was equipped with a nitrogen laser (λ = 337 nm) set to 32% power and triggered at 25 Hz for a total of 300 shots. Analysis was performed in positive ion reflection mode with reflector voltages of 26.30 kV and 13.75 kV respectively. Detector voltage was set at 1898V and all fragments were isolated as hydrogen adducts [M+H]. Spectra were analysed using Flex analysis 3.0 software (Bruker-Daltonik, Germany) and expressed as m/z. Samples were diluted to a concentration of 200 nmol/L in ultrapure water. Matrix solution (0.5 µL) was transferred to a clean ground steel MALDI sample plate and dried under reduced pressure. This was then overlaid with 0.5 µL of sample and dried under reduced pressure. This process was repeated for the peptide mix used for mass calibration. The 3-hydroxypicolinic acid matrix (3-HPA) was prepared by suspending 3-HPA (50 mg) and diammonium hydrogen citrate (10 mg) in 0.1% (v/v) TFA in 50% (v/v) MeCN : H_2O.

HPLC:

HPLC was performed on a ThermoFisher SpectraSYSTEM modular HPLC system consisting of a P2000 binary gradient pump and UV1000 sample detector. Samples were injected manually via a Rheodyne injection valve. The HPLC was interfaced via an SN4000 controller (Thermo Scientific) to a Windows PC running ChromQuest 5.0 data acquisition software (Thermo Scientific). Buffers were prepared using H_2O purified to 18.2 mΩ by reverse osmosis (Barnstead NANOpure Diamond water purification system), acetonitrile (Aldrich 34851) triethylamine (Aldrich 471283), acetic acid (Aldrich 320099) and CO_2 generated by sublimation of the solid compound.
Analytical HPLC was performed using a Phenomenex Clarity 5µm Oligo-RP (150 x 4.60 mm) column eluting at 1 mL min^{-1}, monitoring at 260 nm using gradients G3, G6, G8.
Preparative HPLC was performed using a Phenomenex Clarity 5µm Oligo-RP (150 x 4.60 mm) column eluting at 1 mL min^{-1}, monitoring at 280 nm using gradients G1, G2, G4, G5, G7.
Triethylammonium acetate (TEAA) buffers were prepared from solutions of acetic acid in H_2O following neutralisation with triethylamine to pH 6.5 and suitable dilution to give final concentrations of: 100 mM TEAA (aq.) (Buffer A); or 100 mM TEAA in 65% (v/v) MeCN : H_2O (Buffer B).

Volatile buffers derived from triethylammonium bicarbonate (TEAB) used for desalting were prepared following dilution of 1 M stock solutions of TEAB in H_2O prepared by bubbling CO_2 through a sintered frit into a mixture of triethylamine and H_2O at 0 °C to give homogenous solutions with measured pH values below 8.0. Stock solutions were stored at 4 °C and used within two days following suitable dilution to give final concentrations of: 100 mM TEAB (aq.), pH 7.8 (Buffer A); or 100 mM TEAB in 65:35 (v/v) MeCN : H_2O, pH 8.2 (Buffer B).

Gradient G1 (preparative – TEAA buffers): 0-10 min, 0% Buffer B; 10-11 min, 0-30% Buffer B; 11-35 min, 30-85% Buffer B; 35-40 min, 85% Buffer B; 40-45 min, 85-100% Buffer B; 45-55 min, 100% Buffer B; 55-65 min, 100-0% Buffer B; 65-75 min, 0% Buffer B.
Gradient G2 (preparative – TEAB buffers): 0-10 min, 0% Buffer B; 10-35 min, 0-35% Buffer B; 35-45 min, 35-100% Buffer B; 45-55 min, 100% Buffer B; 55-65 min, 100-0% Buffer B; 65-75 min, 0% Buffer B.
Gradient G3 (analytical – TEAA buffers): 0-10 min, 0% Buffer B; 10-11 min, 0-7% Buffer B; 11-40 min, 7-25% Buffer B; 40-45 min, 25-100% Buffer B; 45-50 min, 100% Buffer B; 50-60 min, 100-0% Buffer B; 60-75 min, 0% Buffer B.
Gradient G4 (preparative - TEAA buffers): 0-10 min, 0% Buffer B; 10-11 min, 0-30% Buffer B; 11-35 min, 30-70% Buffer B; 35-45 min, 70-100% Buffer B; 45-55 min, 100% Buffer B; 55-65 min, 100-0% Buffer B. 65-80 min, 0% Buffer B.
Gradient G5 (preparative – TEAB buffers): 0-10 min, 0% Buffer B; 10-35 min, 0-50% Buffer B; 35-45 min, 50-100% Buffer B; 45-55min, 100% Buffer B; 55-65 min, 100-0% Buffer B; 65-75 min, 0% Buffer B.
Gradient G6 (analytical - TEAA buffers): 0-10 min, 0% Buffer B; 10-11 min, 0-7% Buffer B; 11-45 min, 7-35% Buffer B; 45-50 min, 35-100% Buffer B; 50-55 min, 100% Buffer B; 55-65 min, 100-0% Buffer B; 65-80 min, 0% Buffer B.
Gradient G7 (preparative – TEAA buffers): 0-10 min, 0% Buffer B; 10-11 min, 0-30% Buffer B; 11-36 min, 30-80% Buffer B; 36-45 min, 80-100% Buffer B; 45-50 min, 100% Buffer B; 50-60 min, 100-0% Buffer B. 60-70 min, 0% Buffer B.
Gradient G8 (analytical – TEAA buffers): 0-5 min, 0% Buffer B; 5-35 min, 0-17% Buffer B; 35-40 min, 17-27% Buffer B; 40-48min, 27-100% Buffer B; 48-50 min, 100% Buffer B; 50-60 min, 100-0% Buffer B; 60-70 min, 0% Buffer B.

Section B. Experimental Procedure and Material Characterisation

5'-deoxythymidine-5'-selenocyanate (**2**).

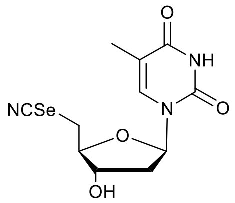

Under a gentle stream of argon, a microwaveable test tube (10 mL) was sequentially charged, in quick succession, with 5'-*O*-tosylthymidine (396 mg, 1.0 mmol), potassium selenocyanate (216 mg, 1.5 mmol, 1.5 eq), anhydrous MeCN (3 mL) and a stir bar. The tube was sealed, and the suspension was stirred to achieve homogeneity and then subject to microwave irradiation (sealed vessel, 100 °C, 20 W, 1.5 h). The reaction mixture was extracted from the vessel in methanol (10 mL) and stored at -20 °C under inert conditions. This was repeated a total of twenty times (over 4 days), the extractates were then combined and stirred at ambient temperature during addition of benzyl bromide (1.40 mL, 11.8 mmol, 0.59 eq). After 60 min complete consumption of excess potassium selenocyanate was observed by tlc and the quenched reaction mixture was reduced in vacuo. After the volume had been reduced by half, silica gel (40 g) was added and residual solvent removed. The crude material was purified by silica gel column chromatography eluting with a gradient of 5–15% (v/v) methanol in DCM. Fractions containing pure **2** were combined and reduced in vacuo to yield a cream, electrostatically-charged amorphous solid (4.94 g, 75%). Characterisation consistent with the literature.[5]

DMTrdA^{PAc}*p*SedT (**4a**).

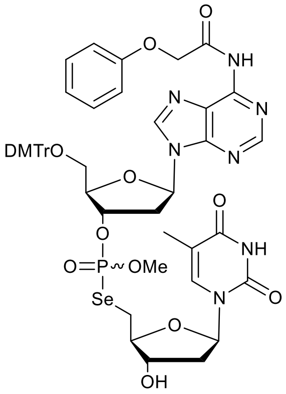

To a stirred solution of 5′-(4,4′-dimethoxytrityl)-N^6-phenoxyacetyl-2′-deoxyadenosine-3′-[methyl-(*N*,*N*-diisopropyl)]-phosphoramidite (3.00 g, 3.53 mmol) in anhydrous MeCN (30 mL) at ambient temperature, under argon, was added 5-(ethylthio)-1*H*-tetrazole (1.84 g, 14.12 mmol, 4.0 eq) in one portion. After 30 min, H_2O (1 mL) was added and stirring continued for a further 15 min. The reaction mixture was diluted with ethyl acetate (300 mL) and washed with satd. aqueous sodium carbonate (3 x 200 mL) and brine (90 mL). The organics were dried over sodium sulfate, filtered and solvents removed in vacuo to yield a white foamy solid of 5′-*O*-(4,4′-dimethoxytrityl)-N^6-phenoxyacetyl-2′-deoxyadenosine-3′-*O*-(methyl)-*H*-phosphonate (**3a**, 2.70 g, 100%). This was stored under argon at -20 °C. ^{31}P NMR (162 MHz, MeCN, D_2O external lock) δ_p = 9.72 and 9.67. Within 24 h, to a stirred solution of *H*-phosphonate **3a** (2.70 g, 3.53 mmol, 1.5 eq) in anhydrous MeCN (25 mL) under argon was added a solution of 5′-deoxythymidine-5′-selenocyanate (**2**, 0.775 g, 2.35 mmol) and 2,6-lutidine (1.36 mL, 11.75 mmol, 5.0 eq) in anhydrous MeCN (25 mL) (this required gentle heat and sonication to effect dissolution) at ambient temperature and in the absence of light. These conditions were maintained for 45 min. The reaction mixture was concentrated to a viscous oil under reduced pressure. The residue was dissolved in the minimum volume of 1% (v/v) MeOH in DCM (10 mL) containing 0.1% (v/v) pyridine and purified by silica gel column chromatography, eluting with 1-5% (v/v) MeOH in DCM with 0.1% (v/v) pyridine. Fractions containing pure product (as a mixture of diastereoisomers) were combined, concentrated to a viscous oil in vacuo and diluted in a minimum volume of DCM (ca. 10 mL) and 50% (v/v) diethyl ether/*n*-hexane added until the first appearance of turbidity. Following addition of DCM (ca. 0.2 mL), pure product was precipitated from vigorously-stirred 50% (v/v) diethyl ether/*n*-hexane (300 mL) at 0 °C and the fine powder isolated following filtration through an S4 sintered funnel and washed with ice cold 50% (v/v) diethyl ether/*n*-hexane (2 x 100 mL) to give **4a** as a cream amorphous solid (2.39 g, 95%).

^{1}H NMR (600 MHz, DMSO-d_6) δ_H = 11.34, 11.34 (1H, 2 x s, T-N^{3}H), 10.96 (1H, s, A-N^{6}H), 8.62, 8.61 (1H, 2 x s, A-H2), 8.53, 8.52 (1H, 2 x s, A-H8), 7.47 (1H, m, T-H6), 7.30-7.35 (4H, m, DMTr-H), 7.17-7.25 (7H, m, 5 x DMTr-H, 2 x PAc-H), 6.96-6.99 (3H, m, PAc-H), 6.79-6.83 (4H, m, DMTr-H), 6.49-6.52 (1H, m, A-H1′), 6.18-6.21 (1H, m, T-H1′), 5.49, 5.49 (1H, 2 x d, $^3J_{HH}$ = 6, 6 Hz, 3′-OH), 5.34-5.37 (1H, m, A-H3′), 5.04, 5.04 (2H, 2 x s, Pac CH_2), 4.33-4.39 (1H, m A-H4′), 4.17-4.20 (1H, m, T-H4′), 3.92-3.95 (1H, m, T-H3′), 3.71-3.74 (9H, m, 2 x Ar-OCH_3, POCH_3), 3.18-3.33 (4H, m, A-H5′,H5′′, T-H5′,H5′′), 3.09-3.16(1H, m, A-H2′) 2.70-2.76 (1H, m, A-H2′′), 2.24-2.28 (1H, m, T-H2′) and 2.07-2.11 (1H, m, T-H2′′), 1.76, 1.76 (3H, 2 x s, T-CH_3).

^{13}C NMR (for reference see **Figure S13**).

^{31}P NMR (162 MHz, DCM, D_2O external lock) δ_p = 22.19 ($^1J_{PSe}$ = 485 Hz), and 22.02 ($^1J_{PSe}$ = 489 Hz).

^{77}Se NMR (114 MHz, DMSO-d_6 with external 0.25M KSeCN / D_2O Insert) δ_{Se} = 95.77 (d, $^1J_{SeP}$ = 488 Hz) and 95.02 (d, $^1J_{SeP}$ = 489 Hz).

HRMS *m/z*: $C_{50}H_{51}N_7O_{13}P^{80}Se$ [M-H]$^-$ calcd: 1068.2455, found: 1068.2449.

DMTrdC^{Ac}*p*SedT (**4b**).

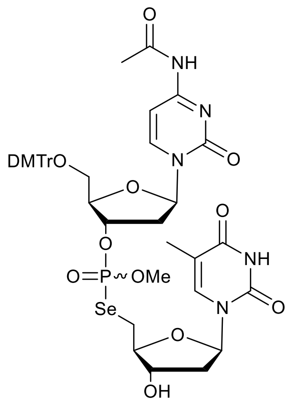

To a stirred solution of 5′-(4,4′-dimethoxytrityl)-N^4-acetyl-2′-deoxycytidine-3′-*O*-[methyl-(*N,N*-diisopropyl)]-phosphoramidite (3.00 g, 4.10 mmol) in anhydrous MeCN (30 mL) at ambient temperature, under argon, was added 5-(ethylthio)-1*H*-tetrazole (2.13 g, 16.40 mmol, 4.0 eq) in one portion. After 30 min, H_2O (1 mL) was added and stirring continued for a further 15 min. The reaction mixture was diluted with ethyl acetate (300 mL) and washed with satd. aqueous sodium carbonate (3 x 200 mL) and brine (90 mL). The organics were dried over sodium sulfate, filtered and solvents removed in vacuo to yield a yellow foamy solid of 5′-*O*-(4,4′-dimethoxytrityl)-N^4-acetyl-2′-deoxycytidine-3′-*O*-(methyl)-*H*-phosphonate (**3b**, 2.66 g, 100%). This was stored under argon at -20 °C. ^{31}P NMR (162 MHz, MeCN, D_2O external lock) δ_p = 9.74 and 9.62. Within 24 h, to a solution of *H*-phosphonate **3b** (2.33 g, 3.59 mmol, 1.5 eq) in anhydrous MeCN (25 mL) under argon was added a solution of 5′-deoxythymidine-5′-selenocyanate (**2**, 0.792 g, 2.39 mmol) and 2,6-lutidine (1.38 mL, 12.0 mmol, 5.0 eq) in anhydrous MeCN (25 mL) (requires gentle heat and sonication to effect dissolution) at ambient temperature and in the absence of light. These conditions were maintained for 60 min. The reaction mixture was concentrated to a viscous oil under reduced pressure. The residue was dissolved in the minimum volume of 1% (v/v) MeOH in DCM (10 mL) containing 0.1% (v/v) pyridine and purified by silica gel column chromatography, eluting with 1-7% (v/v) MeOH in DCM with 0.1% (v/v) pyridine. Fractions containing pure product (as a mixture of diastereoisomers) were combined, concentrated to a viscous oil in vacuo and diluted in a minimum volume of DCM (ca. 10 mL) and 50% (v/v) diethyl ether/*n*-hexane added until the first appearance of turbidity. Following addition of DCM (ca. 0.2 mL), pure product was precipitated from vigorously-stirred 50% (v/v) diethyl ether/*n*-hexane (300 mL) at 0 °C and the fine powder isolated following filtration through an S4 sintered funnel and washed with ice cold 50% (v/v) diethyl ether/*n*-hexane (2 x 100 mL) to give **4b** as a cream amorphous solid (2.20 g, 96%).

^{1}H NMR (600 MHz, DMSO-d_6) δ_H = 11.31, 11.31 (1H, 2 x s, T-N^{3}H), 10.92, 10.91 (1H, 2 x s, C-N^{4}H) 8.09 (1H, d, $^3J_{HH}$ = 7.6 Hz, C-H6), 7.46, 7.46 (1H, m, T-H6), 7.36-7.38 (2H, m, DMTr-H), 7.30-7.32 (2H, m, DMTr-H), 7.21-7.26 (5H, m, DMTr-H), 7.09, 7.09 (1H, 2 x d, $^3J_{HH}$ = 7.5 Hz, C-H5), 6.88-6.90 (4H, m, DMTr-H), 6.11-6.19 (2H, m, T-H1′, C-H1′), 5.46, 5.46 (1H, 2 x d, $^3J_{HH}$ = 4.5, 4.5 Hz, 3′-OH), 5.04-5.07 (1H, m, C-H3′), 4.27-4.31 (1H, m, C-H4′), 4.15-4.18 (1H, m, T-H4′), 3.89-3.92 (1H, m, T-H3′), 3.74 (6H, m, 2 x Ar-OCH$_3$), 3.67-3.70 (3H, m, POCH$_3$), 3.05-3.19 (2H m, T-H5′, H5′′), 2.68-2.72 (1H, m, C-H5′), 2.52-2.53 (2H, C-H2′, H2′′), 2.37-2.42 (1H, m, C-H5′′), 2.23-2.28 (1H, m, T-H2′) and 2.07-2.10 (4H, m, C(O)CH$_3$, T-H2′′), 1.77, 1.77 (3H, 2 x s, T-CH$_3$).

^{13}C NMR (for reference see **Figure S18**).

^{31}P NMR (243 MHz, DCM, D_2O external lock) δ_p = 22.14 ($^1J_{PSe}$ = 487 Hz) and 22.06 ($^1J_{PSe}$ = 488 Hz).

^{77}Se NMR (114 MHz, DMSO-d_6 with external 0.25M KSeCN / D_2O Insert) δ_{Se} = 96.45 (d, $^1J_{SeP}$ = 488 Hz) and 94.34 (d, $^1J_{SeP}$ = 484 Hz).

HRMS *m/z*: $C_{43}H_{47}N_5O_{13}P^{80}Se$ $[M-H]^-$ calcd: 952.2073, found: 952.2078.

MMTrdG^{iBu}*p*SedT (**4c**).

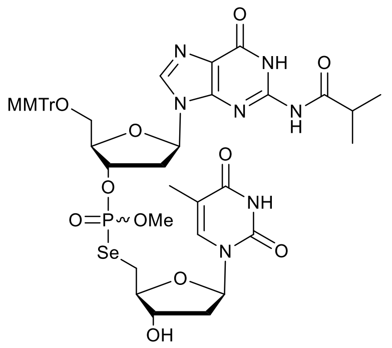

To a stirred solution of 5′-(4-methoxytrityl)-*N*^{2}-isobutyryl-2′-deoxyguanosine-3′-*O*-[methyl-(*N,N*-diisopropyl)]-phosphoramidite (2.00 g, 2.59 mmol) in anhydrous acetonitrile (20 mL) at ambient temperature, under argon, was added 5-(ethylthio)-1*H*-tetrazole (1.35 g, 10.36 mmol, 4.0 eq) in one portion. After 30 min, H_2O (0.7 mL) was added and stirring maintained for a further 15 min. The reaction mixture was diluted with ethyl acetate (250 mL) and washed with satd. aqueous sodium carbonate (3 x 100 mL) and brine (50 mL). The organics were dried over sodium sulfate, filtered and solvents removed in vacuo to yield a white amorphous solid of 5′-*O*-(4-methoxytrityl)-*N*^{2}-isobutyryl-2′-deoxyguanosine-3′-*O*-(methyl)-*H*-phosphonate (**3c**, 1.75 g, 98%). This was stored under argon at -20 °C. ^{31}P NMR (162 MHz, MeCN, D_2O external lock) δ_p = 10.44 and 10.31. Within 24 h, to a solution of *H*-phosphonate **3c** (1.34 g, 1.95 mmol, 1.5 eq) in 1:1 (v/v) anhydrous MeCN / anhydrous DCM (40 mL) under argon was added a solution of 5′-deoxythymidine-5′-selenocyanate (**2**, 0.430 g, 1.30 mmol) and 2,6-lutidine (752 μL, 6.50 mmol, 5.0 eq) in anhydrous MeCN (15 mL) (requires gentle heat and sonication to effect dissolution) at ambient temperature and in the absence of light. These conditions were maintained for 45 min by which time the solution had went clear. The reaction mixture was concentrated to a viscous oil under reduced pressure. The residue was dissolved in the minimum volume of 1% (v/v) MeOH in DCM (10 mL) containing 0.1% (v/v) pyridine and purified by silica gel column chromatography, eluting with 1-6 % (v/v) MeOH in DCM with 0.1% (v/v) pyridine. Fractions containing pure product (as a mixture of diastereoisomers) were combined, concentrated to a viscous oil in vacuo and diluted in a minimum volume of DCM (ca. 10 mL) and 50% (v/v) diethyl ether/*n*-hexane added until the first appearance of turbidity. Following addition of DCM (ca. 0.2 mL), pure product was precipitated from vigorously-stirred 50% (v/v) diethyl ether/*n*-hexane (300 mL) at 0 °C and the fine powder isolated following filtration through an S4 sintered funnel and washed with ice cold 50% (v/v) diethyl ether/n-hexane (2 x 100 mL) to give **4c** as a cream amorphous solid (1.07 g, 83%).

^{1}H NMR (600 MHz, DMSO-*d*$_6$) δ_H = 12.06 (1H, s, G-N^{1}H), 11.61, 11.56 (1H, 2 x s, G-N^{2}H), 11.32 (1H, s, T-N^{3}H), 8.11 (1H, s, G-H8), 7.46 (1H, m, T-H6), 7.34-7.37 (4H, m, DMTr-H), 7.19-7.29 (8H, m, DMTr-H), 6.82-6.85 (2H, m, DMTr-H), 6.26-6.28 (1H, m, G-H1′), 6.16-6.18 (1H, m, T-H1′), 5.47 (1H, br s, 3′-OH), 5.16-5.17 (1H, m, G-H3′), 4.22-4.28 (1H, m, G-H4′), 4.16 (1H, m, T-H4′), 3.88-3.92 (1H, m, T-H3′), 3.69-3.73 (6H, m, Ar-OCH_3, POCH_3), 3.06-3.31 (4H, m, G-H5′, H5′′, T-H5′, H5′′), 3.00-3.05 (1H, m, G-H2′), 2.65-2.77 (2H, m, T-H2′,G-H2′′), 2.23-2.28 (1H, m, T-H2′′), 2.07-2.11 (1H, m, C$H(CH_3)_2$), 1.76, 1.76 (3H, 2 x s, T-CH_3), 1.13 (6H, br d, $^3J_{HH}$ = 6.3 Hz, CH(C$H_3)_2$).

^{13}C NMR (for reference see **Figure S23**).

^{31}P NMR (243 MHz, DCM, D_2O external lock) δ_p = 22.47 ($^1J_{PSe}$ = 491 Hz) and 22.08 ($^1J_{PSe}$ = 490 Hz).

^{77}Se NMR (114 MHz, DMSO-*d*$_6$ with external 0.25M KSeCN / D_2O Insert) δ_{Se} = 96.61 (d, $^1J_{SeP}$ = 494 Hz) and 94.59 (d, $^1J_{SeP}$ = 489 Hz).

HRMS *m/z*: $C_{45}H_{49}N_7O_{12}P^{80}Se$ [M-H]$^-$ calcd: 990.2342, found: 990.2343.

DMTrT*p*SedT (**4d**).

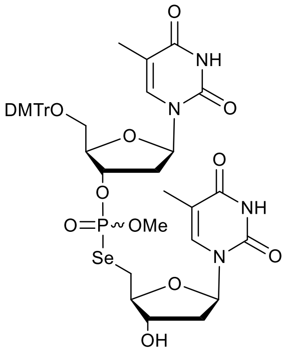

To a stirred solution of 5′-(4,4′-dimethoxytrityl)-thymidine-3′-*O*-[methyl-(*N,N*-diisopropyl)]-phosphoramidite (2.00 g, 2.83 mmol) in anhydrous acetonitrile (10 mL) at ambient temperature, under argon, was added 5-(ethylthio)-1*H*-tetrazole (1.47 g, 11.32 mmol, 4.0 eq). After 30 min, H_2O (0.7 mL) was added and stirring maintained for a further 15 min. The reaction mixture was diluted with ethyl acetate (200 mL) and washed with satd. aqueous sodium carbonate (3 x 120 mL) and brine (50 mL). The organics were dried over sodium sulfate, filtered and the solvents removed in vacuo to produce a white foamy solid of 5′-*O*-(4,4′-dimethoxytrityl)-thymidine-3′-*O*-(methyl)-*H*-phosphonate (**3d**, 1.73 g, 98%). This was stored under argon at -20 °C. ^{31}P NMR (162 MHz, MeCN, D_2O external lock) δ_p = 10.07 and 10.04. Within 24 h, to a solution of *H*-phosphonate **3d** (1.11 g, 1.79 mmol, 1.5 eq) in anhydrous MeCN (7 mL) under argon was added a solution of 5′-deoxythymidine-5′-selenocyanate (**2**, 0.396 g, 1.19 mmol) and 2,6-lutidine (688 µL, 5.95 mmol, 5.0 eq) in anhydrous MeCN (15 mL) (requires gentle heat and sonication to effect dissolution) at ambient temperature and in the absence of light. These conditions were maintained for 45 min. The reaction mixture was concentrated to a viscous oil under reduced pressure. The residue was dissolved in the minimum volume of 1% (v/v) MeOH in DCM (10 mL) containing 0.1% (v/v) pyridine and purified by silica gel column chromatography, eluting with 1-4 % (v/v) MeOH in DCM with 0.1% (v/v) pyridine. Fractions containing pure product (as a mixture of diastereoisomers) were combined, concentrated to a viscous oil in vacuo and diluted in a minimum volume of DCM (ca. 10 mL) and 50% (v/v) diethyl ether/*n*-hexane added until the first appearance of turbidity. Following addition of DCM (ca. 0.2 mL), pure product was precipitated from vigorously-stirred 50% (v/v) diethyl ether/*n*-hexane (300 mL) at 0 °C and the fine powder isolated following filtration through an S4 sintered funnel and washed with ice cold 50% (v/v) diethyl ether/n-hexane (2 x 100 mL) to give **4d** as a cream amorphous solid (1.08 g, 97%).

^{1}H NMR (600 MHz, DMSO-d_6) δ_H = 11.38 (1H, s, T-N^3<u>H</u>) 11.31 (1H, s, T-N^3<u>H</u>′), 7.49 (1H, m, T-H6), 7.44-7.45 (1H, m, T-H6′), 7.37-7.39 (2H, m, DMTr-<u>H</u>), 7.30-7.32 (2H, m, DMTr-<u>H</u>), 7.21-7.26 (5H, m, DMTr-<u>H</u>), 6.87-6.90 (4H, m, DMTr-<u>H</u>), 6.15-6.22 (2H, m, H1′), 5.45 (1H, d, $^3J_{HH}$ = 4.4 Hz, 3′-O<u>H</u>), 5.14-5.16 (1H, m, H3′), 4.14-4.20 (2H, m, H4′, H4′′) 3.87-3.90 (1H, m, H3′′), 3.73 (6H, m, Ar-OC<u>H</u>$_3$), 3.66-3.70 (3H, m, POC<u>H</u>$_3$), 3.28-3.30 (1H, m, H5′), 3.23-3.26 (1H, m, H5′′), 3.04-3.20 (2H, m, H5′, H5′′), 2.52 (2H, m, H2′, H2′′), 2.21-2.27 (1H, m, H2′), 2.05-2.10 (1H, m, H2′′), 1.76, 1.75 (3H, 2 x s, OC<u>H</u>$_3$′), 1.48, 1.48 (3H, 2 x s, OC<u>H</u>$_3$).

^{13}C NMR (for reference see **Figure S28**).

^{31}P NMR (243 MHz, MeCN, D_2O external lock) δ_p = 22.29 ($^1J_{PSe}$ = 486 Hz) and 22.27 ($^1J_{PSe}$ = 487 Hz).

^{77}Se NMR (114 MHz, DMSO-d_6 with external 0.25M KSeCN / D_2O Insert) δ_{Se} = 94.68 (d, $^1J_{SeP}$ = 483 Hz) and 92.32 (d, $^1J_{SeP}$ = 483 Hz).

HRMS *m/z*: $C_{42}H_{46}N_4O_{13}P^{80}Se$ $[M-H]^-$ calcd: 925.1964, found: 925.1956.

DMTrdG^{iBu}*p*SedT (**4e**).

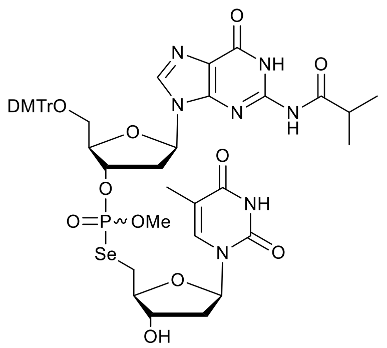

To a stirred solution of 5′-(4,4′-dimethoxytrityl)-*N*^{2}-isobutyryl-2′-deoxyguanosine-3′-*O*-[methyl-(*N,N*-diisopropyl)]-phosphoramidite (2.00 g, 2.49 mmol) in anhydrous MeCN (20 mL) at ambient temperature, under argon, was added 5-(ethylthio)-1*H*-tetrazole (1.30 g, 9.98 mmol, 4.0 eq) in one portion. After 30 min, H_2O (0.7 mL) was added and stirring continued for a further 15 min. The reaction mixture was diluted with ethyl acetate (200 mL) and washed with satd. aqueous sodium carbonate (3 x 150 mL) and brine (60 mL). The first two sodium carbonate washes were combined and back extracted with ethyl acetate (2 x 200 mL). The organics were dried over sodium sulfate, filtered and the solvents removed in vacuo to yield a cream foamy solid of 5′-*O*-(4,4′-dimethoxytrityl)-*N*^{2}-isobutyryl-2′-deoxyguanosine-3′-*O*-(methyl)-*H*-phosphonate (**3e**, 1.75 g, 98%). This was stored under argon at -20 °C. ^{31}P NMR (162 MHz, MeCN, D_2O external lock) δ_p = 9.98 and 9.80. Within 24 h, to a solution of *H*-phosphonate **3e** (1.39 g, 1.95 mmol, 1.5 eq) in anhydrous MeCN (15 mL) and anhydrous DCM (20 mL) under argon was added a solution of 5′-deoxythymidine-5′-selenocyanate (**2**, 0.430 g, 1.30 mmol) and 2,6-lutidine (752 µL, 6.50 mmol, 5.0 eq) in anhydrous MeCN (15 mL) at ambient temperature and in the absence of light. These conditions were maintained for 45 min. The reaction mixture was concentrated to a viscous oil under reduced pressure. The residue was dissolved in the minimum volume of 1% (v/v) MeOH in DCM (10 mL) containing 0.1% (v/v) pyridine and purified by silica gel column chromatography, eluting with 1-6 % (v/v) MeOH in DCM with 0.1% (v/v) pyridine. Fractions containing pure product (as a mixture of diastereoisomers) were combined, concentrated to a viscous oil in vacuo and diluted in a minimum volume of DCM (ca. 10 mL) and 50% (v/v) diethyl ether/*n*-hexane added until the first appearance of turbidity. Following addition of DCM (ca. 0.2 mL), pure product was precipitated from vigorously-stirred 50% (v/v) diethyl ether/*n*-hexane (300 mL) at 0 °C and the fine powder isolated following filtration through an S4 sintered funnel and washed with ice cold 50% (v/v) diethyl ether/*n*-hexane (2 x 100 mL) to give **4e** as a cream amorphous solid (0.935 g, 71%).

^{1}H NMR (600 MHz, DMSO-*d*$_6$) δ_H = 12.07 (1H, s, G-N^{1}H), 11.59, 11.65 (1H, 2 x s, G-N^{2}H), 11.35 (1H, s, T-N^{3}H), 8.13 (1H, s, G-H8) 7.47 (1H, m, T-H6), 7.33-7.35 (2H, m, DMTr-H), 7.25-7.27 (2H, m, DMTr-H), 7.19-7.22 (5H, m, DMTr-H), 6.80-6.85 (4H, m, DMTr-H), 6.26-6.28 (1H, m, G-H1′), 6.17-6.19 (1H, m, T-H1′), 5.48-5.49 (1H, d, $^3J_{HH}$ = 4.4 Hz, 3′-OH), 5.15-5.18 (1H, m, G-H3′), 4.21-4.29 (1H, m, G-H4′), 4.15-4.18 (1H, m, T-H4′), 3.89-3.92 (1H, m, T-H3′), 3.70-3.73 (9H, m, 2 x Ar-OCH_3, POCH_3), 3.07-3.32 (4H, m, G-H5′, H5′′, T-H5′, H5′′), 3.00-3.05 (1H, m, G-H2′), 2.65-2.78 (2H, m, T-H2′,G-H2′′), 2.23-2.28 (1H, m, T-H2′′), 2.07-2.11 (1H, m, CH(CH_3)$_2$), 1.77, 1.76 (3H, 2 x s, T-CH_3), 1.14 (6H, br d, $^3J_{HH}$ = 6.9 Hz, CH(CH_3)$_2$).

^{13}C NMR (for reference see **Figure S33**).

^{31}P NMR (162 MHz, DCM, D_2O external lock) δ_p = 21.87 ($^1J_{PSe}$ = 495 Hz) and 21.51 ($^1J_{PSe}$ = 493 Hz).

^{77}Se NMR (114 MHz, DMSO-*d*$_6$ with external 0.25M KSeCN / D_2O Insert) δ_{Se} = 97.50 (d, $^1J_{SeP}$ = 493 Hz) and 95.41 (d, $^1J_{SeP}$ = 496 Hz).

HRMS *m/z*: $C_{46}H_{51}N_7O_{13}P^{80}Se$ [M-H]$^-$ calcd: 1020.2448, found: 1020.2449.

DMTrdA^{PAc}*p*SedT-Phosphoramidite (**5a**).

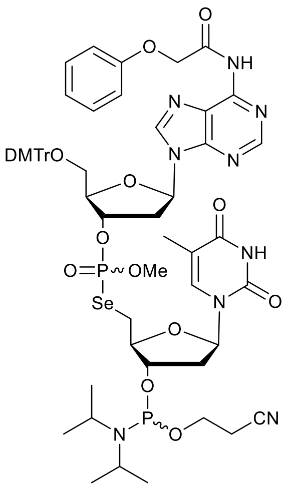

DMTrdA^{PAc}*p*SedT (**4a**, 535 mg, 0.5 mmol) was co-evaporated with anhydrous, deacidified DCM (3 x 5 mL). The solids were dissolved in anhydrous DCM (5 mL) and anhydrous *N,N*-diisopropylethylamine (260 µL, 1.5 mmol, 3.0 eq). The solution was stirred at room temperature under argon during dropwise addition of 2-cyanoethyl-*N,N*-diisopropylchlorophosphoramidite (223 µL, 1 mmol, 2.0 eq) over 3 min. These conditions were maintained for a further 45 min and the reaction quenched following addition of anhydrous MeOH (30 µL, 0.75 mmol, 1.5 eq) and stirring for a further 15 min. The reaction mixture was diluted with DCM (20 mL), washed with brine (10 mL) and dried over sodium sulfate. The organics were filtered and reduced under vacuum to give a white foamy solid. This was dissolved in the minimum anhydrous DCM containing 0.5% (v/v) Et_3N and purified via silica gel column chromatography using isocratic elution with anhydrous / degassed 1:1 (v/v) acetone:DCM containing 0.5% Et_3N (v/v). Fractions containing pure product were combined and reduced in vacuo to afford a yellow viscous oil. Pure **5a** was isolated following precipitation from 1:1 (v/v) diethyl ether / *n*-hexane (100 mL), filtration of the finely-divided solids through an S4 sintered funnel and washing with ice-cold 1:1 (v/v) diethyl ether / *n*-hexane (2 x 50 mL) under a gentle stream of argon. The product was obtained as an electrostatic amorphous white solid after drying in vacuo (470 mg, 74%).

^{31}P NMR (243 MHz, MeCN, D_2O external lock) δ_p = 149.20, 149.13, 149.07, 149.02 and 22.04 ($^1J_{PSe}$ = 485 Hz), 21.93 ($^1J_{PSe}$ = 487 Hz) 21.88 ($^1J_{PSe}$ = 485 Hz), 21.74 ($^1J_{PSe}$ = 485 Hz).

^{1}H NMR (for reference see **Figure S38**).

HRMS *m/z*: $C_{59}H_{68}N_9O_{14}P_2{}^{80}Se$ $[M-H]^-$ calcd: 1268.3526, found: 1268.3528.

DMTrdC^{Ac}*p*SedT-Phosphoramidite (**5b**).

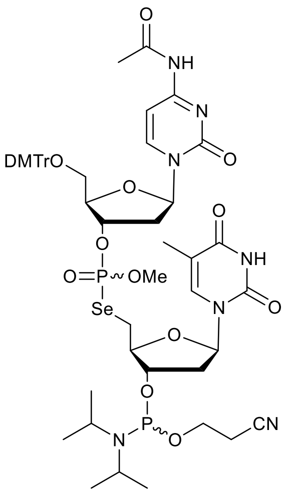

DMTrdC^{Ac}*p*SedT (**4b**, 476 mg, 0.5 mmol) was co-evaporated with anhydrous, deacidified DCM (3 x 5 mL). The solids were dissolved in anhydrous DCM (5 mL) and anhydrous *N,N*-diisopropylethylamine (260 µL, 1.5 mmol, 3.0 eq). The solution was stirred at room temperature under argon during dropwise addition of 2-cyanoethyl-*N,N*-diisopropylchlorophosphoramidite (223 µL, 1 mmol, 2.0 eq) over 3 min. These conditions were maintained for a further 45 min and the reaction quenched following addition of anhydrous MeOH (30 µL, 0.75 mmol, 1.5 eq) and stirring for a further 15 min. The reaction mixture was diluted with DCM (20 mL), washed with brine (10 mL) and dried over sodium sulfate. The organics were filtered and reduced under vacuum to give an off-white foamy solid. This was dissolved in the minimum anhydrous DCM containing 0.5% (v/v) Et_3N and purified via silica gel column chromatography using isocratic elution with anhydrous / degassed 1:1 (v/v) acetone:DCM containing 0.5% Et_3N (v/v). Fractions containing pure product were combined and reduced in vacuo to afford a yellow viscous oil. Pure **5b** was isolated following precipitation from 1:1 (v/v) diethyl ether / *n*-hexane (100 mL), filtration of the finely-divided solids through an S4 sintered funnel and washing with ice-cold 1:1 (v/v) diethyl ether / *n*-hexane (2 x 50 mL) under a gentle stream of argon. The product was obtained as an electrostatic amorphous white solid after drying in vacuo (458 mg, 79%).

^{31}P NMR (162 MHz, MeCN, D_2O external lock) δ_p = 149.27, 149.19 (2P), 149.14 and 21.95 ($^1J_{PSe}$ = 486 Hz), 21.93 ($^1J_{PSe}$ = 484 Hz), 21.79 ($^1J_{PSe}$ = 484 Hz), 21.74 ($^1J_{PSe}$ = 485 Hz).

^{1}H NMR (for reference see **Figure S41**).

HRMS *m/z*: $C_{52}H_{64}N_7O_{14}P_2{}^{80}Se$ $[M-H]^-$ calcd: 1152.3152, found: 1152.3158.

MMTrdG^{iBu}*p*SedT-Phosphoramidite (**5c**).

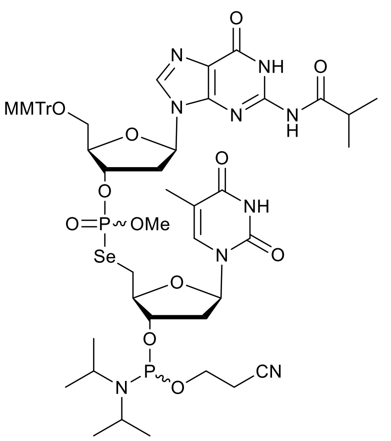

MMTrdG^{iBu}*p*SedT (**4d**, 495 mg, 0.5 mmol) was co-evaporated with anhydrous, deacidified DCM (3 x 5 mL). The solids were dissolved in anhydrous DCM (5 mL) and anhydrous *N,N*-diisopropylethylamine (260 µL, 1.5 mmol, 3.0 eq) The solution was stirred at room temperature under argon during dropwise addition of 2-cyanoethyl-*N,N*-diisopropylchlorophosphoramidite (223 µL, 1 mmol, 2.0 eq) over 3 min. These conditions were maintained for a further 45 min and the reaction quenched following addition of anhydrous MeOH (30 µL, 0.75 mmol, 1.5 eq) and stirring for a further 15 min. The reaction mixture was diluted with DCM (20 mL), washed with brine (10 mL) and dried over sodium sulfate. The organics were filtered and reduced under vacuum to give an off-white foamy solid. This was dissolved in in the minimum anhydrous DCM containing 0.5% (v/v) Et_3N and purified via silica gel column chromatography using isocratic elution with anhydrous / degassed 1:1 (v/v) acetone:DCM containing 0.5% Et_3N (v/v). Fractions containing pure product were combined and reduced in vacuo to afford a yellow viscous oil. Pure **5c** was isolated following precipitation from 1:1 (v/v) diethyl ether / *n*-hexane (100 mL), filtration of the finely-divided solids through an S4 sintered funnel and washing with ice-cold 1:1 (v/v) diethyl ether / *n*-hexane (2 x 50 mL) under a gentle stream of argon. The product was obtained as an electrostatic amorphous white solid after drying in vacuo (475 mg, 80%).

^{31}P NMR (243 MHz, MeCN, D_2O external lock) δ_p = 149.23, 149.14, 149.10, 148.96 and 22.26 ($^1J_{PSe}$ = 489 Hz) 22.17 ($^1J_{PSe}$ = 489 Hz), 21.67 ($^1J_{PSe}$ = 488 Hz), 21.56 ($^1J_{PSe}$ = 487 Hz).

^{1}H NMR (for reference see **Figure S44**).

HRMS *m/z*: $C_{54}H_{66}N_9O_{13}P_2{}^{80}Se$ $[M-H]^-$ calcd: 1190.3421, found: 1190.3422.

DMTrT*p*SedT-Phosphoramidite (**5d**).

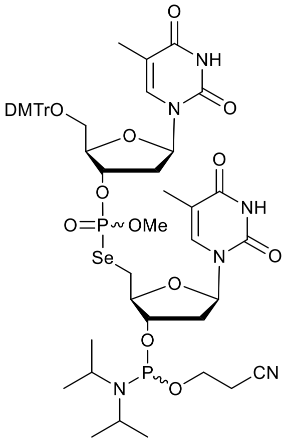

DMTrT*p*SedT (**4e**, 555 mg, 0.6 mmol) was co-evaporated with anhydrous, deacidified DCM (3 x 5 mL). The solids were dissolved in anhydrous DCM (5 mL) and anhydrous *N,N*-diisopropylethylamine (313 µL, 1.8 mmol, 3.0 eq). The solution was stirred at room temperature under argon during dropwise addition of 2-cyanoethyl-*N,N*-diisopropylchlorophosphoramidite (266 µL, 1.2 mmol, 2.0 eq) over 3 min. These conditions were maintained for a further 60 min and the reaction quenched following addition of anhydrous MeOH (36 µL, 0.9 mmol, 1.5 eq) and stirring for a further 15 min. The reaction mixture was diluted with DCM (20 mL), washed with brine (10 mL) and dried over sodium sulfate. The organics were filtered and reduced under vacuum to give an off-white foamy solid. This was dissolved in the minimum anhydrous DCM containing 0.5% (v/v) Et_3N and purified via silica gel column chromatography using isocratic elution with anhydrous / degassed 1:1 (v/v) acetone:DCM containing 0.5% Et_3N (v/v). Fractions containing pure product were combined and reduced in vacuo to afford a yellow viscous oil. Pure **5d** was isolated following precipitation from 1:1 (v/v) diethyl ether / *n*-hexane (100 mL), filtration of the finely-divided solids through an S4 sintered funnel and washing with ice-cold 1:1 (v/v) diethyl ether

/ *n*-hexane (2 x 50 mL) under a gentle stream of argon. The product was obtained as an electrostatic amorphous white solid after drying in vacuo. (585 mg, 86%).

^{31}P NMR (243 MHz, MeCN, D_2O external lock) δ_P = 149.20, 149.13 (2P), 149.06, 22.19 ($^1J_{PSe}$ = 486 Hz), 22.06 ($^1J_{PSe}$ = 483 Hz), 21.96 ($^1J_{PSe}$ = 486 Hz), 21.91 ($^1J_{PSe}$ = 483 Hz).

^{1}H NMR (for reference see **Figure S47**).

HRMS *m/z*: $C_{51}H_{63}N_6O_{14}P_2{}^{80}Se$ [M-H]$^-$ calcd: 1125.3043, found: 1125.3035.

5′-deoxythymidine-5′-selenocyanate-3′-*O*-succinate (**6**).

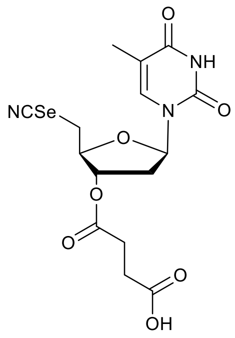

To a stirred solution of 5′-deoxythymidine-5′-selenocyanate (**2**) (100 mg, 0.3 mmol) and DMAP (30 mg, 0.24 mmol, 0.8 eq) in anhydrous pyridine (3 mL) under argon at ambient temperature was added succinic anhydride (30 mg, 0.3 mmol, 1 eq) portion wise over 30 min. The reaction mixture was stirred under these conditions overnight in the absence of light. Once complete, the reaction mixture was reduced in vacuo to afford a brown gum. Residual pyridine was removed via co-evaporation with toluene (3 x 5mL). The gum was redissolved in a mixture of 1:1 (v/v) $CHCl_3$ / MeCN (10 mL), diluted further with EtOAc (60 mL) and washed with ice cold 10% (w/v) citric acid (aq) (2 x 10 mL). The organics were dried over sodium sulfate, filtered and reduced in vacuo to an orange foam. (87 mg, 67%).

^{1}H NMR (600 MHz, DMSO-*d*$_6$) δ_H = 12.25 (1H, s, C(O)O<u>H</u>), 11.37 (1H, s, N<u>H</u>), 7.57 (1H, s, H6), 6.14-6.19 (1H, m, H1′), 5.21-5.25 (1H, m, H3′), 4.14-4.21 (1H, m, H4′), 3.33-3.46 (2H, m, H5′, H5″), 2.51-2.59 (5H, m, H2′, (C<u>H</u>$_2$)$_2$), 2.25-2.29 (1H, m, H2″). 1.79 (3H, s, C<u>H</u>$_3$).

^{77}Se NMR (114 MHz, DMSO-*d*$_6$ with external 0.25M KSeCN / D_2O Insert) δ_{Se} = 289.20 (diselenide), 187.12 (**6**).

HRMS *m/z*: $C_{15}H_{16}N_3O_7{}^{80}Se$ [M-H]$^-$ calcd: 430.0154, found: 430.0193.

5′-deoxythymidine-5′-selenocyanate-functionalised CPG (**7**).

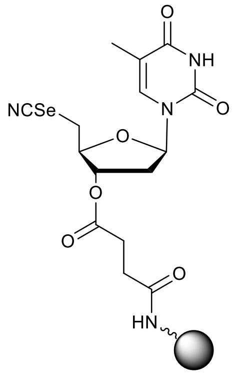

To a stirred solution of 5′-deoxythymidine-5′-selenocyanate-3′-*O*-succinate (**6**) (77 mg, 0.18 mmol) in anhydrous DCM (2 mL) was added 4-nitrophenol (25 mg, 0.18 mmol, 1 eq) and *N*,*N*′-dicyclohexylcarbodiimide (DCC) (92 mg, 0.45 mmol, 2.5 eq). The reaction mixture was stirred for 2 h under argon and in the absence of light during which precipitation of *N*,*N*′-dicyclohexyl urea was observed. The reaction mixture was filtered, the solids washed with anhydrous DCM (2 x 1 mL) and the combined organics added to a suspension of Amino SynBase 500/110 CPG (500 mg) in anhydrous DMF (2 mL), followed by addition of Et_3N (100 µL, 0.7 mmol, 4 eq). The suspension was shaken on an orbital shaker at room temperature under argon for 5 h. The CPG was isolated following filtration, washed with DMF (3 x 2 mL), MeOH (3 x 2 mL) and Et_2O (3 x 2 mL). The ninhydrin positive material was then capped with a solution of acetic anhydride (100 µL) and DMAP (5 mg) in anhydrous pyridine (3 mL) for 30 min at room temperature under argon. Following this treatment, the CPG gave a negative ninhydrin reaction. The CPG loading was determined following a test reaction as follows. Selenocyanate-functionalised CPG **7** (30 mg) was washed with anhydrous MeCN (3 x 2 mL) and to this material was added a solution of 5′-*O*-(4,4′-dimethoxytrityl)-thymidine-3′-*O*-(2-cyanoethyl)-*H*-phosphonate[6] in anhydrous MeCN (0.1 M, 1 mL). This suspension was shaken at room temperature for 1 h, filtered and the support washed with DMF (3 x 2 mL), MeOH (3 x 2 mL) and Et_2O (3 x 2 mL). Trityl loading = 39 µmol g^{-1}. (Determined by $HClO_4$ assay of DMTr cation).

Oligodeoxynucleotide synthesis:

Modified oligodeoxynucleotides were synthesised trityl-on using the manual syringe method on 1 µmol scale in duplicate (**ODN 1**, **ODN 2**, **ODN 3**, **ODN 4**, **ODN 5**) or on a 1.75 µmol scale (**ODN 7**). Modified oligodeoxynucleotide **ODN 6** was synthesised trityl-on under automated conditions using a MerMade 4 DNA synthesiser on 1 µmol scale.
Prior to use, dimer phosphoramidites (**5a-d**) were co-evaporated with anhydrous diluent grade MeCN (3 x 5 mL), dissolved in the same solvent to give a final concentration of 0.1 M and the solution filtered under argon through a Whatman syringe filter (13 mm, 0.45 µm PTFE filter).

Native oligodeoxynucleotides were purchased (HPLC-purified) from ATDBio Ltd or prepared trityl-on using a MerMade 4 DNA synthesiser under standard automated solid-phase synthesis conditions (1 µmol scale), purified using RP-HPLC and detritylated in 80% (v/v) acetic acid (aq).

d(TSeTTSeTT) (**ODN 1**)

ODN 1 was prepared from a standard T-succinylate-CPG (Synbase 500 Å / 110 S) (Link Technologies, 39 µmol g^{-1}) using the following synthesis cycle:

1. Wash (3 x 2 mL, MeCN)
2. Deblock (3 x 2 mL, 3% (w/v) TCA/DCM)
3. Wash (3 x 2 mL, MeCN)
4. Couple (200 µL 0.1 M dimer phosphoramidite **5d** + 300 µL 0.3 M BTT both in MeCN, 5 min)
5. Wash (3 x 2 mL, MeCN)
6. Cap (500 µL Cap Mix A (9:1 (v/v) THF/acetic anhydride), 500 µL Cap Mix B (10% (m/v) methylimidazole in 8:1 (v/v) THF/pyridine), 45 s (only after first dimer coupling))
7. Wash (3 x 2 mL, MeCN)
8. Oxidise (500 µL 0.02 M I_2 in 0.4% (v/v) pyridine/THF), 45 s
9. Wash (3 x 2 mL, MeCN)
10. Wash (1 x 1 mL, 10% (v/v) pyridine/MeCN)
11. Wash (3 x 2 mL, MeCN)

Following completion of the second synthetic cycle, the two CPG-bound pentamers were separately treated at room temperature with either 1:2:2 (v/v/v) thiophenol/triethylamine/dioxane (1 mL) over 30 min or 0.2 M sodium diethyldithiocarbamate in anhydrous MeCN (1 mL) over 30 min. The demethylated materials were then washed with acetonitrile (3 x 2 mL), dried and the CPG transferred to screw-capped vials with 1 mL AMA (1:1 (v/v) 35% (w/v) NH_3 (aq) : 40% (w/v) $MeNH_2$ (aq)). The reaction mixtures were stored at ambient temperature for 2 h, the CPG removed by filtration using a Corning Co-star centrifuge tube filter (2 mL volume, 0.45 µm cellulose acetate filter) at 13,000 rpm, washed with water (2 x 1 mL) reduced in vacuo and analysed by RP-HPLC. The chromatographic profiles from the crude thiophenol and NaDEC-treated tritylated oligomers were essentially identical and were therefore combined and purified by RP-HPLC using gradient G1. Fractions containing pure material were pooled, reduced in vacuo to ca. 50 µL and to this solution was added 80% (v/v) aqueous acetic acid (1 mL) and stored at ambient temperature for 1 h. The reaction mixture was diluted with absolute ethanol (1 volume) and reduced in vacuo by half. This procedure was repeated but subsequently evaporated to dryness and the residues suspended in TEAB buffer A (1 mL) and centrifuge (10 min, 13,000 rpm). The solution of detritylated oligomer **ODN 1** was subject to desalting by RP-HPLC using buffers derived from volatile salts (gradient G2) and subsequent

co-evaporation with H_2O (6 x 500 μL). Analytical RP-HPLC performed using gradient G3. t_R = 34.91 min. ^{31}P NMR (243 MHz, 40% (v/v) D_2O / H_2O) δ_p = 10.56 ($^1J_{PSe}$ = 393 Hz), 10.48 ($^1J_{PSe}$ = 390 Hz), -1.19 and -1.25. ESI mass spec is available in **Table 1.**

ODN 2, **ODN 3**, **ODN 4** And **ODN 5** were prepared from the appropriate support-bound 8-mers which were commercially sourced from LGC Biosearch and had been prepared from the appropriate nucleoside-glycolate-CPG using ultramild-synthesis reagents. The synthesis cycle above was adapted as follows:

Step 4. Coupling of dimer phosphoramidites **5a** (**ODN 2**), **5b** (**ODN 3**) or **5c** (**ODN 4**) were performed using a double addition: 200 μL 0.1 M phosphoramidite + 300 μL 0.3 M BTT (both in MeCN) - 2 x 7.5 min; **5d** (**ODN 5**) was coupled as described for **ODN 1**

Steps 6, 7, 9 and 10 (capping and downstream washings) were omitted

Post-synthesis, NaDEC was used to effect demethylation and subsequent processing was performed as described for **ODN 1** except that the oligomer sequence **ODN 4** which contained a 5′-terminal 2′-deoxyguanine residue (MMT- and isobutyryl protected) was subject to extended deprotection times using AMA (3 h) and aqueous acetic acid (1.5 h).

d(ASeTCCCGGGAT) (**ODN 2**).

ODN 2 was prepared from synthesis columns containing 5′-DMTr-d[$(C^{Ac})_3(G^{iPrPac})_3A^{Pac}T$]-3′-CPG. The DMTr-ON oligomer was purified by RP-HPLC using gradient G4. Post detritylation the oligomer was desalted by RP-HPLC using gradient G5 followed by co-evaporation with H_2O (6 x 500 μL). Analytical RP-HPLC performed using gradient G6. t_R = 35.30 min. MALDI-TOF and ESI mass spec are available in **Table 1** and **2** respectively.

d(CSeTCCCGGGAG) (**ODN 3**).

ODN 3 was prepared from synthesis columns containing 5′-DMTr-d[$(C^{Ac})_3(G^{iPrPac})_3A^{Pac}G^{iPrPac}$]-3′-CPG. The DMTr-ON oligomer was purified by RP-HPLC using gradient G4. Post detritylation the oligomer was desalted by RP-HPLC using gradient G5 followed by co-evaporation with H_2O (6 x 500 μL). Analytical RP-HPLC performed using gradient G6. t_R = 34.11 min. MALDI-TOF and ESI mass spec are available in **Table 1** and **2** respectively.

d(GSeTCCCGGGAC) (**ODN 4**).

ODN 4 was prepared from synthesis columns containing 5′-DMTr-d[$(C^{Ac})_3(G^{iPrPac})_3A^{Pac}C^{Ac}$]-3′-CPG. The DMTr-ON oligomer was purified by RP-HPLC using gradient G4. Post detritylation the oligomer was desalted by RP-HPLC using gradient G5 followed by co-evaporation with H_2O (6 x 500 μL). Analytical RP-HPLC performed using gradient G6. t_R = 34.56 min. MALDI-TOF and ESI mass spec are available in **Table 1** and **2** respectively.

d(TSeTCCCGGGAA) (**ODN 5**).

ODN 5 was prepared from synthesis columns containing 5′-DMTr-d[$(C^{Ac})_3(G^{iPrP})_3(A^{Pac})_2$]-3′-CPG. The DMTr-ON oligomer was purified by RP-HPLC using gradient G4. Post detritylation the oligomer was desalted by RP-HPLC using gradient G5 followed by co-evaporation with H_2O (6 x 500 μL). Analytical RP-HPLC performed using gradient G6. t_R = 36.34 min. MALDI-TOF and ESI mass spec are available in **Table 1** and **2** respectively.

d(CGCGAASeTTCGCG) (**ODN 6**).

ODN 6 was prepared from iBu-dG SynBase CPG 500/110 S employing native (cyanoethyl-protected) monomer phosphoramidites (step 4a), or dimer phosphoramidite **5a** (step 4b) using the following synthesis cycle:

1. Wash (3 x 275 µL, MeCN)
2. Deblock (3 x 220 µL, 3% (w/v) TCA/DCM, 3 x 60 s)
3. Wash (3 x 275 µL, MeCN)

4a. Couple (80 µL 0.1M phosphoramidite, 100 µL 0.3 M BTT, 2 x 60 s)

4b. Couple (80 µL 0.1M dimer phosphoramidite **5a**, 100 µL 0.3 M BTT, 2 x 7.5 min)

5. Wash (3 x 275 µL, MeCN)
6. Cap (125 µL Cap Mix A (9:1 (v/v) THF/acetic anhydride), 125 µL Cap Mix B (10% (m/v) methylimidazole in 8:1 (v/v) THF/pyridine), 60 s)
7. Wash (3 x 275 µL, MeCN)
8. Oxidise (200 µL 0.02 M I_2 in 0.4% (v/v) pyridine/THF, 60 s)
9. Wash (3 x 275 µL, MeCN)
10. Wash (275 µL, 60 s 10% (v/v) pyridine/MeCN)

See trityl log for respective coupling efficiencies (**Figure S 1**). Post synthesis the sequence was treated with a 1 mL, 150 mM DTT solution (1:1 (v/v) EtOH/H_2O) for 1 h at ambient temperature in a screw cap vial. The CPG was filtered and washed with anhydrous MeCN (3 x 2 mL). This was followed by AMA deprotection (3 h)**.** The DMTr-ON oligomer was purified by RP-HPLC using gradient G7. Detritylation was effected using 80% (v/v) aqueous acetic acid (1 mL) and storage at ambient temperature for 1 hr. Desalting was achieved by RP-HPLC using gradient G5 followed by co-evaporation with H_2O (6 x 500 µL). Analytical RP-HPLC performed using gradient G6. Initial attempts resolved two peaks. The analytical chromatogram was repeated at 52 °C to resolve one peak. t_R = 29.60 min. ESI mass spec is available in **Table 2.**

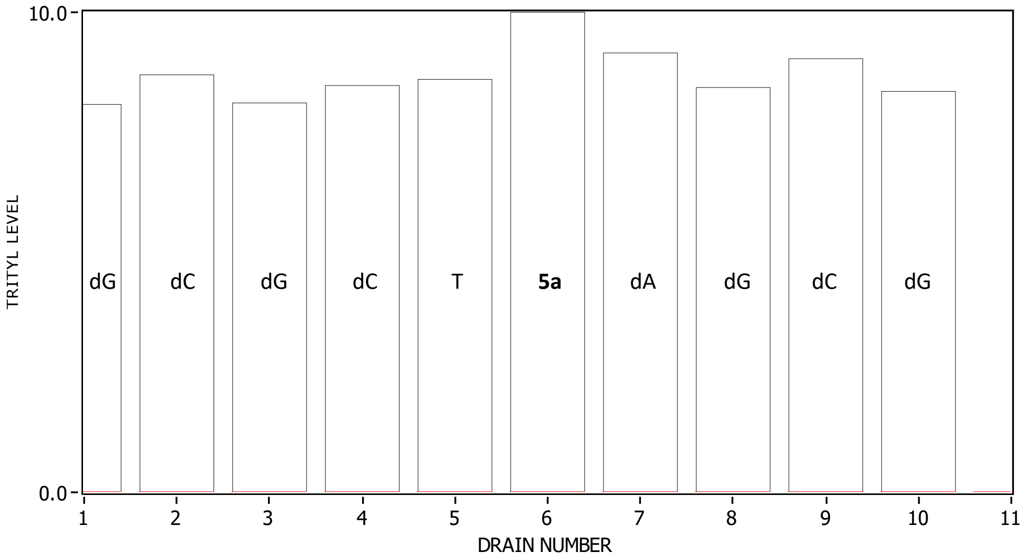

Figure S 1. Trityl log for the synthesis of **ODN 6**.

d(CCCSeT) (**ODN 7**).

To a stirred solution of 5′-(4,4′-dimethoxytrityl)-*N*[4]-acetyl-2′-deoxycytidine-3′-*O*-[cyanoethyl-(*N,N*-diisopropyl)]-phosphoramidite (Ac-dC-(CE) phosphoramidite) (500 mg, 0.65 mmol) in anhydrous MeCN (5 mL) at ambient temperature, under argon, was added 5-(ethylthio)-1*H*-tetrazole (337 mg, 2.6 mmol, 4.0 eq) in one portion. After 30 min, H_2O (122 µL) was added and stirring continued for a further 15 min. The reaction mixture was diluted with ethyl acetate (50 mL) and washed with satd. aqueous sodium carbonate (3 x 150 mL) and brine (60 mL). The first two sodium carbonate washes were combined and back extracted with ethyl acetate (2 x 50 mL). The organics were dried over sodium sulfate, filtered and the solvents removed in vacuo to yield a cream foamy solid of 5′-(4,4′-dimethoxytrityl)-*N*[4]-acetyl-2′-deoxygcytidine-3′-*O*-(cyanoethyl)-*H*-phosphonate (**3f**, 454 mg, 94%). This was stored under argon at -20 °C and used within 24 h. ^{31}P NMR (162 MHz, MeCN, D_2O external lock) δ_p = 8.11 and 8.06.

ODN 7 was prepared from selenocyanate-functionalised CPG **7** (45 mg, 1.75 µmol, 39 µmol g^{-1}) using the manual syringe method. After washing the CPG with MeCN (3 x 2 mL), a solution of 5′-*O*-(4,4′-dimethoxytrityl)-*N*[4]-acetyl-2′-deoxycytidine-3′-*O*-(2-cyanoethyl)-*H*-phosphonate **3f** (0.1 M, 1 mL) in anhydrous MeCN was passed through the DNA synthesis column for 1 h. Addition of native (cyanoethyl-protected) monomer phosphoramidites was performed by adopting the following synthesis cycle:

1. Wash (3 x 2 mL, MeCN)
2. Deblock (3 x 2 mL, 3% (w/v) TCA/DCM)
3. Wash (3 x 2 mL, MeCN)
4. Couple (200 µL 0.1 M Ac-dC-(CE) phosphoramidite + 300 µL, 0.3 M BTT, both in MeCN, 5 min)
5. Wash (3 x 2 mL, MeCN)
6. Cap (500 µL Cap Mix A (9:1 (v/v) THF/acetic anhydride), 500 µL Cap Mix B (10% (m/v) methylimidazole in 8:1 (v/v) THF/pyridine), 45 s (only after first phosphoramidite coupling))
7. Wash (3 x 2 mL, MeCN)
8. Oxidise (500 µL 0.02 M I_2 in 0.4% (v/v) pyridine/THF), 45 s
9. Wash (3 x 2 mL, MeCN)
10. Wash (1 x 1 mL, 10% (v/v) pyridine/MeCN)
11. Wash (3 x 2 mL, MeCN)

Post synthesis processing was carried out as described for **ODN 1**, but, omitting the demethylation step. The DMTr-ON oligomer was purified by RP-HPLC using gradient G1. Post detritylation the oligomer was desalted by RP-HPLC using gradient G2 followed by co-evaporation with H_2O (6 x 500 µL). Analytical RP-HPLC performed using gradient G3. t_R = 31.30 min. ^{31}P NMR (243 MHz, 40% (v/v) D_2O / H_2O) δ_p = 11.20 ($^1J_{PSe}$ = 396 Hz), -0.96 and -0.99. ESI mass spec is available in **Table 2**

Oligodeoxynucleotide Characterisation:

Table 1. MALDI-TOF

Oligodeoxynucleotide	Calculated (^{80}Se)	Found
**ODN 2**	3091.480	3091.778
**ODN 3**	3092.475	3092.794
**ODN 4**	3092.475	3092.756
**ODN 5**	3091.480	3091.808

Table 2. ESI

Oligodeoxynucleotide	Calculated (^{80}Se)	Found
**ODN 1**	1585.112	1585.070
**ODN 2**	3090.472	3090.370
**ODN 3**	3091.468	3091.365
**ODN 4**	3091.468	3091.351
**ODN 5**	3090.472	3090.360
**ODN 6**	3709.573	3708.450
**ODN 7**	1172.143	1172.144

Enzyme Digest

Solutions containing 2 OD^{260nm} units of either **ODN 5** (d(TSeTCCCGGGAA)) or its native congener (d(TTCCCGGGAA)) in 100 mM Tris HCl buffer (pH 8), and 100 mM NaCl were heated at 95 °C for 3 min and snap cooled on ice. To these solutions were then added 4.2 µL $MgCl_2$ (1 M stock soln, final concentration = 14 mM) followed by 10 µL of a freshly prepared solution of snake venom (Sigma - V7000, 1 mg/mL aqueous stock soln, final concentration = 33 µg/mL) (total end volume of digest = 300 µL). After vortex mixing, digestions were incubated at 37 °C for 8 h. From each digest, an aliquot (150 µL) was removed and quenched following heating at 95 °C for 3 min. The reaction mixture was then analysed by RP-HPLC using gradient G8. (**Figures S2-4**).

S 2.

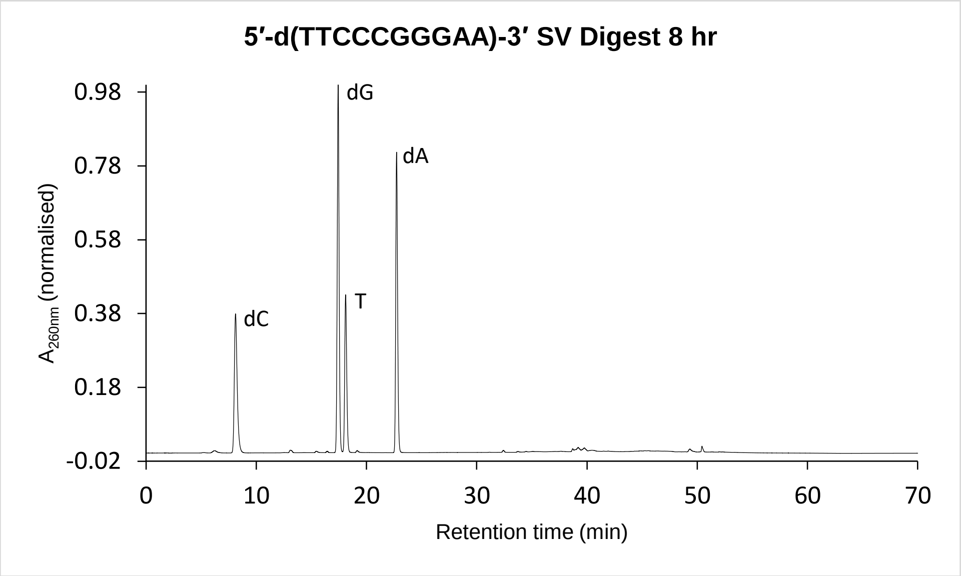

5′-d(TTCCCGGGAA)-3′ SV Digest 8 hr
0.98
0.78
0.58
0.38
0.18
-0.02
A260nm (normalised)
dG
dA
T
dC
0
10
20
30
40
50
60
70
Retention time (min)

S 3.

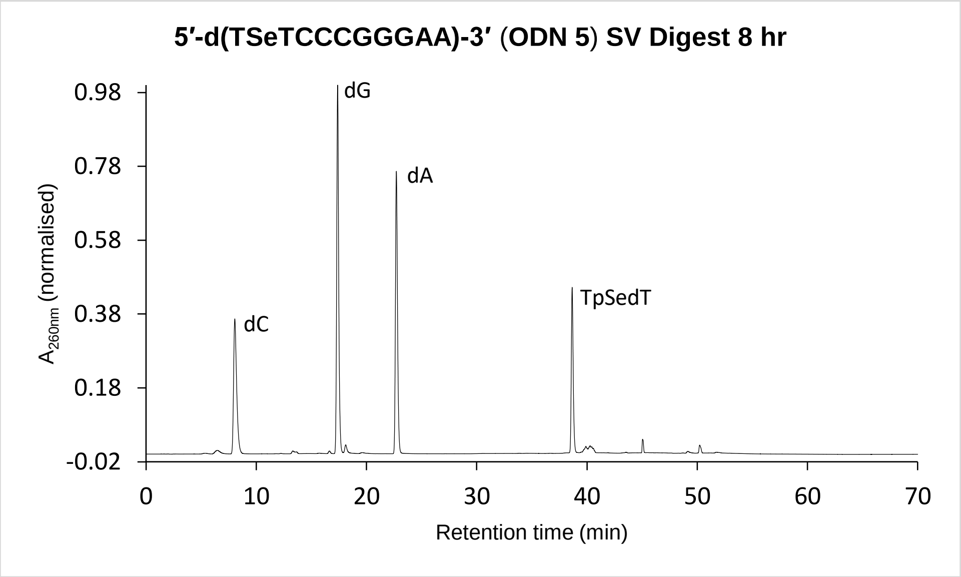

5′-d(TSeTCCCGGGAA)-3′ (ODN 5) SV Digest 8 hr
0.98
0.78
0.58
0.38
0.18
-0.02
A260nm (normalised)
dG
dA
TpSedT
dC
0
10
20
30
40
50
60
70
Retention time (min)

S 4.

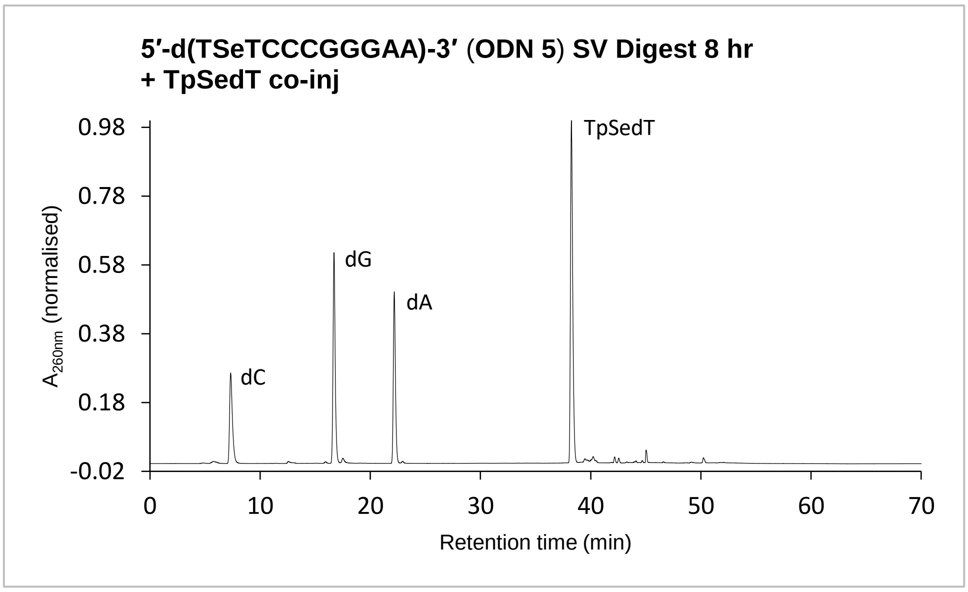

5′-d(TSeTCCCGGGAA)-3′ (ODN 5) SV Digest 8 hr
+ TpSedT co-inj
0.98
0.78
0.58
0.38
0.18
-0.02
A260nm (normalised)
TpSedT
dG
dA
dC
0
10
20
30
40
50
60
70
Retention time (min)

UV Thermal Denaturation Studies

ODN 2-6 along with their native analogues were diluted in sodium phosphate buffer (10 mM, pH 7) and NaCl (100 mM) to a final concentration of 10 µM ssDNA and a final volume of 600 µL. The sequences were annealed by heating to 90 °C for 3 min and allowed to slowly cool to room temperature. UV spectra were recorded on an Agilent technologies Cary 100/300 UV-vis spectrophotometer equipped with a 6 x 6 multicell block Peltier cuvette holder and a thermoelectric temperature controller. For the measurement the instrument was programmed to heat from 10-70 °C (**ODN 2-5**) or 10-90 °C (**ODN 6**) with a temperature change rate of 0.5 °C per minute in a 1 cm pathlength cuvette. UV absorbance was monitored at 260 nm and recorded at 0.5-min intervals. Melting temperatures (T_m) were determined by the maximum of the first derivative averaged over two runs (**ODN 2-5**). T_m for **ODN 6** was determined as the midpoint of the normalised absorbance and averaged (T_m) over two runs.

CD Spectroscopy Studies

ODN 2-6 along with their native analogues were diluted in sodium phosphate buffer (10 mM, pH 7) and NaCl (100 mM) to a final concentration of 10 µM ssDNA and final volume of 600 µL. The sequences were annealed by heating to 90 °C and slowly cooling to room temperature. CD spectra were recorded at 10 °C between 220 and 350 nm with 1 nm wavelength increments in a 1 cm pathlength cuvette on a Chirascan spectrophotometer at Diamond Light Source.

S 5.

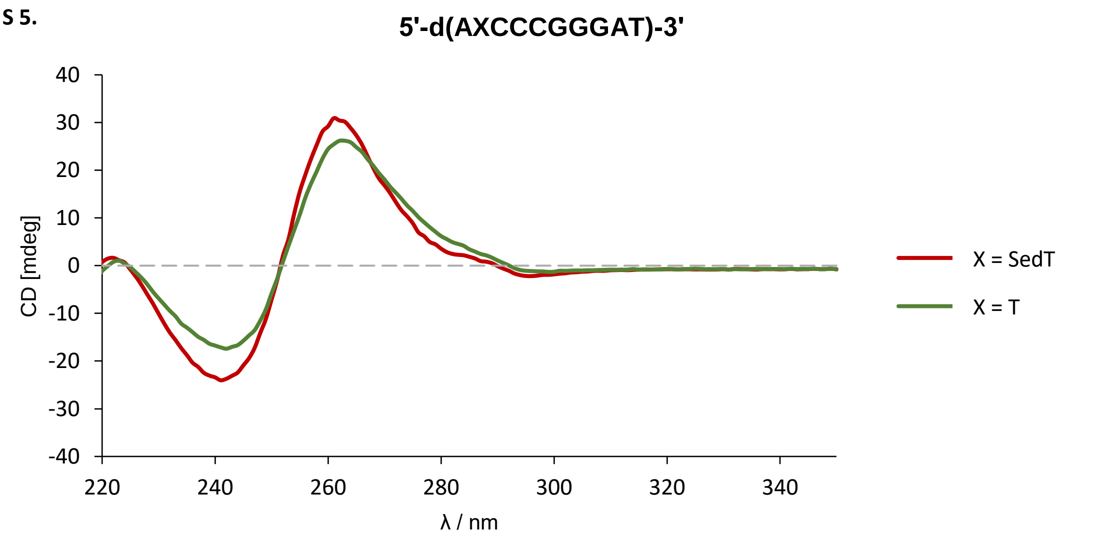

S 6.

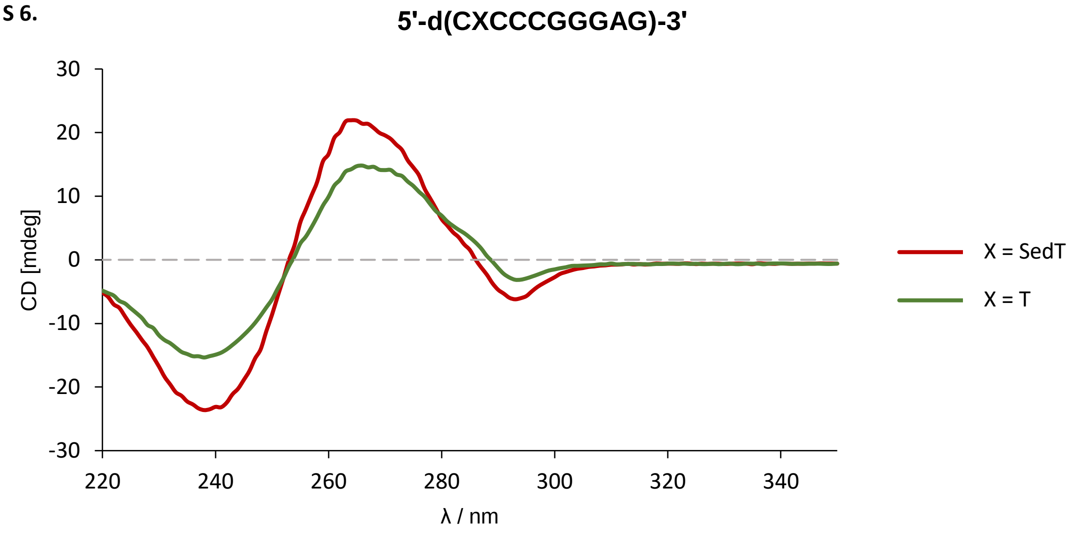

5'-d(CXCCCGGGAG)-3'
CD [mdeg]
30
20
10
0
-10
-20
-30
220
240
260
280
300
320
340
λ / nm
X = SedT
X = T

S 7.

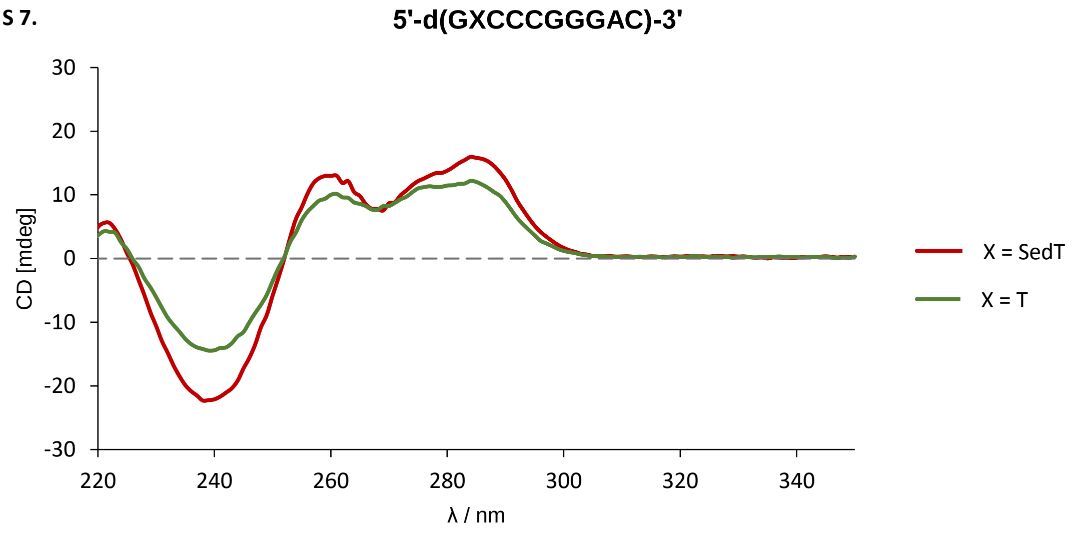

5'-d(GXCCCGGGAC)-3'
CD [mdeg]
30
20
10
0
-10
-20
-30
220
240
260
280
300
320
340
λ / nm
X = SedT
X = T

S 8.

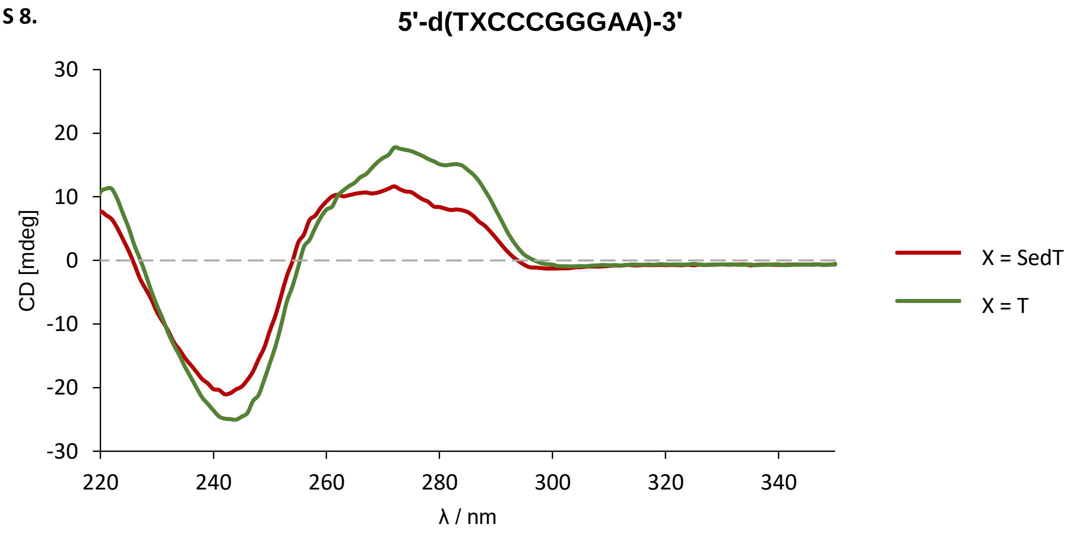

5'-d(TXCCCGGGAA)-3'
CD [mdeg]
30
20
10
0
-10
-20
-30
220
240
260
280
300
320
340
λ / nm
X = SedT
X = T

S 9.

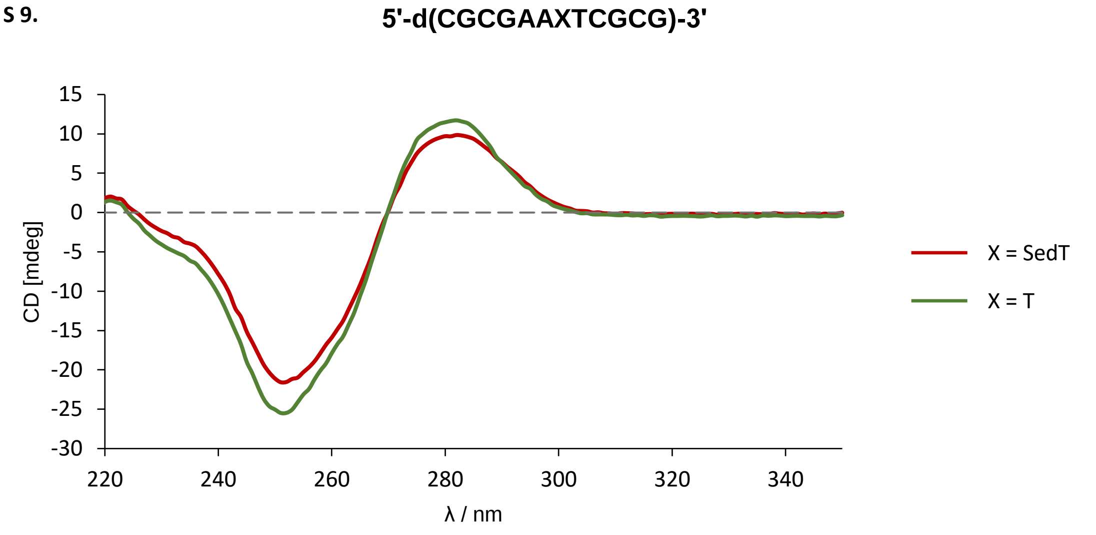

Crystallisation, X-ray data processing and refinement

The crystallisation solution contained 1 µL of 2 mM oligonucleotide and 6 µL of a solution containing 10% (v/v) 2-methyl-2,4-pentanediol, 40 mM Na-cacodylate pH 6, 12 mM spermine *tetra*-HCl, 80 mM NaCl and 20 mM $BaCl_2$. This was equilibrated against 100 µL of 35% (v/v) 2-methyl-2,4-pentanediol. Crystals grew in approximately 1-2 weeks and were grown using the sitting-drop method at 291 K.

Data were collected on beamline I03 at Diamond Light Source. 3600 images were collected, using a 0.1° oscillation and 0.05 s exposure time. The data were collected just above the Se-edge, to facilitate anomalous phasing, using a wavelength of 0.9596 Å. The data was processed using xia2[7] with DIALS[8] and the structure solved using Phenix.autosol[9], with the anomalous signal of Se. It was subsequently found that the crystal suffered radiation damage, so the dataset was cut to use the first 600 images. The model was built using Coot[10] and refined with Phenix.refine, to give a final R_{factor} of 0.18 and R_{free} of 0.23. The structure was initially refined in spacegroup *P* $6_1$22 but, following analysis with Phenix.xtriage, it became apparent that the crystal was a near-perfect twin with true symmetry in a lower space group. This was assigned as *P* $3_1$21 following attempted model building and refinement in all possible lower symmetry spacegroups. The twin law applied during refinement was -h,-k,l and the crystal possessed merohedral twinning with a twin fraction of 0.47. Full data processing and refinement statistics can be found in **Table 3**.

The asymmetric unit of the structure contains two DNA strands (in an A-DNA double helix), two Na^+, one Cl^-, two Ba^{2+} bifurcated to guanine bases, 64 water molecules and one spermine molecule bound between the phosphate backbones (**Figure S 10.**)

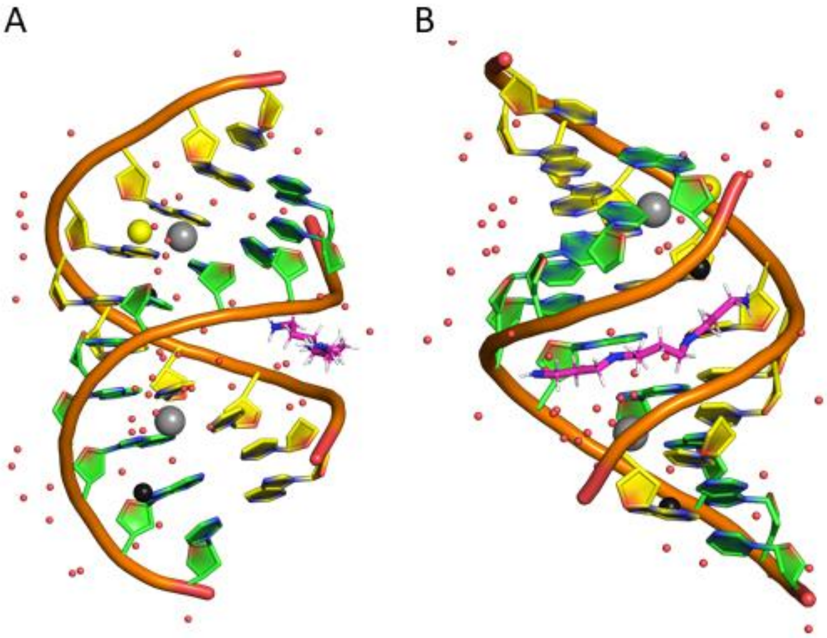

Figure S 10. Two views of the asymmetric unit (A) along the side of the phosphate backbone and (B) into the major groove. The two strands are coloured with carbon atoms in either yellow or green, to aid clarity. The carbon atoms of the spermine molecule are coloured magenta. All other atoms are coloured according to type with Na^{+} black, Ba^{2+} silver, Cl^{-} yellow, nitrogen blue, hydrogen white, oxygen red and the phosphate backbone is drawn as an orange strand. Water molecules are drawn as red spheres.

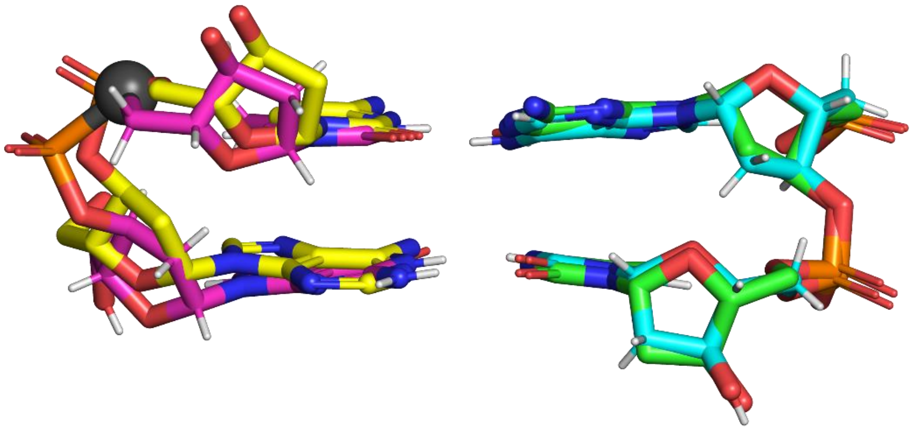

Figure S 11: A least-squares superimposition of the phosphoroselenoate-modified dinucleotide step in the structure reported here (**6S7D, carbon atoms in yellow and green**) with the analogous step from a previously reported structure[11] with an RMSD of 0.37 Å (carbon atoms in magenta and cyan). Other atoms are coloured according to type with oxygen in red, phosphorus in orange, nitrogen in blue, carbon in white and selenium as a grey sphere.

Table 3: Crystallographic data processing and refinement statistics

**Data processing**	
Space group	$P3_121$
Resolution, Å	25.57-1.45 (1.47-1.45)
R_{merge}	0.050 (0.556)
R_{meas}	0.060 (0.674)
R_{pim}	0.032 (0.375)
Total number of observations	37183 (1630)
Total number of unique observations	12548 (625)
I/σI	12.5 (1.3)
$CC_{1/2}$	0.998 (0.741)
Completeness, %	98.4 (98.7)
Multiplicity	3.0 (2.9)
**Refinement**	
No. Reflections	12530
R_{work}/R_{free}	0.18/0.23
**No. Atoms**	
DNA	630
Ligands	45
Water	64
**Average B-factors**	
DNA	25.84
Ligands	39.96
Water	29.77
**RMSD**	
Bond Lengths, Å	0.009
Bond Angles, °	0.936
**PDB ID**	**6S7D**

References

1 R. L. Puntel, D. S. Avila, D. H. Roos and S. Pinton, *Curr. Org. Chem.*, 2016, **20**, 198–210.

2 M. Vinceti, P. Ballotari, C. Steinmaus, C. Malagoli, F. Luberto, M. Malavolti and P. G. Rossi, *Environ. Res.*, 2016, **150**, 348–356.

3 D. G. Barceloux and D. Barceloux, *J. Toxicol. Clin. Toxicol.*, 1999, **37**, 145–172.

4 K. Verlinden, H. Buhl, W. Frank and C. Ganter, *Eur. J. Inorg. Chem.*, 2015, **2015**, 2416–2425.

5 O. Eguaogie, P. F. Conlon, F. Ravalico, J. S. T. Sweet, T. B. Elder, L. P. Conway, M. E. Lennon, D. R. W. Hodgson and J. S. Vyle, *Beilstein J. Org. Chem.*, 2017, **13**, 87–92.

6 J. S. Vyle, N. H. Williams and J. A. Grasby, *Tetrahedron Lett.*, 1998, **39**, 7975–7978.

7 G. Winter, *J. Appl. Crystallogr.*, 2010, **43**, 186–190.

8 D. G. Waterman, G. Winter, J. M. Parkhurst, L. Fuentes-Montero, J. Hattne, A. S. Brewster, N. K. Sauter and G. Evans, *CCP4 Newsl. protein Crystallogr.*, 2013, **49**, 16–19.

9 P. D. Adams, P. V. Afonine, G. Bunkóczi, V. B. Chen, I. W. Davis, N. Echols, J. J. Headd, L. W. Hung, G. J. Kapral, R. W. Grosse-Kunstleve, A. J. McCoy, N. W. Moriarty, R. Oeffner, R. J. Read, D. C. Richardson, J. S. Richardson, T. C. Terwilliger and P. H. Zwart, *Acta Crystallogr. Sect. D Biol. Crystallogr.*, 2010, **66**, 213–221.

10 P. Emsley, B. Lohkamp, W. G. Scott and K. Cowtan, *Acta Crystallogr. Sect. D Biol. Crystallogr.*, 2010, **66**, 486–501.

11 C. A. Frederick, G. J. Quigley, M. -K Teng, M. Coll, G. A. van der Marel, J. H. van Boom, A. Rich and A. H.-J. Wang, *Eur. J. Biochem.*, 1989, **181**, 295–307.

Section C. Supplementary Figures

S 12. DMTrdA^{PAc}*p*SedT (4a). ^{1}H NMR

(600 MHz, DMSO-d_6)

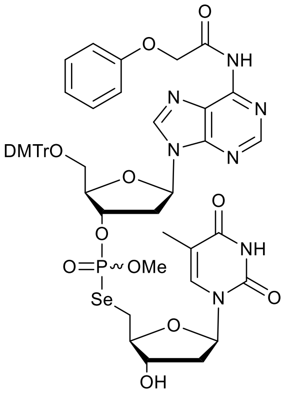

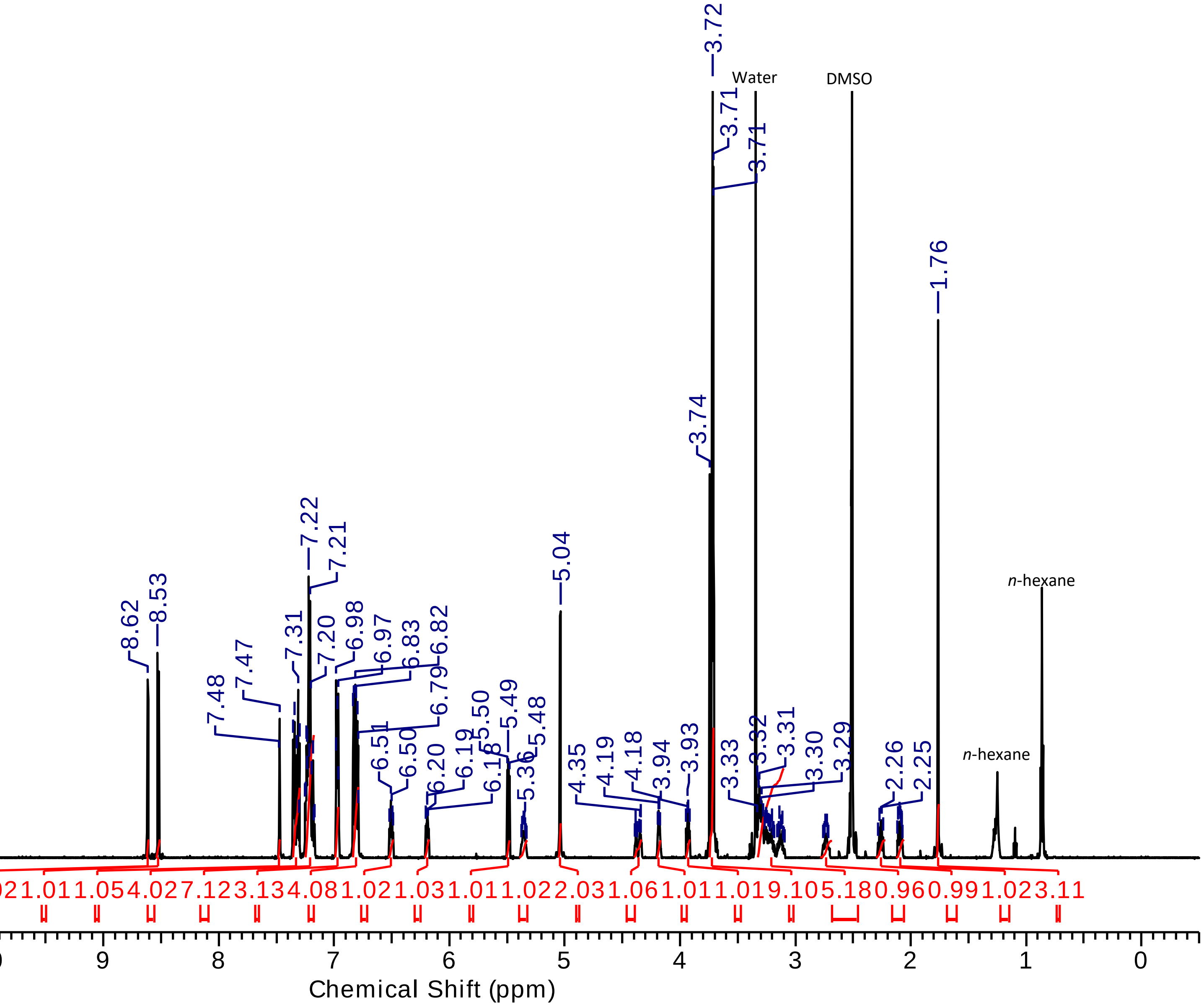

S 13. DMTrdA[PAc]*p*SedT (4a). ¹³C NMR

(150 MHz, DMSO-d_6)

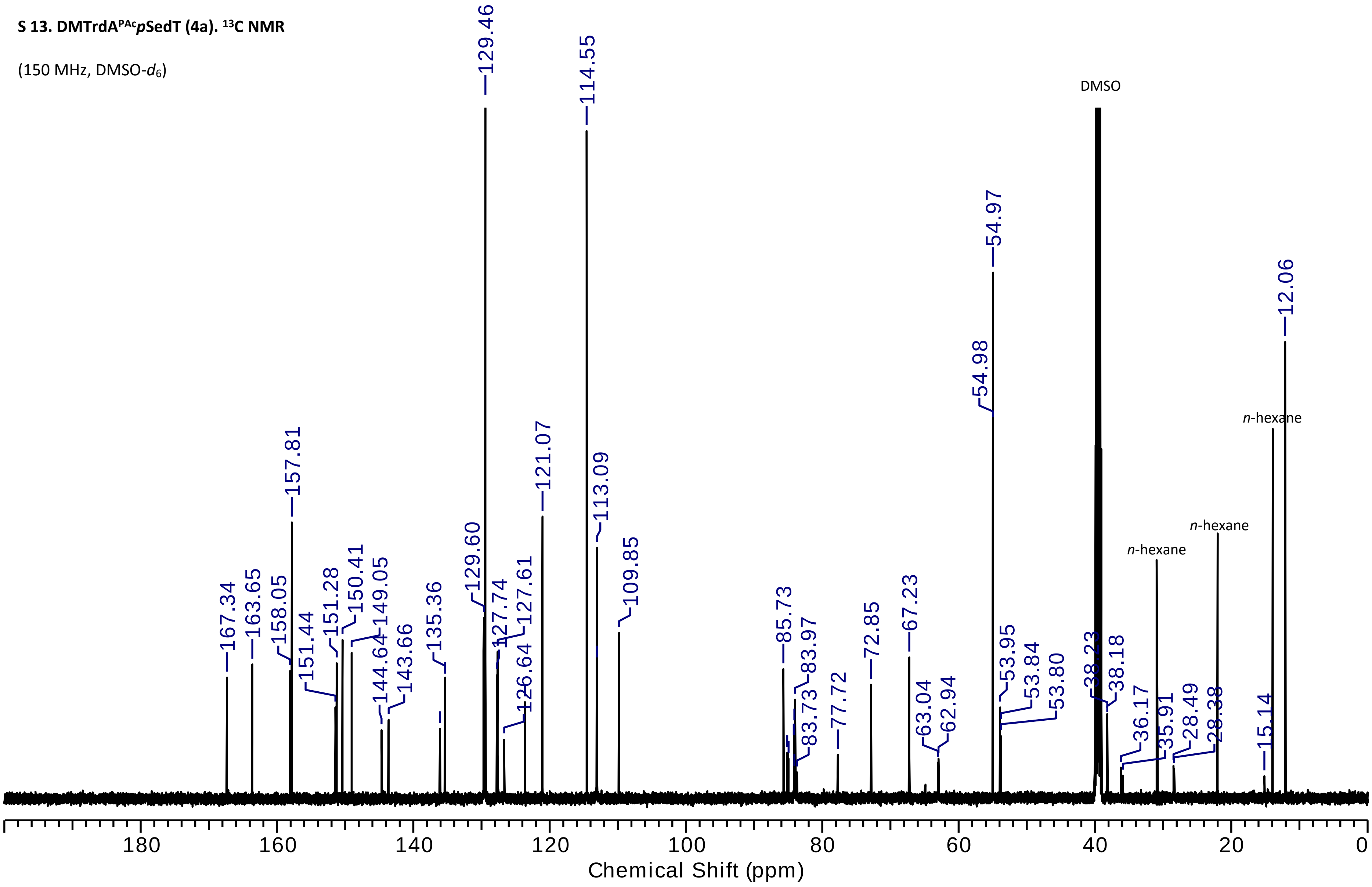

S 14. DMTrdA^{PAc}*p*SedT (4a). $^{31}P\{^1H\}$ NMR

(162 MHz, DCM, D_2O external lock)

With expansion to show P-Se couplings

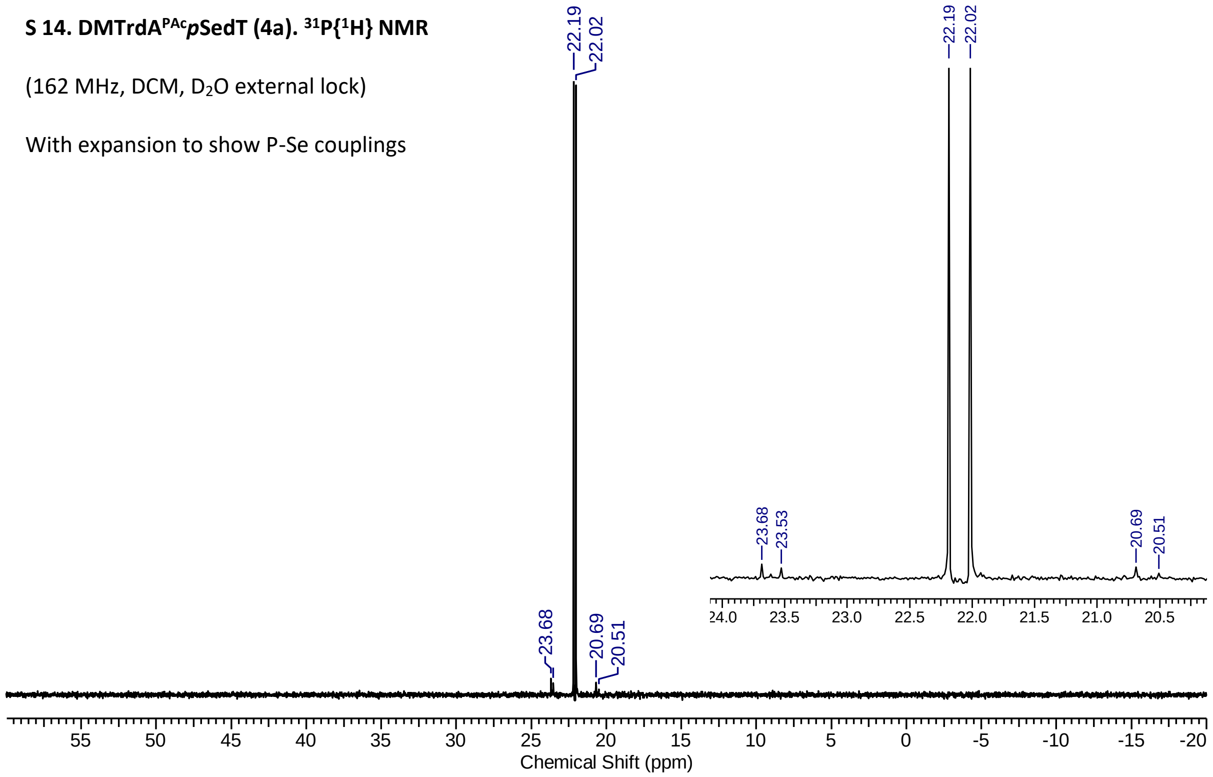

S 15. DMTrdA^{PAc}*p*SedT (4a). $^{77}Se\{^1H\}$ NMR

(114 MHz, DMSO-d_6 with external 0.25M KSeCN / D_2O Insert)

With expansion to show Se-P couplings

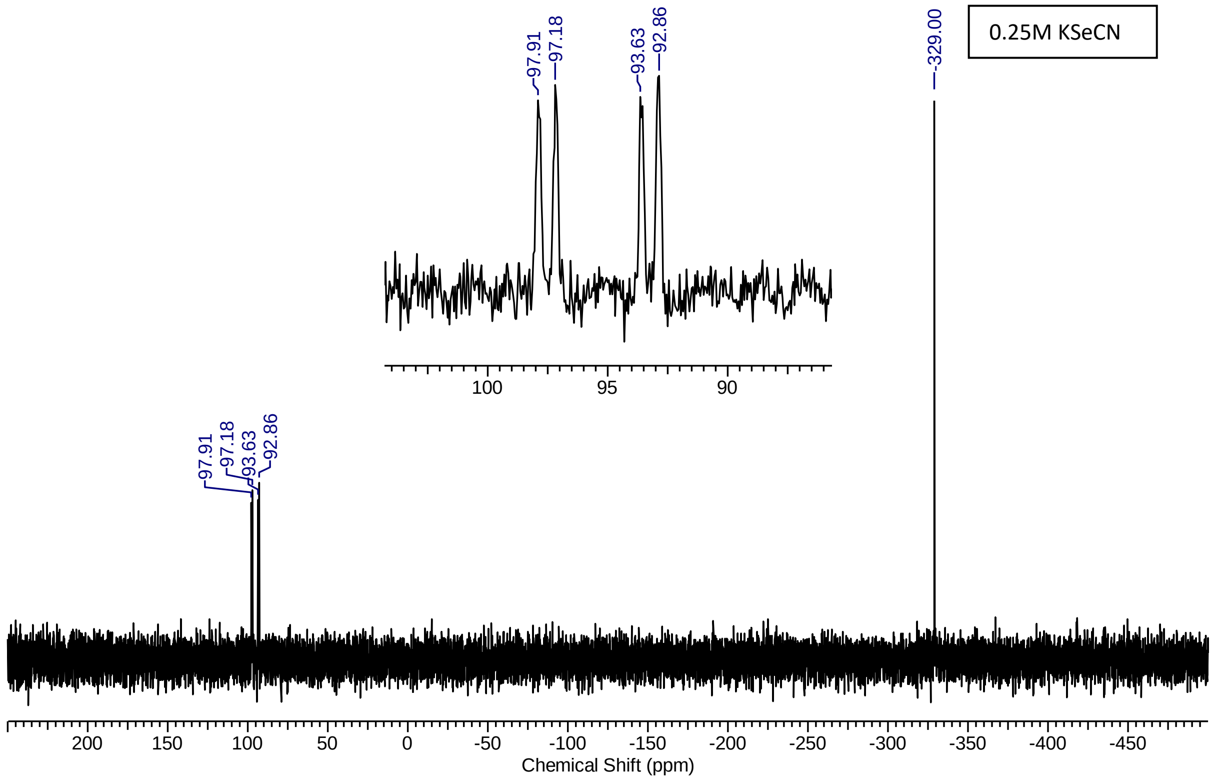

S 16. DMTrdA^{PAc}*p*SedT (4a). ES Mass spec

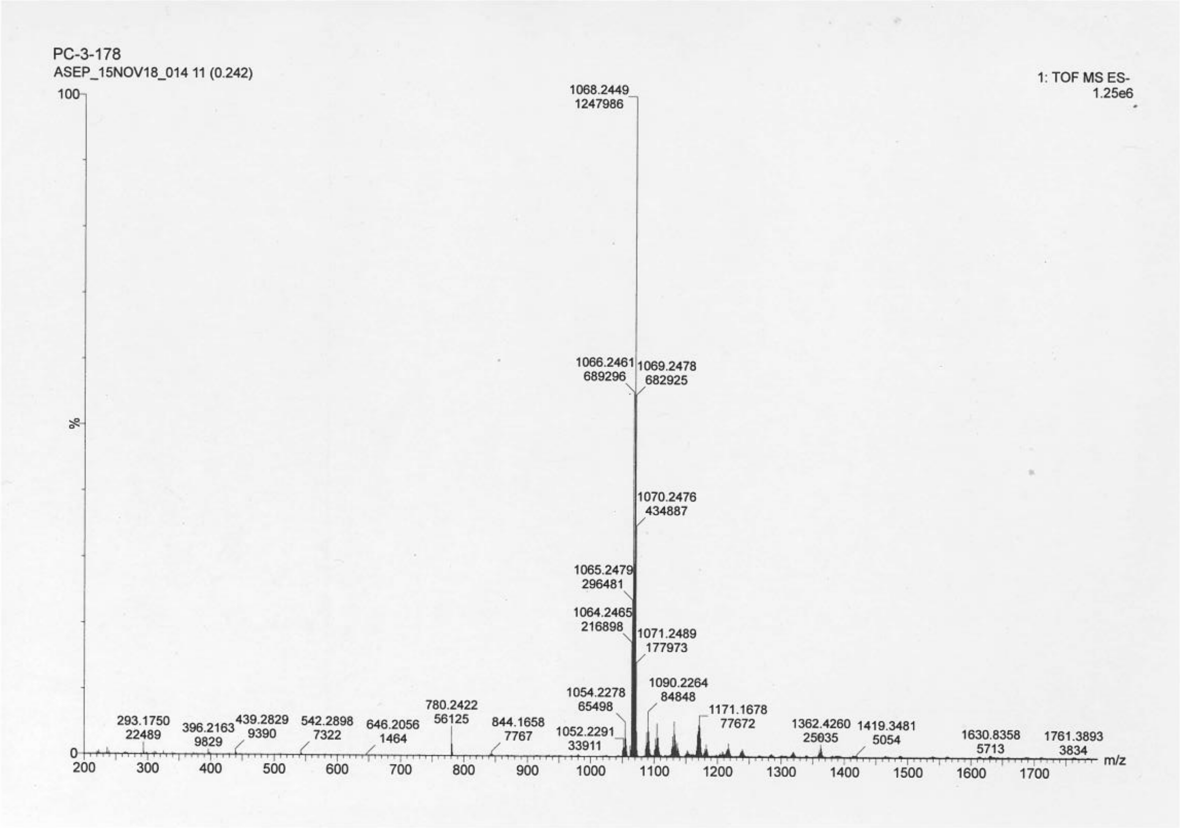

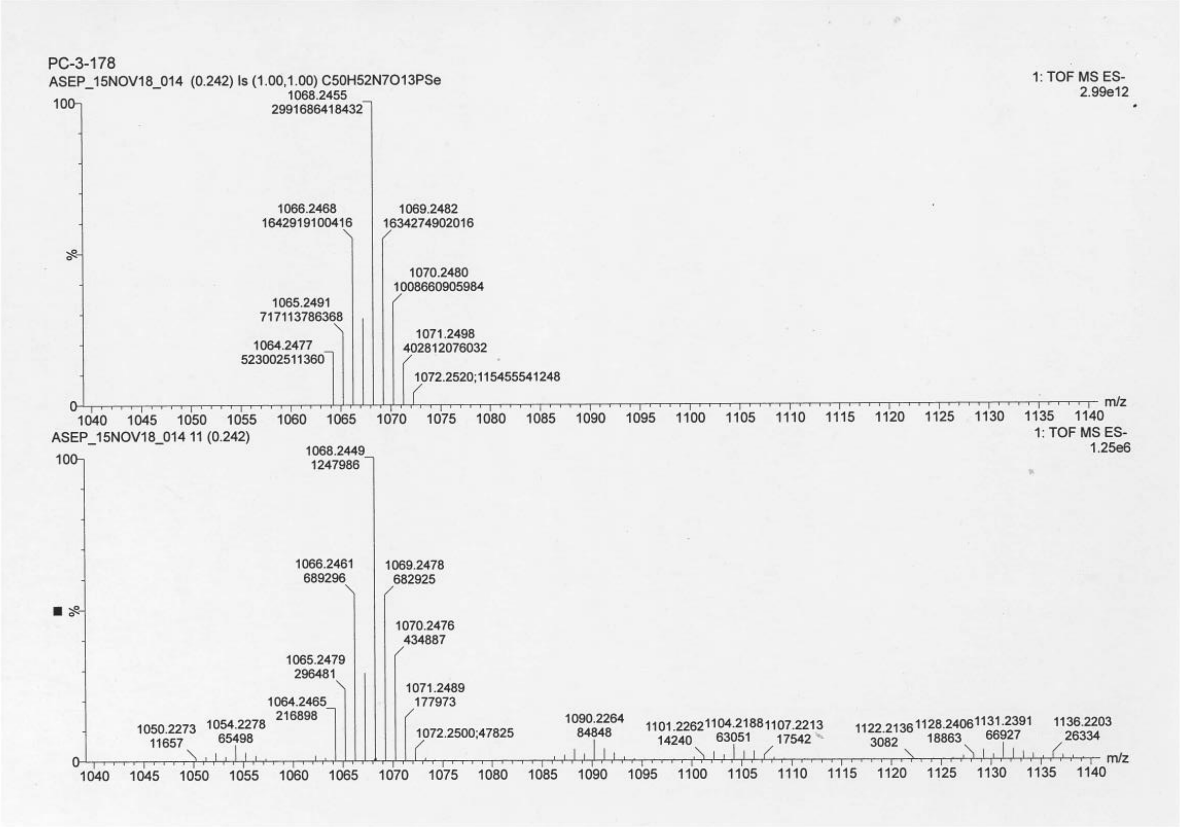

S 17. DMTrdC^{Ac}*p*SedT (4b). ^{1}H NMR

(600 MHz, DMSO-d_6)

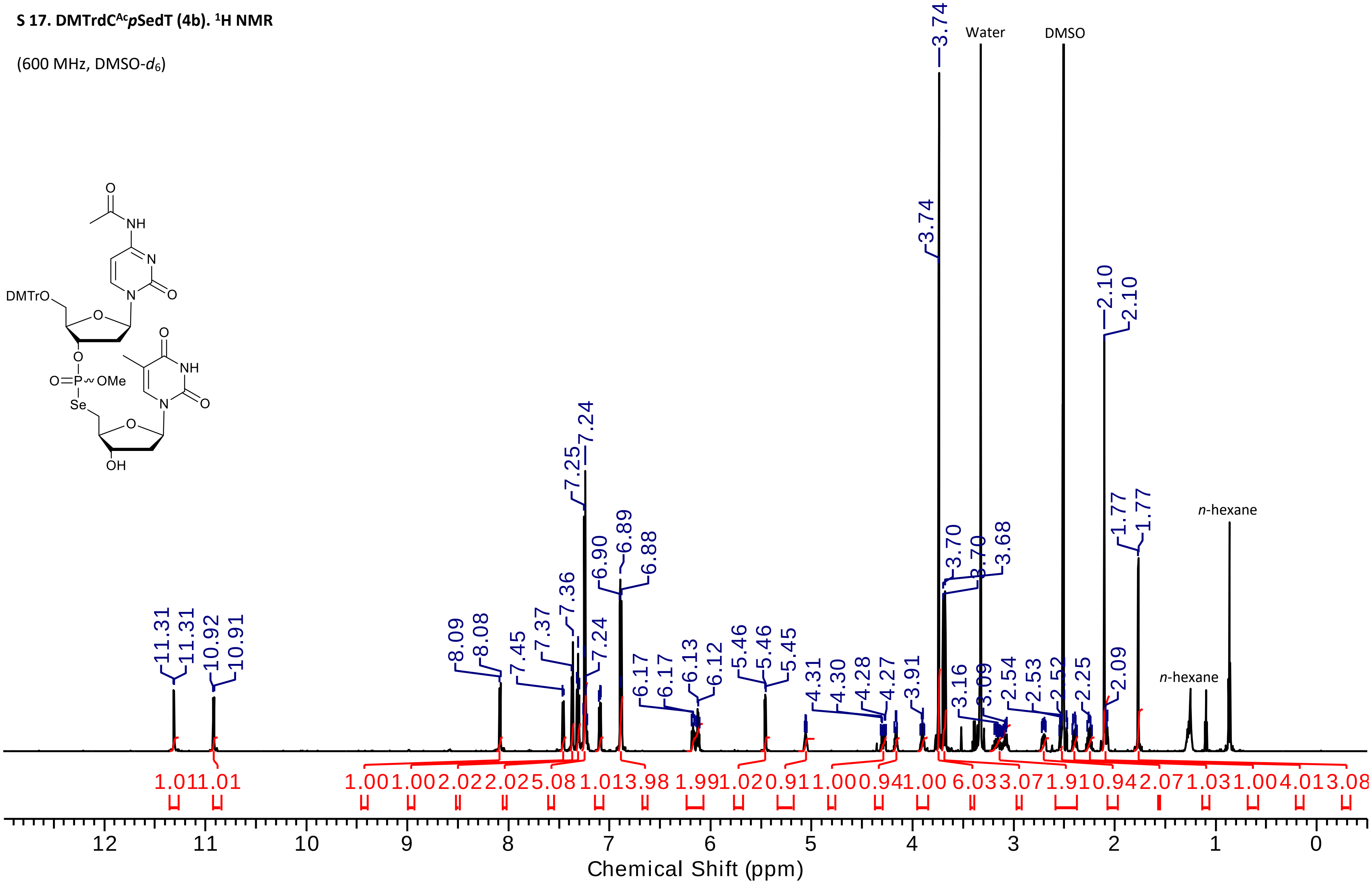

S 18. DMTrdC^{Ac}*p*SedT (4b). ^{13}C NMR

(150 MHz, DMSO-d_6)

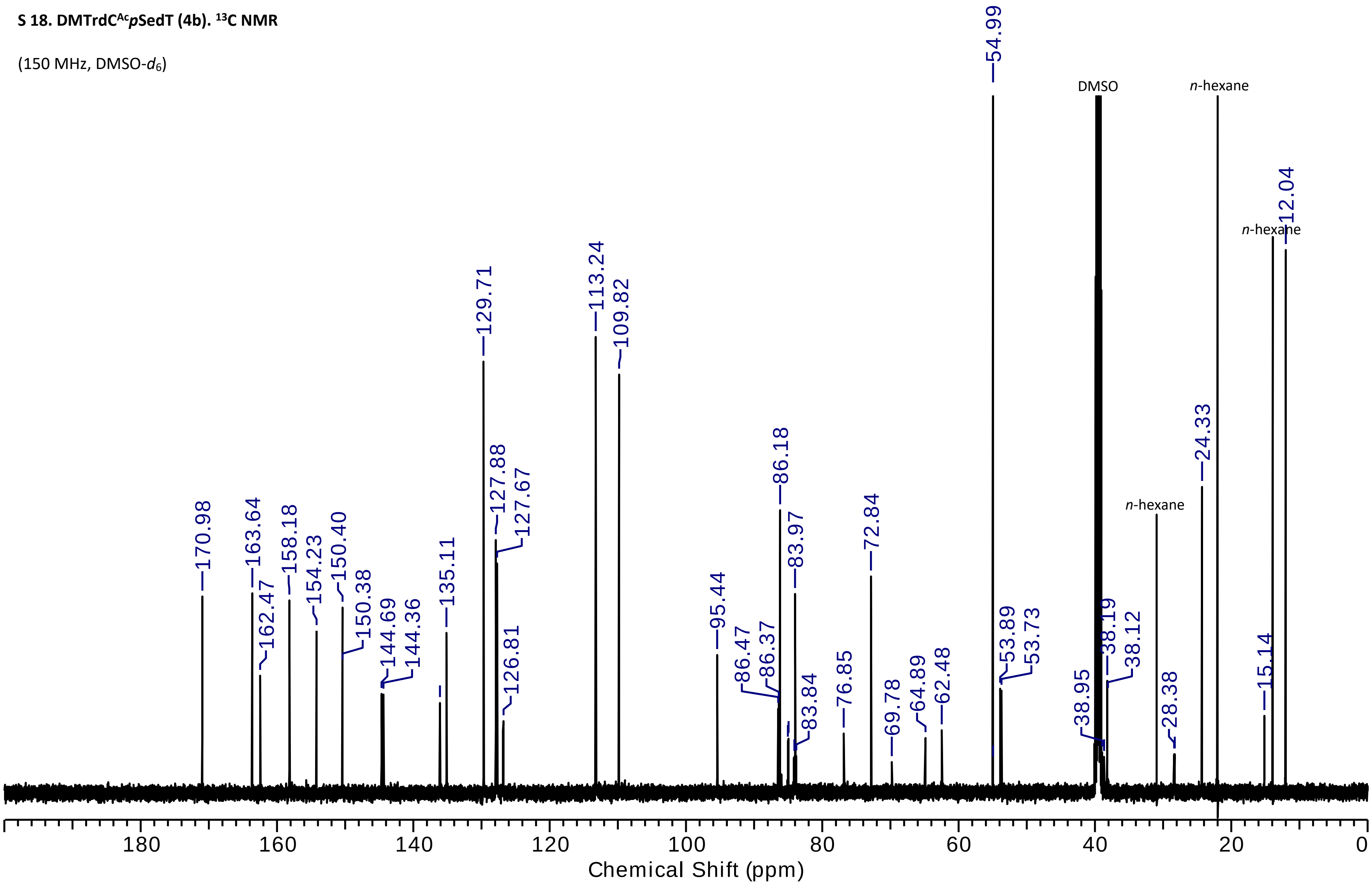

S 19. DMTrdC^{Ac}*p*SedT (4b). $^{31}P\{^{1}H\}$ NMR

(243 MHz, DCM, D_2O external lock)

With expansion to show P-Se couplings

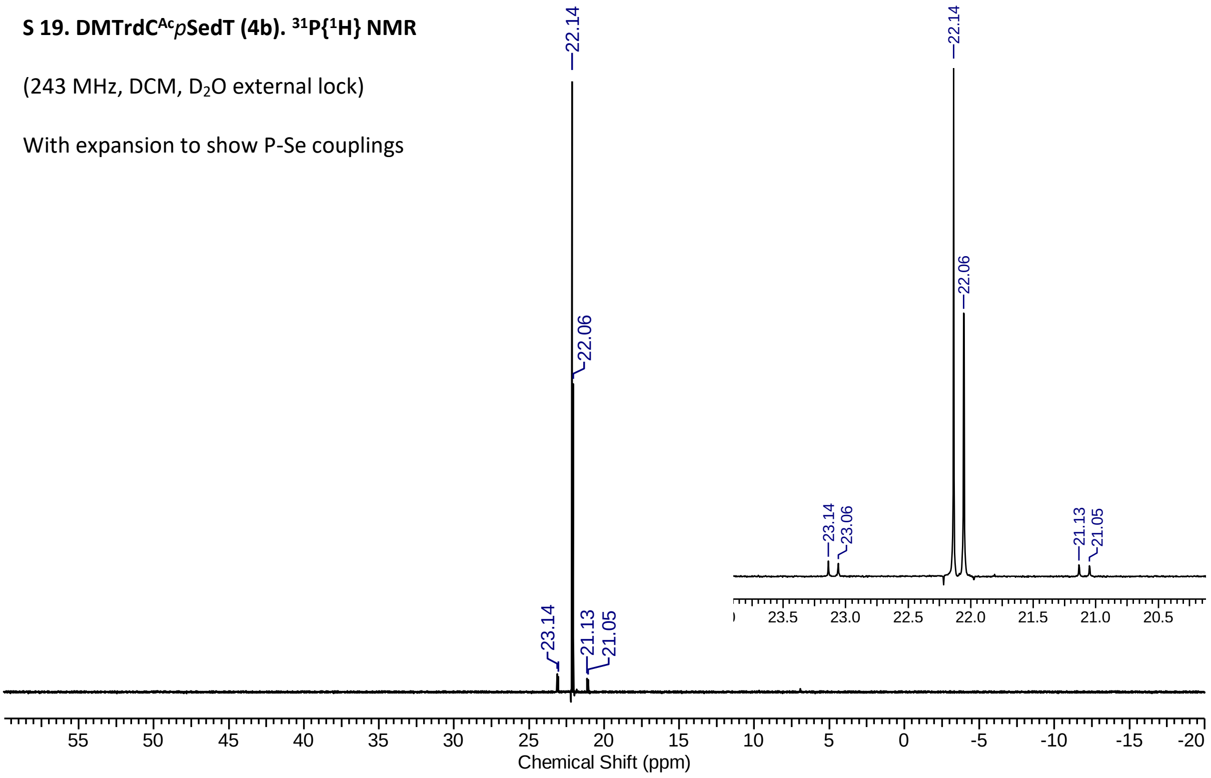

S 20. DMTrdC^{Ac}*p*SedT (4b). $^{77}Se\{^{1}H\}$ NMR

(114 MHz, DMSO-d_6 with external 0.25M KSeCN / D_2O Insert)

With expansion to show Se-P couplings

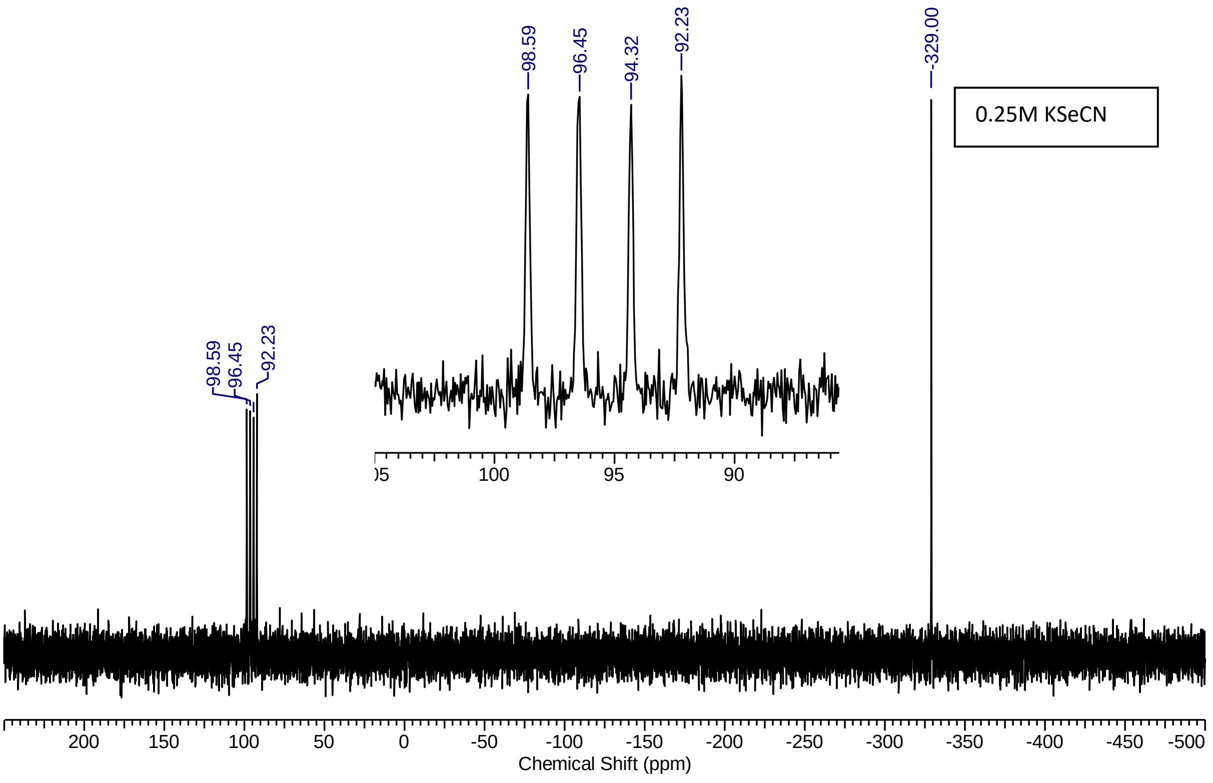

S 21. DMTrdC^{Ac}*p*SedT (4b). ES Mass spec

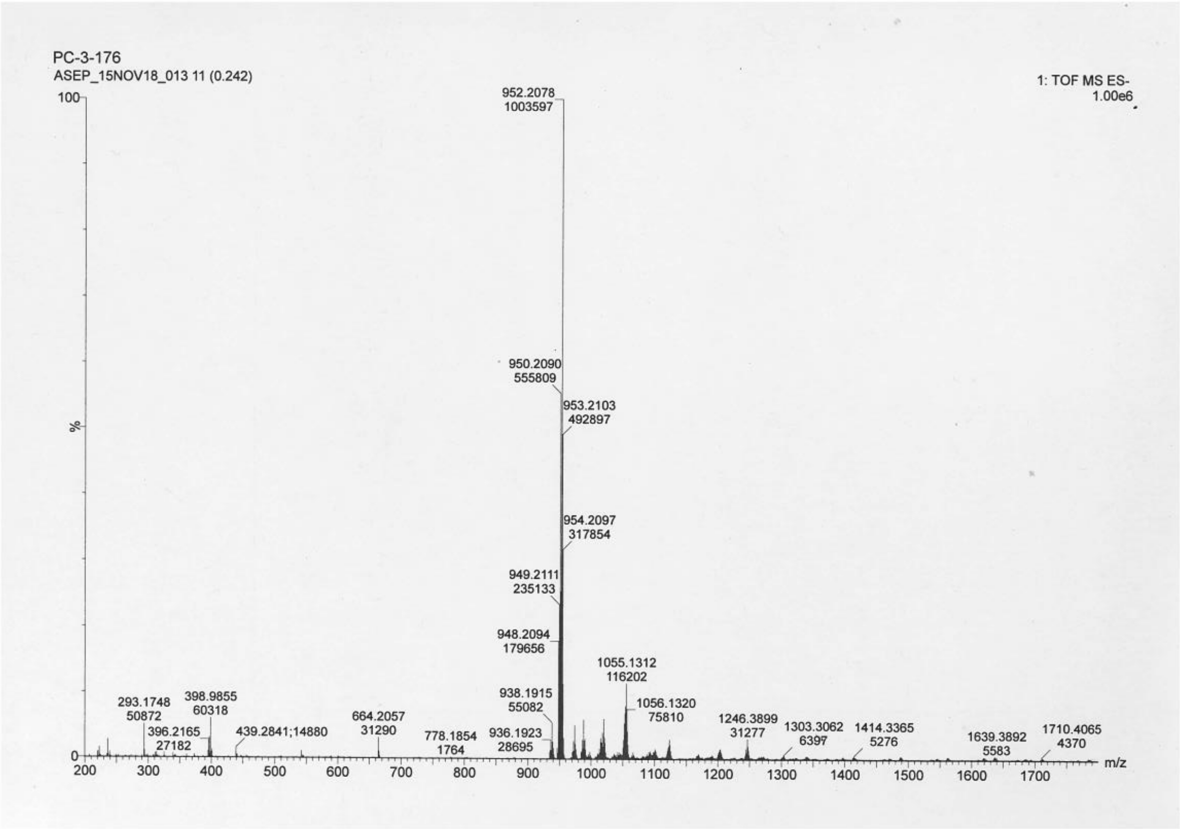

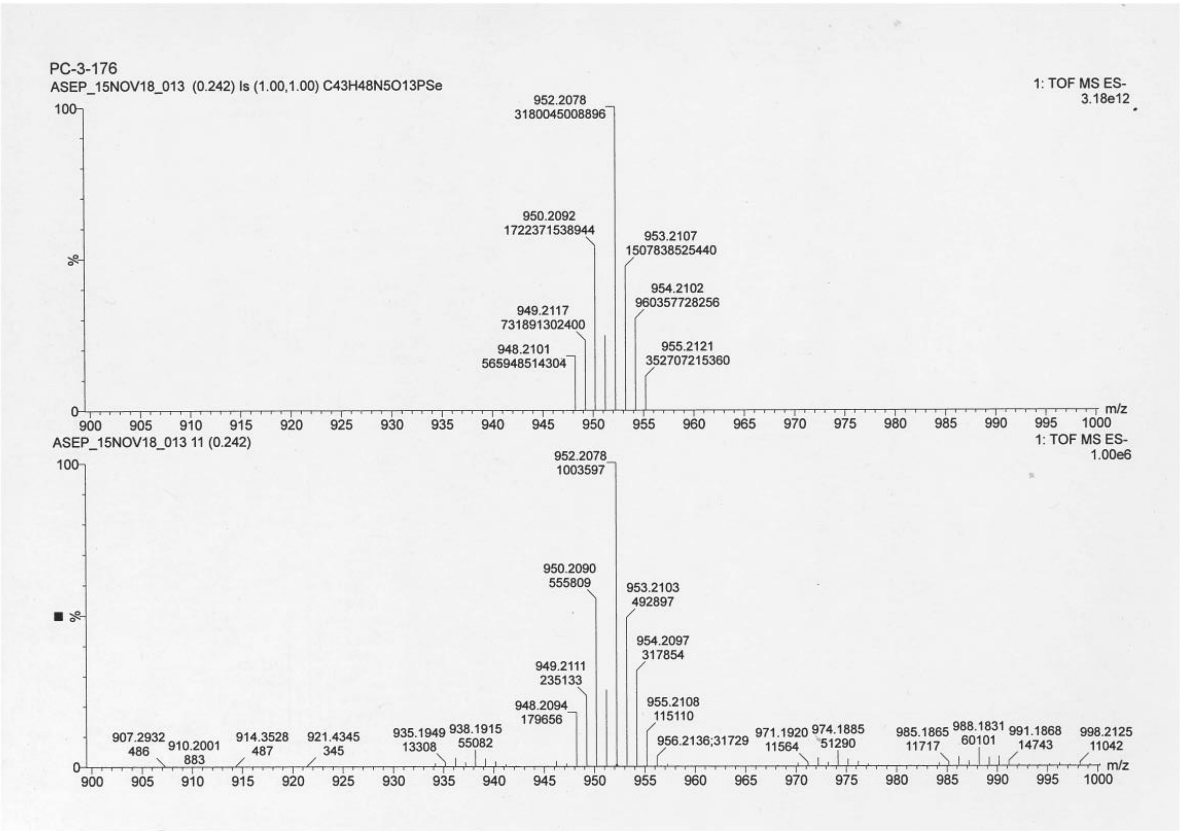

S 22. MMTrdG^{iBu}*p*SedT (4c). ^{1}H NMR

(600 MHz, DMSO-d_6)

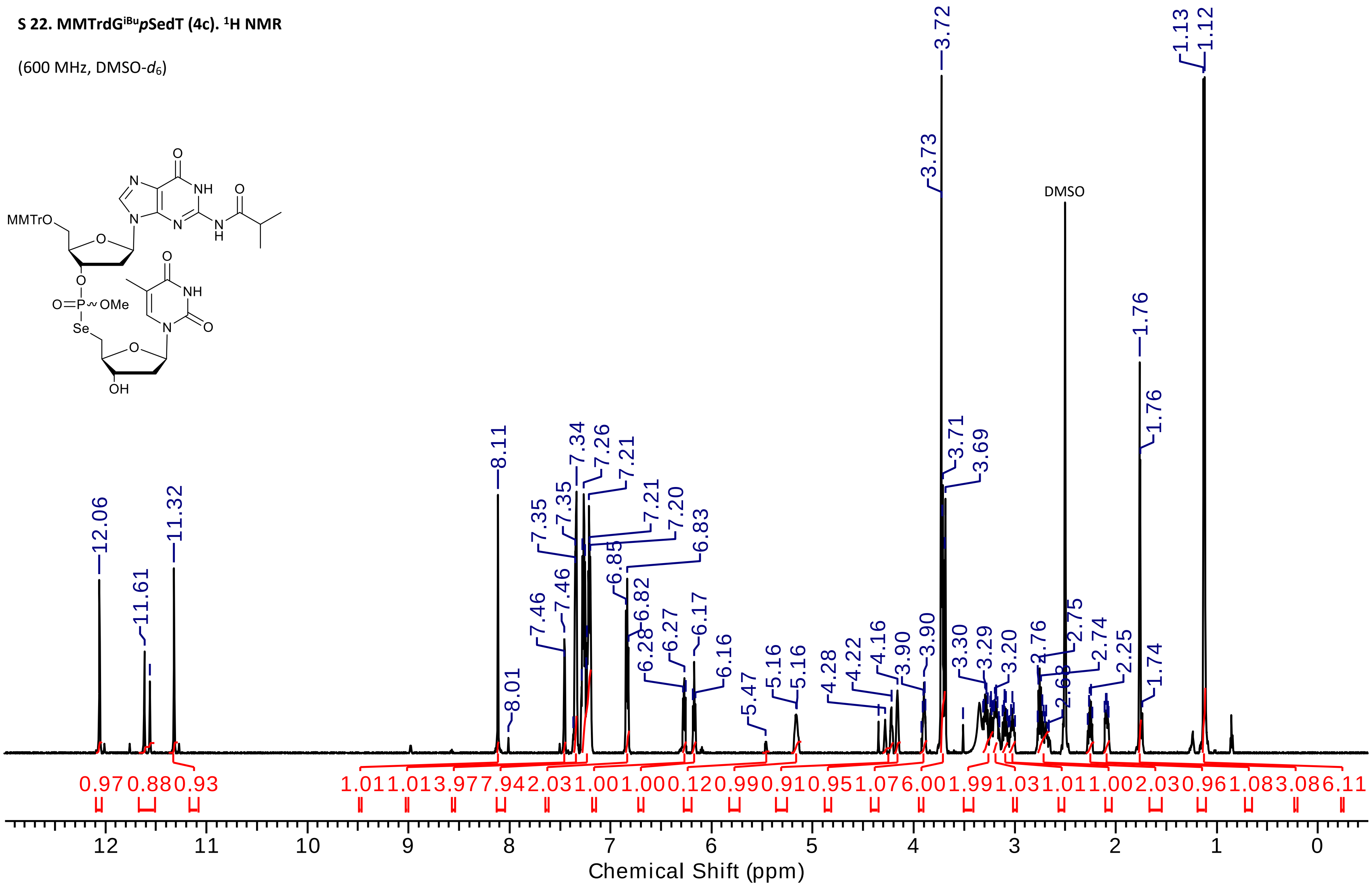

S 23. MMTrdG^{iBu}*p*SedT (4c). ^{13}C NMR

(150 MHz, DMSO-d_6)

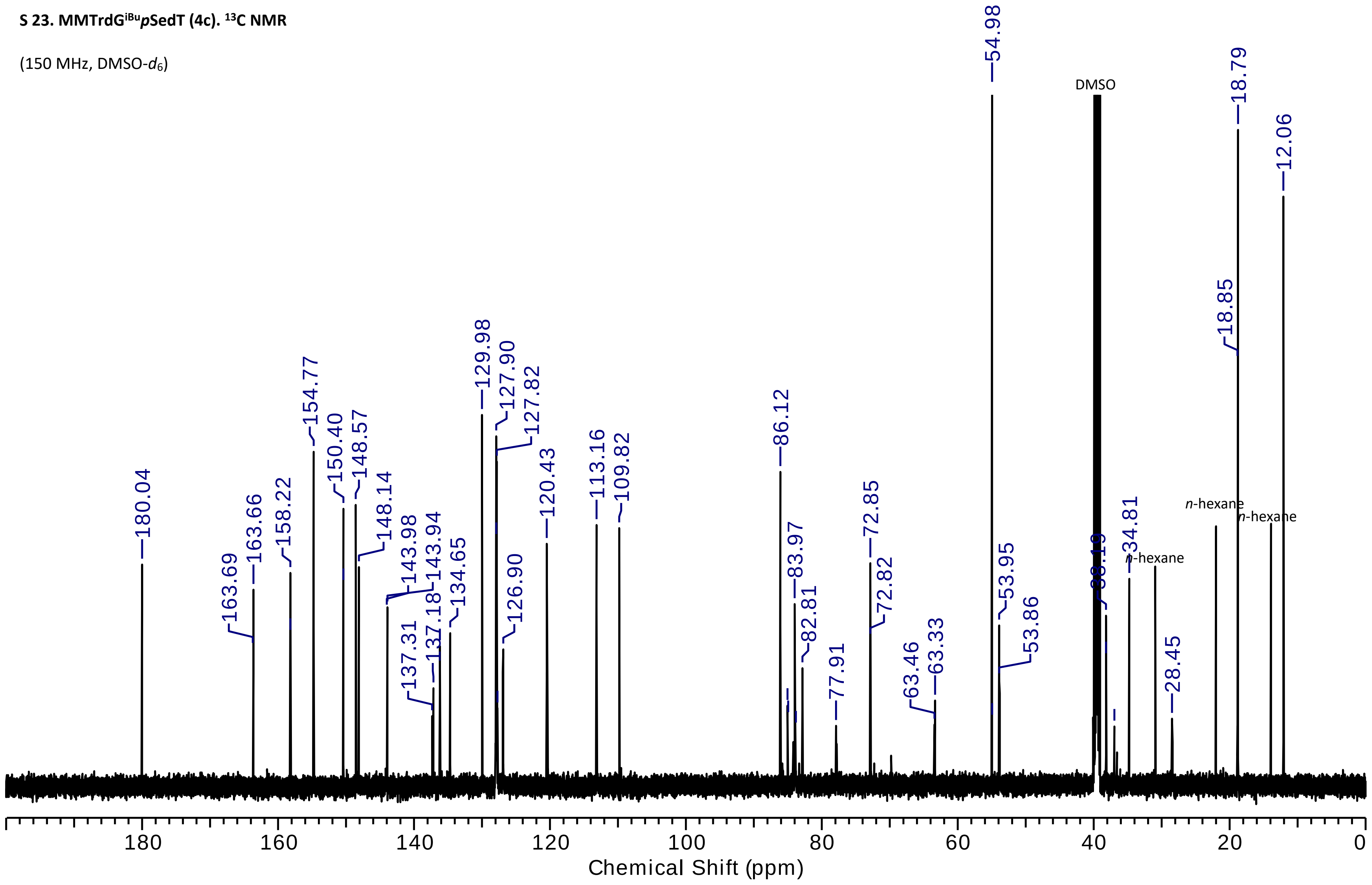

S 24. MMTrdG^{iBu}*p*SedT (4c). $^{31}P\{^{1}H\}$ NMR

(243 MHz, DCM, D_2O external lock)

With expansion to show P-Se couplings

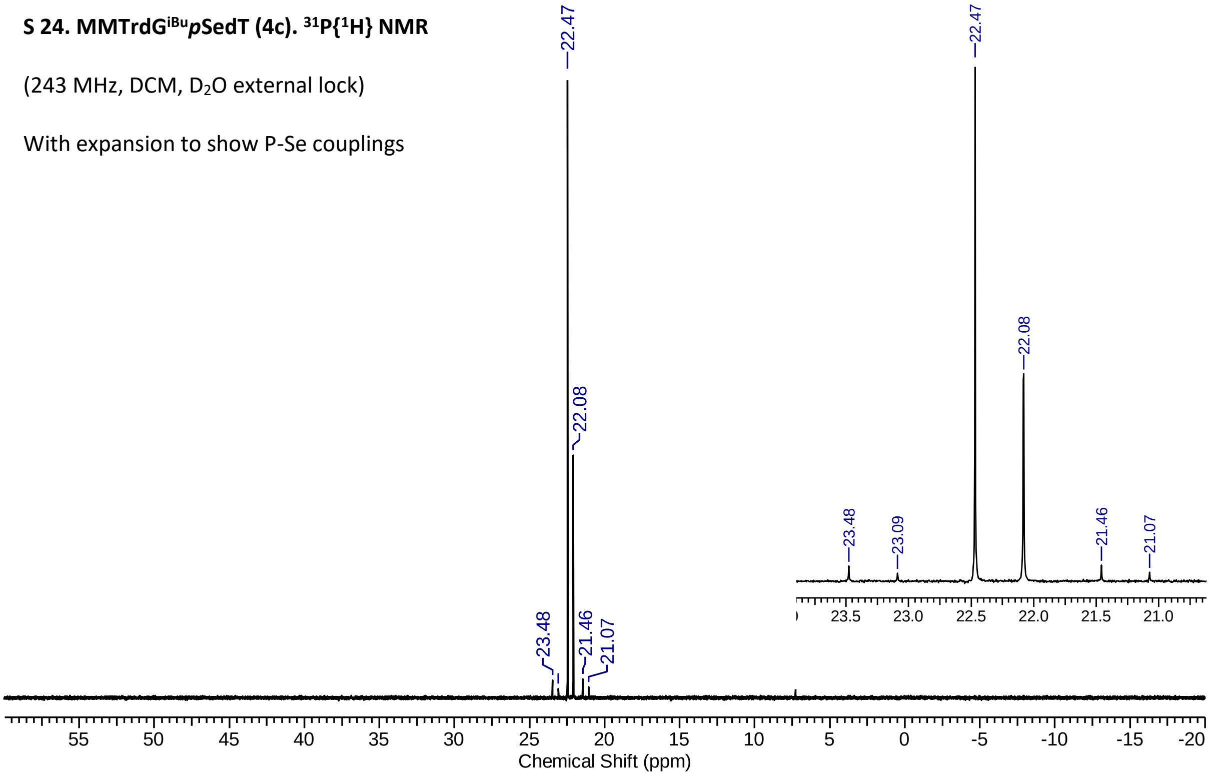

S 25. MMTrdG^{iBu}*p*SedT (4c). $^{77}Se\{^{1}H\}$ NMR

(114 MHz, DMSO-d_6 with external 0.25M KSeCN / D_2O Insert)

With expansion to show Se-P couplings

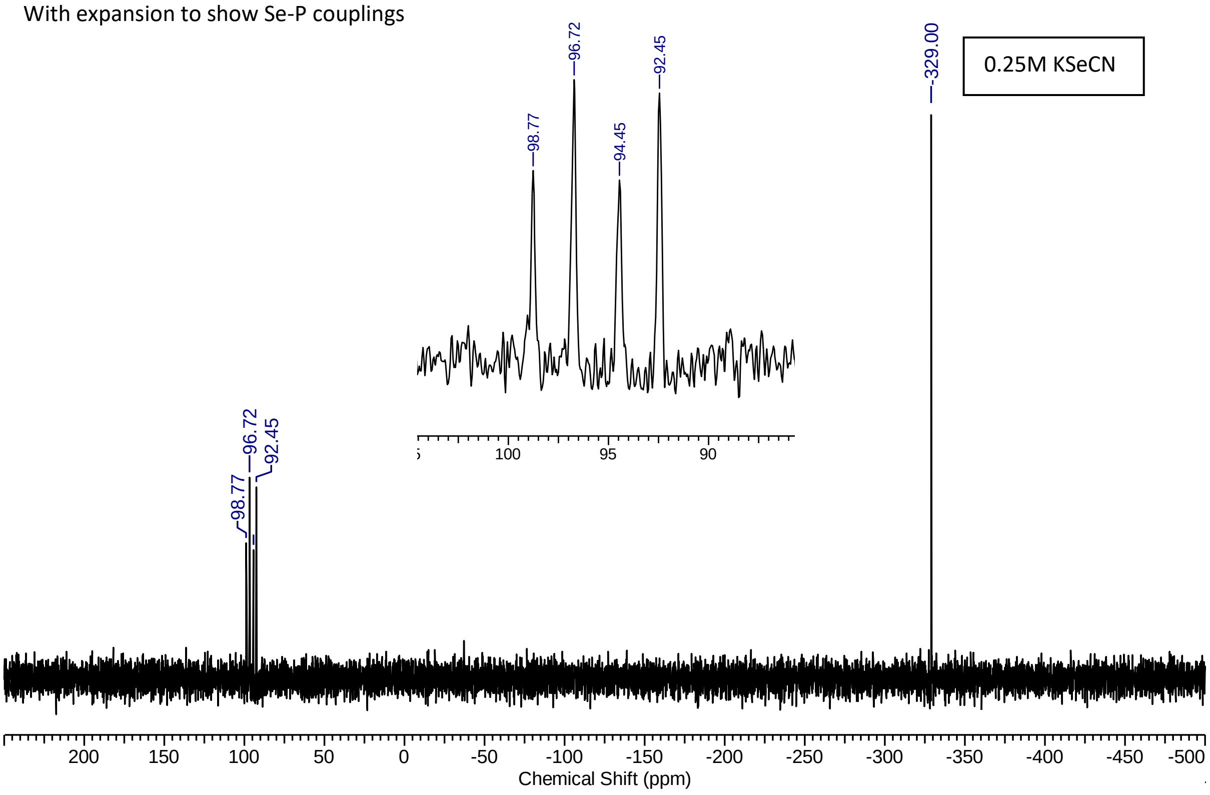

S 26. MMTrdG^{iBu}*p*SedT (4c). ES Mass spec

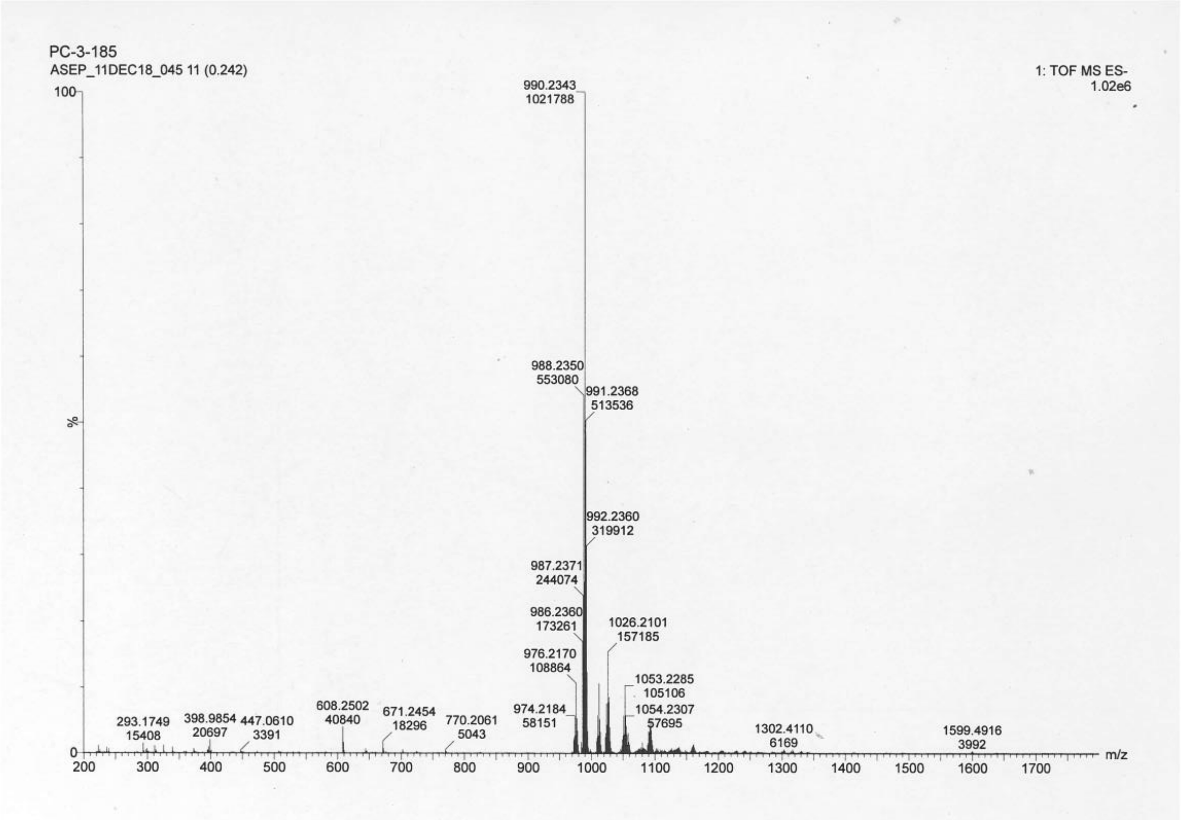

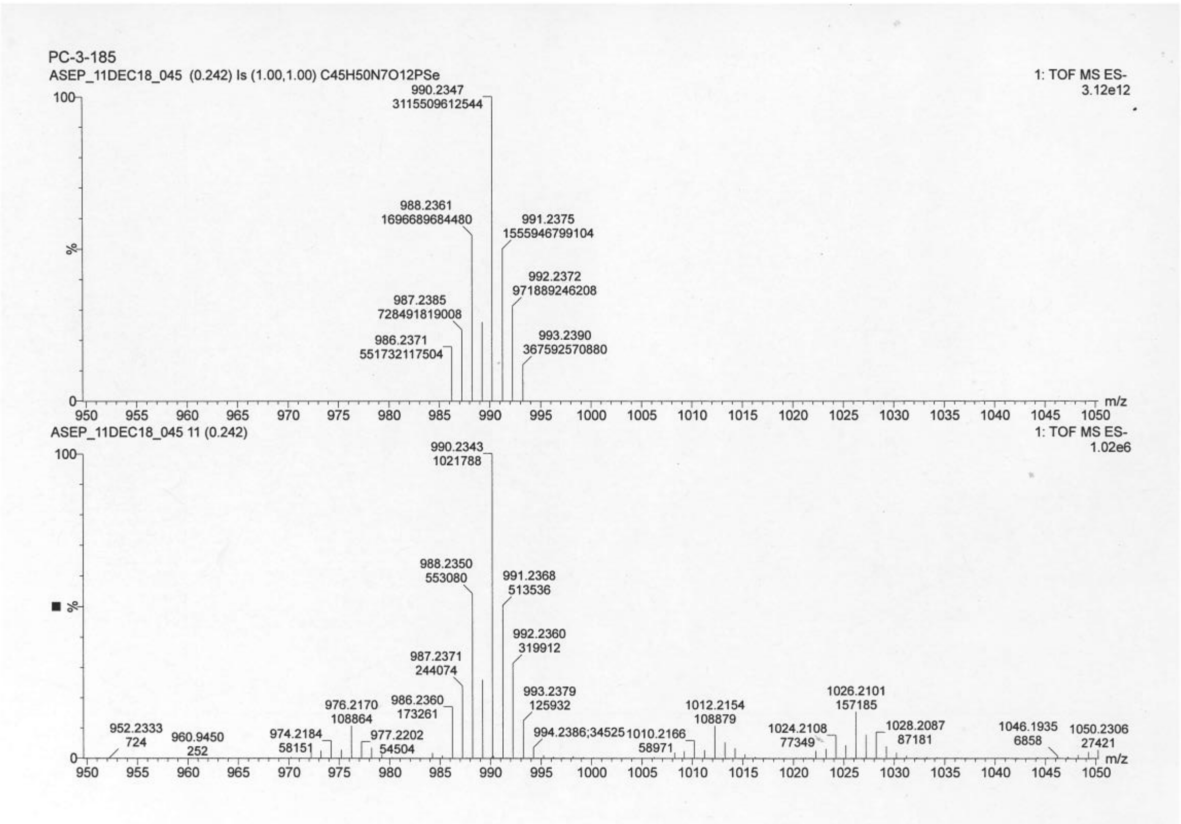

S 27. DMTrT*p*SedT (4d). ^{1}**H NMR**

(600 MHz, DMSO-d_6)

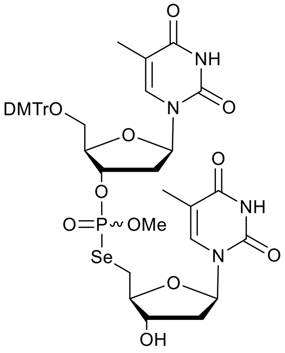

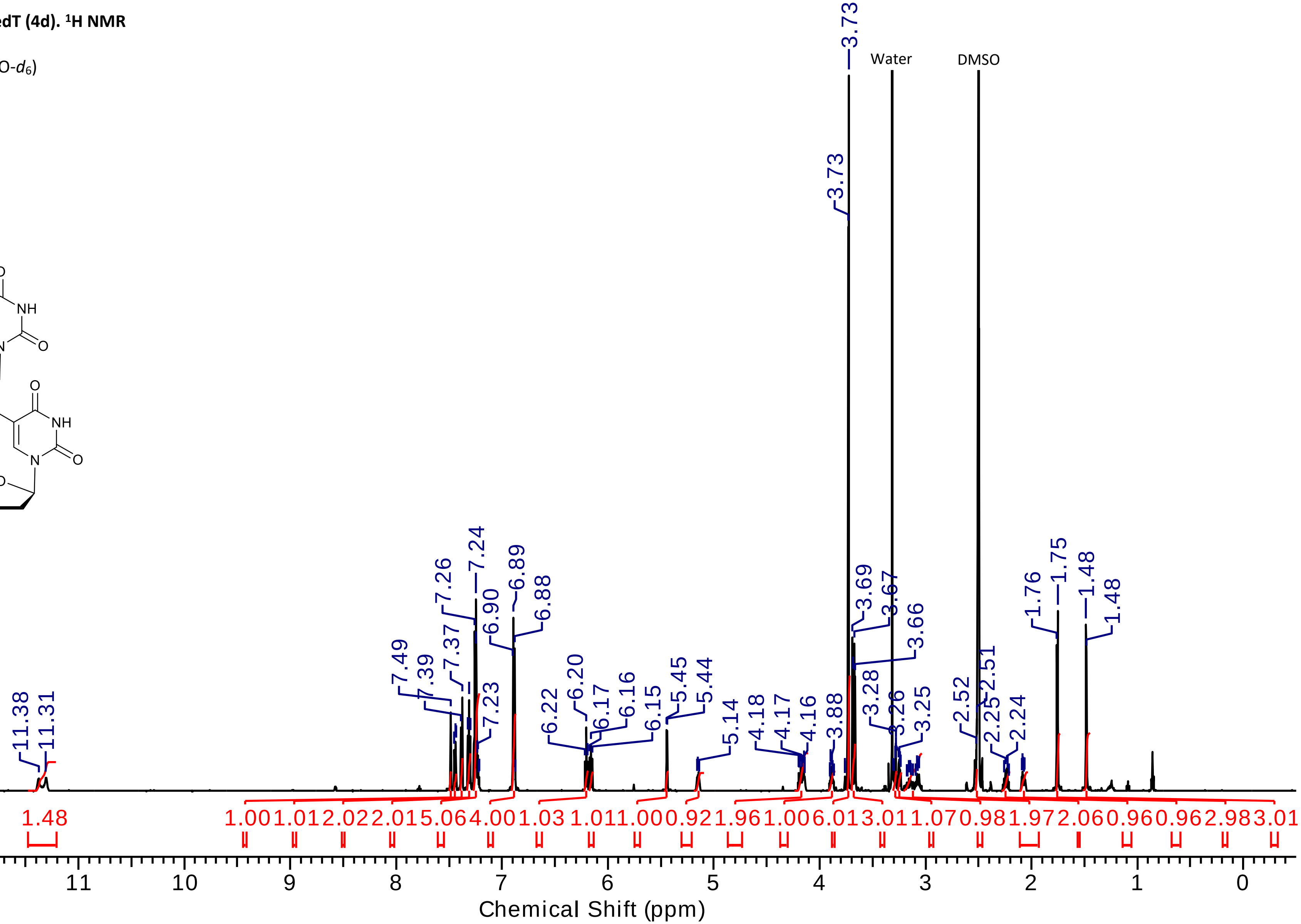

S 28. DMTrT*p*SedT (4d). ^{13}C NMR

(150 MHz, DMSO-d_6)

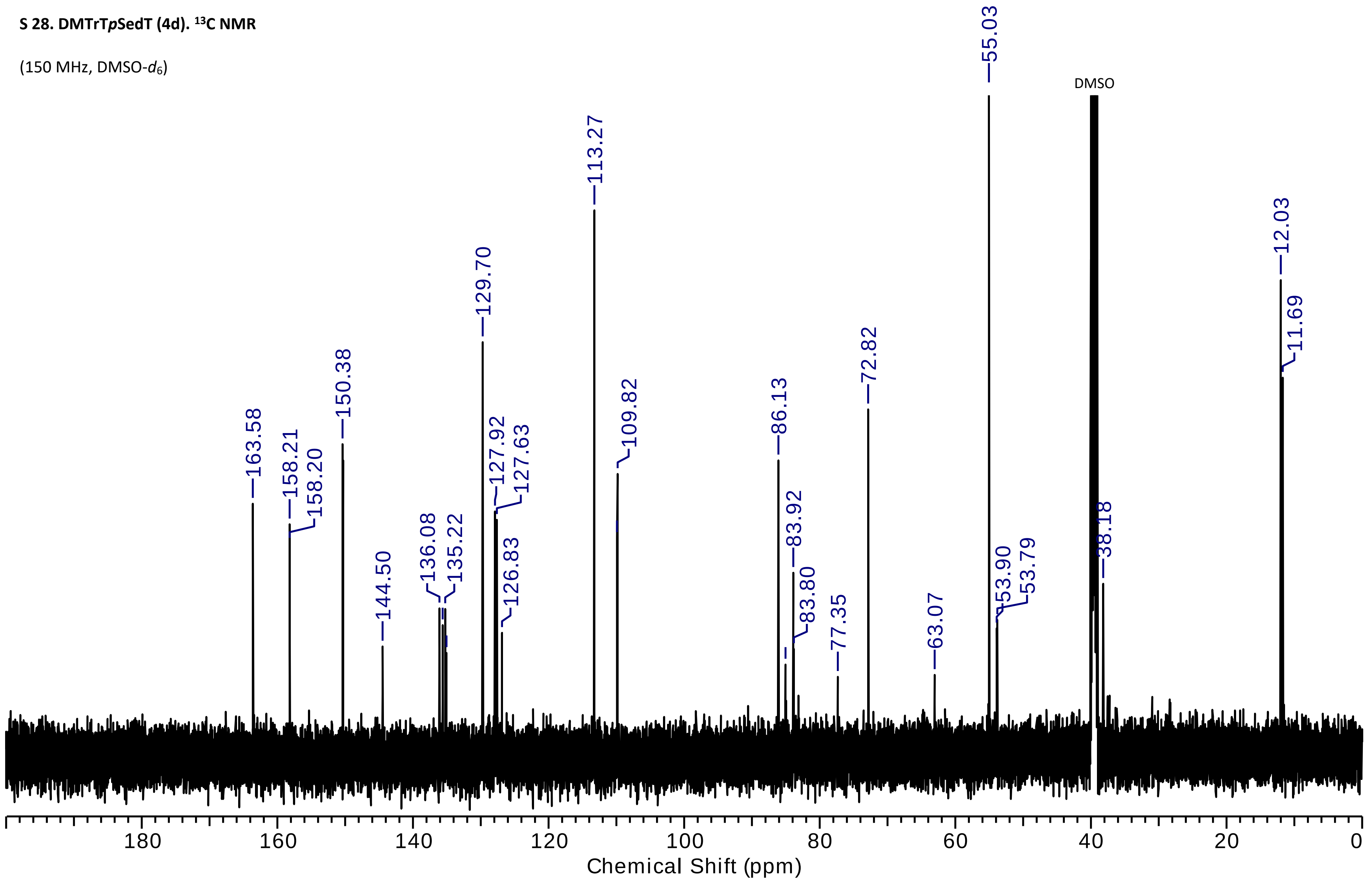

S 29. DMTrT*p*SedT (4d). $^{31}P\{^{1}H\}$ NMR

(243 MHz, MeCN, D_2O external lock)

With expansion to show P-Se couplings

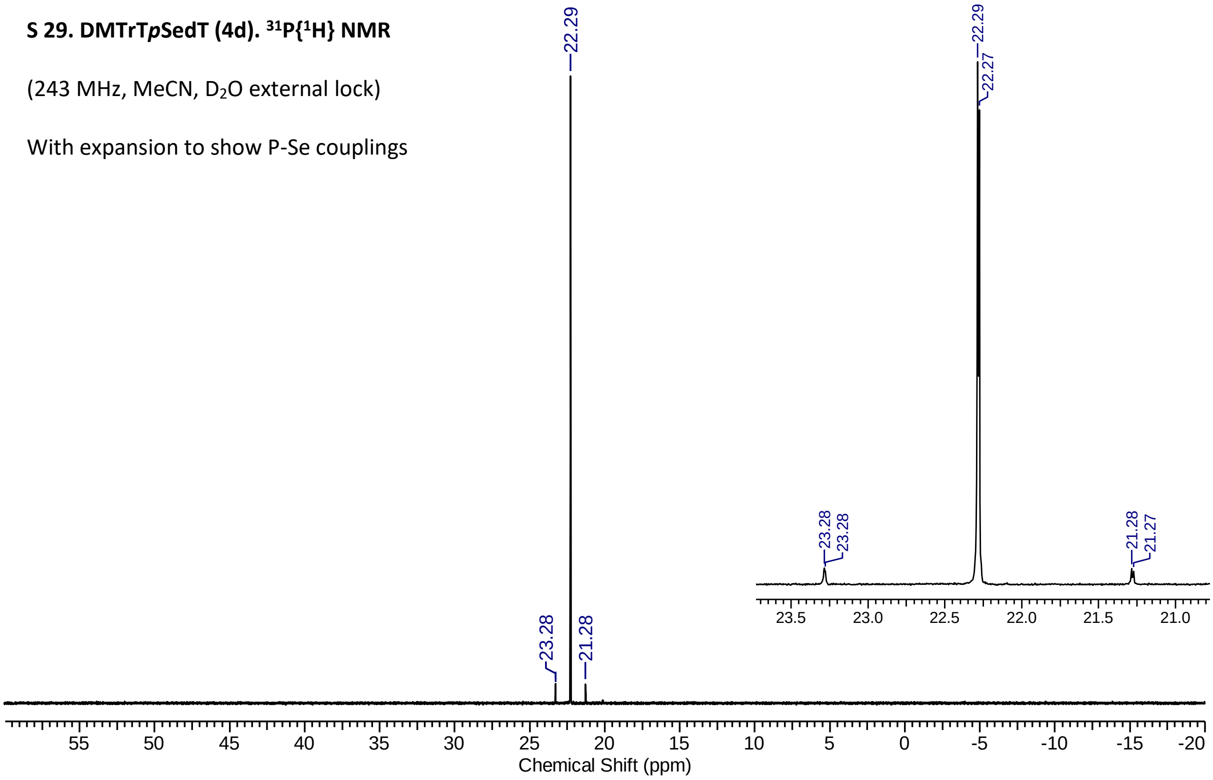

S 30. DMTrT*p*SedT (4d). $^{77}Se\{^{1}H\}$ NMR

(114 MHz, DMSO-d_6 with external 0.25M KSeCN / D_2O Insert)

With expansion to show Se-P couplings

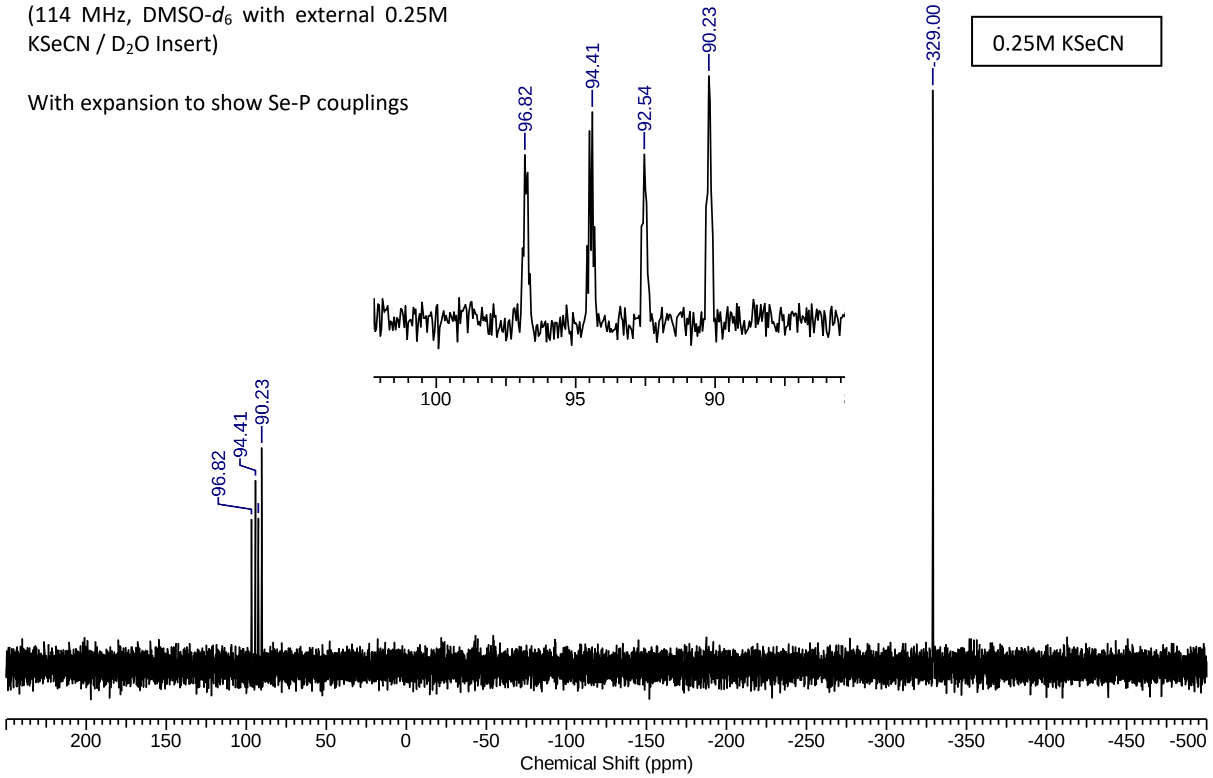

S 31. DMTrT*p*SedT (4d). ES Mass spec

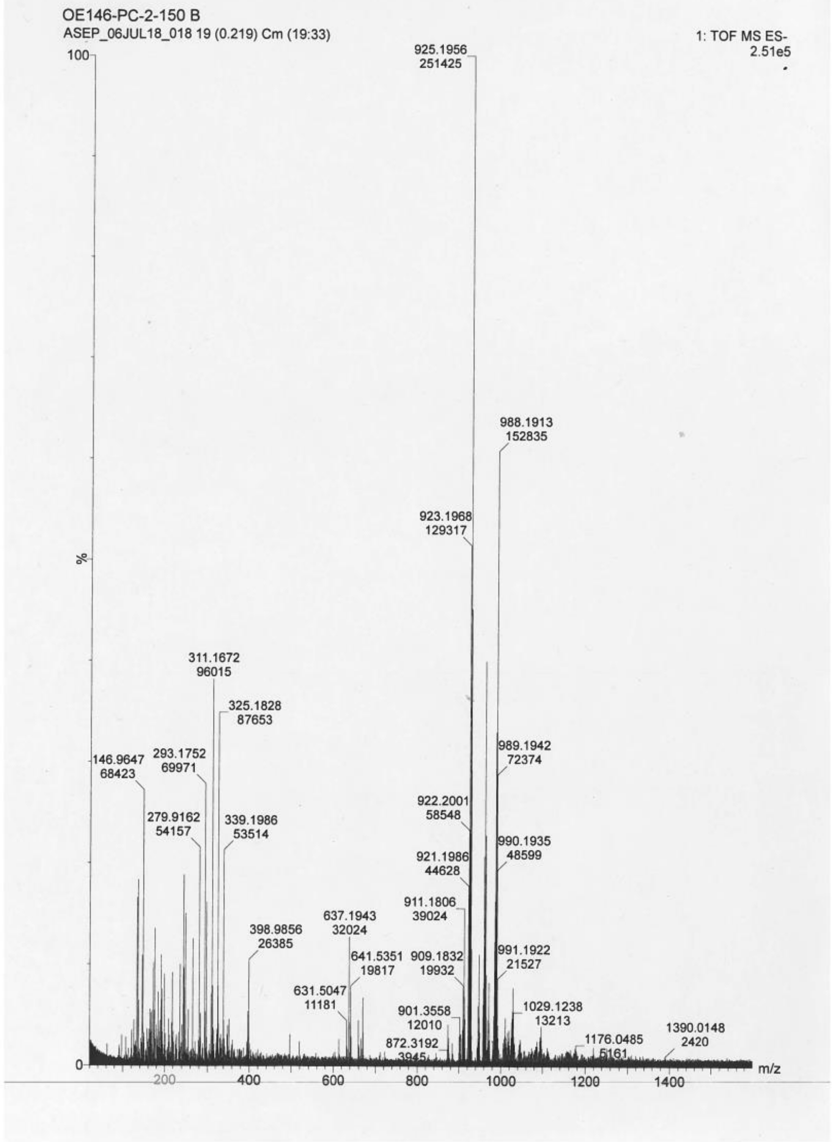

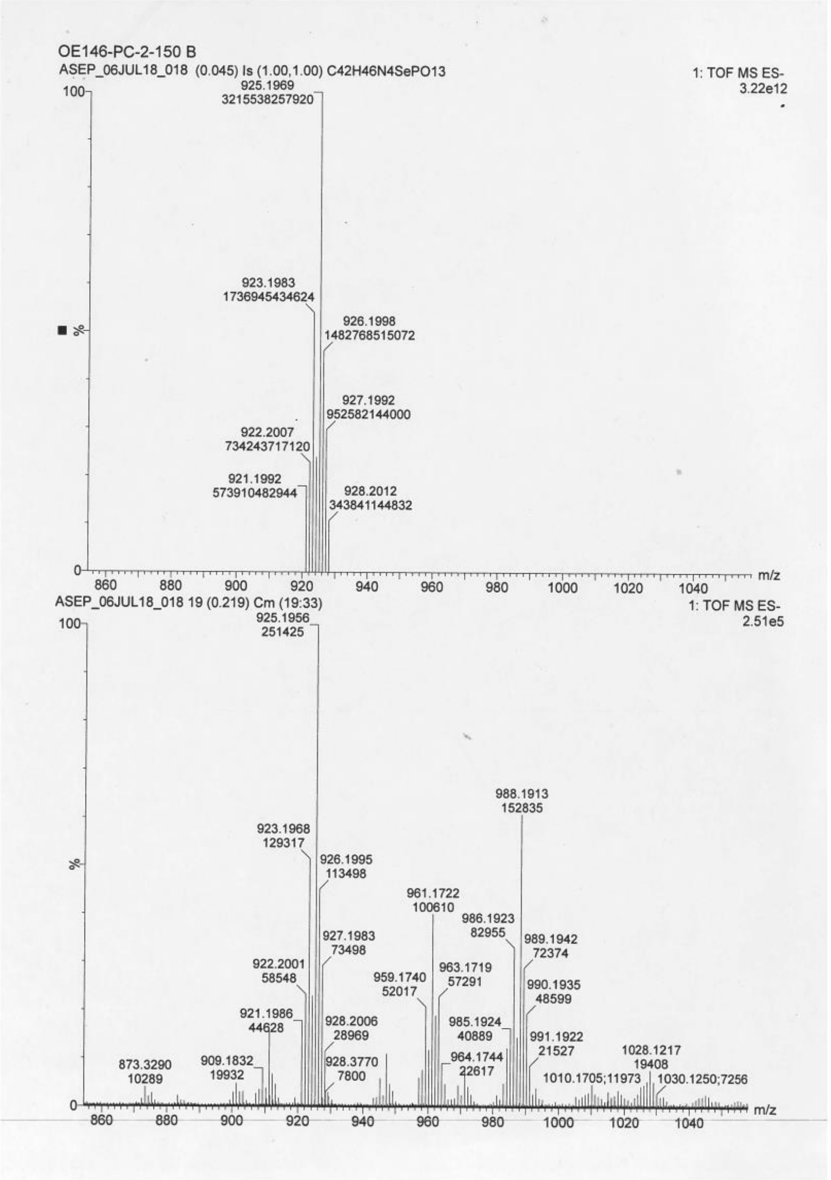

S 32. DMTrdG^{iBu}*p*SedT (4e). ^{1}H NMR

(600 MHz, DMSO-d_6)

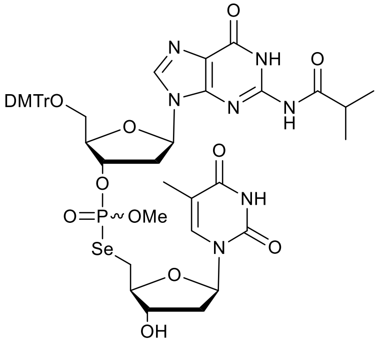

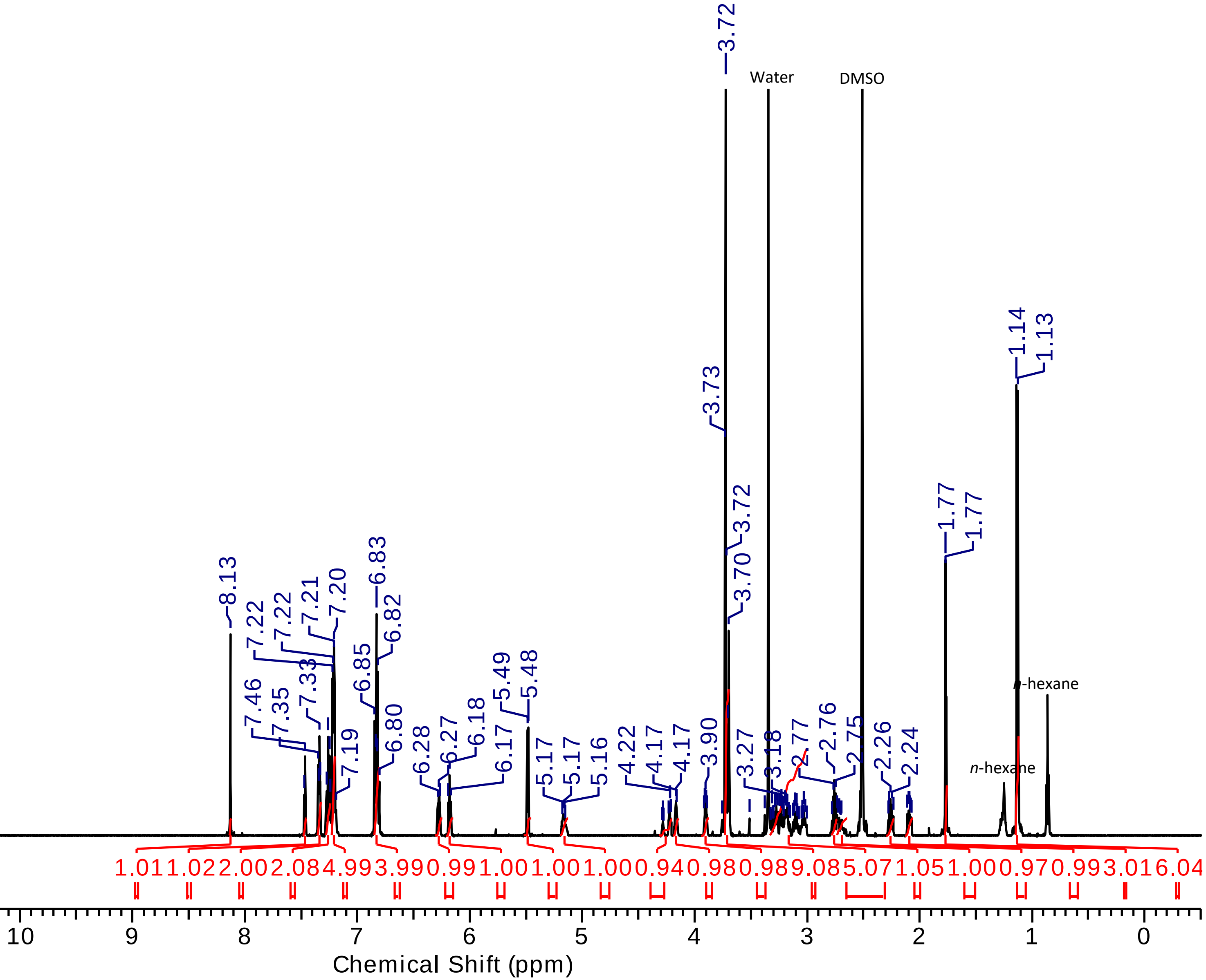

S 33. DMTrdG^{iBu}*p*SedT (4e). ^{13}C NMR

(150 MHz, DMSO-d_6)

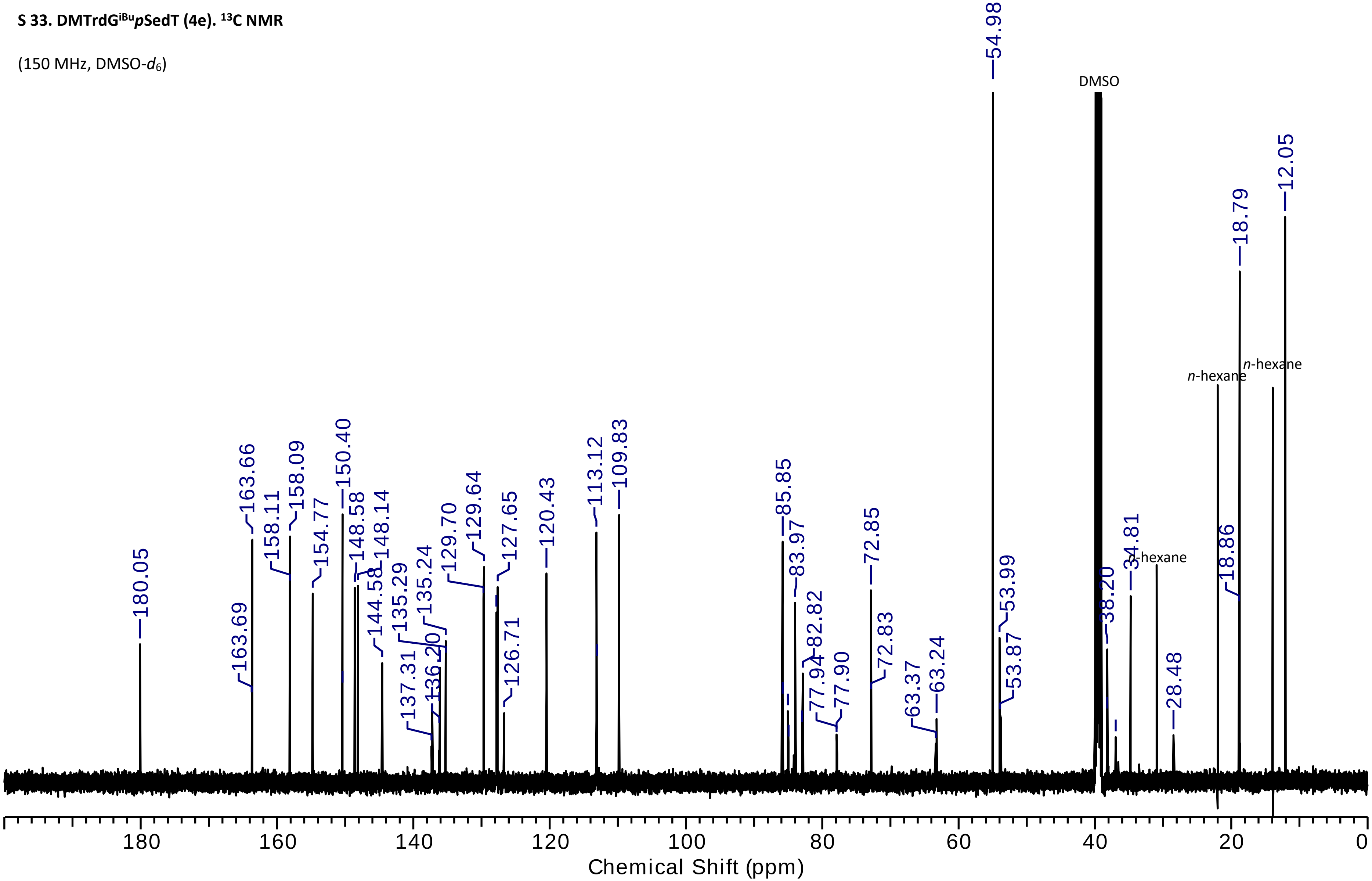

S 34. DMTrdG^{iBu}*p*SedT (4e). $^{31}P\{^{1}H\}$ NMR

(162 MHz, DCM, D_2O external lock)

With expansion to show P-Se couplings

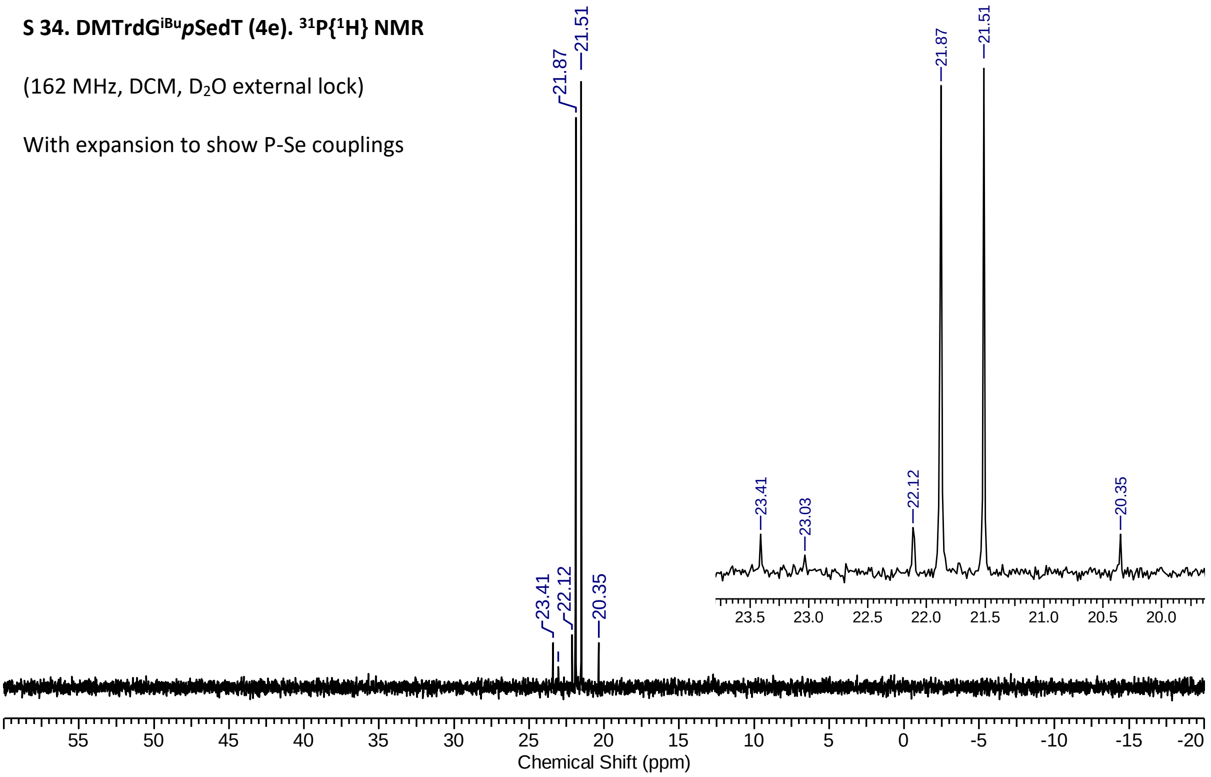

S 35. DMTrdG^{iBu}*p*SedT (4e). $^{77}Se\{^{1}H\}$ NMR

(114 MHz, DMSO-d_6 with external 0.25M KSeCN / D_2O Insert)

With expansion to show Se-P couplings

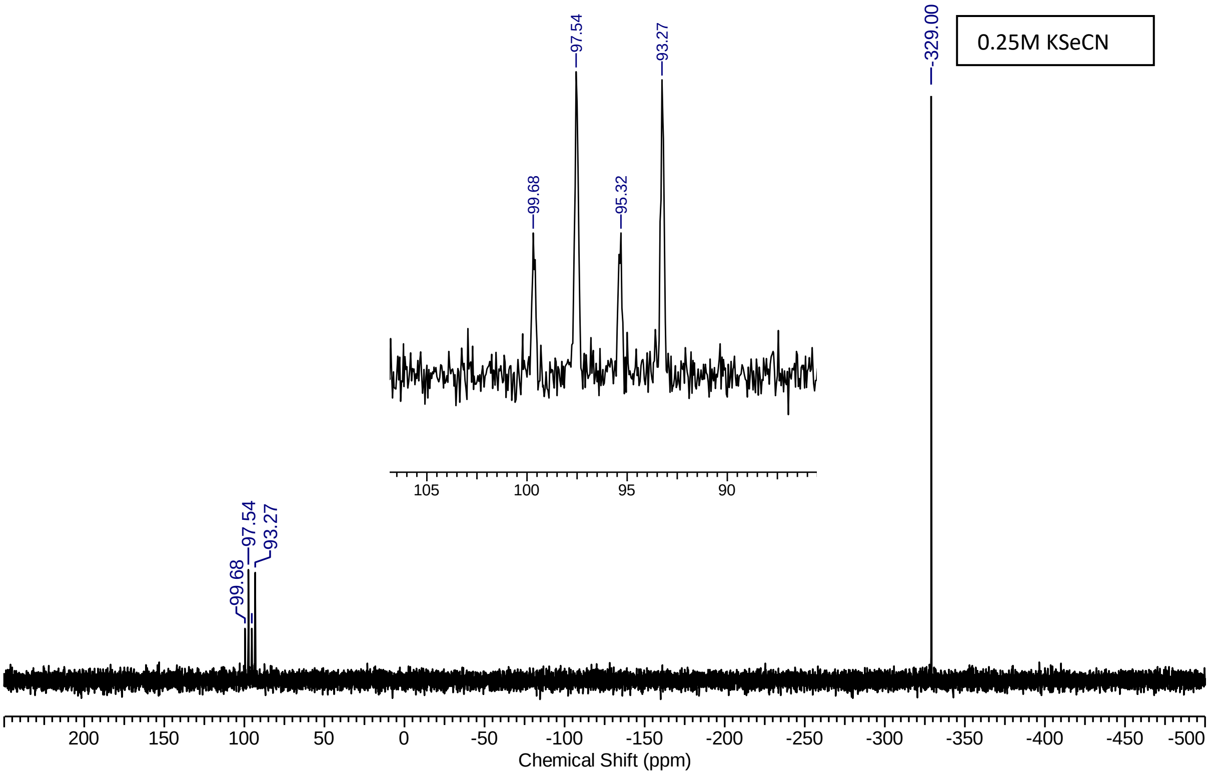

S 36. DMTrdG^{iBu}*p*SedT (4e). ES Mass spec

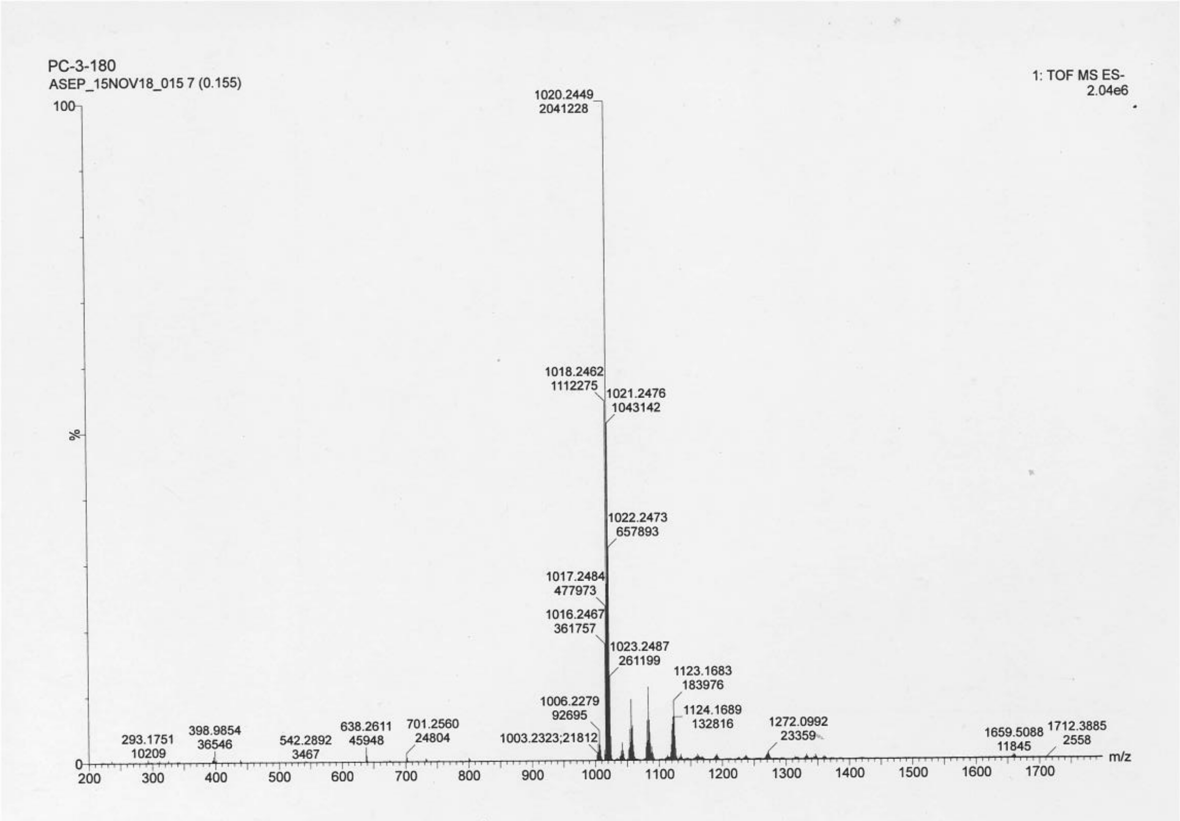

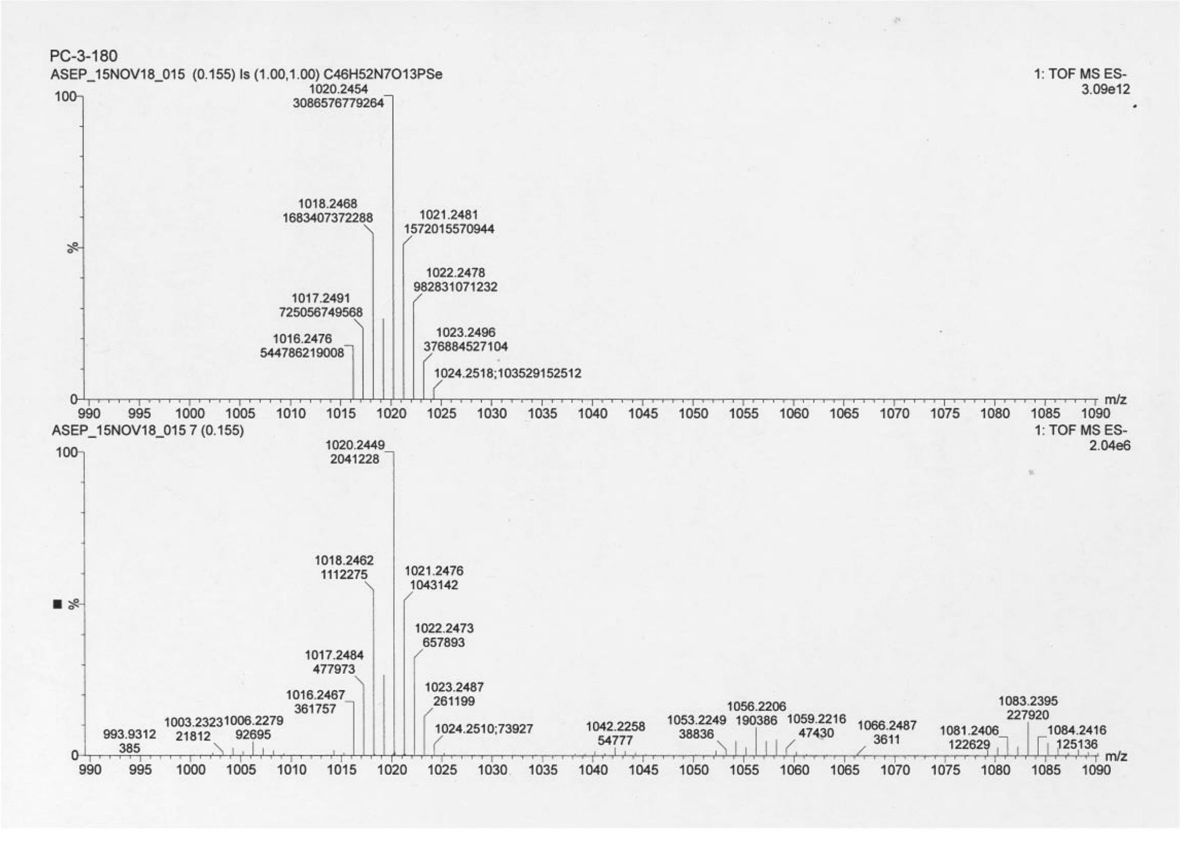

S 37. DMTrdA^{PAc}*p*SedT-Phosphoramidite (5a). $^{31}P\{^{1}H\}$ NMR

(243 MHz, MeCN, D_2O external lock)

With expansion to show P-Se couplings

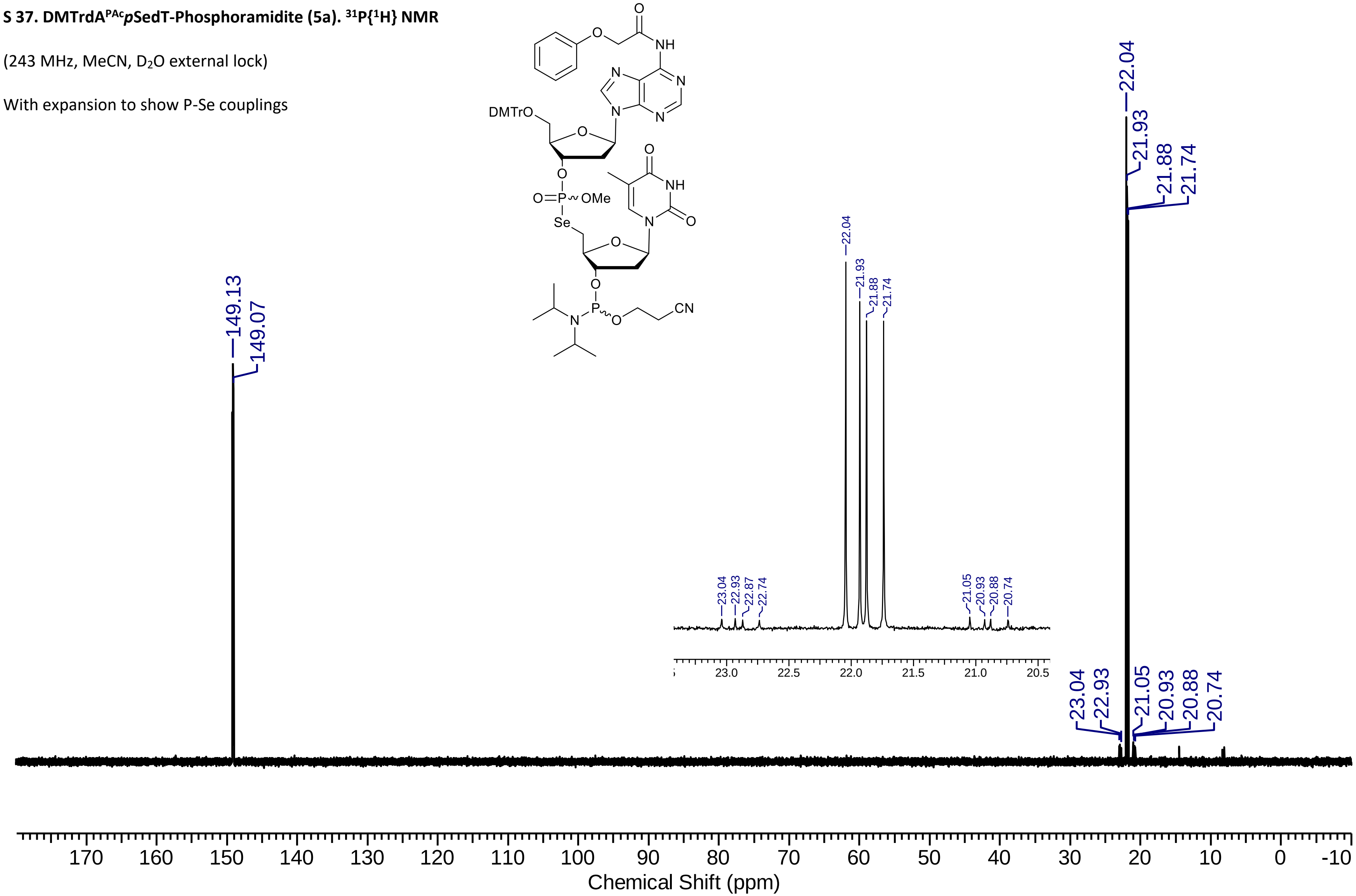

S 38. DMTrdA^{PAc}*p*SedT-Phosphoramidite (5a). ^{1}H NMR

(600 MHz, MeCN-d_3)

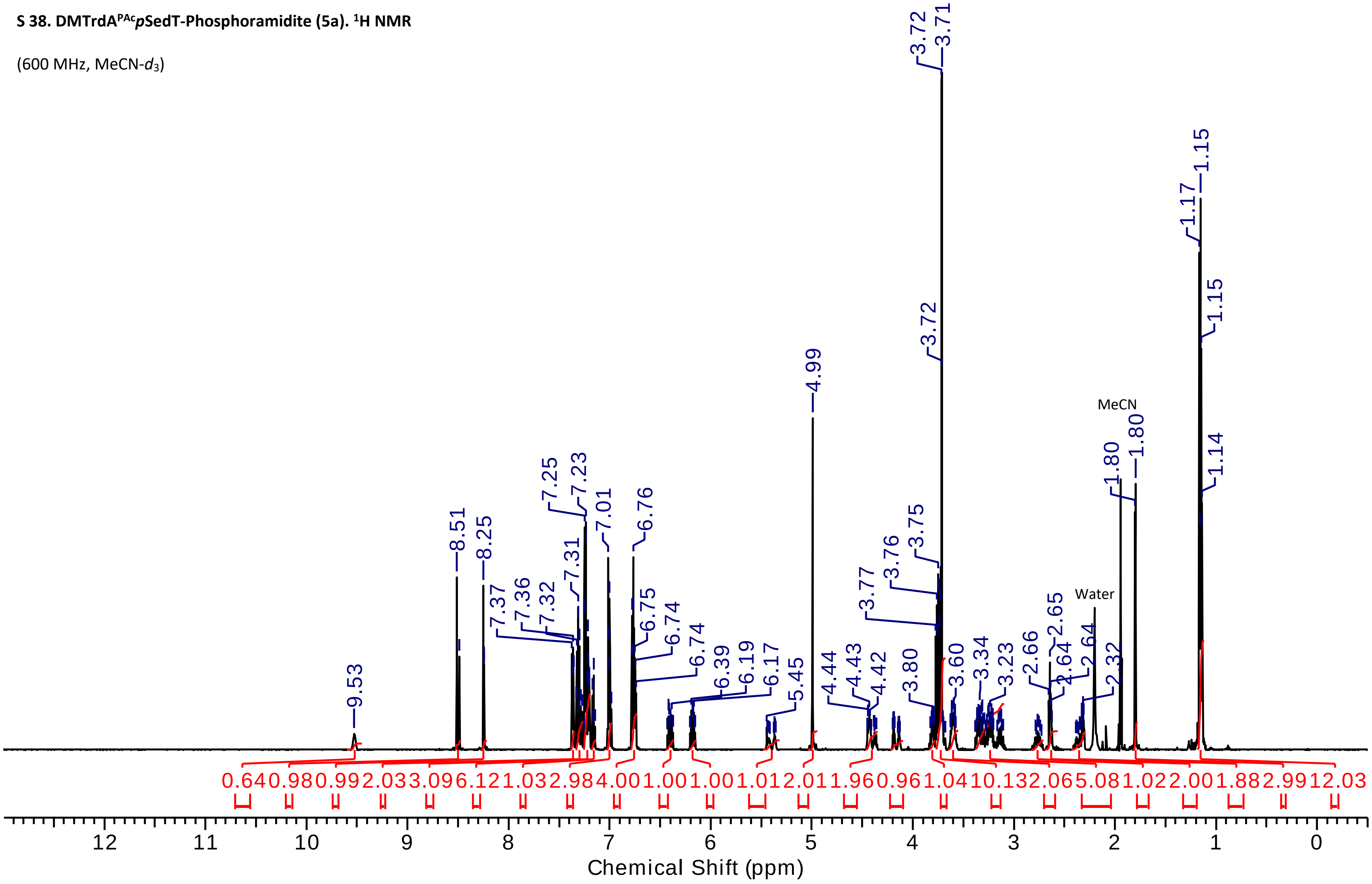

S 39. DMTrdA^{PAc}*p*SedT-Phosphoramidite (5a). ES Mass spec

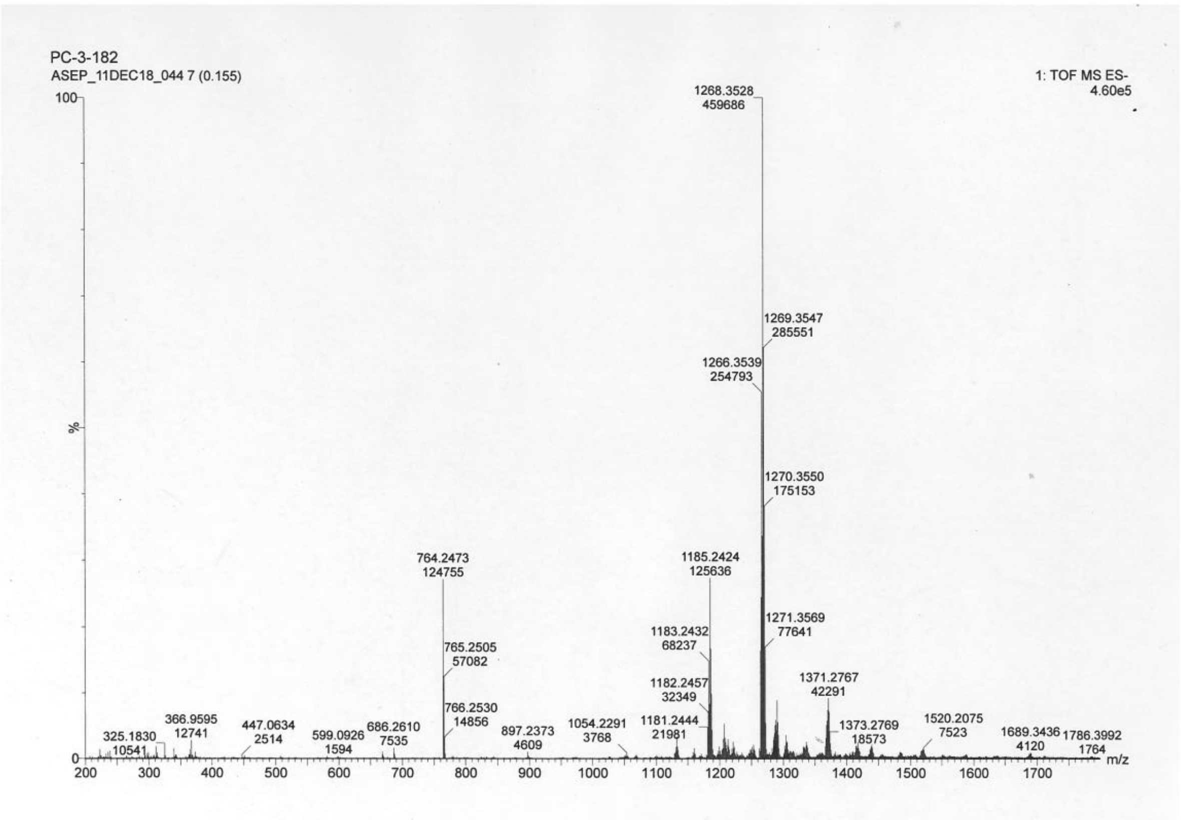

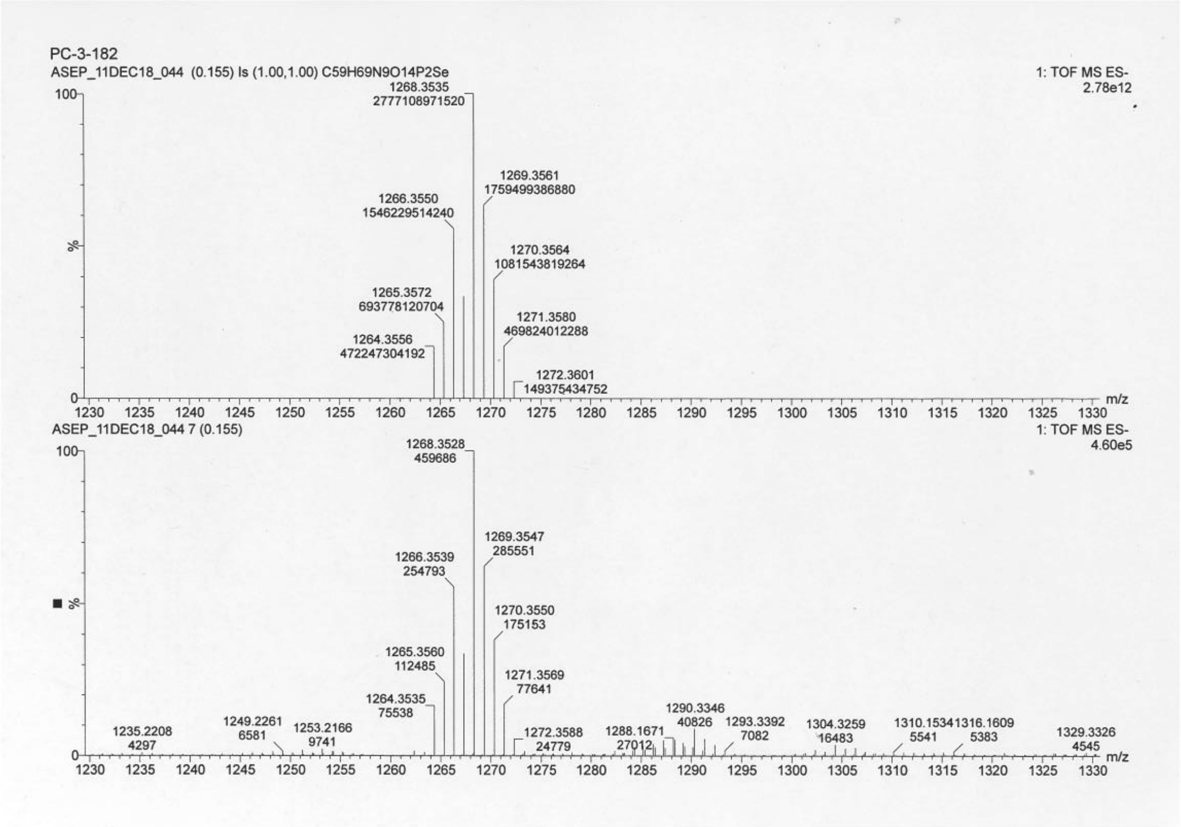

S 40. DMTrdC^{Ac}*p*SedT-Phosphoramidite (5b). $^{31}P\{^{1}H\}$ NMR

(162 MHz, MeCN, D_2O external lock)

With expansion to show P-Se couplings

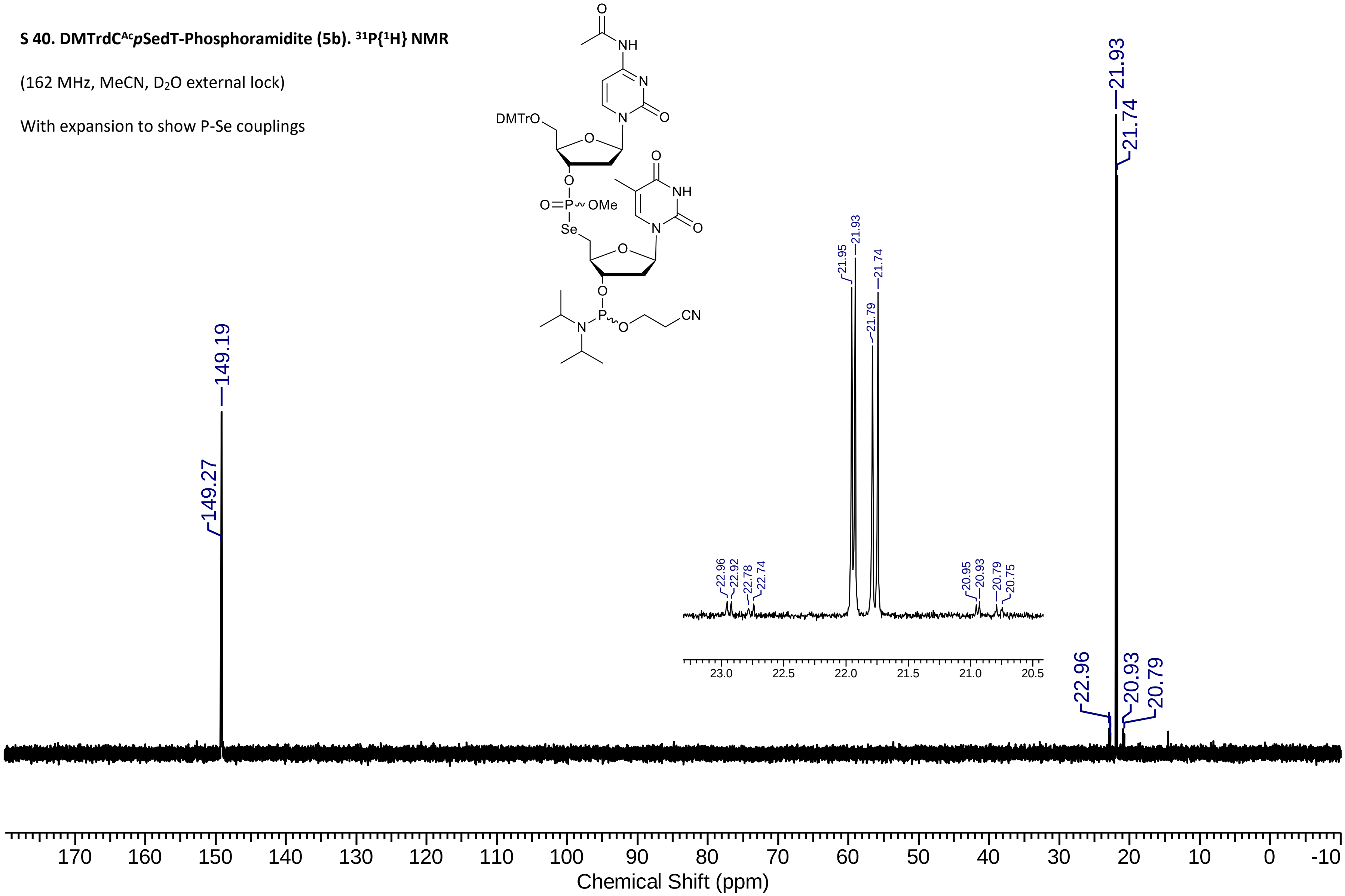

S 41. DMTrdC^{Ac}*p*SedT-Phosphoramidite (5b). ^{1}H NMR

(600 MHz, MeCN-d_3)

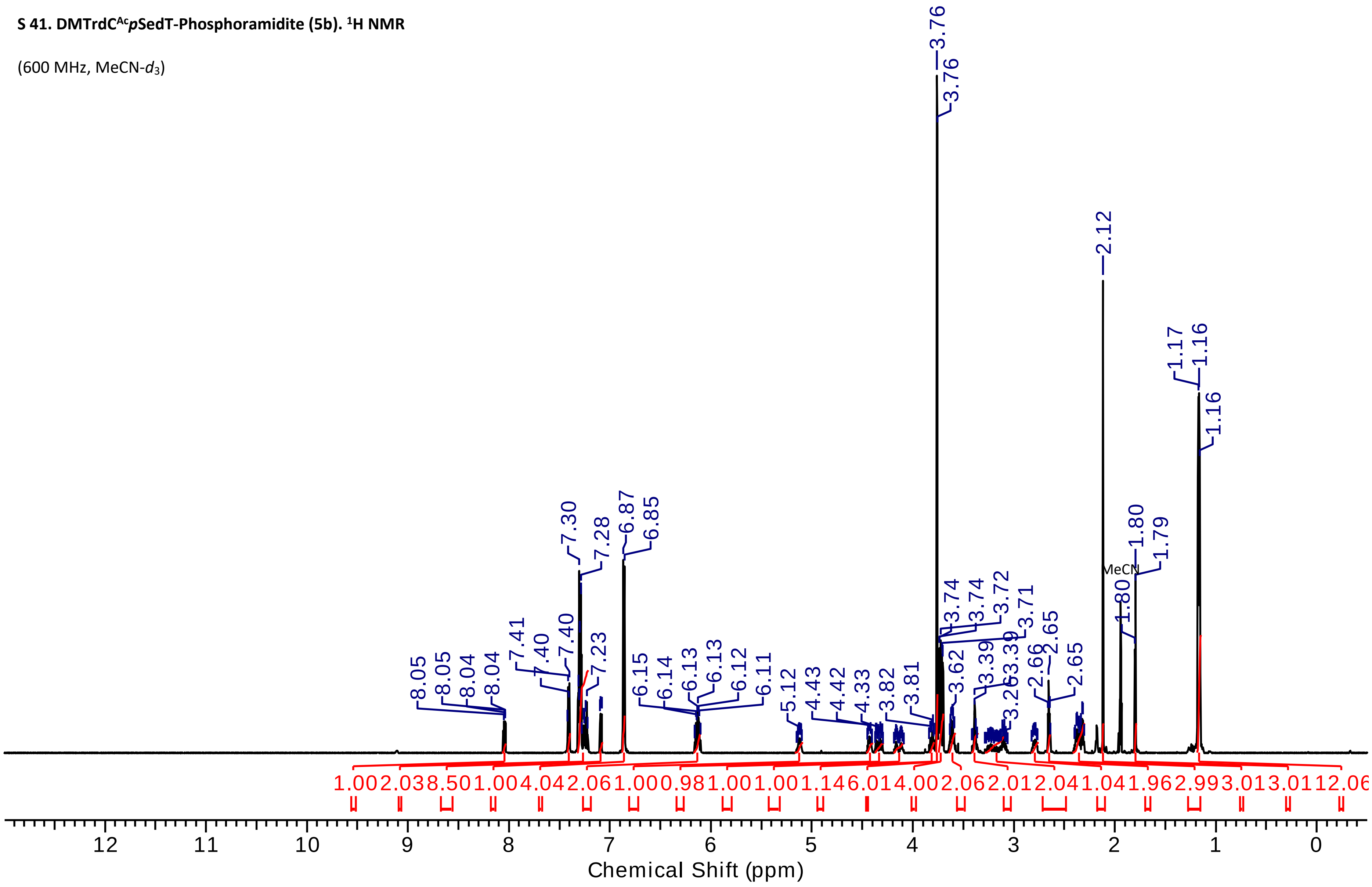

S 42. DMTrdC^{Ac}*p*SedT-Phosphoramidite (5b). ES Mass spec

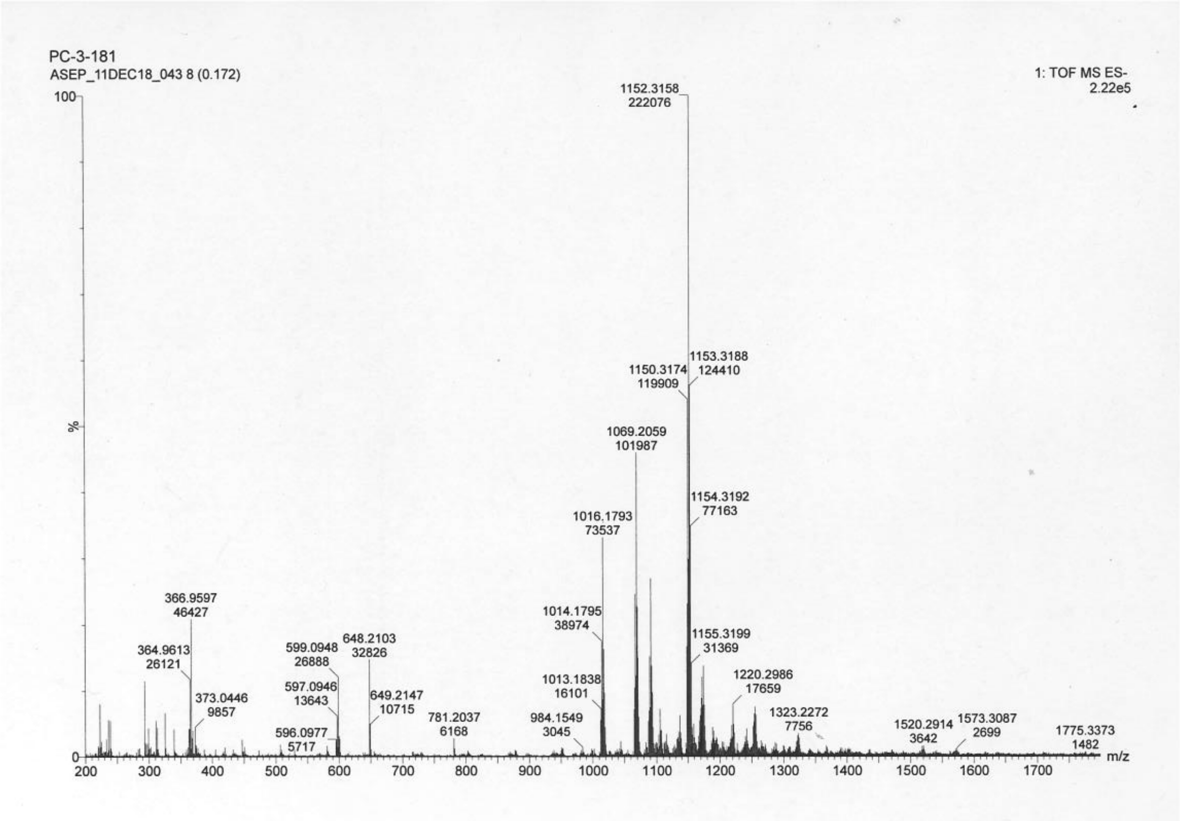

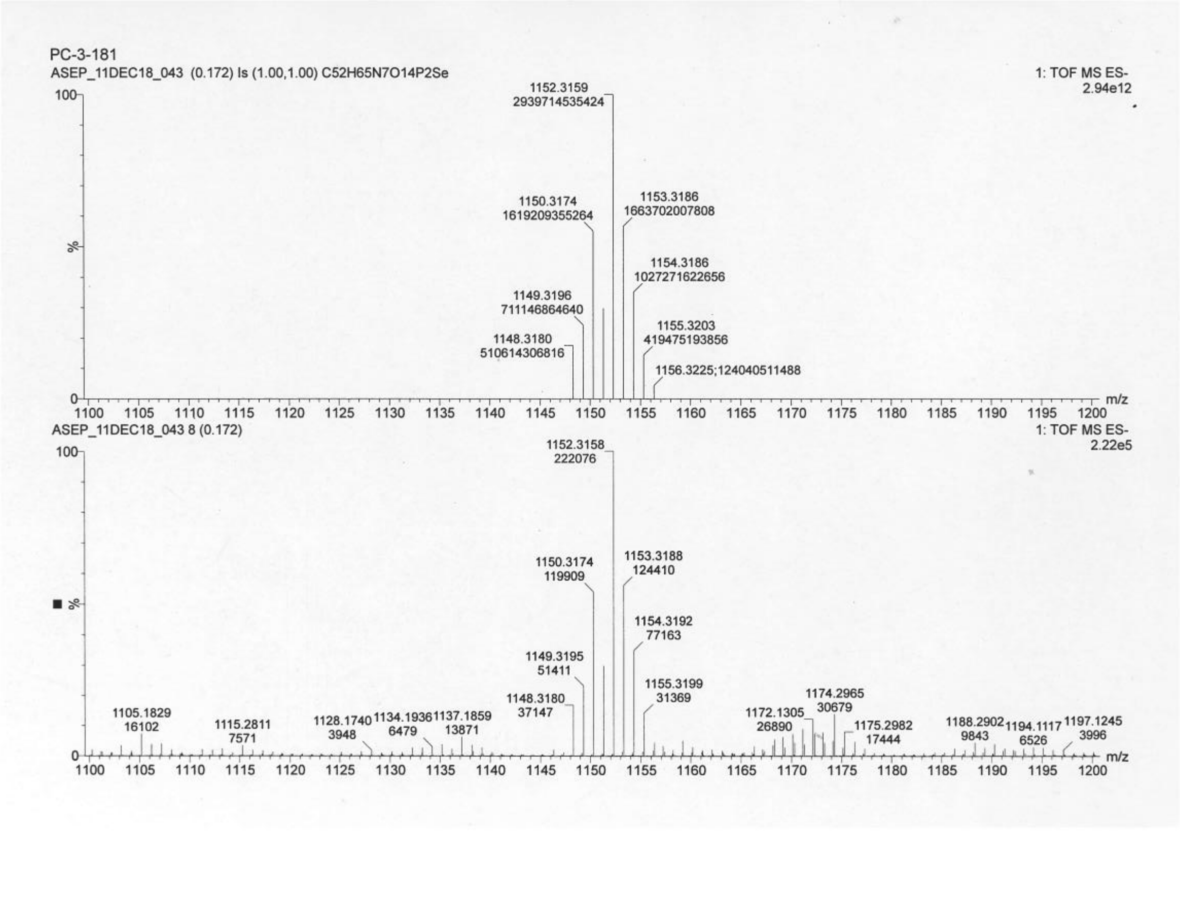

S 43. MMTrdG^{iBu}*p*SedT-Phosphoramidite (5c). $^{31}P\{^1H\}$ NMR

(243 MHz, MeCN, D_2O external lock)

With expansion to show P-Se couplings

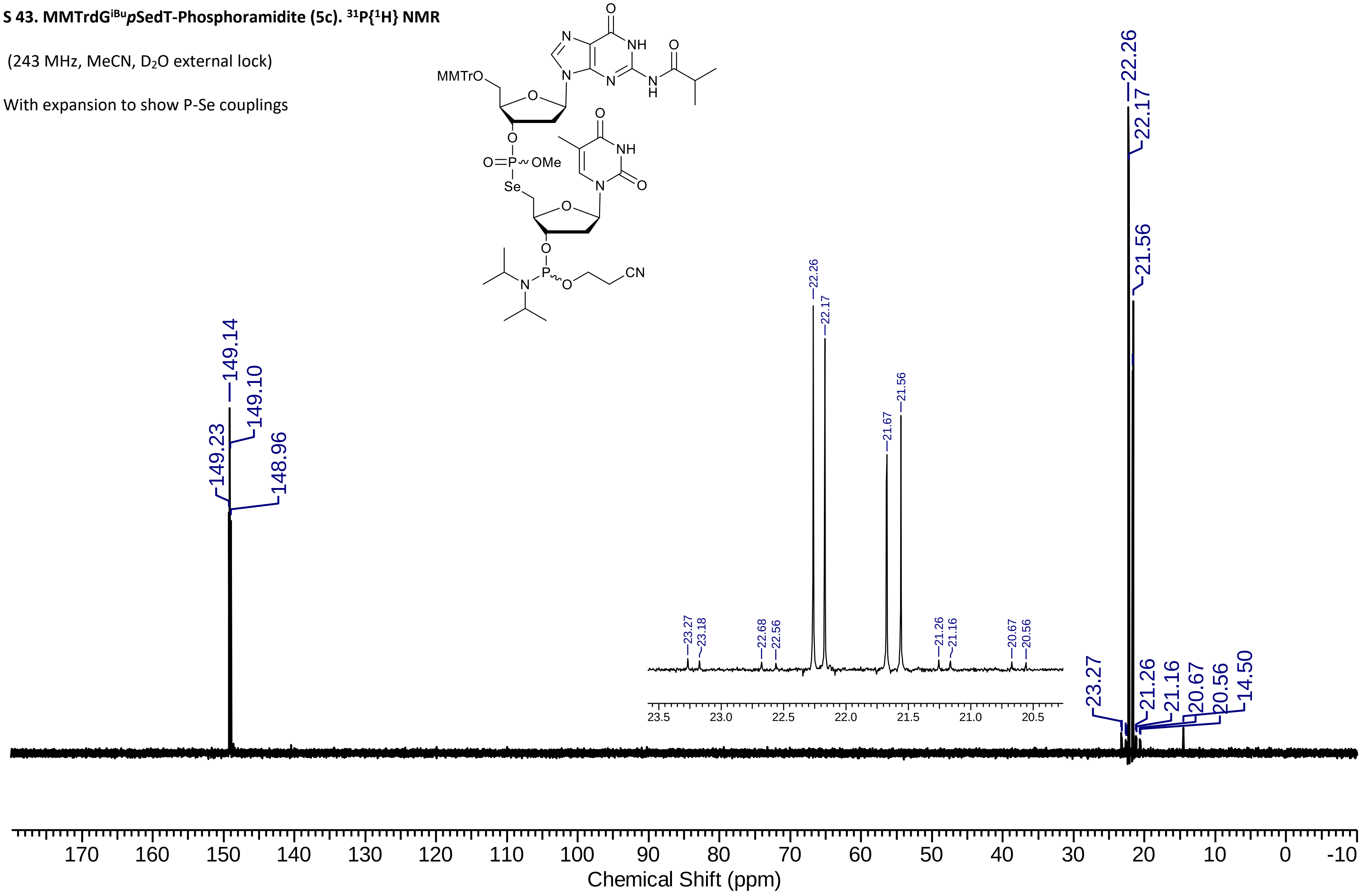

S 44. MMTrdG^{iBu}*p*SedT-Phosphoramidite (5c). ^{1}H NMR

(600 MHz, MeCN-*d*$_3$)

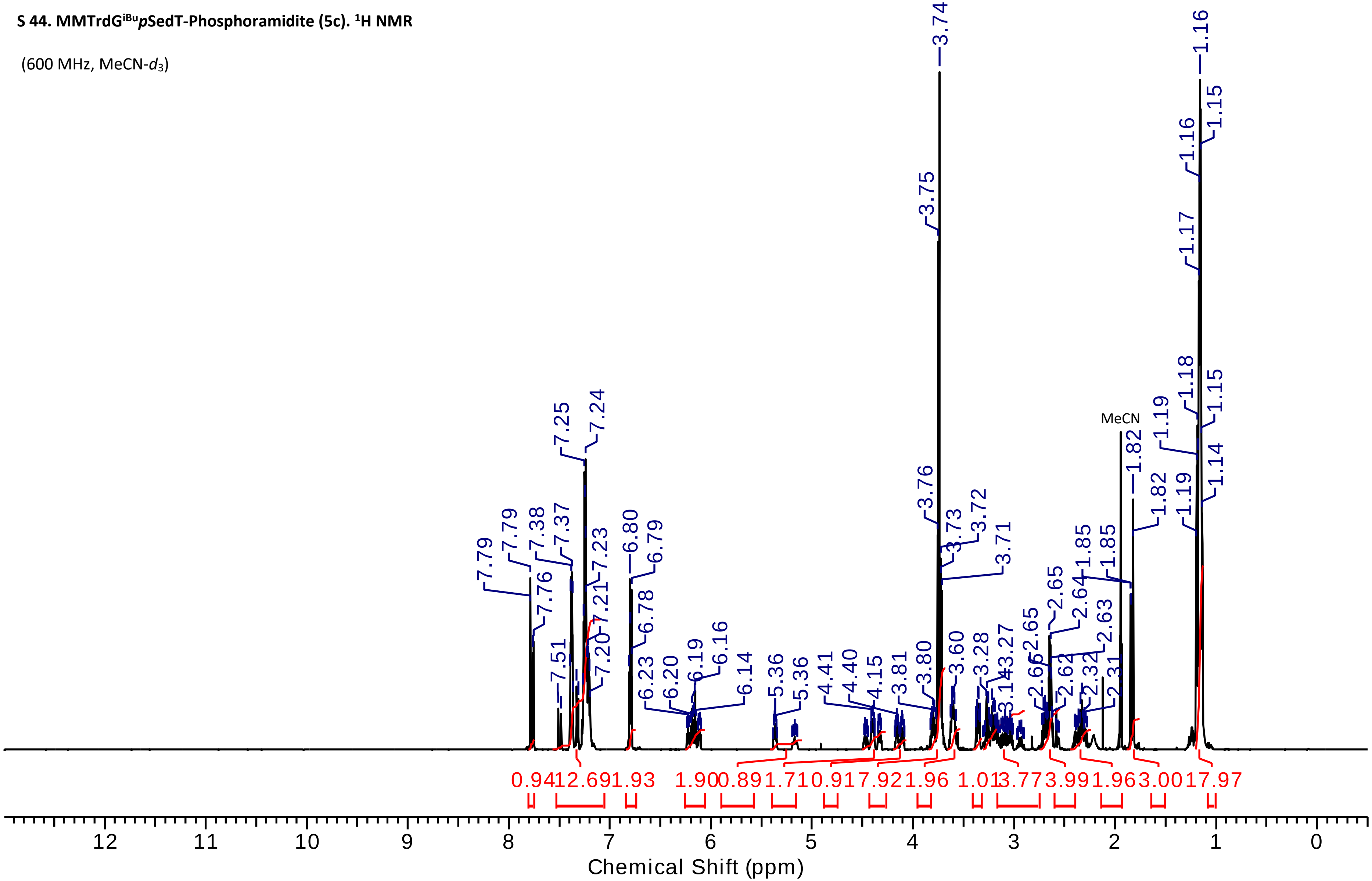

S 45. MMTrdG^{iBu}*p*SedT-Phosphoramidite (5c). ES Mass spec

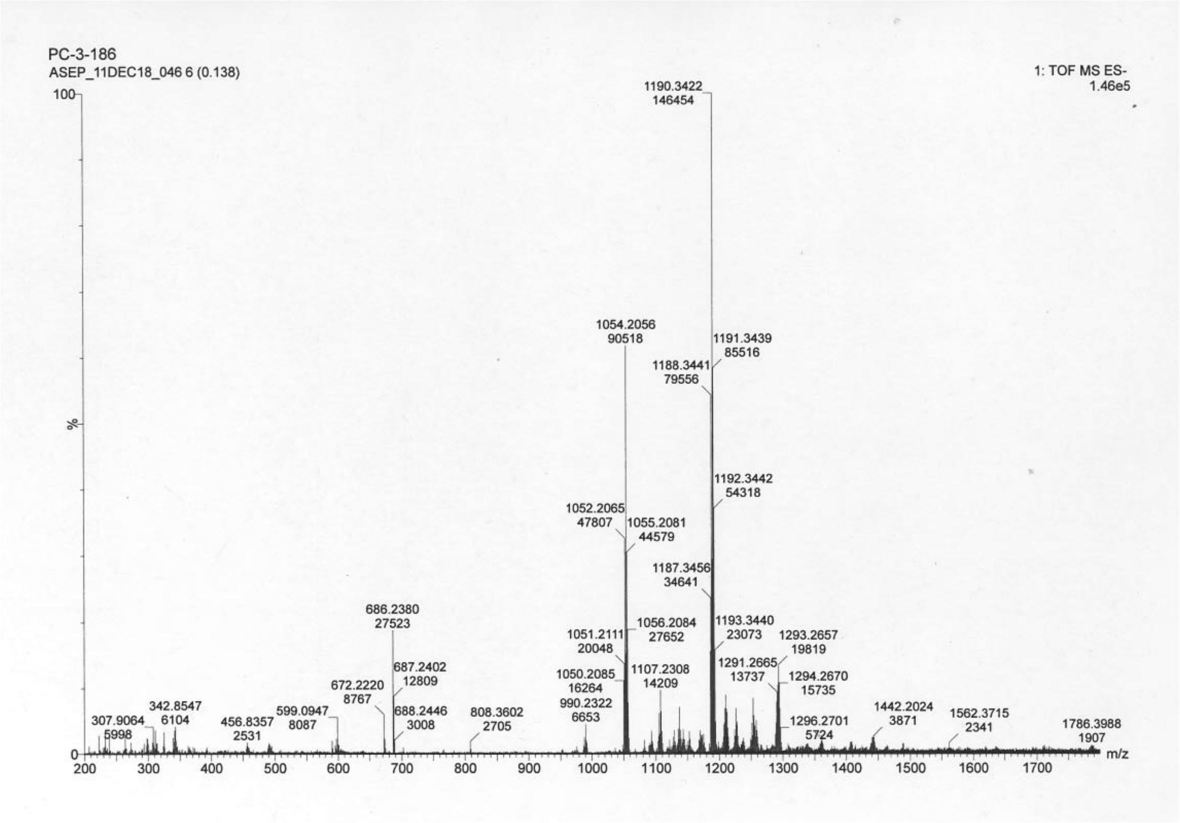

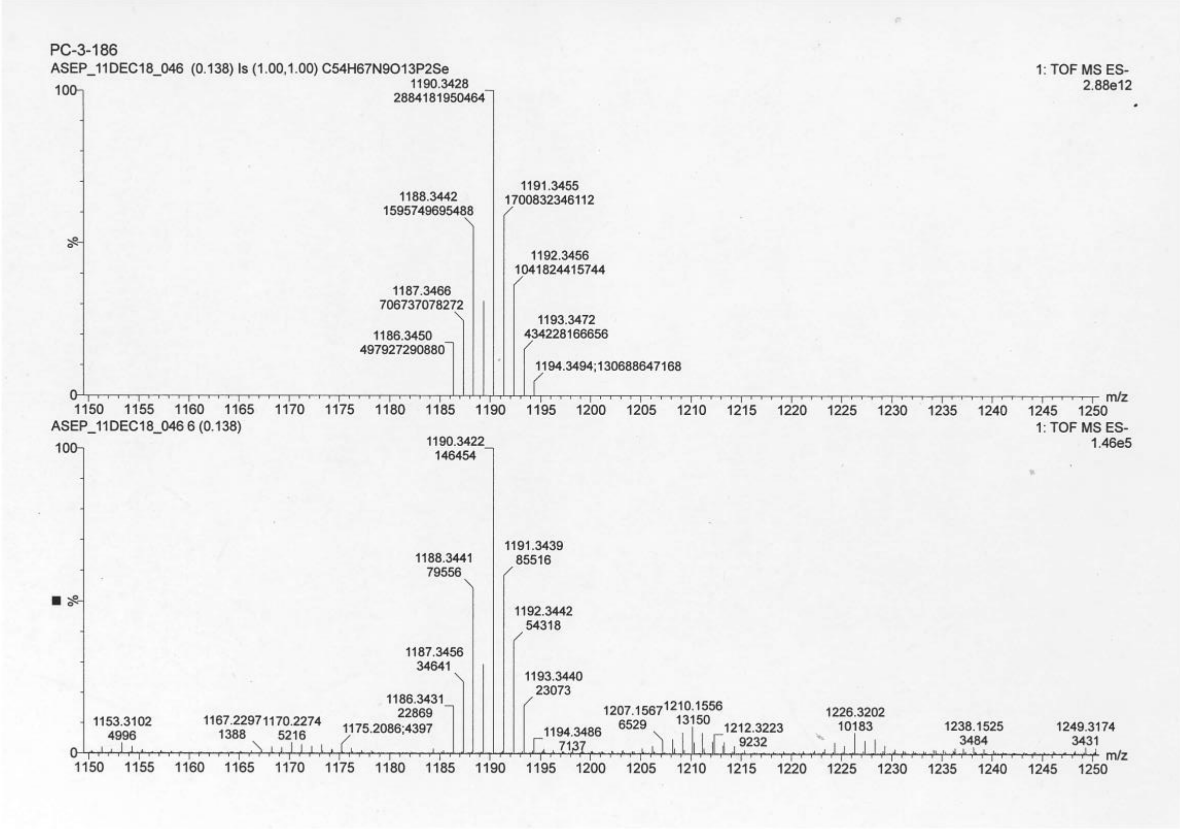

S 46. DMTrT*p*SedT-Phosphoramidite (5d). $^{31}P\{^{1}H\}$ NMR

(243 MHz, MeCN,
D_2O external lock)

With expansion to show
P-Se couplings

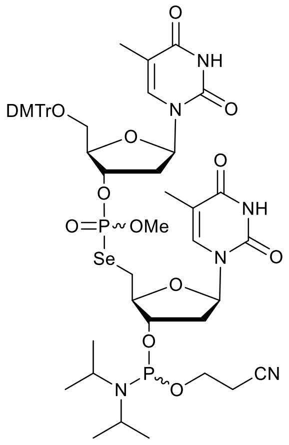

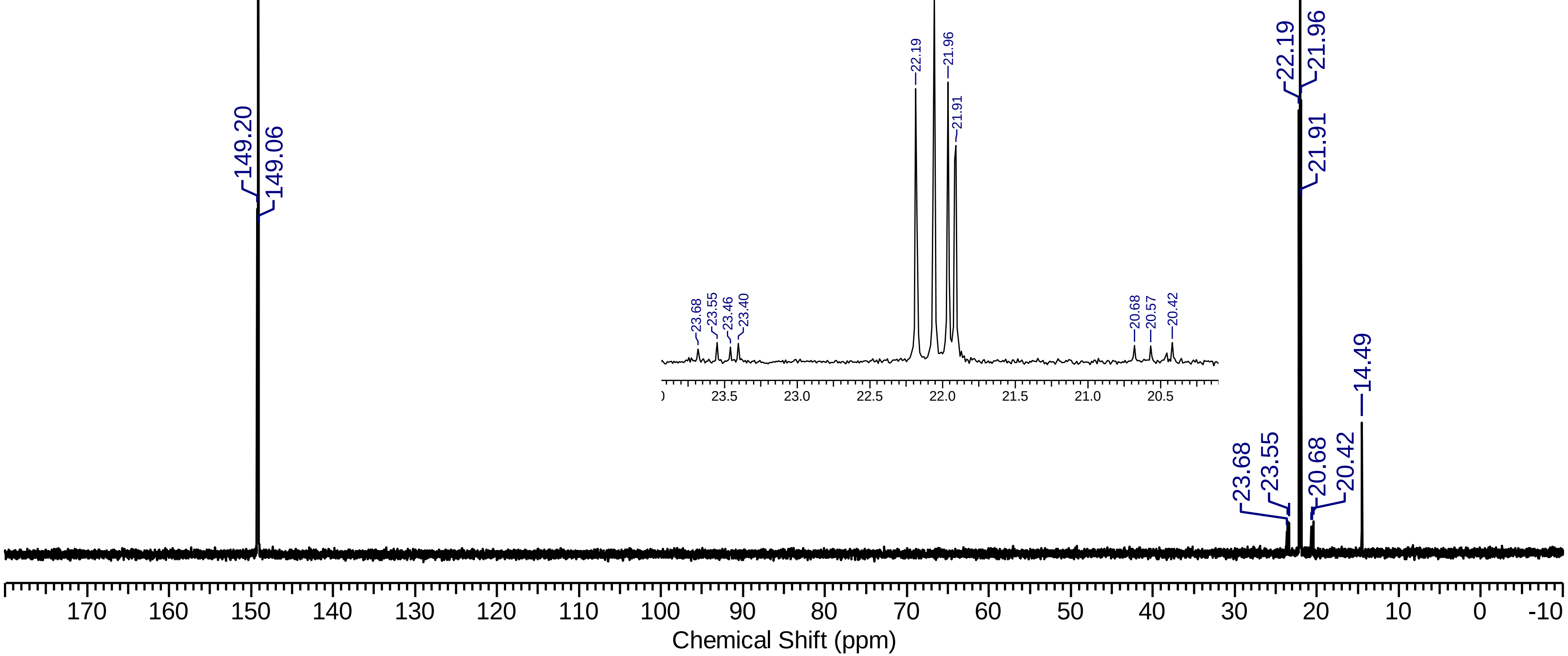

S 47. DMTrT*p*SedT-Phosphoramidite (5d). ^{1}H NMR

(600 MHz, MeCN-d_3)

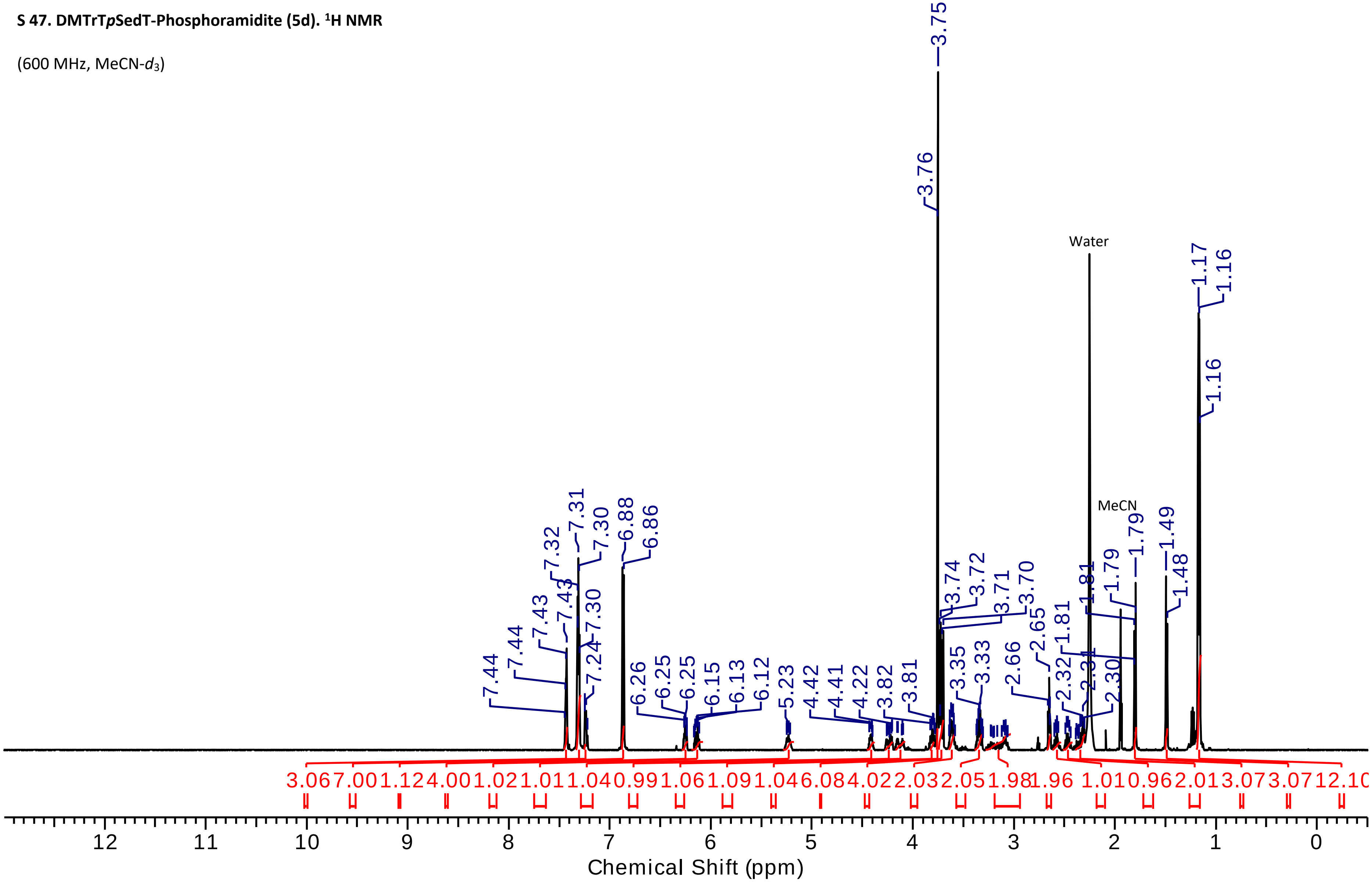

S 48. DMTrT*p*SedT-Phosphoramidite (5d). ES Mass spec

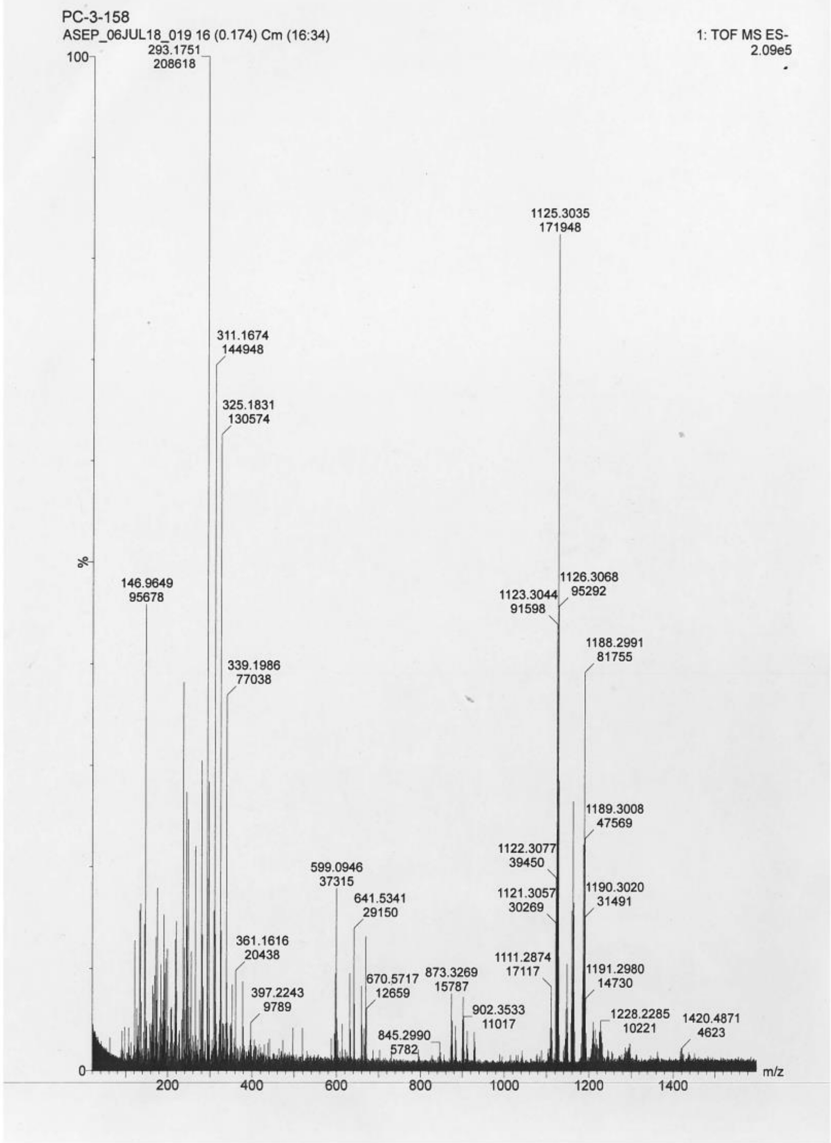

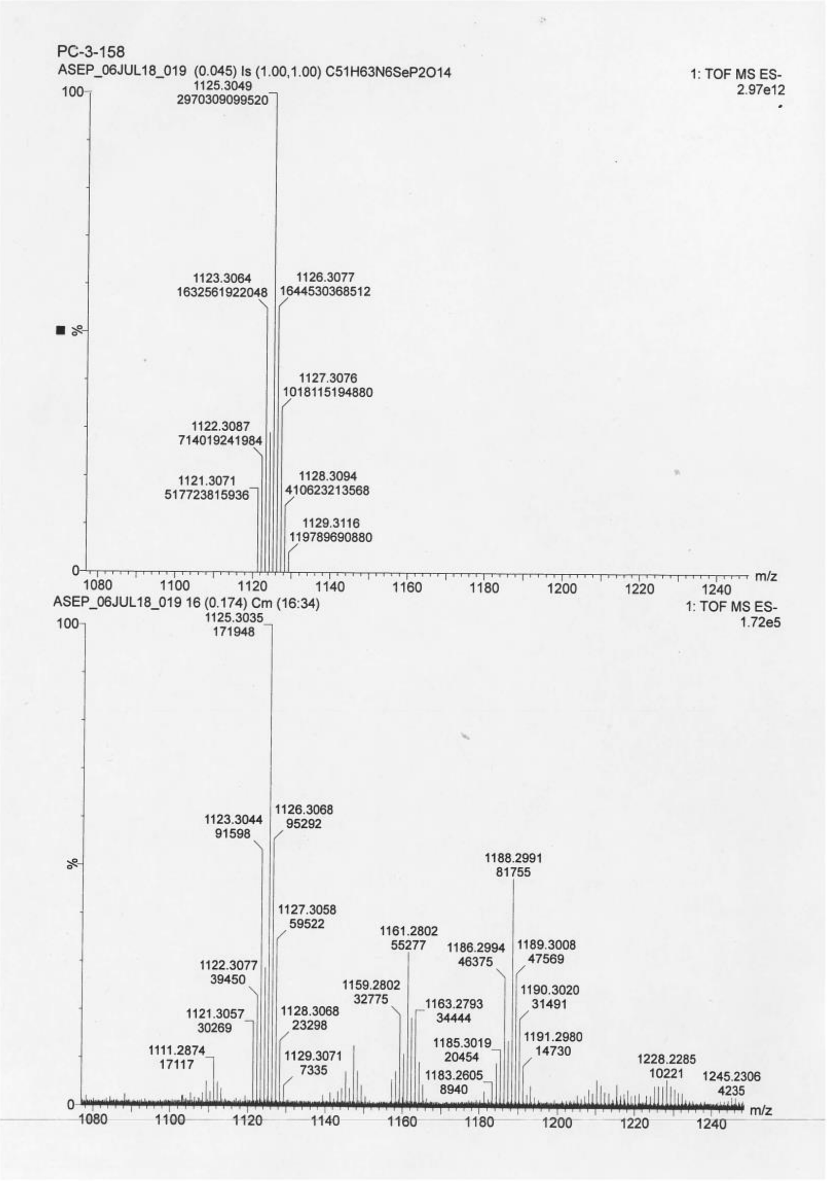

S 49. 5′-deoxythymidine-5′-selenocyanate-3′-O-succinate (6). ^{1}H NMR

(600 MHz, DMSO-d_6)

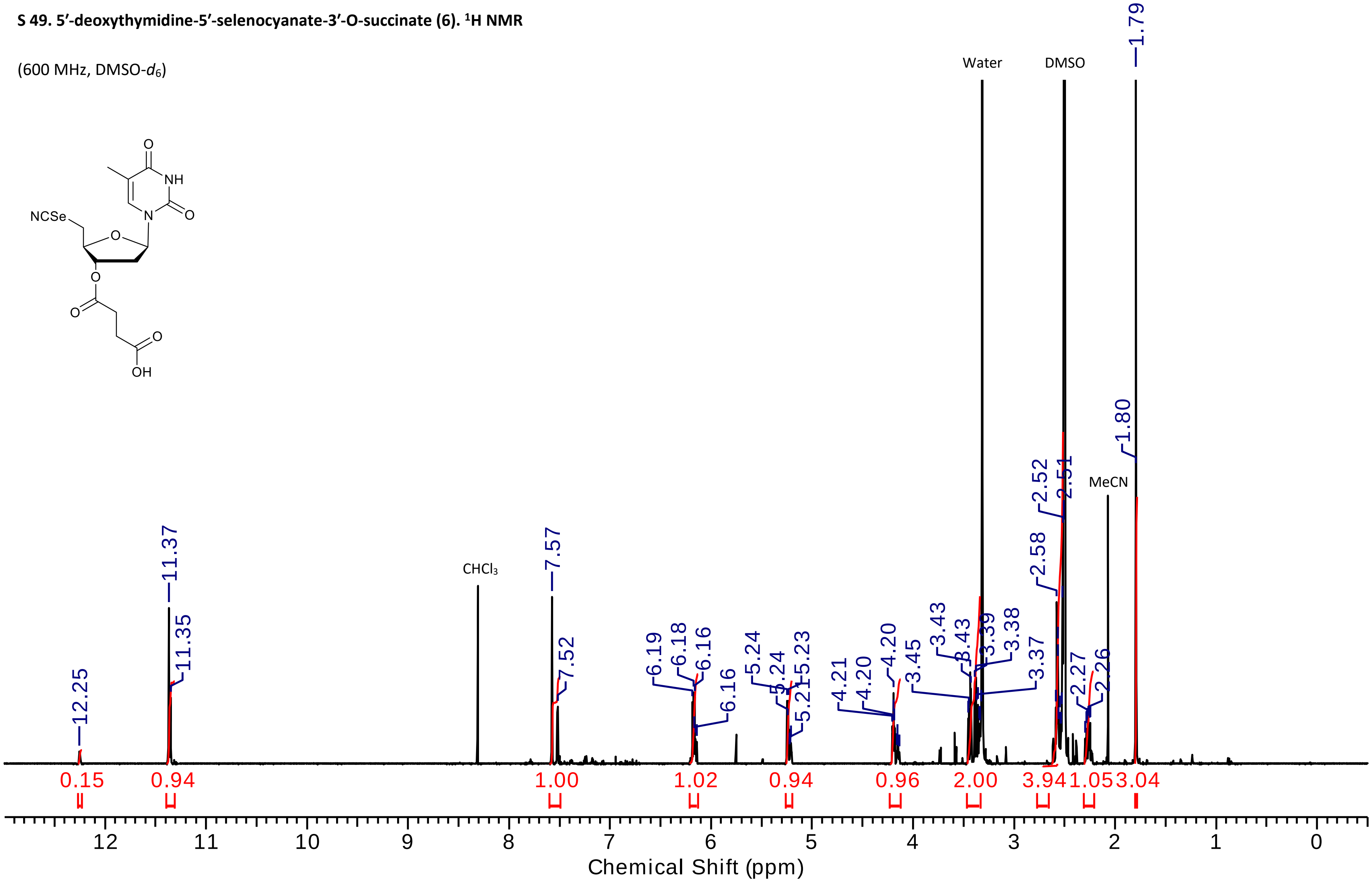

S 50. 5′-deoxythymidine-5′-selenocyanate-3′-O-succinate (6). $^{77}Se\{^{1}H\}$ NMR

(114 MHz, DMSO-d_6 with external 0.25M KSeCN / D_2O Insert)

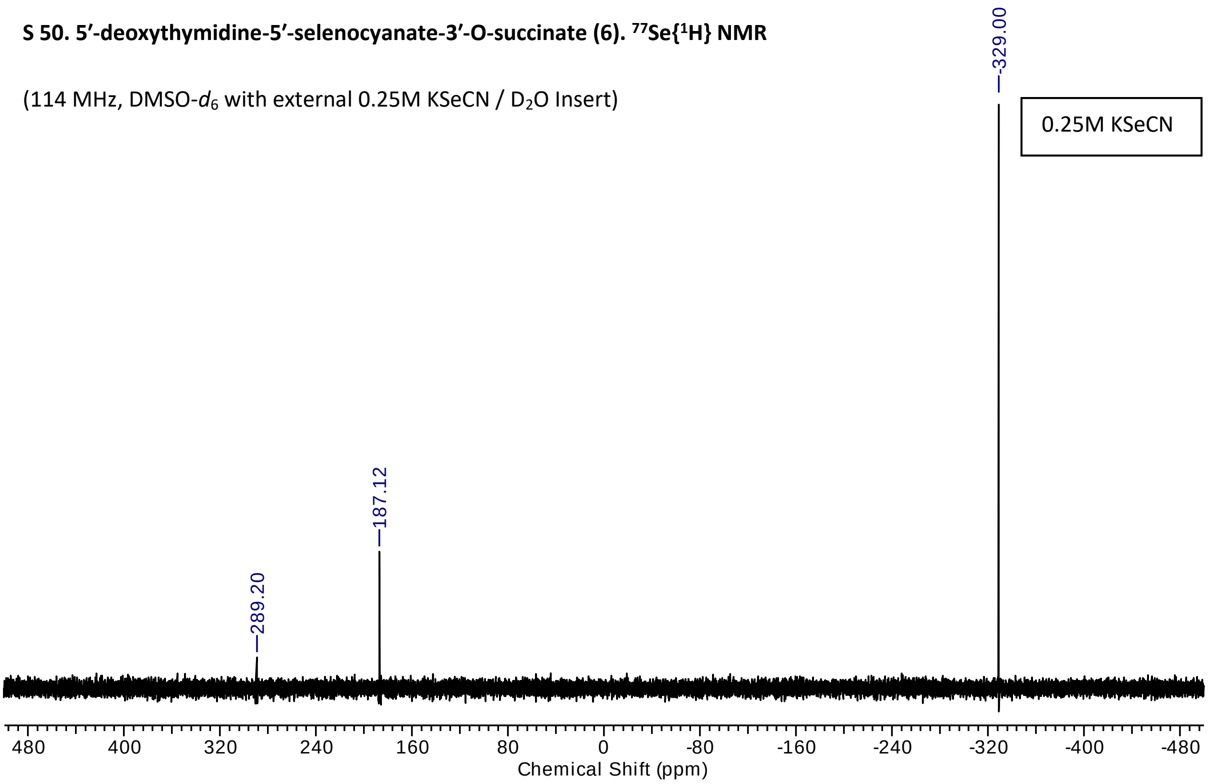

S 51. 5′-deoxythymidine-5′-selenocyanate-3′-O-succinate (6). ES Mass spec

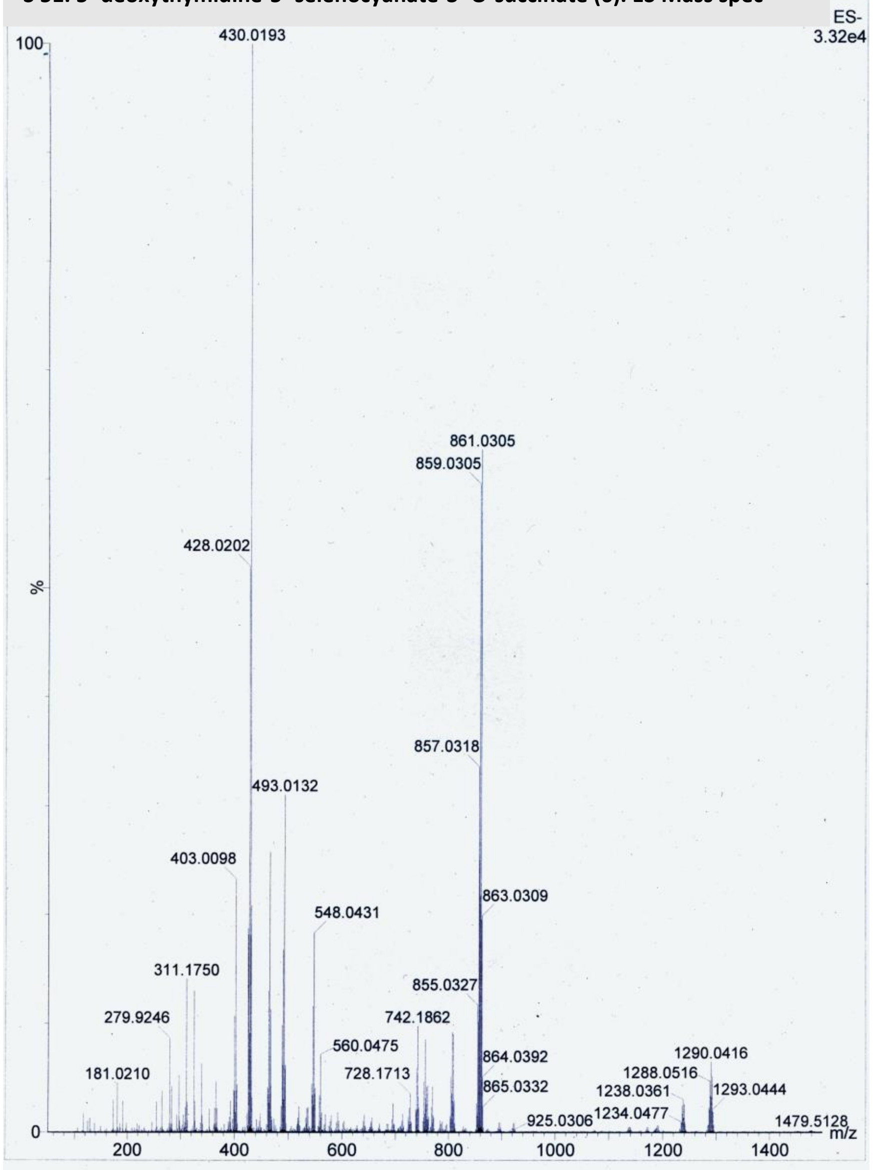

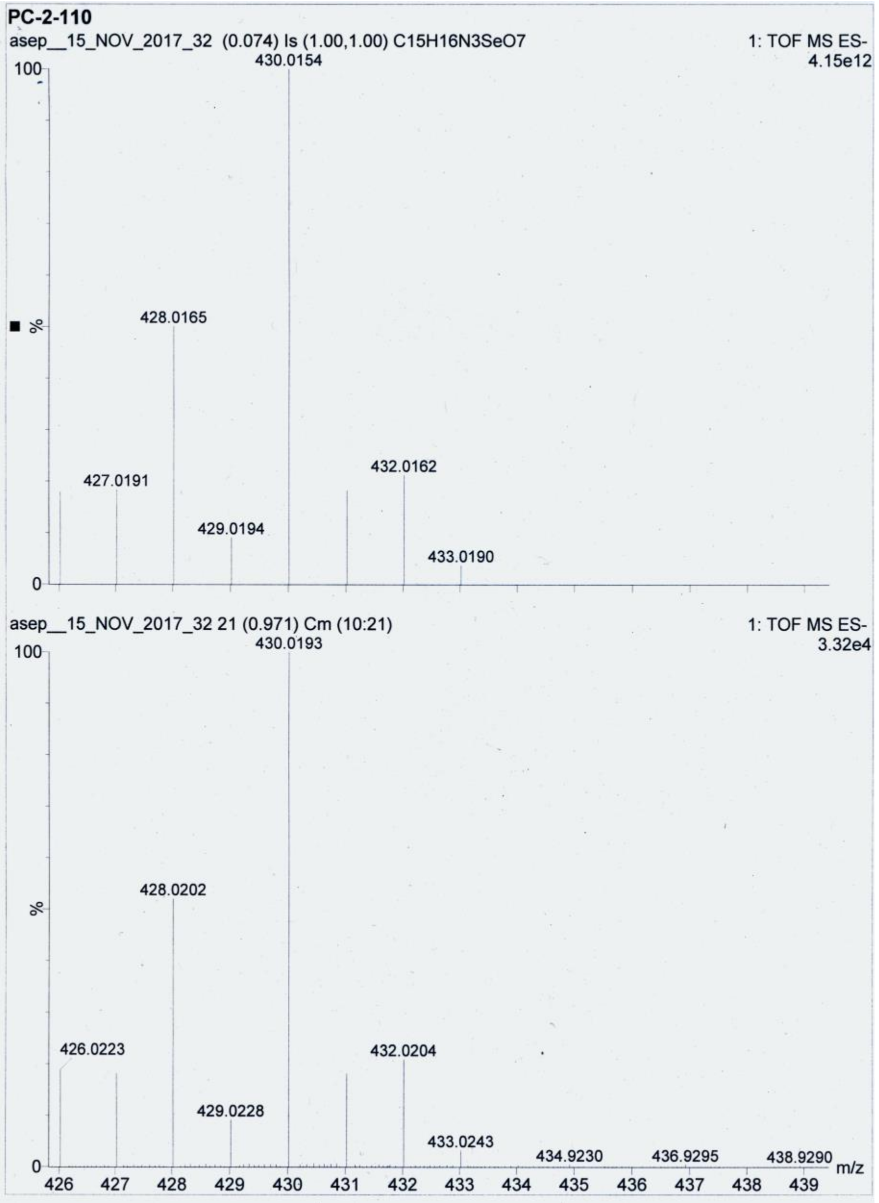

S 52. d(TSeTTSeTT) (ODN 1). $^{31}P\{^1H\}$ NMR

(243 MHz, 40% D_2O / H_2O)

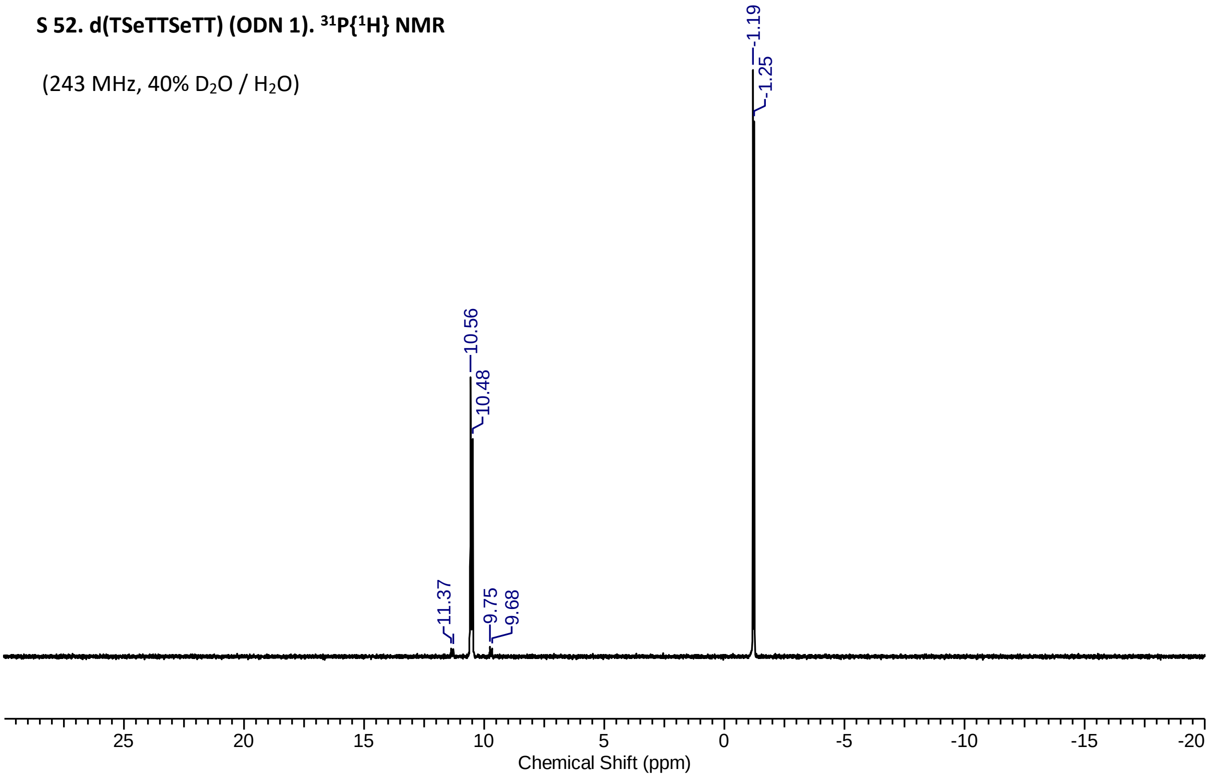

S 53. d(TSeTTSeTT) (ODN 1) RP-HPLC

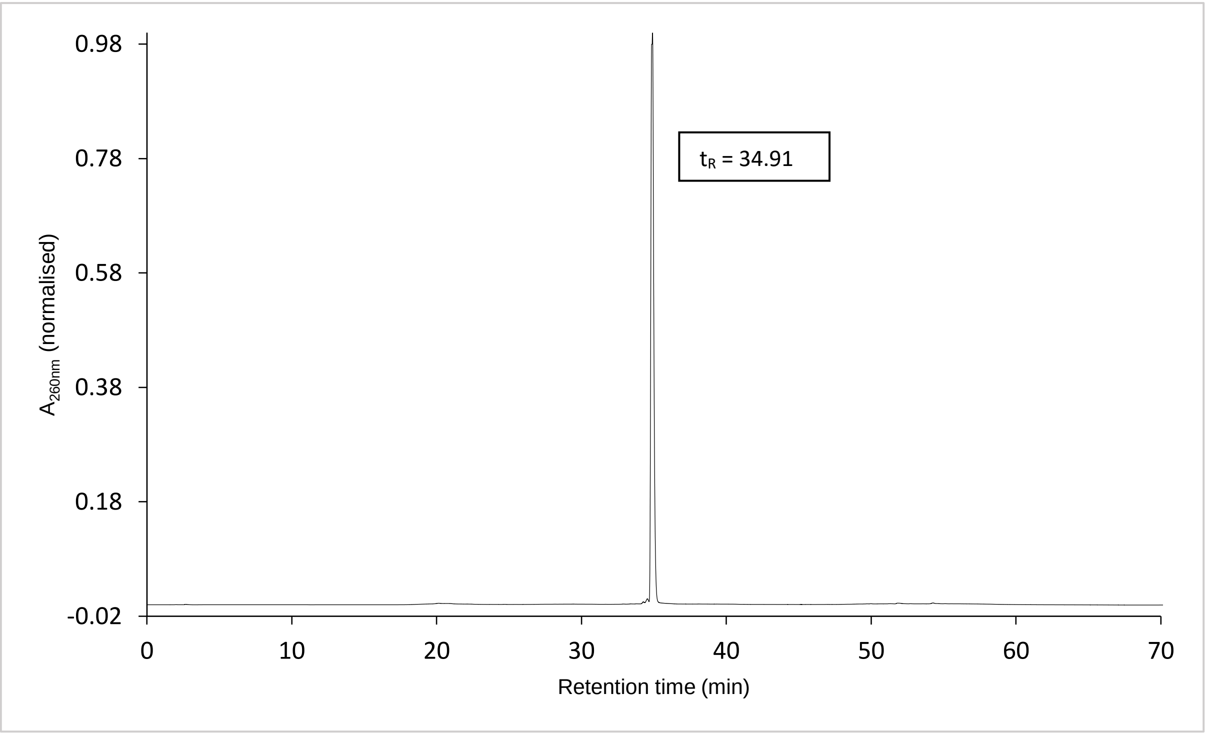

S 54. d(TSeTTSeT) (ODN 1) ES Mass spec

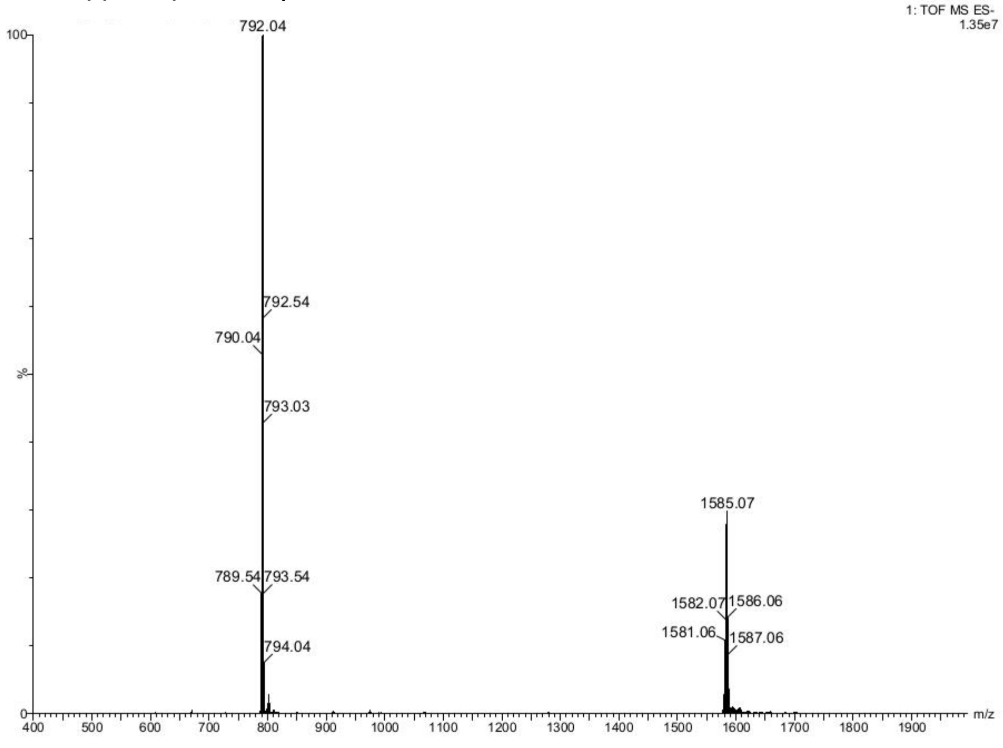

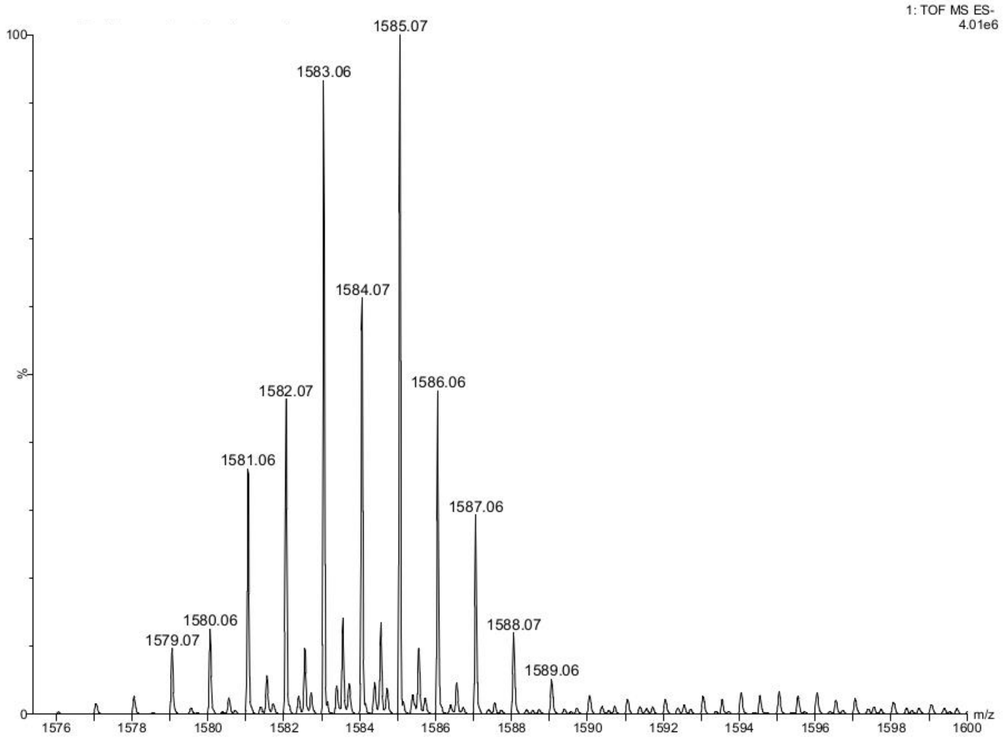

S 55. d(ASeTCCCGGGAT) (ODN 2) RP-HPLC

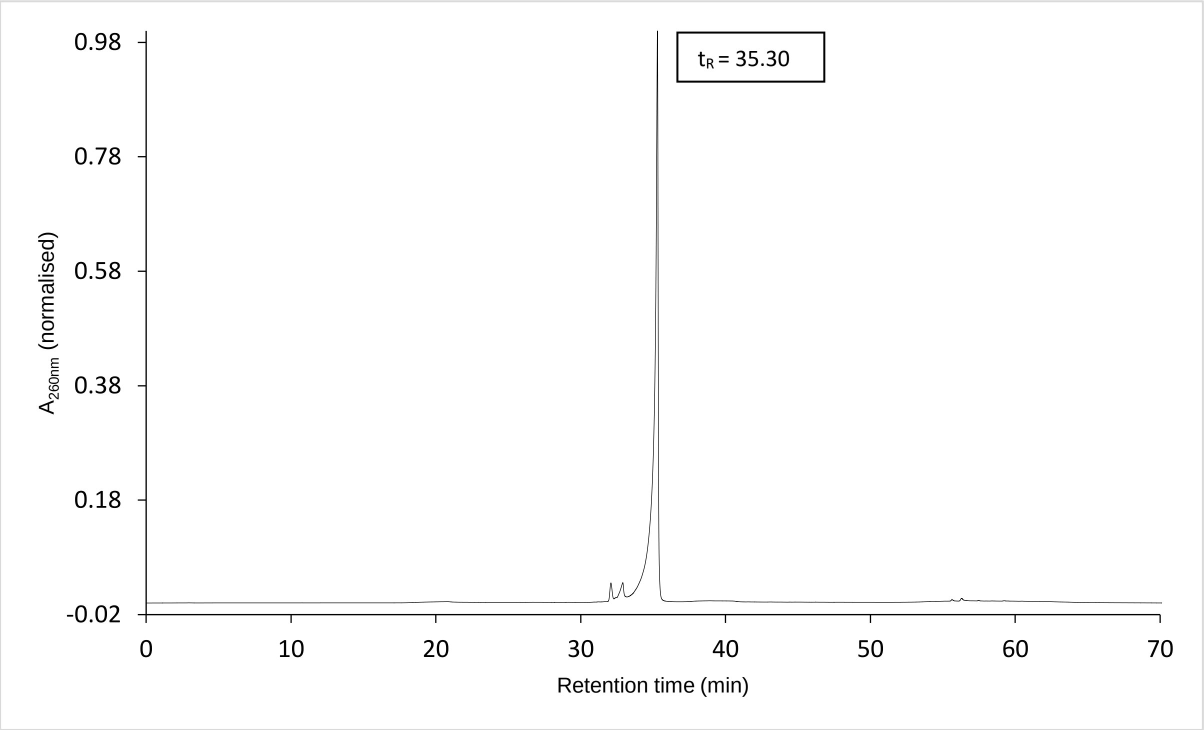

S 56. d(ASeTCCCGGGAT) (ODN 2) ES Mass spec

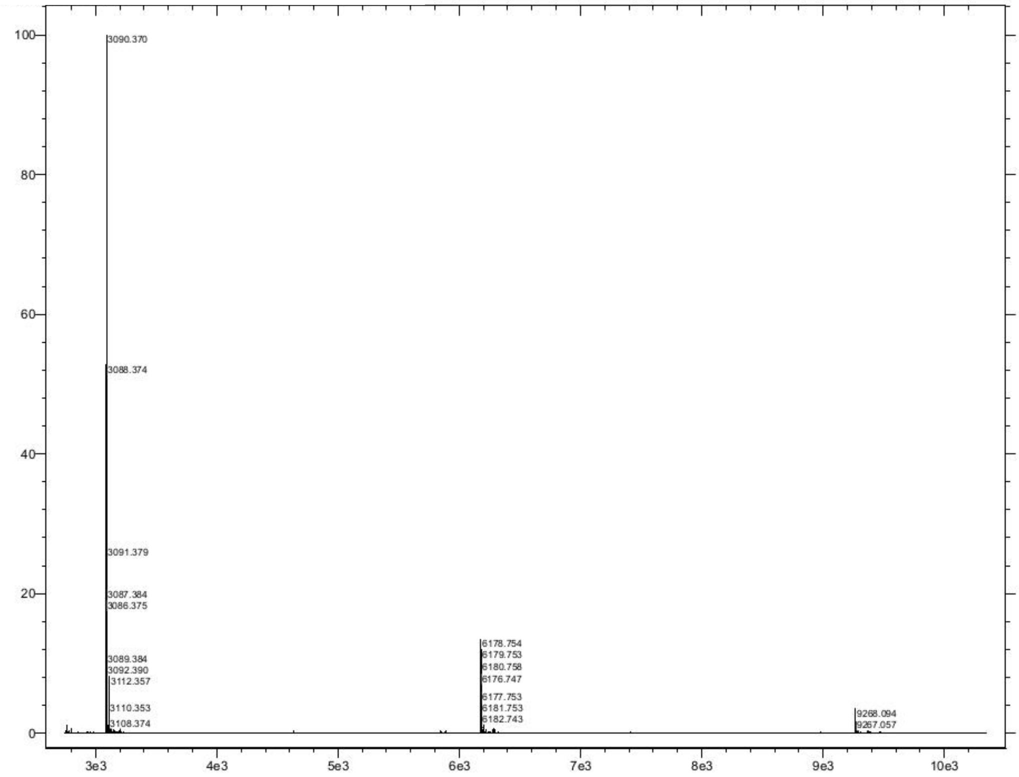

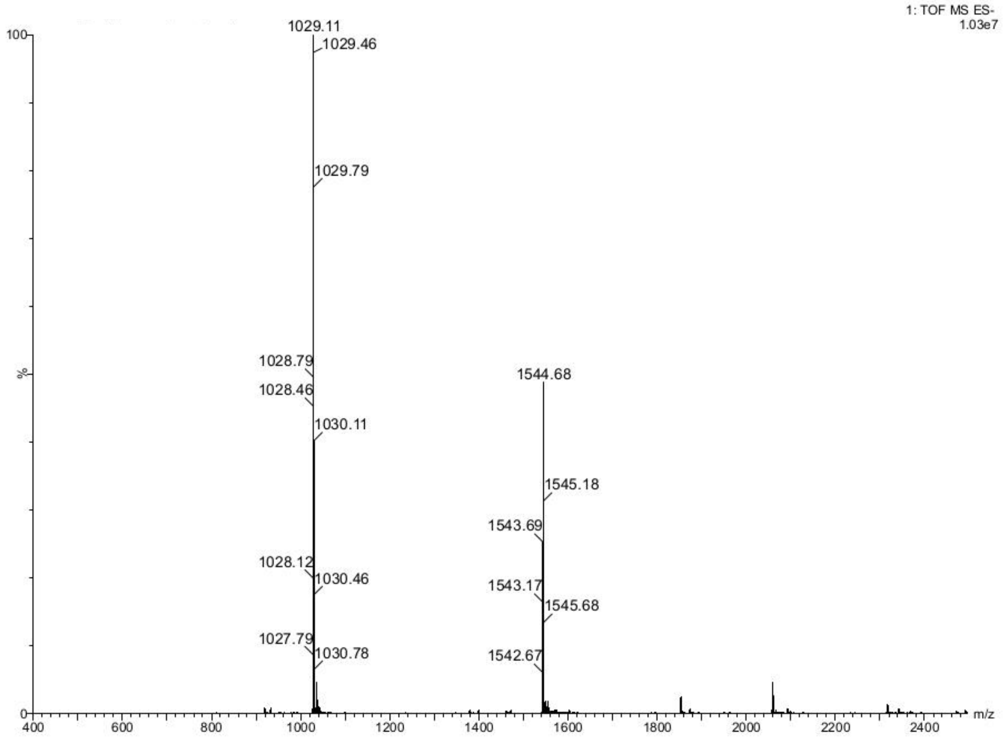
1: TOF MS ES-
1.03e7
100
%
0
1029.11
1029.46
1029.79
1028.79
1028.46
1030.11
1028.12
1030.46
1027.79
1030.78
1544.68
1545.18
1543.69
1543.17
1545.68
1542.67
400
600
800
1000
1200
1400
1600
1800
2000
2200
2400
m/z

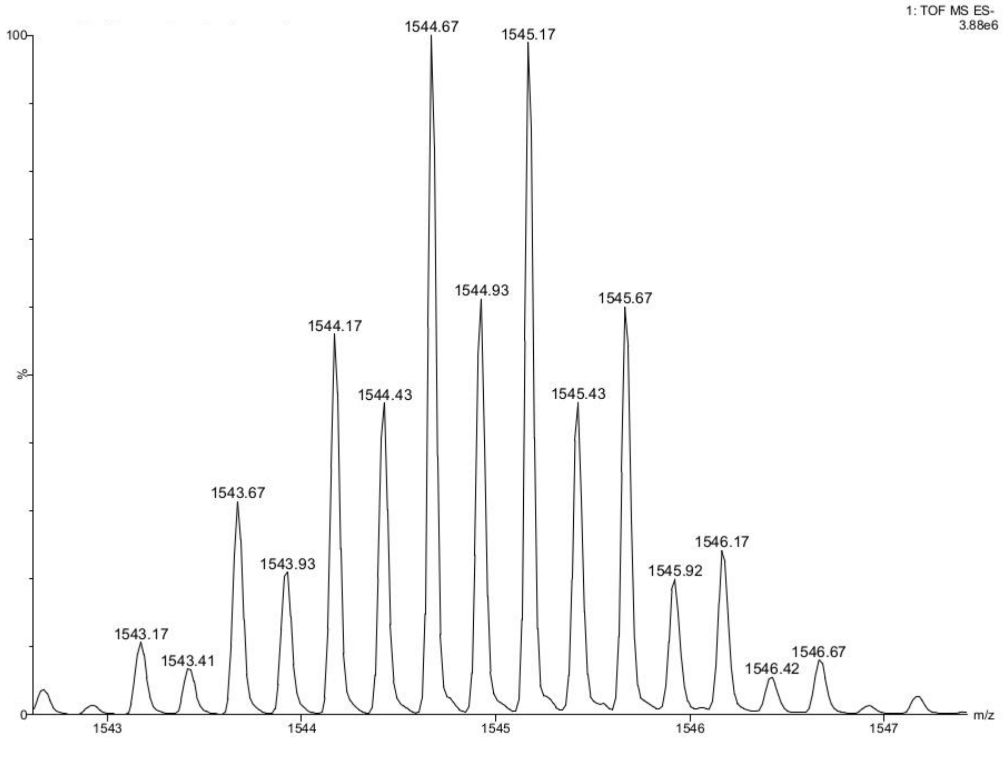
1: TOF MS ES-
3.88e6
100
%
0
1544.67
1545.17
1544.93
1545.67
1544.17
1544.43
1545.43
1543.67
1546.17
1543.93
1545.92
1543.17
1543.41
1546.67
1546.42
1543
1544
1545
1546
1547
m/z

S 57. d(CSeTCCCGGGAG) (ODN 3) RP-HPLC

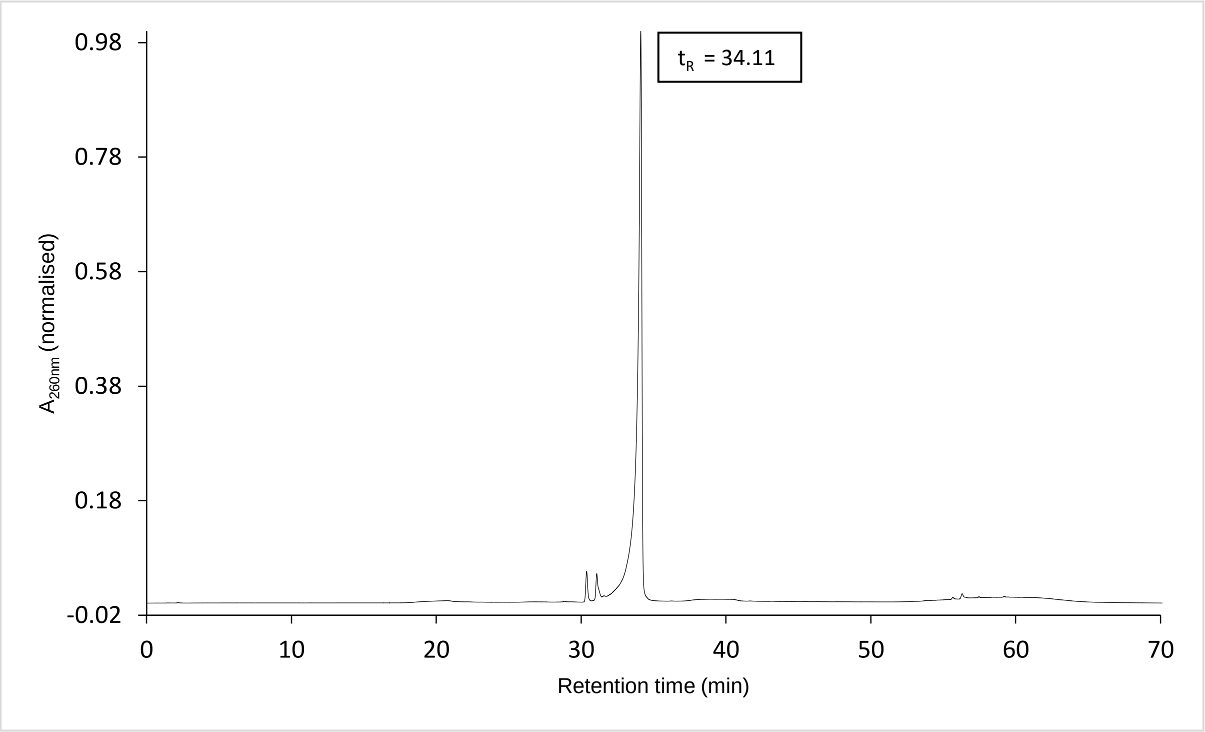

S 58. d(CSeTCCCGGGAG) (ODN 3) ES Mass spec

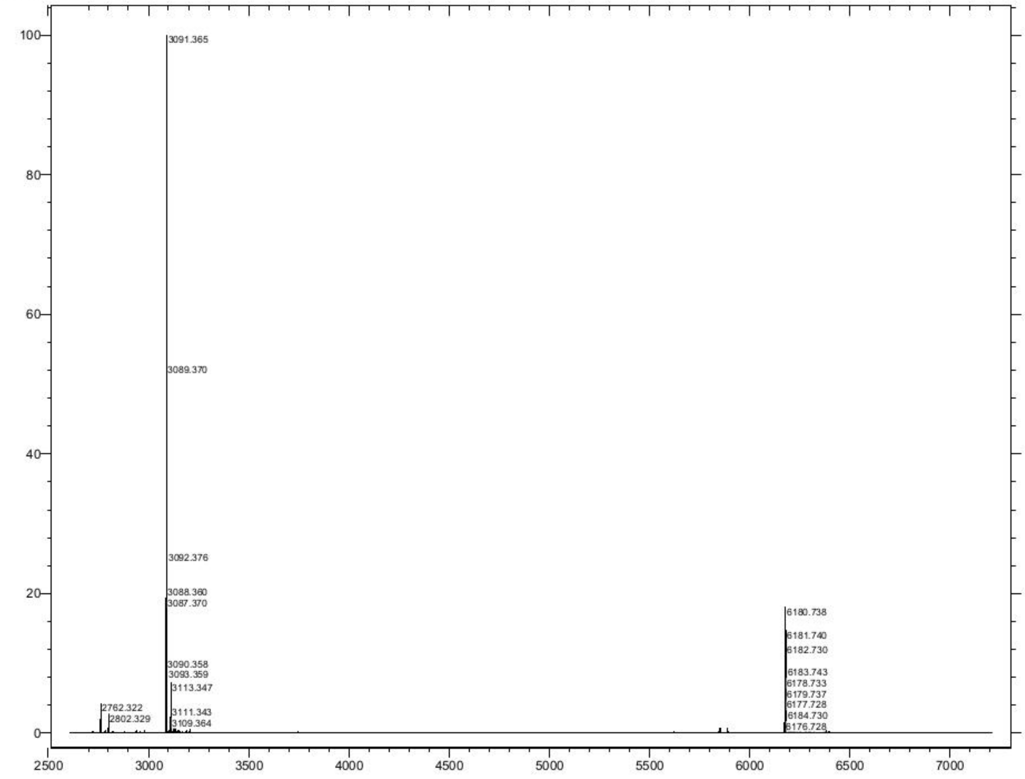

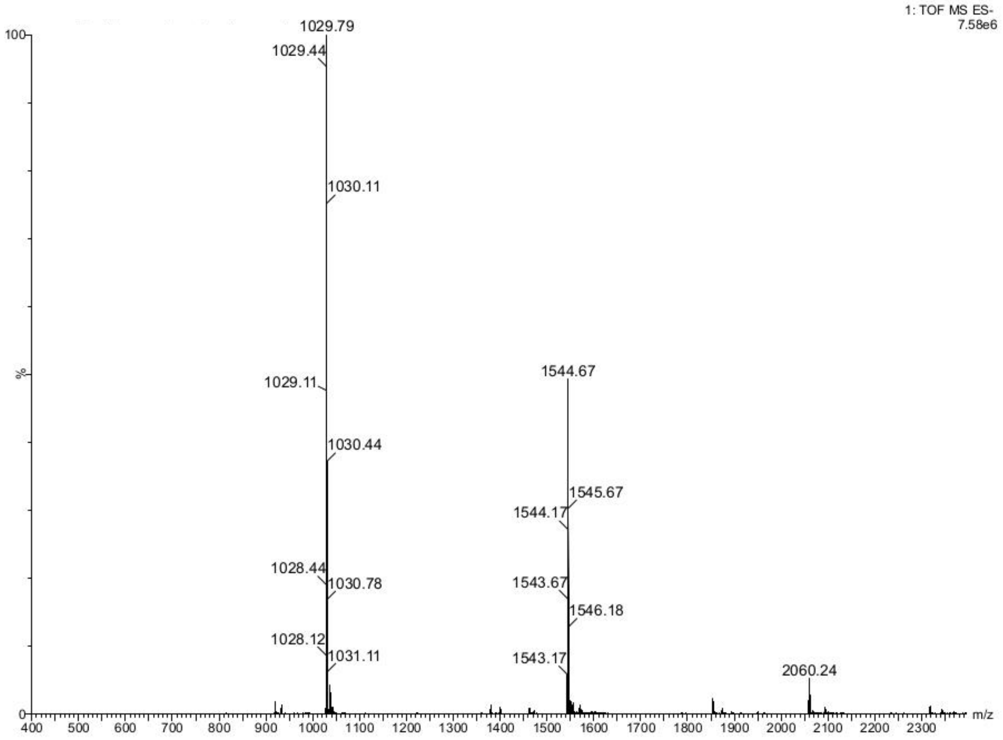
1: TOF MS ES-
7.58e6
1029.79
1029.44
1030.11
1029.11
1030.44
1028.44
1030.78
1028.12
1031.11
1544.67
1545.67
1544.17
1543.67
1546.18
1543.17
2060.24
m/z
%

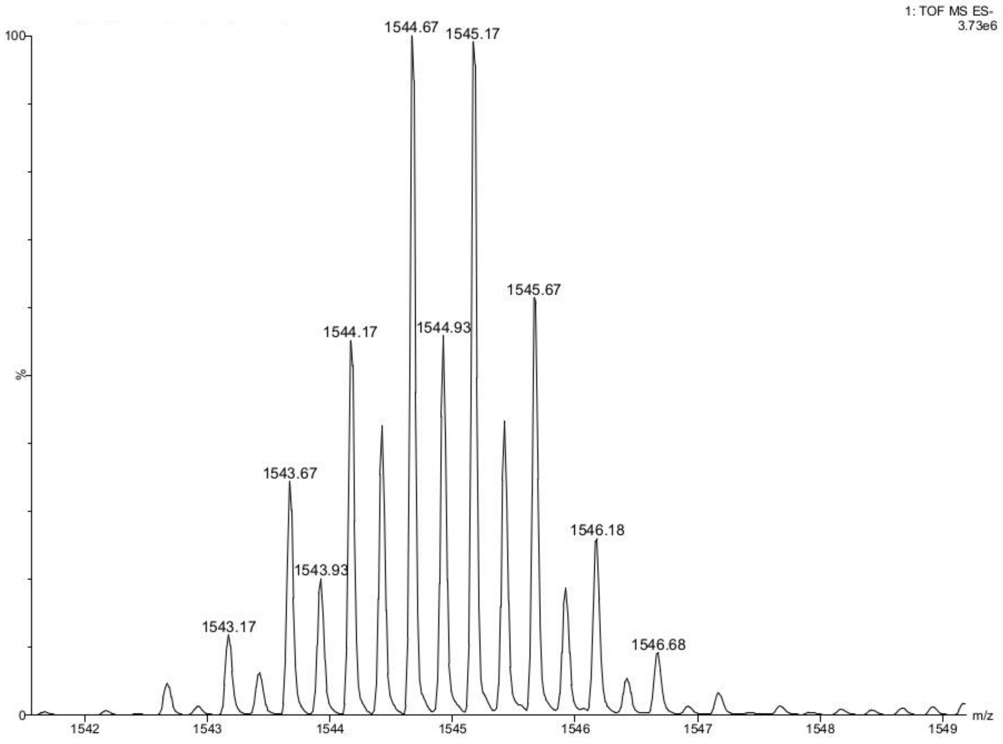
1: TOF MS ES-
3.73e6
1544.67
1545.17
1545.67
1544.17
1544.93
1543.67
1546.18
1543.93
1543.17
1546.68
m/z
%

S 59. d(GSeTCCCGGGAC) (ODN 4) RP-HPLC

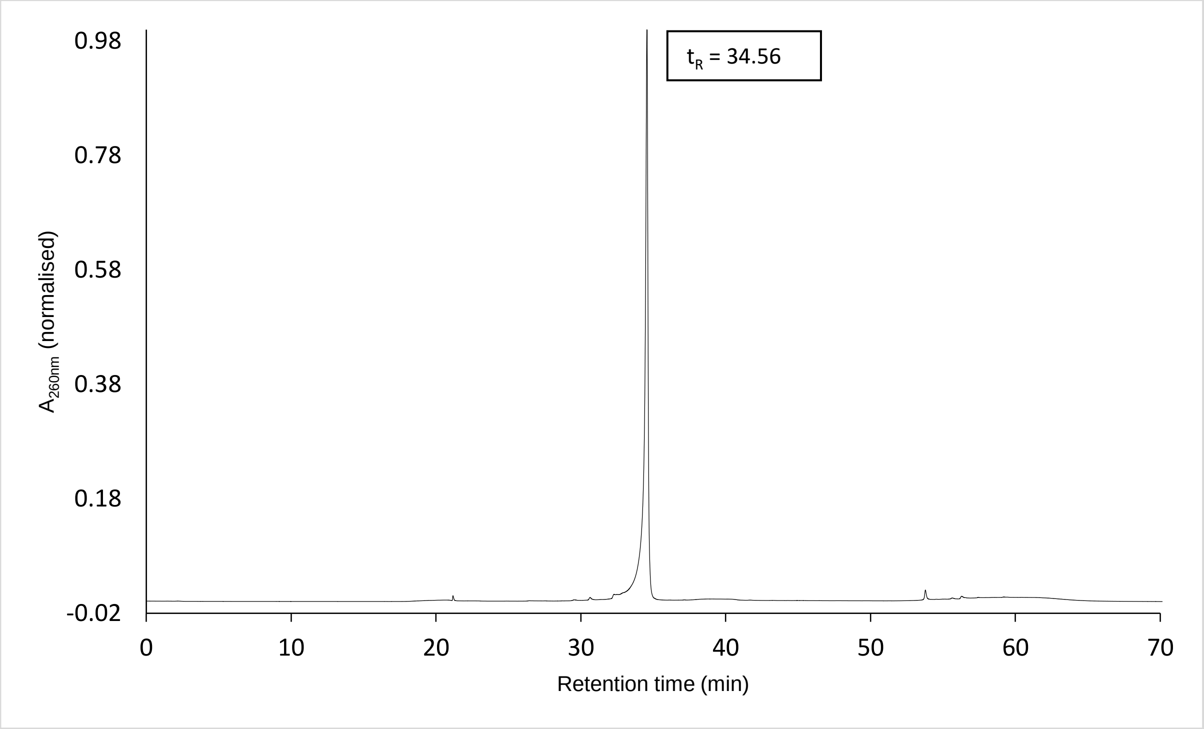

S 60. d(GSeTCCCGGGAC) (ODN 4) ES Mass spec

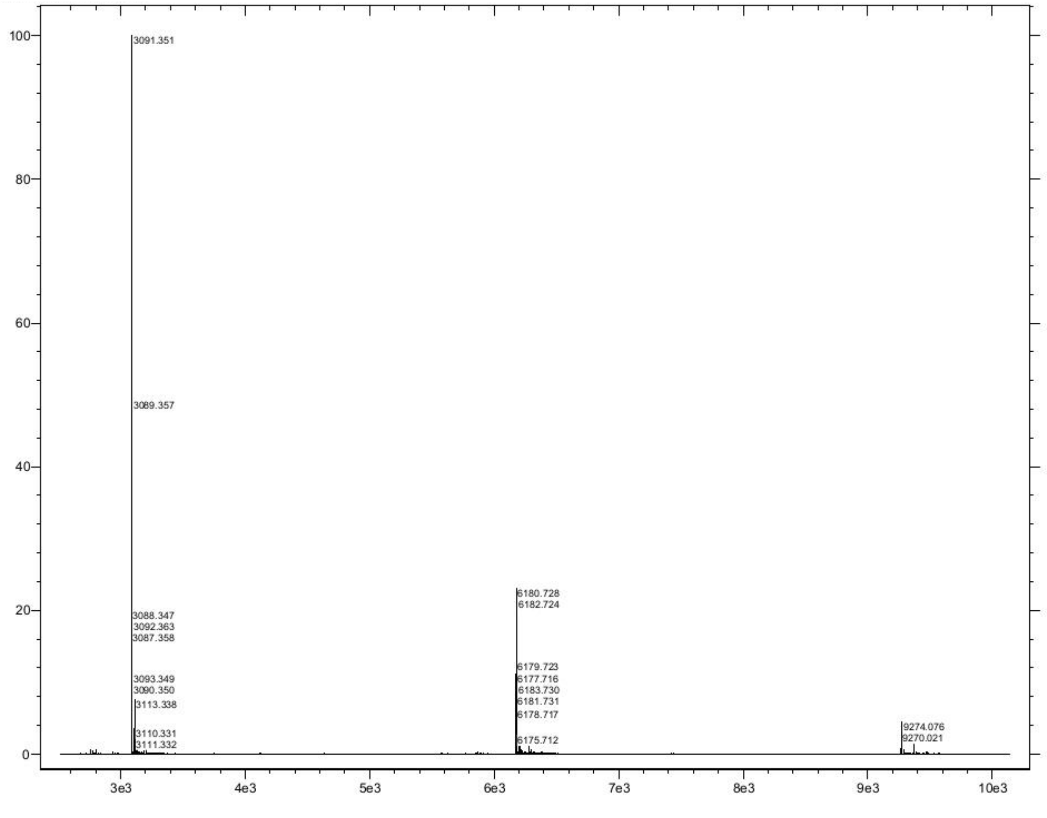

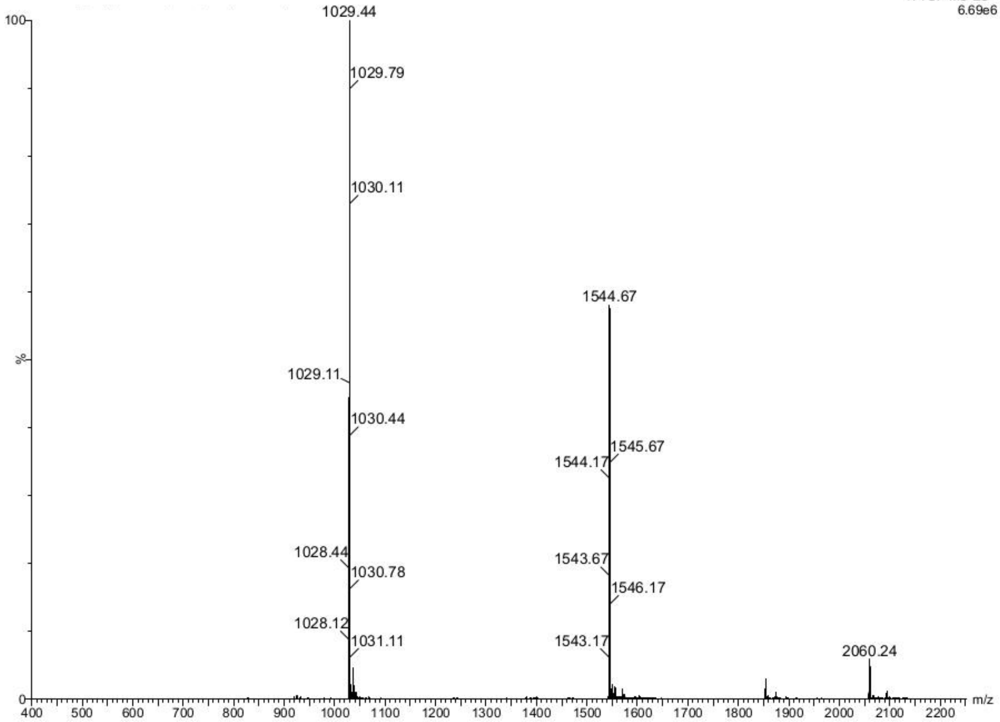
1: TOF MS ES-
6.69e6
1029.44
1029.79
1030.11
1544.67
1029.11
1030.44
1545.67
1544.17
1028.44
1543.67
1030.78
1546.17
1028.12
1031.11
1543.17
2060.24
%
m/z

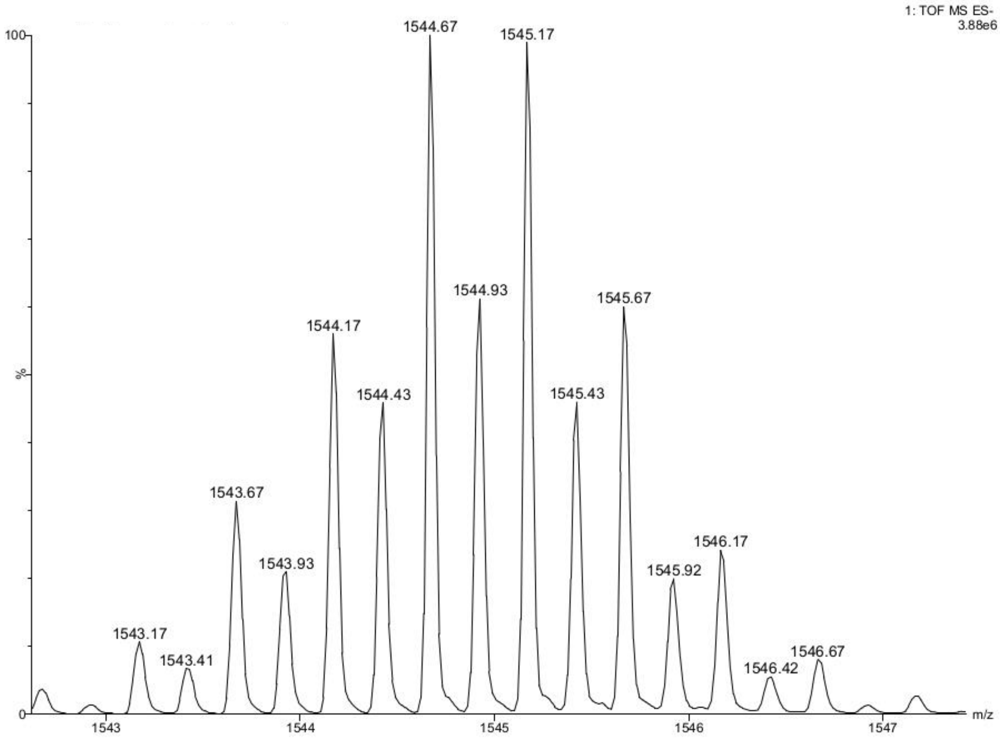
1: TOF MS ES-
3.88e6
1544.67
1545.17
1544.93
1545.67
1544.17
1544.43
1545.43
1543.67
1546.17
1543.93
1545.92
1543.17
1546.67
1543.41
1546.42
%
m/z

S 61. d(TSeTCCCGGGAA) (ODN 5) RP-HPLC

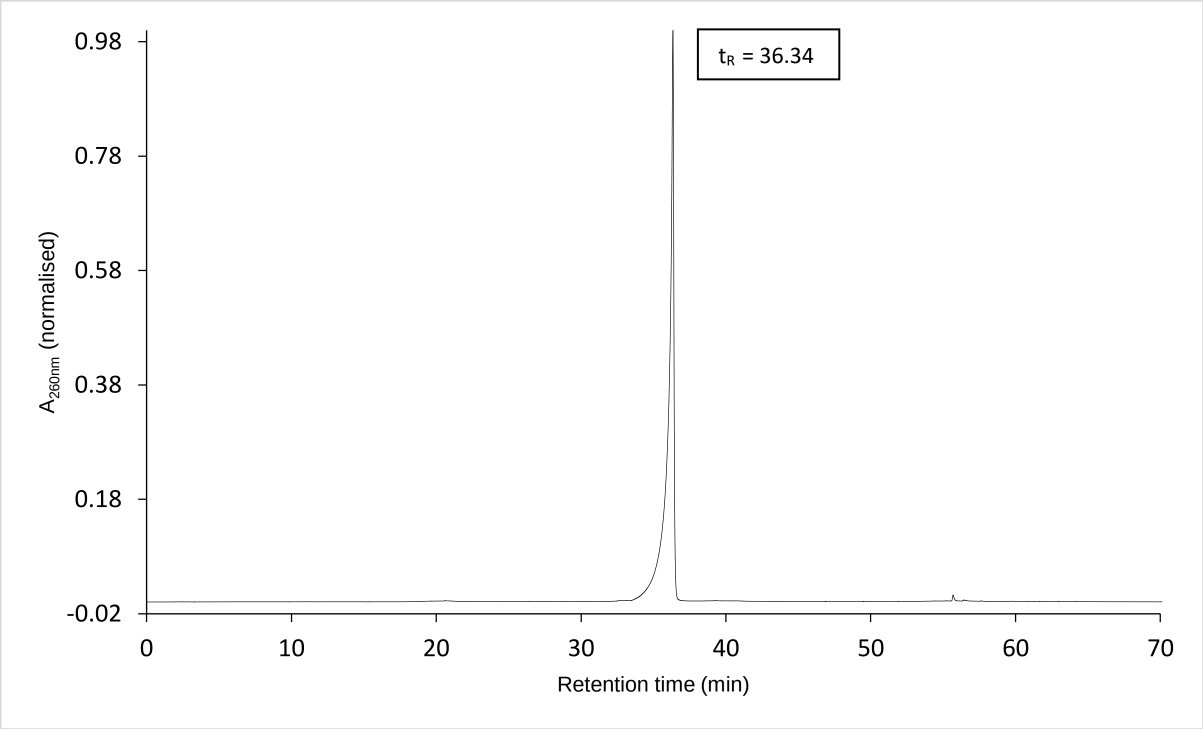

S 62. d(TSeTCCCGGGAA) (ODN 5) ES Mass spec

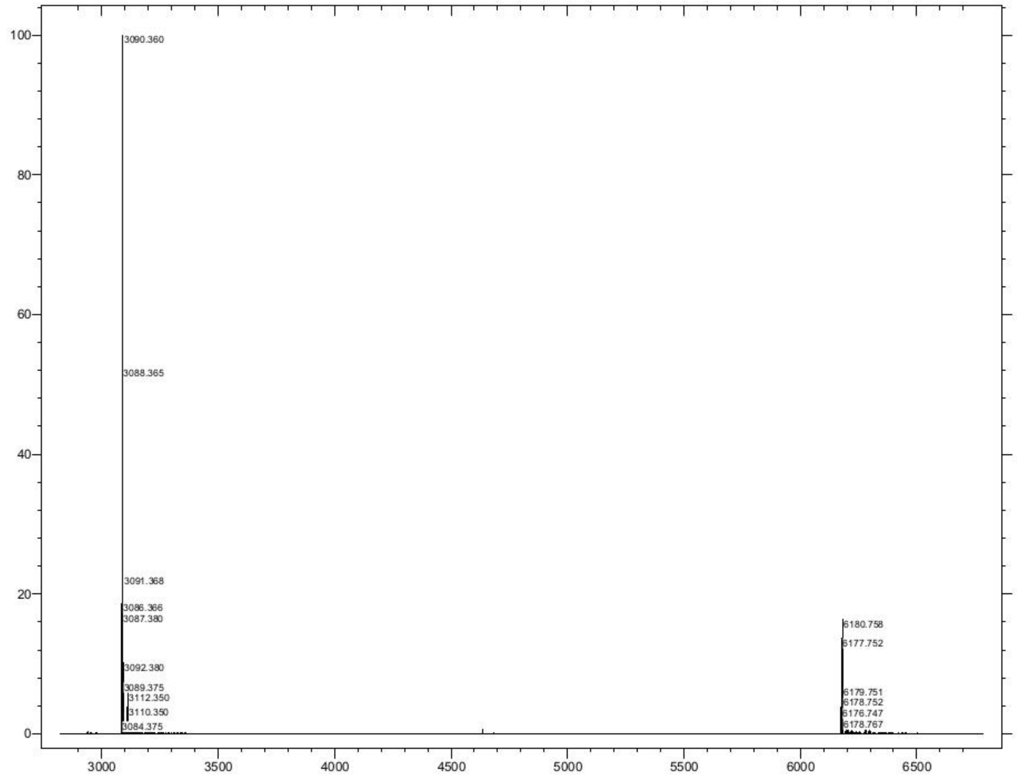

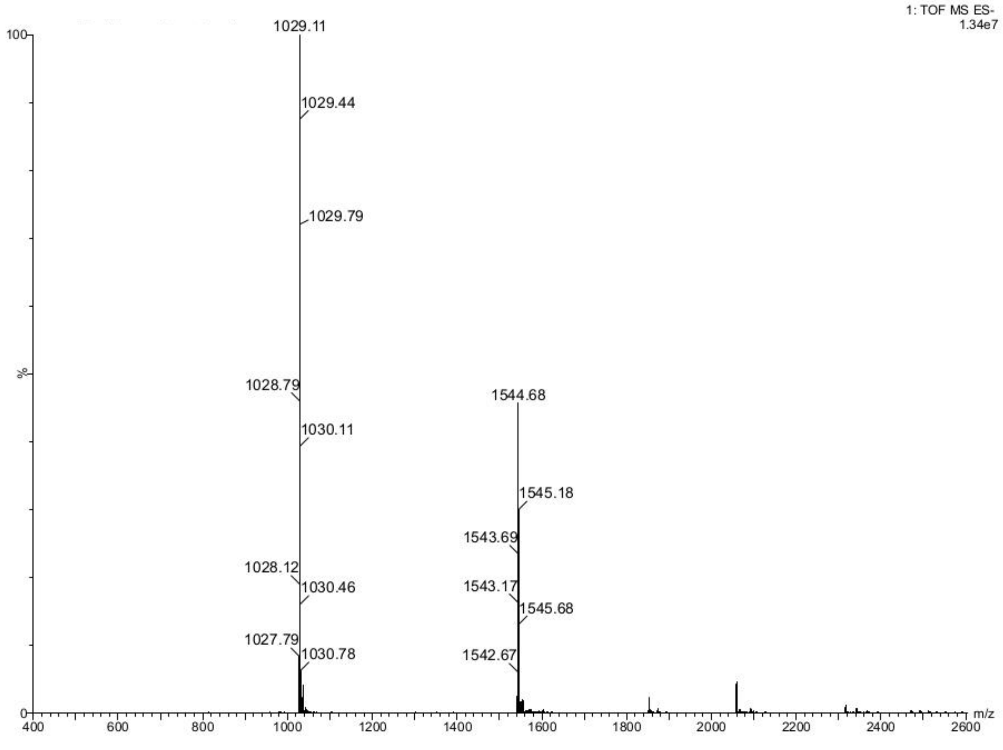
1: TOF MS ES-
1.34e7
1029.11
1029.44
1029.79
1028.79
1030.11
1028.12
1030.46
1027.79
1030.78
1544.68
1545.18
1543.69
1543.17
1545.68
1542.67
%
m/z

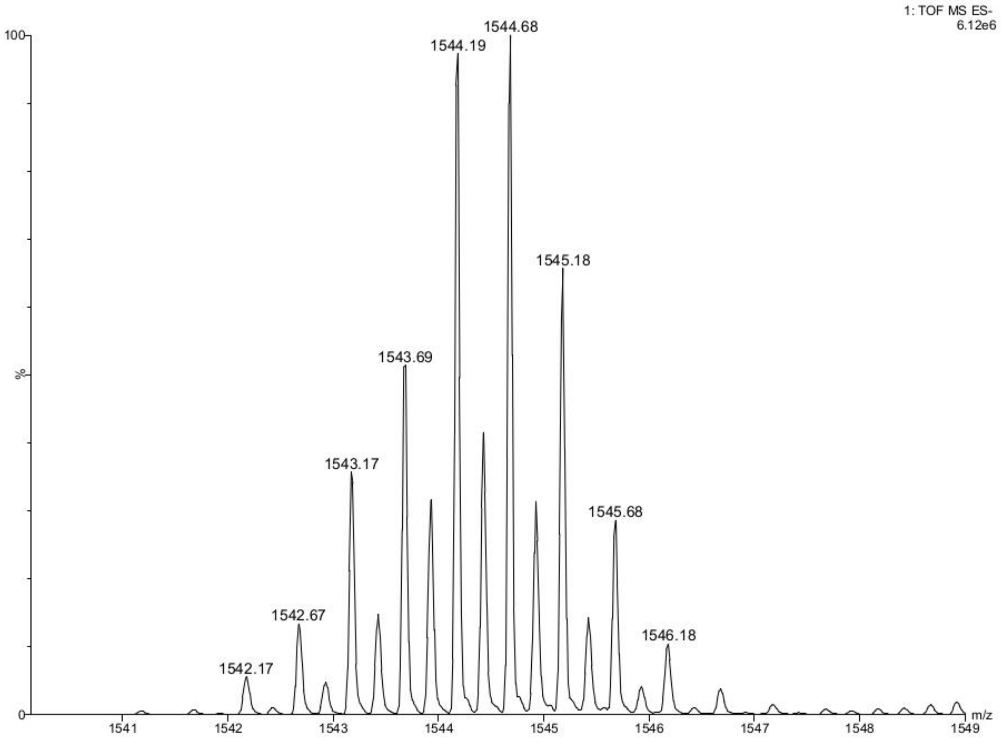
1: TOF MS ES-
6.12e6
1544.19
1544.68
1545.18
1543.69
1543.17
1545.68
1542.67
1546.18
1542.17
%
m/z

S 63. d(CGCGAASeTTCGCG) (ODN 6) 52 °C RP-HPLC

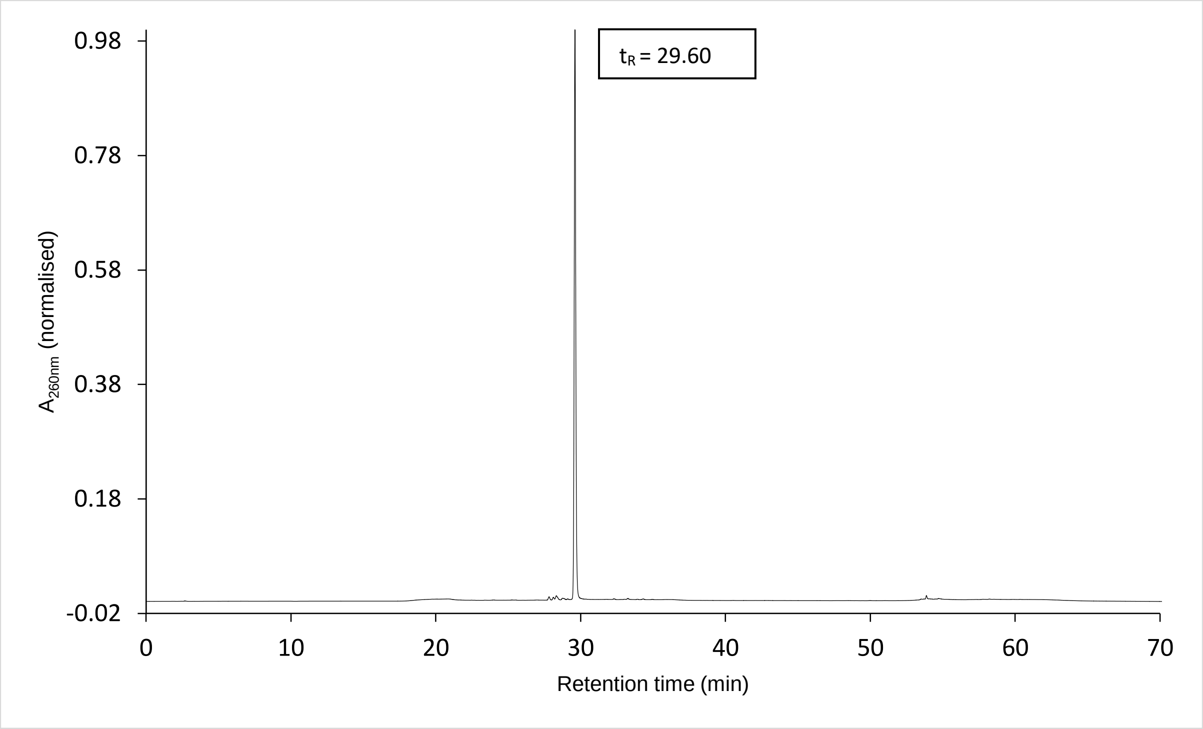

S 64. d(CGCGAASeTTCGCG) (ODN 6) ES Mass spec

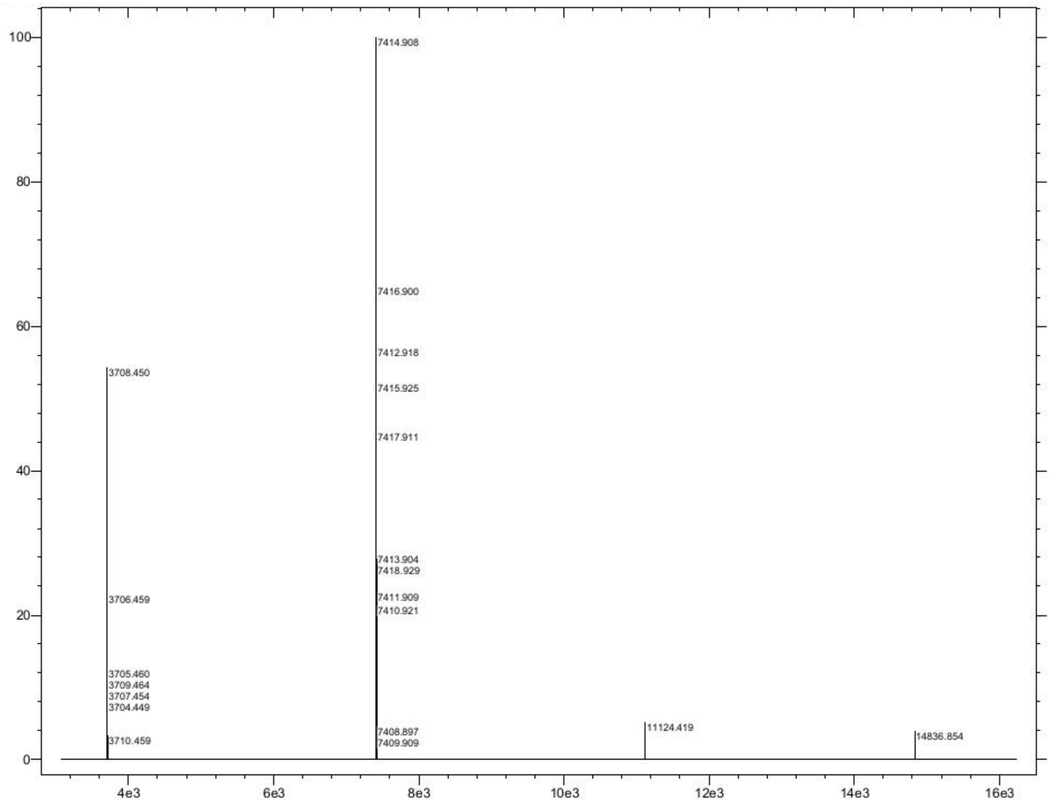

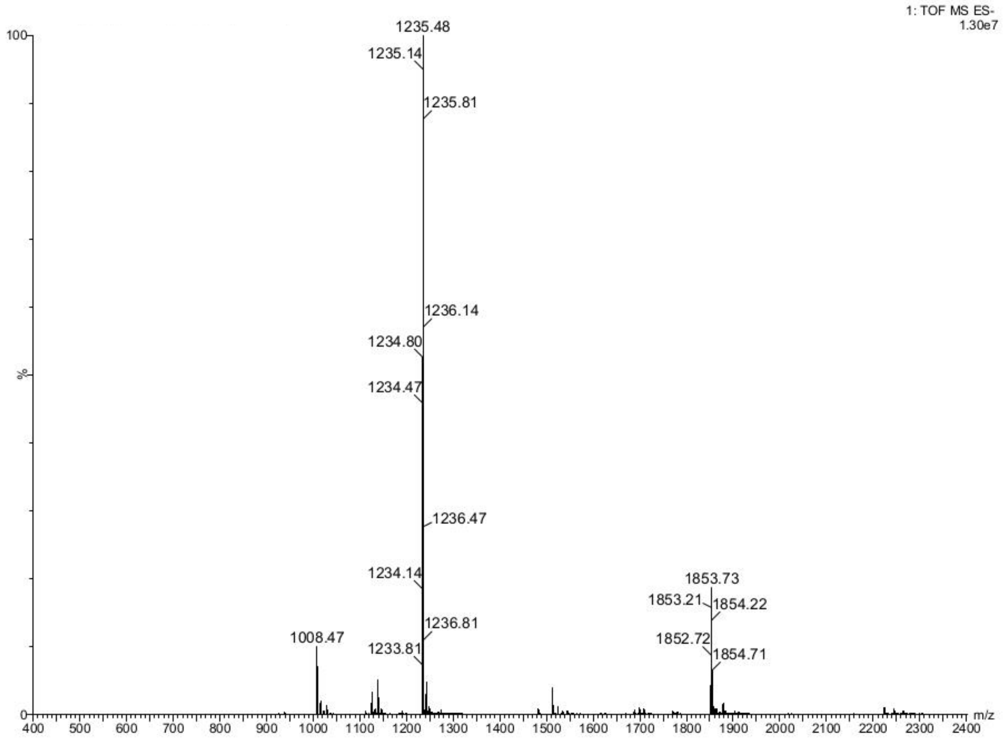
1: TOF MS ES-
1.30e7
1235.48
1235.14
1235.81
1236.14
1234.80
1234.47
1236.47
1234.14
1236.81
1233.81
1008.47
1853.73
1853.21
1854.22
1852.72
1854.71
%
m/z

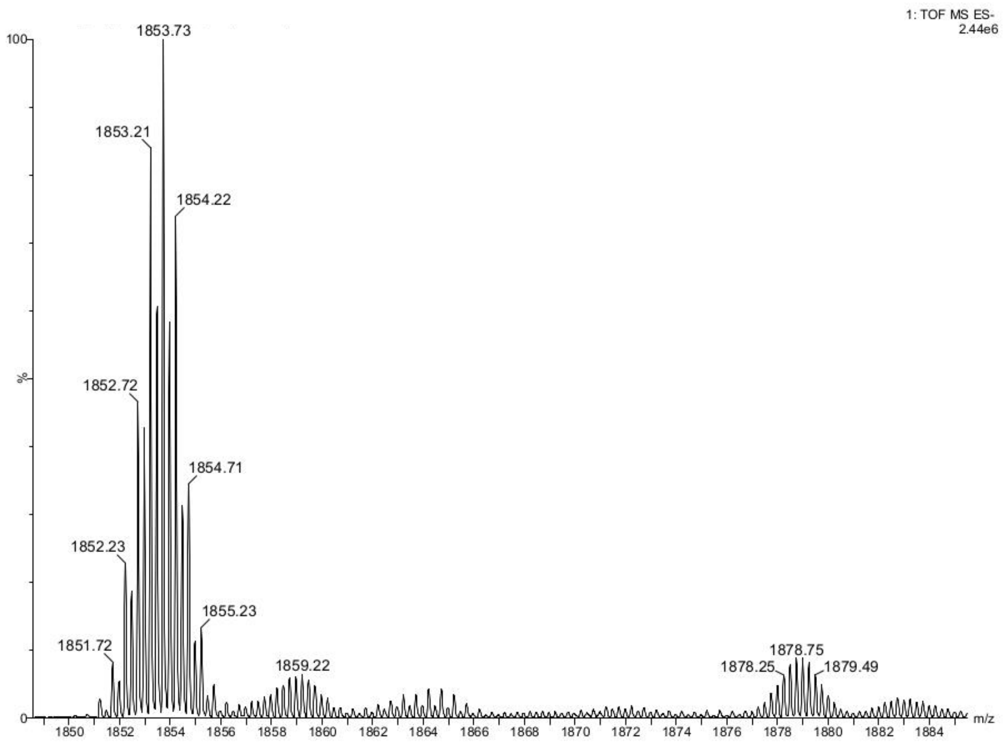
1: TOF MS ES-
2.44e6
1853.73
1853.21
1854.22
1852.72
1854.71
1852.23
1855.23
1851.72
1859.22
1878.75
1878.25
1879.49
%
m/z

S 65. d(CCCSeT) (ODN 7). $^{31}P\{^{1}H\}$ NMR

(243 MHz, 40% D_2O / H_2O)

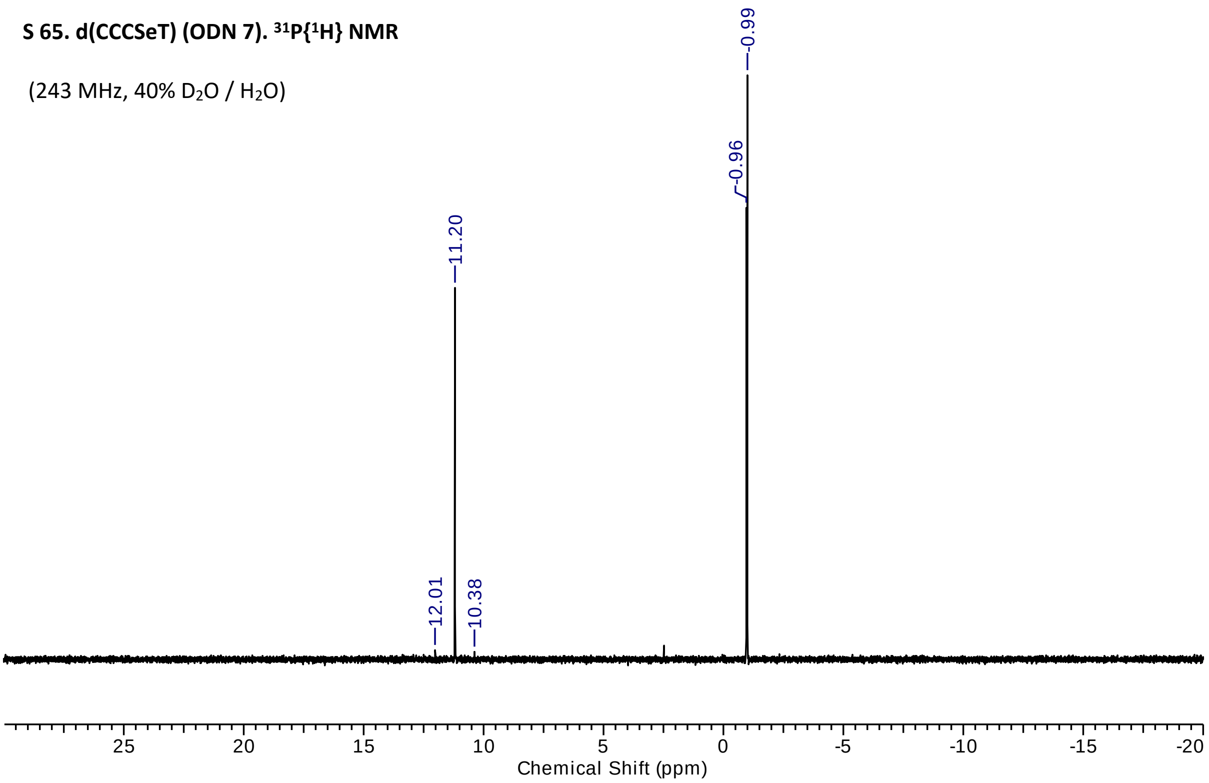

S 66. d(CCCSeT) (ODN 7) RP-HPLC

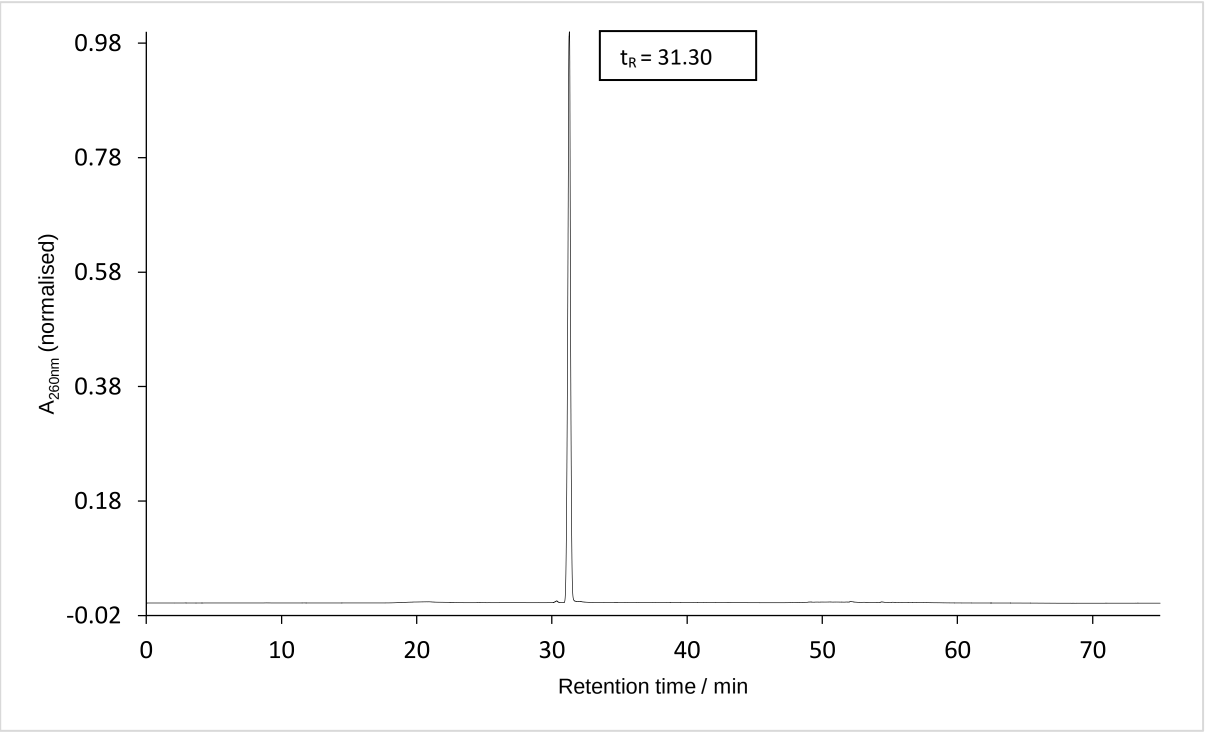

S 67. d(CCCSeT) (ODN 7) ES Mass spec

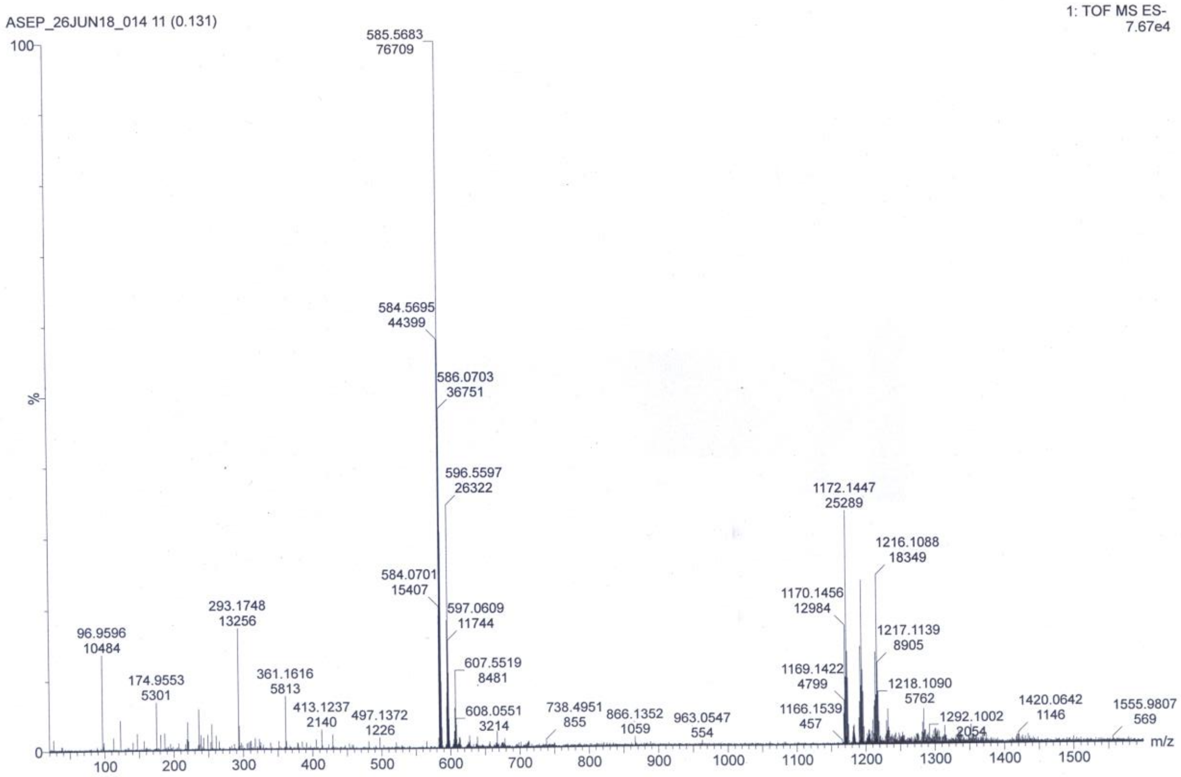

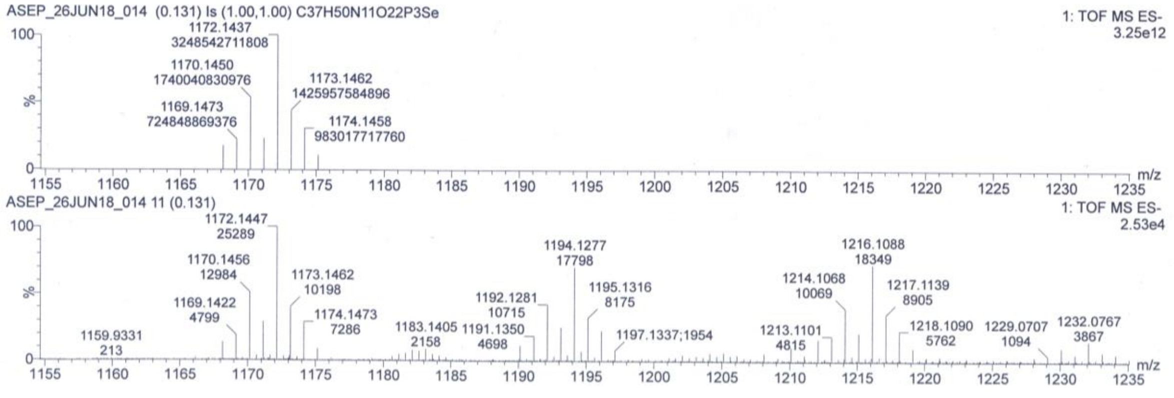

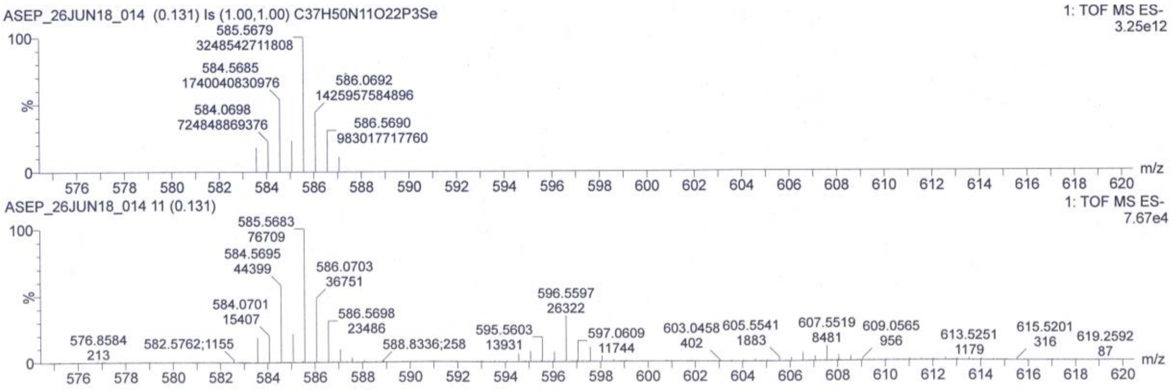

Structural analysis for ODN 4

```
****************************************************************************
   3DNA v2.4.3-2019apr06, created and maintained by xiangjun@x3dna.org
****************************************************************************
1. The list of the parameters given below correspond to the 5' to 3' direction
   of strand I and 3' to 5' direction of strand II.

2. All angular parameters, except for the phase angle of sugar pseudo-
   rotation, are measured in degrees in the range of [-180, +180], and all
   displacements are measured in Angstrom units.
****************************************************************************
File name: GSeT5_7symmAssign_refine_32_3DNA_9.pdb
Date and time: Tue Jul 16 11:45:39 2019

Number of base-pairs: 10
Number of atoms: 735
****************************************************************************
****************************************************************************
RMSD of the bases (----- for WC bp, + for isolated bp, x for helix change)

            Strand I                    Strand II          Helix
   1   (0.021) ....>A:...1_:[.DG]G-----C[.DC]:..10_:B<.... (0.003)     |
   2   (0.023) ....>A:...2_:[.DT]T-----A[.DA]:...9_:B<.... (0.005)     |
   3   (0.003) ....>A:...3_:[.DC]C-----G[.DG]:...8_:B<.... (0.003)     |
   4   (0.002) ....>A:...4_:[.DC]C-----G[.DG]:...7_:B<.... (0.004)     |
   5   (0.003) ....>A:...5_:[.DC]C-----G[.DG]:...6_:B<.... (0.004)     |
   6   (0.004) ....>A:...6_:[.DG]G-----C[.DC]:...5_:B<.... (0.003)     |
   7   (0.004) ....>A:...7_:[.DG]G-----C[.DC]:...4_:B<.... (0.003)     |
   8   (0.004) ....>A:...8_:[.DG]G-----C[.DC]:...3_:B<.... (0.003)     |
   9   (0.005) ....>A:...9_:[.DA]A-----T[.DT]:...2_:B<.... (0.005)     |
  10   (0.003) ....>A:..10_:[.DC]C-----G[.DG]:...1_:B<.... (0.018)     |
****************************************************************************
Detailed H-bond information: atom-name pair and length [ O N]
   1 G-----C  [3]  O6 - N4  2.94  N1 - N3  2.99  N2 - O2  2.97
   2 T-----A  [2]  N3 - N1  3.15  O4 - N6  3.44
   3 C-----G  [3]  O2 - N2  2.59  N3 - N1  2.77  N4 - O6  2.91
   4 C-----G  [3]  O2 - N2  2.71  N3 - N1  2.75  N4 - O6  2.72
   5 C-----G  [3]  O2 - N2  2.64  N3 - N1  2.78  N4 - O6  2.86
   6 G-----C  [3]  O6 - N4  2.95  N1 - N3  2.77  N2 - O2  2.67
   7 G-----C  [3]  O6 - N4  2.74  N1 - N3  2.66  N2 - O2  2.52
   8 G-----C  [3]  O6 - N4  2.85  N1 - N3  2.69  N2 - O2  2.60
   9 A-----T  [2]  N6 - O4  3.21  N1 - N3  3.21
  10 C-----G  [3]  O2 - N2  2.81  N3 - N1  3.18  N4 - O6  3.47
****************************************************************************
Overlap area in Angstrom^2 between polygons defined by atoms on successive
bases. Polygons projected in the mean plane of the designed base-pair step.

Values in parentheses measure the overlap of base ring atoms only. Those
outside parentheses include exocyclic atoms on the ring. Intra- and
inter-strand overlap is designated according to the following diagram:

                    i2  3'      5' j2
                       /|\      |
                        |       |
               Strand I |       | II
                        |       |
                        |       |
                        |      \|/
                    i1  5'      3' j1

     step      i1-i2        i1-j2        j1-i2        j1-j2        sum
   1 GT/AC  7.20( 2.51)  0.00( 0.00)  0.00( 0.00)  4.23( 2.60) 11.42( 5.11)
```

2 TC/GA	2.20(0.46)	0.00(0.00)	0.00(0.00)	4.79(1.94)	6.98(2.41)
3 CC/GG	0.00(0.00)	0.00(0.00)	0.82(0.00)	3.05(1.53)	3.86(1.53)
4 CC/GG	0.09(0.00)	0.00(0.00)	0.17(0.00)	4.31(3.16)	4.58(3.16)
5 CG/CG	0.02(0.00)	0.00(0.00)	4.52(1.66)	0.01(0.00)	4.55(1.66)
6 GG/CC	4.04(2.65)	0.00(0.00)	0.63(0.00)	0.00(0.00)	4.67(2.65)
7 GG/CC	3.93(2.49)	0.00(0.00)	0.85(0.00)	0.00(0.00)	4.78(2.49)
8 GA/TC	3.95(1.87)	0.00(0.00)	0.00(0.00)	0.51(0.00)	4.46(1.88)
9 AC/GT	5.96(4.39)	0.00(0.00)	0.00(0.00)	7.11(2.23)	13.07(6.62)

****

Origin (Ox, Oy, Oz) and mean normal vector (Nx, Ny, Nz) of each base-pair in
the coordinate system of the given structure

bp	Ox	Oy	Oz	Nx	Ny	Nz
1 G-C	25.035	-9.032	16.616	-0.728	0.426	-0.537
2 T-A	22.950	-7.082	15.648	-0.669	0.438	-0.601
3 C-G	21.429	-5.222	13.742	-0.626	0.440	-0.644
4 C-G	20.556	-2.104	11.148	-0.591	0.317	-0.741
5 C-G	19.933	-0.383	8.217	-0.507	0.194	-0.840
6 G-C	19.332	-0.339	4.506	-0.570	-0.090	-0.817
7 G-C	18.401	-2.488	1.448	-0.615	-0.220	-0.758
8 G-C	16.822	-5.666	-0.483	-0.652	-0.401	-0.643
9 A-T	15.091	-7.759	-2.236	-0.720	-0.290	-0.631
10 C-G	12.006	-8.574	-2.704	-0.793	-0.246	-0.557

****

Local base-pair parameters

bp	Shear	Stretch	Stagger	Buckle	Propeller	Opening
1 G-C	-0.46	-0.10	-0.25	-15.11	-11.07	-1.54
2 T-A	-0.82	0.19	0.09	1.36	-12.16	4.08
3 C-G	0.42	-0.24	-0.26	13.22	-14.21	3.28
4 C-G	-0.23	-0.24	-0.11	3.54	-9.42	-0.98
5 C-G	0.24	-0.28	-0.61	6.34	-10.01	3.20
6 G-C	-0.37	-0.26	0.03	-7.68	-20.80	2.29
7 G-C	-0.15	-0.35	0.15	-11.70	-12.52	0.28
8 G-C	-0.64	-0.39	-0.29	-15.53	-20.35	3.30
9 A-T	0.21	0.17	0.55	-10.13	-5.58	-2.93
10 C-G	1.03	-0.01	0.28	11.61	1.22	7.66
ave.	-0.08	-0.15	-0.04	-2.41	-11.49	1.86
s.d.	0.56	0.21	0.33	10.92	6.47	3.16

****

Local base-pair step parameters

step	Shift	Slide	Rise	Tilt	Roll	Twist
1 GT/AC	0.07	-0.97	2.85	-2.61	4.36	27.62
2 TC/GA	0.35	-0.59	2.99	-0.05	3.45	40.88
3 CC/GG	-0.11	-2.19	3.52	-3.75	8.40	24.42
4 CC/GG	-0.32	-1.47	3.11	3.08	9.80	34.12
5 CG/CG	-0.01	-1.52	3.44	-2.18	16.63	30.13
6 GG/CC	0.65	-1.88	3.30	1.35	8.49	32.94
7 GG/CC	0.74	-2.12	3.36	7.80	9.74	24.79
8 GA/TC	-0.33	-1.07	3.03	-6.04	4.45	39.76
9 AC/GT	0.98	-1.19	2.83	2.72	5.87	31.44
ave.	0.23	-1.44	3.16	0.04	7.91	31.79
s.d.	0.48	0.54	0.25	4.21	4.06	5.88

****

Local base-pair helical parameters

step	X-disp	Y-disp	h-Rise	Incl.	Tip	h-Twist
1 GT/AC	-2.88	-0.66	2.65	9.04	5.40	28.08
2 TC/GA	-1.18	-0.51	2.93	4.92	0.08	41.02
3 CC/GG	-7.09	-0.76	2.62	19.02	8.49	26.07
4 CC/GG	-3.67	0.92	2.57	16.25	-5.10	35.59
5 CG/CG	-5.02	-0.30	2.30	29.31	3.83	34.39
6 GG/CC	-4.48	-0.91	2.77	14.66	-2.33	34.02

7 GG/CC	-6.65	0.23	2.49	21.07	-16.88	27.71
8 GA/TC	-2.00	-0.15	2.92	6.48	8.79	40.43
9 AC/GT	-3.05	-1.35	2.65	10.70	-4.95	32.08

~~~~~~

ave.	-4.00	-0.39	2.65	14.60	-0.30	33.26
s.d.	2.00	0.67	0.20	7.80	8.17	5.36

****

The 'simple' parameters are intuitive for non-Watson-Crick base pairs
and associated base-pair steps (where the above corresponding 3DNA
parameters often appear cryptic). Note that they are for structural
description only, not to be fed into the 'rebuild' program. See URL
http://x3dna.org/highlights/details-on-the-simple-base-pair-parameters
and related blogposts on the 3DNA home page for details.

This structure contains 0 non-Watson-Crick (with leading *) base pair(s)

Simple base-pair parameters based on RC8--YC6 vectors

bp	Shear	Stretch	Stagger	Buckle	Propeller	Opening
1 G-C	-0.46	-0.08	-0.25	-15.49	-10.54	-1.54
2 T-A	-0.80	0.26	0.09	0.31	-12.23	4.06
3 C-G	0.43	-0.23	-0.26	13.65	-13.79	3.27
4 C-G	-0.23	-0.24	-0.11	3.27	-9.51	-0.98
5 C-G	0.25	-0.28	-0.61	6.52	-9.90	3.19
6 G-C	-0.38	-0.25	0.03	-8.30	-20.56	2.26
7 G-C	-0.15	-0.35	0.15	-11.77	-12.46	0.28
8 G-C	-0.66	-0.35	-0.29	-16.53	-19.54	3.28
9 A-T	0.20	0.18	0.55	-9.98	-5.85	-2.94
10 C-G	1.03	0.10	0.28	11.43	2.37	7.70

~~~~~~

ave.	-0.08	-0.12	-0.04	-2.69	-11.20	1.86
s.d.	0.56	0.23	0.33	11.15	6.53	3.16

Simple base-pair step parameters based on consecutive C1'-C1' vectors

step	Shift	Slide	Rise	Tilt	Roll	Twist
1 GT/AC	0.01	-0.98	2.85	-2.35	4.51	30.12
2 TC/GA	0.34	-0.60	2.99	0.03	3.45	34.30
3 CC/GG	-0.09	-2.20	3.52	-3.81	8.38	27.57
4 CC/GG	-0.32	-1.47	3.11	3.07	9.80	31.67
5 CG/CG	-0.02	-1.52	3.44	-2.10	16.64	33.14
6 GG/CC	0.61	-1.89	3.30	1.52	8.46	31.76
7 GG/CC	0.68	-2.14	3.36	8.09	9.50	27.11
8 GA/TC	-0.34	-1.06	3.03	-5.98	4.54	35.59
9 AC/GT	1.05	-1.13	2.83	2.35	6.03	26.98

~~~~~~

ave.	0.21	-1.44	3.16	0.09	7.92	30.92
s.d.	0.48	0.55	0.25	4.23	4.02	3.19

****

Structure classification:

This is a right-handed nucleic acid structure

****

lambda: virtual angle between C1'-YN1 or C1'-RN9 glycosidic bonds and the
base-pair C1'-C1' line

C1'-C1': distance between C1' atoms for each base-pair
RN9-YN1: distance between RN9-YN1 atoms for each base-pair
RC8-YC6: distance between RC8-YC6 atoms for each base-pair

bp	lambda(I)	lambda(II)	C1'-C1'	RN9-YN1	RC8-YC6
1 G-C	53.2	53.8	10.7	9.0	9.8
2 T-A	53.8	60.2	10.7	9.1	10.2
3 C-G	55.9	53.9	10.4	8.7	9.7
4 C-G	50.5	55.4	10.7	8.9	9.8
~~~~~~

5 C-G	55.4	54.5	10.5	8.8	9.7
6 G-C	53.4	54.7	10.5	8.8	9.7
7 G-C	54.1	53.4	10.4	8.7	9.6
8 G-C	52.5	56.5	10.3	8.6	9.6
9 A-T	54.4	52.2	11.0	9.2	10.1
10 C-G	62.0	52.9	10.5	8.9	10.0

****

Classification of each dinucleotide step in a right-handed nucleic acid structure: A-like; B-like; TA-like, or other cases.

step	Xp	Yp	Zp	XpH	YpH	ZpH	Form
1 GT/AC	-1.50	9.16	1.61	-4.33	8.80	3.06	A
2 TC/GA	-1.87	8.69	1.90	-2.98	8.51	2.59	A
3 CC/GG	-1.90	8.72	2.39	-8.72	7.51	5.00	A
4 CC/GG	-1.55	8.36	2.37	-5.06	7.43	4.50	A
5 CG/CG	-1.73	8.37	2.33	-6.52	6.27	6.00	A
6 GG/CC	-1.86	8.55	2.41	-6.12	7.71	4.40	A
7 GG/CC	-2.03	8.77	2.11	-8.16	7.51	4.79	A
8 GA/TC	-1.66	8.46	2.37	-3.61	8.16	3.20	A
9 AC/GT	-1.53	9.01	2.36	-4.44	8.44	3.94	A

****

Minor and major groove widths: direct P-P distances and refined P-P distances which take into account the directions of the sugar-phosphate backbones

(Subtract 5.8 Angstrom from the values to take account of the vdw radii of the phosphate groups, and for comparison with FreeHelix and Curves.)

Ref: M. A. El Hassan and C. R. Calladine (1998). ``Two Distinct Modes of Protein-induced Bending in DNA.'' J. Mol. Biol., v282, pp331-343.

	Minor Groove		Major Groove	
	P-P	Refined	P-P	Refined
1 GT/AC	---	---	---	---
2 TC/GA	---	---	---	---
3 CC/GG	16.9	---	17.9	---
4 CC/GG	17.0	15.6	17.1	11.3
5 CG/CG	17.2	15.9	16.4	10.4
6 GG/CC	16.9	15.5	16.7	11.2
7 GG/CC	16.7	---	17.4	---
8 GA/TC	---	---	---	---
9 AC/GT	---	---	---	---

****

Global linear helical axis defined by equivalent C1' and RN9/YN1 atom pairs
Deviation from regular linear helix: 2.55(0.44)
Helix: -0.7261 0.0437 -0.6862
HETATM 9998 XS X X 999 27.423 -5.520 14.526
HETATM 9999 XE X X 999 10.836 -4.522 -1.149
Average and standard deviation of helix radius:
P: 9.51(0.62), O4': 9.10(0.59), C1': 8.52(0.56)

Global parameters based on C1'-C1' vectors:

disp.: displacement of the middle C1'-C1' point from the helix
angle: inclination between C1'-C1' vector and helix (subtracted from 90)
twist: helical twist angle between consecutive C1'-C1' vectors
rise: helical rise by projection of the vector connecting consecutive C1'-C1' middle points onto the helical axis

bp	disp.	angle	twist	rise
1 G-C	7.22	15.20	31.53	2.78
2 T-A	6.86	19.43	36.51	2.48
3 C-G	6.11	18.42	28.92	2.13
4 C-G	6.23	14.22	32.87	2.63

5 C-G	7.36	11.09	34.72	2.73
6 G-C	7.48	8.94	32.62	2.48
7 G-C	6.55	14.74	28.76	2.21
8 G-C	6.18	19.43	37.95	2.15
9 A-T	7.07	19.16	28.31	3.25
10 C-G	7.37	17.53	---	---

****

Main chain and chi torsion angles:

Note: alpha: O3'(i-1)-P-O5'-C5'
beta: P-O5'-C5'-C4'
gamma: O5'-C5'-C4'-C3'
delta: C5'-C4'-C3'-O3'
epsilon: C4'-C3'-O3'-P(i+1)
zeta: C3'-O3'-P(i+1)-O5'(i+1)

chi for pyrimidines(Y): O4'-C1'-N1-C2
chi for purines(R): O4'-C1'-N9-C4

Strand I

base	alpha	beta	gamma	delta	epsilon	zeta	chi
1 G	---	---	156.5	108.8	-116.7	-59.3	-163.8
2 T	-68.7	140.9	66.4	71.3	-143.6	-65.6	-163.5
3 C	-70.7	165.6	59.7	81.8	-139.9	-93.5	-152.5
4 C	80.4	-143.5	-124.4	95.5	-127.7	-76.2	-173.5
5 C	-65.6	169.5	48.0	81.3	-151.8	-71.4	-157.0
6 G	-70.3	171.3	58.6	78.3	-130.1	-89.0	-167.8
7 G	81.1	-150.9	-114.7	101.1	-138.6	-72.7	-167.2
8 G	102.2	-160.2	-137.3	88.3	-147.4	-72.0	-167.3
9 A	-58.6	177.6	45.3	88.5	-148.9	-59.8	-159.5
10 C	-68.1	178.0	49.6	81.1	---	---	-144.0

Strand II

base	alpha	beta	gamma	delta	epsilon	zeta	chi
1 C	-63.9	171.4	55.9	85.8	---	---	-143.5
2 A	-56.8	177.3	41.9	88.2	-159.6	-68.3	-146.3
3 G	-68.5	173.1	66.9	78.7	-153.2	-80.2	-167.7
4 G	-67.9	166.4	61.5	78.0	-152.3	-68.6	-168.5
5 G	-70.1	176.6	49.1	78.3	-148.4	-70.9	-161.6
6 C	-69.5	-178.7	49.9	80.3	-147.5	-69.8	-157.3
7 C	-71.8	173.8	60.8	79.2	-157.9	-71.2	-163.4
8 C	-75.5	178.4	52.3	81.6	-159.6	-69.1	-147.4
9 T	-57.3	131.9	61.2	78.3	-149.1	-64.1	-167.6
10 G	---	---	153.5	110.7	-119.5	-84.0	-162.5

****

Sugar conformational parameters:

Note: v0: C4'-O4'-C1'-C2'
v1: O4'-C1'-C2'-C3'
v2: C1'-C2'-C3'-C4'
v3: C2'-C3'-C4'-O4'
v4: C3'-C4'-O4'-C1'

tm: the amplitude of pucker
P: the phase angle of pseudorotation

Strand I

base	v0	v1	v2	v3	v4	tm	P	Puckering
1 G	21.7	-30.2	26.4	-14.8	-4.1	29.6	333.1	C2'-exo
2 T	0.6	-25.6	38.4	-39.5	24.9	40.4	17.9	C3'-endo
3 C	-6.0	-17.6	33.0	-37.2	27.5	37.2	27.6	C3'-endo
4 C	18.5	-32.3	33.0	-23.0	3.0	34.0	346.3	C2'-exo
5 C	2.4	-25.8	38.1	-37.5	22.3	39.4	15.1	C3'-endo

6 G	3.8	-28.7	41.2	-39.8	22.8	42.3	13.4	C3'-endo
7 G	23.8	-33.9	30.7	-17.6	-3.7	33.8	335.1	C2'-exo
8 G	12.5	-31.3	37.1	-30.5	11.5	37.1	359.1	C2'-exo
9 A	8.1	-27.0	34.4	-30.4	14.2	34.6	5.2	C3'-endo
10 C	-3.8	-20.1	34.9	-37.9	26.4	38.2	24.1	C3'-endo

Strand II

base	v0	v1	v2	v3	v4	tm	P	Puckering
1 C	-1.8	-19.2	31.6	-33.3	22.3	34.0	21.4	C3'-endo
2 A	12.5	-31.3	37.0	-30.6	11.6	37.0	359.2	C2'-exo
3 G	-6.8	-18.7	35.4	-40.2	29.7	40.1	28.0	C3'-endo
4 G	1.5	-27.1	40.8	-40.8	24.8	42.6	16.4	C3'-endo
5 G	1.7	-26.9	40.5	-40.4	24.5	42.2	16.2	C3'-endo
6 C	2.9	-27.0	39.4	-38.5	22.6	40.7	14.4	C3'-endo
7 C	-5.7	-19.4	35.6	-39.8	28.8	39.9	26.6	C3'-endo
8 C	-0.8	-22.8	36.3	-37.5	24.3	38.6	19.6	C3'-endo
9 T	1.9	-24.6	36.3	-35.7	21.7	37.7	15.4	C3'-endo
10 G	19.6	-28.3	25.9	-15.0	-2.7	28.4	336.0	C2'-exo

****

Same strand P--P and C1'--C1' virtual bond distances

	Strand I			Strand II	
step	P--P	C1'--C1'	step	P--P	C1'--C1'
1 G/T	---	5.30	1 C/A	5.84	5.10
2 T/C	6.99	5.18	2 A/G	6.06	5.69
3 C/C	5.86	5.14	3 G/G	6.03	5.57
4 C/C	6.23	5.86	4 G/G	5.59	5.26
5 C/G	5.77	5.42	5 G/C	5.54	5.61
6 G/G	5.53	5.57	6 C/C	6.03	5.70
7 G/G	6.29	5.43	7 C/C	5.88	5.40
8 G/A	6.59	5.59	8 C/T	7.11	5.44
9 A/C	5.71	5.44	9 T/G	---	5.46

****

Helix radius (radial displacement of P, O4', and C1' atoms in local helix
frame of each dimer)

	Strand I			Strand II		
step	P	O4'	C1'	P	O4'	C1'
1 GT/AC	9.90	8.02	7.38	9.77	8.66	7.88
2 TC/GA	8.82	7.06	6.17	9.23	7.83	6.88
3 CC/GG	10.68	10.54	10.30	12.35	11.80	11.35
4 CC/GG	9.81	9.40	8.70	8.17	8.08	7.48
5 CG/CG	8.51	9.20	8.77	9.58	9.68	9.18
6 GG/CC	9.78	8.75	8.30	9.92	9.72	9.07
7 GG/CC	12.90	11.10	10.72	9.29	10.05	9.65
8 GA/TC	8.58	7.73	6.91	9.30	7.85	7.02
9 AC/GT	8.40	7.23	6.62	10.71	9.64	8.81

****

Position (Px, Py, Pz) and local helical axis vector (Hx, Hy, Hz)
for each dinucleotide step

step	Px	Py	Pz	Hx	Hy	Hz
1 GT/AC	25.93	-6.85	14.40	-0.73	0.27	-0.63
2 TC/GA	22.87	-6.54	13.77	-0.67	0.37	-0.65
3 CC/GG	23.51	-7.38	7.13	-0.84	0.17	-0.52
4 CC/GG	21.84	-3.87	7.80	-0.72	0.06	-0.69
5 CG/CG	18.75	-5.08	6.45	-0.86	0.15	-0.48
6 GG/CC	16.76	-4.22	5.59	-0.76	-0.04	-0.65
7 GG/CC	15.21	-6.27	6.06	-0.90	-0.08	-0.42
8 GA/TC	14.43	-6.31	-0.33	-0.59	-0.27	-0.76
9 AC/GT	12.66	-5.14	-1.91	-0.73	-0.09	-0.68
