# Supplementary material for: Solid-phase synthesis and structural characterisation of phosphoroselenolate-modified DNA: a backbone analogue which does not impose conformational bias and facilitates SAD X-ray crystallography
Source: Chem Sci. 2019 Oct 11;10(47):10948–57. doi: 10.1039/c9sc04098f (PMC7066676; doi:10.1039/c9sc04098f)
Supplement: Supplementary file 2 [file SC-010-C9SC04098F-s002.pdf]

Electronic Supplementary Material (ESI) for Chemical Science.
This journal is © The Royal Society of Chemistry 2019

Preliminary Full wwPDB X-ray Structure Validation Report (i)

Jul 3, 2019 – 04:30 pm BST

Deposition ID : D_1292103206
PDB ID : *(not yet assigned)*

This is a Preliminary Full wwPDB X-ray Structure Validation Report.

This report is produced by the wwPDB Deposition System during initial deposition but before annotation of the structure.

We welcome your comments at *validation@mail.wwpdb.org*
A user guide is available at
https://www.wwpdb.org/validation/2017/XrayValidationReportHelp
with specific help available everywhere you see the (i) symbol.

PRELIMINARY VALIDATION REPORT

The following versions of software and data (see references (i)) were used in the production of this report:

MolProbity	:	4.02b-467
Mogul	:	1.8.0 (224370), CSD as540be (2019)
Xtriage (Phenix)	:	1.13
EDS	:	2.4
Percentile statistics	:	20171227.v01 (using entries in the PDB archive December 27th 2017)
Refmac	:	5.8.0158
CCP4	:	7.0 (Gargrove)
Ideal geometry (proteins)	:	Engh & Huber (2001)
Ideal geometry (DNA, RNA)	:	Parkinson et al. (1996)
Validation Pipeline (wwPDB-VP)	:	2.4

1 Overall quality at a glance (i)

The following experimental techniques were used to determine the structure:
X-RAY DIFFRACTION

The reported resolution of this entry is 1.45 Å.

Percentile scores (ranging between 0-100) for global validation metrics of the entry are shown in the following graphic. The table shows the number of entries on which the scores are based.

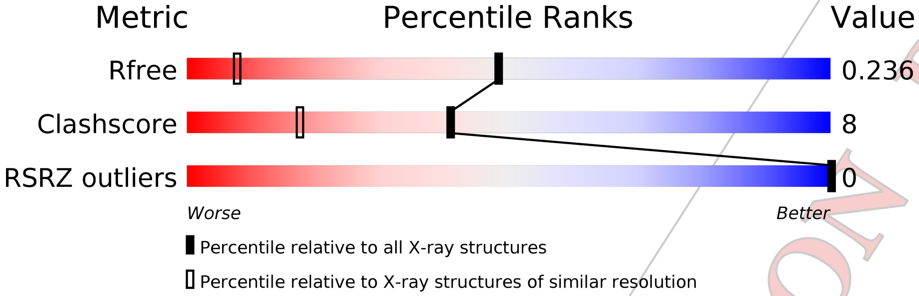

Metric	Whole archive (#Entries)	Similar resolution (#Entries, resolution range(Å))
R_{free}	111664	1761 (1.48-1.44)
Clashscore	122126	1816 (1.48-1.44)
RSRZ outliers	108989	1733 (1.48-1.44)

The table below summarises the geometric issues observed across the polymeric chains and their fit to the electron density. The red, orange, yellow and green segments on the lower bar indicate the fraction of residues that contain outliers for >=3, 2, 1 and 0 types of geometric quality criteria. A grey segment represents the fraction of residues that are not modelled. The numeric value for each fraction is indicated below the corresponding segment, with a dot representing fractions <=5% The upper red bar (where present) indicates the fraction of residues that have poor fit to the electron density. The numeric value is given above the bar.

Mol	Chain	Length	Quality of chain
1	A	10	90% 10%
1	B	10	90% 10%

The following table lists non-polymeric compounds, carbohydrate monomers and non-standard residues in protein, DNA, RNA chains that are outliers for geometric or electron-density-fit criteria:

Mol	Type	Chain	Res	Chirality	Geometry	Clashes	Electron density
4	CL	A	103	-	-	X	-

PRELIMINARY VALIDATION REPORT

2 Entry composition (i)

There are 6 unique types of molecules in this entry. The entry contains 739 atoms, of which 252 are hydrogens and 0 are deuteriums.

In the tables below, the ZeroOcc column contains the number of atoms modelled with zero occupancy, the AltConf column contains the number of residues with at least one atom in alternate conformation and the Trace column contains the number of residues modelled with at most 2 atoms.

- Molecule 1 is a DNA chain called DNA (5'-D(*GP*(XCI)P*CP*CP*CP*GP*GP*GP*AP*C)-3').

Mol	Chain	Residues	Atoms							ZeroOcc	AltConf	Trace
1	A	10	Total 315	C 96	H 113	N 39	O 57	P 9	Se 1	0	0	0
1	B	10	Total 315	C 96	H 113	N 39	O 57	P 9	Se 1	0	0	0

- Molecule 2 is BARIUM ION (three-letter code: BA) (formula: Ba).

Mol	Chain	Residues	Atoms		ZeroOcc	AltConf
2	A	1	Total 1	Ba 1	0	0
2	B	1	Total 1	Ba 1	0	0

- Molecule 3 is SODIUM ION (three-letter code: NA) (formula: Na).

Mol	Chain	Residues	Atoms		ZeroOcc	AltConf
3	A	1	Total 1	Na 1	0	0
3	B	1	Total 1	Na 1	0	0

- Molecule 4 is CHLORIDE ION (three-letter code: CL) (formula: Cl).

Mol	Chain	Residues	Atoms		ZeroOcc	AltConf
4	A	1	Total 1	Cl 1	0	0

- Molecule 5 is SPERMINE (three-letter code: SPM) (formula: $C_{10}H_{26}N_4$).

PRELIMINARY VALIDATION REPORT

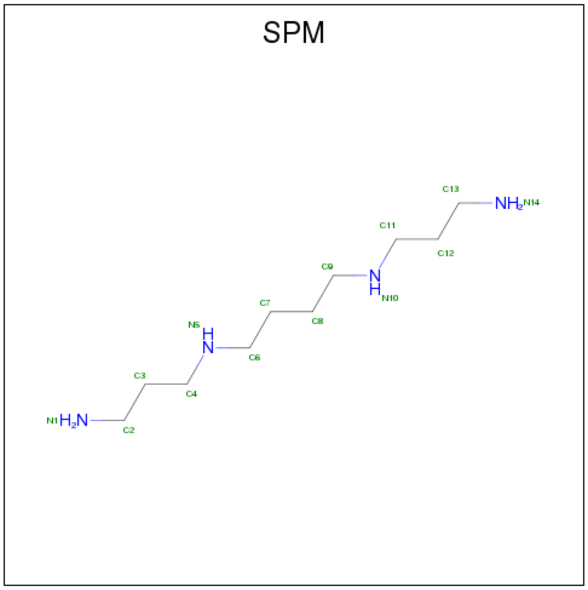

Mol	Chain	Residues	Atoms	ZeroOcc	AltConf
5	B	1	Total C H N<br>40 10 26 4	0	0

- Molecule 6 is water.

Mol	Chain	Residues	Atoms	ZeroOcc	AltConf
6	A	23	Total O<br>26 26	0	3
6	B	37	Total O<br>38 38	0	1

PRELIMINARY VALIDATION REPORT

3 Residue-property plots (i)

These plots are drawn for all protein, RNA and DNA chains in the entry. The first graphic for a chain summarises the proportions of the various outlier classes displayed in the second graphic. The second graphic shows the sequence view annotated by issues in geometryand electron density. Residues are color-coded according to the number of geometric quality criteria for which they contain at least one outlier: green = 0, yellow = 1, orange = 2 and red = 3 or more. A red dot above a residue indicates a poor fit to the electron density (RSRZ > 2). Stretches of 2 or more consecutive residues without any outlier are shown as a green connector. Residues present in the sample, but not in the model, are shown in grey.

- Molecule 1: DNA (5'-D(*GP*(XCI)P*CP*CP*CP*GP*GP*GP*AP*C)-3')

Chain A:
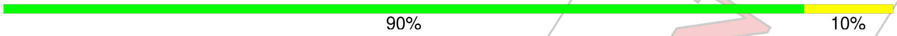

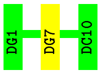

- Molecule 1: DNA (5'-D(*GP*(XCI)P*CP*CP*CP*GP*GP*GP*AP*C)-3')

Chain B:
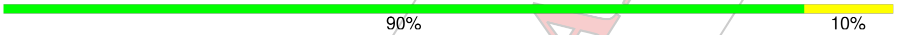

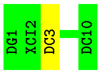

PRELIMINARY VALIDATION REPORT

4 Data and refinement statistics (i)

Property	Value	Source
Space group	P 31 2 1	Depositor
Cell constants<br>a, b, c, α, β, γ	38.64Å 38.64Å 79.31Å<br>90.00° 90.00° 120.00°	Depositor
Resolution (Å)	25.57 – 1.45<br>25.57 – 1.45	Depositor<br>EDS
% Data completeness<br>(in resolution range)	98.3 (25.57-1.45)<br>98.3 (25.57-1.45)	Depositor<br>EDS
R_{merge}	(Not available)	Depositor
R_{sym}	(Not available)	Depositor
$< I/\sigma(I) >$ [1]	1.55 (at 1.45Å)	Xtriage
Refinement program	phenix.refine 1.16_3549, Phenix 1.16_3549	Depositor
R, R_{free}	0.185 , 0.237<br>0.188 , 0.236	Depositor<br>DCC
R_{free} test set	571 reflections (4.56%)	wwPDB-VP
Wilson B-factor (Å^2)	19.2	Xtriage
Anisotropy	0.501	Xtriage
Bulk solvent k_{sol}(e/Å^3), B_{sol}(Å^2)	0.31 , 20.6	EDS
L-test for twinning[2]	$< \|L\| > = 0.41$, $< L^2 > = 0.24$	Xtriage
Estimated twinning fraction	0.470 for -h,-k,l	Xtriage
F_o,F_c correlation	0.97	EDS
Total number of atoms	739	wwPDB-VP
Average B, all atoms (Å^2)	27.0	wwPDB-VP

Xtriage's analysis on translational NCS is as follows: *The largest off-origin peak in the Patterson function is 14.78% of the height of the origin peak. No significant pseudotranslation is detected.*

[1]Intensities estimated from amplitudes.

[2]Theoretical values of $< |L| >$, $< L^2 >$ for acentric reflections are 0.5, 0.333 respectively for untwinned datasets, and 0.375, 0.2 for perfectly twinned datasets.

PRELIMINARY VALIDATION REPORT

5 Model quality (i)

5.1 Standard geometry (i)

Bond lengths and bond angles in the following residue types are not validated in this section: NA, SPM, XCI, BA, CL

The Z score for a bond length (or angle) is the number of standard deviations the observed value is removed from the expected value. A bond length (or angle) with $|Z| > 5$ is considered an outlier worth inspection. RMSZ is the root-mean-square of all Z scores of the bond lengths (or angles).

Mol	Chain	Bond lengths		Bond angles	
		RMSZ	$\#\|Z\| > 5$	RMSZ	$\#\|Z\| > 5$
1	A	0.52	0/203	0.71	0/309
1	B	0.51	0/203	0.82	0/309
All	All	0.51	0/406	0.76	0/618

There are no bond length outliers.

There are no bond angle outliers.

There are no chirality outliers.

There are no planarity outliers.

5.2 Too-close contacts (i)

In the following table, the Non-H and H(model) columns list the number of non-hydrogen atoms and hydrogen atoms in the chain respectively. The H(added) column lists the number of hydrogen atoms added and optimized by MolProbity. The Clashes column lists the number of clashes within the asymmetric unit, whereas Symm-Clashes lists symmetry related clashes.

Mol	Chain	Non-H	H(model)	H(added)	Clashes	Symm-Clashes
1	A	202	113	101	1	0
1	B	202	113	101	2	0
2	A	1	0	0	0	0
2	B	1	0	0	0	0
3	A	1	0	0	0	0
3	B	1	0	0	0	0
4	A	1	0	0	2	0
5	B	14	26	26	1	0
6	A	26	0	0	3	0
6	B	38	0	0	1	0
All	All	487	252	228	5	0

PRELIMINARY VALIDATION REPORT

The all-atom clashscore is defined as the number of clashes found per 1000 atoms (including hydrogen atoms). The all-atom clashscore for this structure is 8.

All (5) close contacts within the same asymmetric unit are listed below, sorted by their clash magnitude.

Atom-1	Atom-2	Interatomic distance (Å)	Clash overlap (Å)
4:A:103:CL:CL	6:A:210[A]:HOH:O	2.46	0.70
1:A:7:DG:OP1	6:A:201:HOH:O	2.12	0.67
4:A:103:CL:CL	6:A:219:HOH:O	2.50	0.66
1:B:3:DC:OP2	6:B:201:HOH:O	2.16	0.60
1:B:3:DC:OP2	5:B:101:SPM:N5	2.52	0.42

There are no symmetry-related clashes.

5.3 Torsion angles (i)

5.3.1 Protein backbone (i)

There are no protein molecules in this entry.

5.3.2 Protein sidechains (i)

There are no protein molecules in this entry.

5.3.3 RNA (i)

There are no RNA molecules in this entry.

5.4 Non-standard residues in protein, DNA, RNA chains (i)

There are no non-standard protein/DNA/RNA residues in this entry.

5.5 Carbohydrates (i)

There are no carbohydrates in this entry.

PRELIMINARY VALIDATION REPORT

5.6 Ligand geometry (i)

Of 6 ligands modelled in this entry, 5 are monoatomic - leaving 1 for Mogul analysis.

In the following table, the Counts columns list the number of bonds (or angles) for which Mogul statistics could be retrieved, the number of bonds (or angles) that are observed in the model and the number of bonds (or angles) that are defined in the Chemical Component Dictionary. The Link column lists molecule types, if any, to which the group is linked. The Z score for a bond length (or angle) is the number of standard deviations the observed value is removed from the expected value. A bond length (or angle) with $|Z| > 2$ is considered an outlier worth inspection. RMSZ is the root-mean-square of all Z scores of the bond lengths (or angles).

Mol	Type	Chain	Res	Link	Bond lengths			Bond angles		
					Counts	RMSZ	$\#\|Z\| > 2$	Counts	RMSZ	$\#\|Z\| > 2$
5	SPM	B	101	-	13,13,13	0.34	0	12,12,12	0.75	0

In the following table, the Chirals column lists the number of chiral outliers, the number of chiral centers analysed, the number of these observed in the model and the number defined in the Chemical Component Dictionary. Similar counts are reported in the Torsion and Rings columns. '-' means no outliers of that kind were identified.

Mol	Type	Chain	Res	Link	Chirals	Torsions	Rings
5	SPM	B	101	-	-	5/11/11/11	-

There are no bond length outliers.

There are no bond angle outliers.

There are no chirality outliers.

All (5) torsion outliers are listed below:

Mol	Chain	Res	Type	Atoms
5	B	101	SPM	C7-C6-N5-C4
5	B	101	SPM	C7-C8-C9-N10
5	B	101	SPM	C6-C7-C8-C9
5	B	101	SPM	C2-C3-C4-N5
5	B	101	SPM	C8-C9-N10-C11

There are no ring outliers.

1 monomer is involved in 1 short contact:

Mol	Chain	Res	Type	Clashes	Symm-Clashes
5	B	101	SPM	1	0

PRELIMINARY VALIDATION REPORT

5.7 Other polymers (i)

There are no such residues in this entry.

5.8 Polymer linkage issues (i)

There are no chain breaks in this entry.

PRELIMINARY VALIDATION REPORT

6 Fit of model and data (i)

6.1 Protein, DNA and RNA chains (i)

In the following table, the column labelled '#RSRZ> 2' contains the number (and percentage) of RSRZ outliers, followed by percent RSRZ outliers for the chain as percentile scores relative to all X-ray entries and entries of similar resolution. The OWAB column contains the minimum, median, 95th percentile and maximum values of the occupancy-weighted average B-factor per residue. The column labelled 'Q< 0.9' lists the number of (and percentage) of residues with an average occupancy less than 0.9.

Mol	Chain	Analysed	<RSRZ>	#RSRZ>2			OWAB(Å^2)	Q<0.9
1	A	9/10 (90%)	-0.22	0	100	100	22, 23, 28, 29	0
1	B	9/10 (90%)	-0.24	0	100	100	23, 24, 25, 25	0
All	All	18/20 (90%)	-0.23	0	100	100	22, 24, 28, 29	0

There are no RSRZ outliers to report.

6.2 Non-standard residues in protein, DNA, RNA chains (i)

There are no non-standard protein/DNA/RNA residues in this entry.

6.3 Carbohydrates (i)

There are no carbohydrates in this entry.

6.4 Ligands (i)

In the following table, the Atoms column lists the number of modelled atoms in the group and the number defined in the chemical component dictionary. The B-factors column lists the minimum, median, 95th percentile and maximum values of B factors of atoms in the group. The column labelled 'Q< 0.9' lists the number of atoms with occupancy less than 0.9.

Mol	Type	Chain	Res	Atoms	RSCC	RSR	B-factors(Å^2)	Q<0.9
5	SPM	B	101	14/?	0.81	0.15	36,43,44,44	0
3	NA	B	103	1/?	0.93	0.11	37,37,37,37	0
3	NA	A	102	1/?	0.96	0.14	33,33,33,33	0
4	CL	A	103	1/?	0.99	0.12	22,22,22,22	0
2	BA	B	102	1/?	1.00	0.06	21,21,21,21	0
2	BA	A	101	1/?	1.00	0.02	22,22,22,22	0

PRELIMINARY VALIDATION REPORT

6.5 Other polymers (i)

There are no such residues in this entry.

PRELIMINARY VALIDATION REPORT
